# Genome-Wide Identification of the ASMT Gene Family and Expression Analysis of Wheat ASMTs Under Abiotic and Biotic Stress

**DOI:** 10.3390/biology15161430

**Published:** 2026-08-19

**Authors:** Baoyue Cui, Tianle Ji, Peisen Su, Jun Yan

**Affiliations:** 1 College of Information Science and Engineering, Shandong Agricultural University, Tai’an 271018, China; c897729777@163.com (B.C.); a1464002760@163.com (T.J.); 2 College of Agriculture and Biology, Liaocheng University, Liaocheng 252059, China; 3 Key Laboratory of Huang-Huai-Hai Smart Agricultural Technology, Ministry of Agriculture and Rural Affairs, Tai’an 271018, China

**Keywords:** wheat, ASMT, evolution, biotic and abiotic stresses, expression analysis

## Abstract

Melatonin is an important stress-protective agent in plant growth. Methyltransferase (ASMT) is an important enzyme in the concluding phase of melatonin production in plants. In this study, we performed the genome-wide identification and functional investigation of the *ASMT* gene family in hexaploid wheat and 14 other plants. *ASMT* genes in 15 plants were identified by using HMM scanning against the proteomes derived from a single representative reference genome for each species. They were classified into three subfamilies I-III by constructing four types of phylogenetic trees (Neighbour-joining with p-distance model, Neighbour-joining with JTT model, Maximum likelihood, and Bayesian inference). Based on exon–intron structure and domain diagrams, a conserved structural pattern characterized by successive intron phases 1 and 0 (the “1-0” pattern) was found in *ASMT* genes during evolution. Collinear events analysis indicated that polyploidization and tandem duplication synergistically promote the expansion of *T. aestivum ASMT* members. Cis-acting element analysis revealed that numerous stress- and hormone-responsive motifs (such as ABRE and LTR) were present in *ASMT*s of wheat, suggesting a role for ASMTs in adaptive signal transduction. Transcriptome analysis revealed that specific *T. aestivum ASMT* genes were strongly responsive to stress; for instance, *II_TraesCS2B02G041200* and *II_TraesCS2B02G606200* were strongly upregulated under drought and salt stress, respectively. To independently confirm the stress responsiveness of these candidates in a different genetic background, we performed quantitative real-time PCR (qRT-PCR) on selected genes under drought and salt treatments. The result showed that the expression trends of transcriptome and qRT-PCR were almost the same, identifying these *ASMT* genes as strong stress-responsive candidates under drought and salt treatments. In this study, we performed identification, classification, evolution analysis and expression pattern analysis of *ASMT*s in wheat and 14 other plants. Our study will provide a framework for the targeted genetic improvement of melatonin-mediated stress resistance.

## Simple Summary

Wheat is a vital global food source, but its production is severely threatened by environmental challenges such as drought and salty soils. To survive these harsh conditions, plants naturally produce a protective compound called melatonin. The goal of this study was to discover and understand the specific genes responsible for the final step of producing melatonin in wheat. By analyzing the entire genetic instructions of wheat and related plants, we identified one hundred and fifty-six genes potentially associated with this biological process. We traced how these genes evolved and multiplied over millions of years. Importantly, our experiments revealed that a select few of these genes become highly active when wheat is exposed to drought and salty conditions. These findings provide a clear genetic map of how wheat defends itself against environmental stress. Ultimately, agricultural scientists and breeders can use this knowledge to develop stronger, more resilient wheat varieties, which is a crucial step toward ensuring a stable global food supply in the face of ongoing climate change.

## 1. Introduction

Melatonin (N-acetyl-5-methoxytryptamine) is a prominent indoleamine neurohormone that modulates physiological processes and participates in various biological functions. Melatonin was first discovered in the pineal gland of vertebrates and was found in nine different plant species through further research [1]. Recent research has reported that many plants contain melatonin [2]. These studies showed that melatonin, as an antioxidant, is involved in multiple stress response processes in plants. In a drought stress experiment of apples (*Malus domestica* Borkh.), continuous exogenous application of 100 μM melatonin effectively alleviated oxidative stress and delayed leaf senescence [3]. In the cold stress study of *Arabidopsis thaliana*, melatonin treatment (10–30 μM) significantly increased the plant biomass, primary root length and plant height [4]. Subsequent research on the physiological responses of cucumbers (*Cucumis sativus* L.) to waterlogging stress validated the notion that melatonin improves the drought resistance of cucumbers through many synergistic mechanisms [5]. Melatonin is essential for the abiotic stress resistance of monocotyledonous plants and wheat [6,7]. The production of melatonin in higher plants is similar to that in animals, consisting of four enzymatic stages mediated by at least six enzymes, including serotonin N-acetyltransferase (SNAT), N-acetylserotonin methyltransferase (ASMT), tryptophan decarboxylase (TDC), tryptophan hydroxylase (TPH), and tryptamine 5-hydroxylase (T5H) [8]. In the final biosynthesis stage, ASMT catalyzes the O-methylation of N-acetylserotonin, making it an essential terminal catalyst for melatonin generation [9].

The *ASMT* gene family has been identified in a variety of plants, including Arabidopsis (*Arabidopsis thaliana*), tea plant (*Camellia sinensis*), pepper (*Capsicum annuum* L.), apple (*Malus domestica*), tomato (*Solanum lycopersicum*), banana (*Musa nana* Lour), poplar (*Populus tomentosa* Carr.), and Walnut (*Juglans regia*) [10,11,12,13,14,15,16,17]. There was a considerable amount of research on ASMT in response to abiotic stress in plants. An *ASMT* gene was cloned from apple rootstock (*Malus zumi* Mats). Transgenic Arabidopsis plants with overexpression of the *MzASMT1* gene have improved efficiency in eliminating reactive oxygen species (ROS) and enhanced drought tolerance [10]. Thirty-seven *MdASMT* genes were identified in the apple (*Malus domestica*) genome, among which *MdASMT11* and *MdASMT14* play key roles in root-mediated pathways and influence various stress resistances [13]. Twenty-four *MaASMT* genes were identified in bananas (*Musa nana* Lour). *MaASMT10-1* and *MaASMT4-2*, as the intersection of cold stress and melatonin signaling, enhance the cold adaptability of bananas by activating the hormone pathway (ABA/SA) [15]. A recent study in poplar further solidified this link, demonstrating that the *PtoASMT* gene is induced by drought, ABA, and jasmonic acid (JA), and that its overexpression increases endogenous melatonin levels, thereby conferring enhanced tolerance to drought stress [16]. In rice (*Oryza sativa*), a key model monocot, the simultaneous overexpression of *OsASMT1*, *OsASMT2*, and *OsASMT3* was shown to enhance both enzyme activity and drought resistance [18]. Consequently, these results demonstrate that the ASMT gene family is crucial to the resistance of plants to environmental abiotic stress.

Wheat (*T. aestivum*) is a globally crucial food crop and a significant source of starch and energy [19]. Wheat production is greatly impacted by many abiotic stresses, including low temperatures, drought, and salt stress, which significantly affect morphological and biochemical traits [20,21,22,23]. Regarding the biological functions of wheat melatonin, current research mainly focuses on how exogenous melatonin can increase wheat yield and stress resistance under adverse conditions [24,25]. However, systematic studies on the genetic mechanisms of melatonin synthesis in wheat are limited. The genome-wide identification and characterization of stress-related gene families have proven to be effective strategies for understanding stress adaptation in various crops, such as *Brassica* and potato [26,27,28]. The public release of the wheat reference genome provides an excellent foundation to bridge this knowledge gap by systematically identifying melatonin-related genes through bioinformatics analysis. Recently, the functions and genomic features of *ASMT* genes in wheat have begun to attract significant attention. For instance, Shamloo-Dashtpagerdi et al. (2023) unraveled the regulatory role of the MYC2 transcription factor on *ASMT* gene expression in wheat under drought stress [29]. Furthermore, a recent massive pan-genomic study by Liu and Yu (2026) identified *ASMT/COMT* genes across over a thousand plant species to explore their copy number variations and dosage balance, which included datasets from wheat [30]. Despite these remarkable advancements, a systematic and detailed genome-wide characterization specifically focusing on the structural conservation, collinearity evolution, and comprehensive transcriptomic profiling of the entire *ASMT* gene family under diverse abiotic and biotic stresses in hexaploid wheat remains highly necessary. To address this gap, the specific aims of this study were to use bioinformatics methods to identify the members of the wheat *ASMT* gene family and to analyze their classification, intron–exon structures, collinearity, and promoter cis-regulatory elements (CREs). We also sought to propose an evolutionary model of *ASMTs* based on gene structure analysis. Furthermore, we aimed to study the expression pattern of wheat *ASMT*s under abiotic and biotic stress treatments using public transcriptomic data, and to provide independent experimental confirmation by selecting key wheat *ASMT* genes for qRT-PCR under drought and salt treatments.

This study provides a theoretical basis for exploring the molecular mechanism and function of melatonin in enhancing wheat’s resistance to abiotic stress, and it also offers new ideas for the functional research of the wheat *ASMT* gene family.

## 2. Materials and Methods

### 2.1. Identification and Classification of ASMTs in Wheat and Other Plants

The Genome and proteome data of *T. aestivum* were obtained from Ensembl Plant release-59 (http://plants.ensembl.org/). The Genome and proteome data of the other fourteen investigated plants, including *Triticum_dicoccoides*, *Triticum_turgidum*, *Triticum_spelta*, *Brachypodium_distachyon*, *Aegilops_tauschii*, *Triticum_urartu*, *Vitis_vinifera*, *A. thaliana*, *Oryza_sativa*, *Zea_mays*, *Amborella_trichopoda*, *Selaginella_moellendorffii*, *Physcomitrium_patens*, and *Chlamydomonas_reinhardtii*, were also downloaded from Ensembl Plant release-59 (http://plants.ensembl.org/). To identify *ASMT*s, we initially scanned the proteomes of all 15 plants in batches using HMMER 3.1 based on the broad O-methyltransferase domain (Pfam profile PF00891) with an E-value of 0.01 in our local server and website Pfam 35.0 (https://www.ebi.ac.uk/interpro/download/Pfam/ (accessed on 16 August 2026)). Atypical sequences with an O-methyltransferase domain coverage of less than 50% were excluded to remove partial sequences and fragments. To further ensure the accurate identification of true *ASMT*s, we performed a strict independent verification step. All putative sequences were subjected to reciprocal BLASTP (version 2.13.0) searches against functionally characterized, true ASMT proteins from *Oryza sativa* (e.g., OsASMT1/2/3). Furthermore, the structural identity was cross-verified through our comprehensive phylogenetic clustering (as detailed below), which ensured that the final 156 *TaeASMT* candidates tightly co-clustered with the established *ASMT* lineages rather than other related outgroups. This rigorous, multi-step pipeline is a widely adopted practice for ensuring the high fidelity and functional accuracy of the target gene family dataset.

The alignment of truncated sequences in the O-methyltransferase domain was performed by ClustalW v2.0 [31]. Neighbour-joining (NJ) phylogenetic trees, with models (p-distance and JTT) and 1000 bootstrap repetitions, were constructed by using MEGA 11.0 [32]. A maximum likelihood (ML) phylogenetic tree with 100 bootstrap repetitions was constructed by using PhyML v3.1 [33]. The suitable model for the ML method (including model parameters) was calculated by using ProtTest v3.4 in Akaike information criterion (AIC) [34]. A Bayesian phylogenetic tree was constructed by using MrBayes v3.2.7 with a mixed amino acid substitution model and a Markov Chain Monte Carlo (MCMC) chain of 10,000,000 [35]. ‘Burn-in’ trees were removed from the top 25% of trees in the Markov chain, which was sampled every 100 generations. The results of MrBayes v3.2.7 were analyzed using MEGA 11.0. The use of multiple, distinct algorithms (distance-based, maximum likelihood, and Bayesian) was intended to ensure the robustness and reliability of the final phylogenetic topology and the resulting subfamily classifications. While all four methods yielded highly consistent topologies, the Neighbour-Joining (NJ) tree based on the JTT model was ultimately utilized as the primary reference tree for downstream classification and visualization. Four phylogenetic trees were constructed on our local server.

### 2.2. Domain and Intron–Exon Structure Diagrams of ASMTs

The intron–exon structure diagrams of *ASMT*s in 15 plants were generated by our own R (version 4.3.3) and Perl (version 5.38.2) scripts based on the relevant GFF files from Ensembl Plant release-59 (http://plants.ensembl.org/). The domain diagrams of *ASMT*s in 15 plants were also generated by our own R and Perl scripts based on the Pfam scanning results in our server through the PfamA models from Pfam 37.0 (https://www.ebi.ac.uk/interpro/download/Pfam/, (accessed on 16 August 2026)).

### 2.3. Chromosomal Location, Tandem Duplication and Collinearity Analysis of Wheat ASMTs

Based on the GFF files from Ensembl Plants release-59 (http://plants.ensembl.org/), we generated the chromosomal location map of *T. aestivum ASMT*s by using the GenomePixelizer software (version October_01_2003) [36]. BLAST was performed against *ASMT*s from *O. sativa*, *B. distachyon* and *T. aestivum* with the E value of 1 × 10^−100^. Based on the GFF files and BLAST results, Mcscan software (version 1.0) was used to search for tandem duplications and segmental duplications [37]. The accuracy of this analysis is inherently dependent on the quality and completeness of the input genome annotation (GFF) files. The values of Ka and Ks were calculated by using the “add_ka_and_ks_to_collinearity.pl” from the MCScanX software (version 1.0). Based on the GFF files and MCScanX results, collinearity plots between *O. sativa*, *B. distachyon* and *T. aestivum* were generated using the visualization functions of the TBtools program (version 2.096) [38]. Mapchat v2.3 (https://www.wur.nl/en/show/Mapchart.htm) (accessed on 16 August 2026) was used to create the tandemly duplicated *ASMT* chromosomal position map on each chromosome.

### 2.4. Analysis of Cis-Acting Elements of the Wheat ASMTs

We extracted the sequences of each wheat *ASMT* gene spanning 2000 bp upstream of the transcription start site as the promoter region sequence using our Python (version 3.11) script. Prediction and analysis of *cis*-acting elements were conducted using the PlantCARE online website (http://bioinformatics.psb.ugent.be/webtools/plantcare/html/ (accessed on 16 August 2026)) [39]. The distribution heatmap of the *cis*-acting elements of wheat *ASMT* genes was visualised using TBtools (version 2.096) [38].

### 2.5. Bioinformatics Analysis of Public Transcriptomic Expression Data

Public wheat (*T. aestivum*) transcriptome expression dataset was retrieved from the Sequence Read Archive (SRA) from NCBI (https://www.ncbi.nlm.nih.gov/sra, accessed on 16 August 2026). (1) Abiotic stresses: Two wheat drought RNA-seq datasets involved three wheat cultivars, namely ‘Zhengmai1860,’ ‘Zhoumai18,’ and ‘Bainong207’ (BioProject ID: 1077667, BioProject ID: 994277). Three wheat abiotic stress RNA-seq datasets were used, including heat stress (BioProject: 971860), salt stress (BioProject: 936261), and waterlogging stress (BioProject: 1097929). (2) Biotic stresses: Five wheat RNA-seq datasets of biotic stress were used for *Fusarium graminearum* inoculation (BioProject ID: 1183537), stripe rust (BioProject ID: 1006331), powdery mildew (BioProject ID: 914331) and *Puccinia triticina* infection (BioProject IDs: 972750 and 782527).

Fast QC v0.12.1 (https://www.bioinformatics.babraham.ac.uk/projects/fastqc/, accessed on 16 August 2026) was used for quality control evaluation of raw data. High-quality RNA reads were aligned to the wheat (*T. aestivum*) reference genome from Ensembl Plants release-59 (http://plants.ensembl.org/) by using STAR v2.7.11 software [40]. Gene expression counts were calculated using RSEM v1.3.1 software [41]. The raw count data were normalized using the median of ratios method via the DESeq2 R package (version 1.42.0) to determine transcriptome expression levels. All selected public transcriptomic datasets contained at least three biological replicates per condition (Table S1). Given the inherent variability across different public datasets, RNA-seq data were utilized primarily for exploratory trend analysis to identify initial candidate genes, using a primary screening threshold of |log_2_(FoldChange)| > 1. Definitive independent statistical confirmation of stress responsiveness was subsequently provided by our qRT-PCR experiments. The R package Pheatmap was used to create heat maps showing the expression patterns of wheat *ASMTs*.

### 2.6. Plant Material and Stress Treatments

Wheat (*Triticum aestivum* L.) cultivar “SuMai 3” was used in this study and obtained from Liaocheng University. The seedlings were planted in plates and grown at a temperature of 25 °C under a photoperiod of 16/8 h.

At the three-leaf stage, the seedlings of wheat were subjected to drought stress (20% PEG6000) or salt stress (200 mM NaCl). The wheat leaves were harvested at 0 h and 48 h after PEG6000 treatment. The wheat leaves were harvested at 0 h, 24 h, and 48 h after salt treatment.

### 2.7. RNA Extraction and qRT-PCR

Total RNA was extracted from wheat leaves under drought and salt treatments using RNAprep Pure Plant Kit (Tiangen, Beijing, China). The RNA was reverse-transcribed into cDNA and used to perform the quantitative RT-PCR analysis. The expression levels of related genes were measured by qRT-PCR using the Roche LightCycler^®^ 480 system (Roche, Mannheim, Germany). The 18S rRNA was used as an endogenous control in wheat. The gene relative expression was calculated using the 2^−ΔΔCT^ method. All qRT-PCR analyses were performed with three independent biological replicates, and each biological replicate included three technical replicates. Statistical significance between the treatment and control groups was determined using a two-tailed Student *t*-test, with the significance threshold set at *p* < 0.05. Error bars in the corresponding figures represent the standard deviation (SD) of the biological replicates. All primers in this study are listed in Table S2.

## 3. Results

### 3.1. Genome-Wide Identification and Classification of the ASMT Gene Family in Wheat and Other Plant Species

We identified *ASMT* genes with the O-methyltransferase domain in 15 representative plants (see Section 2) (Table 1 and Table S3). Among them, we identified *ASMT* genes with typical O-methyltransferase domains in five wheat species (*T. aestivum* 156, *T. spelta* 159, *T. dicoccoides* 77, *T. turgidum* 58, and *T. urartu* 37) (Table S3). Sequences with atypical O-methyltransferase domains (Pfam domain models (PF00891) with less than 50% coverage) were excluded in *T. aestivum* (12), *T. urartu* (4), *T. dicoccoides* (12), *T. turgidum* (4), and *T. spelta* (7), respectively (Table S4). Atypical *ASMT*s of the other 10 plants were also excluded (Table S4). Overall, these rigorous filtering procedures (detailed in Tables S3 and S4) successfully removed truncated sequences and potential pseudogenes across all 15 species, establishing a highly reliable dataset of typical *ASMT*s for subsequent evolutionary and functional analyses.

**Figure 1:**
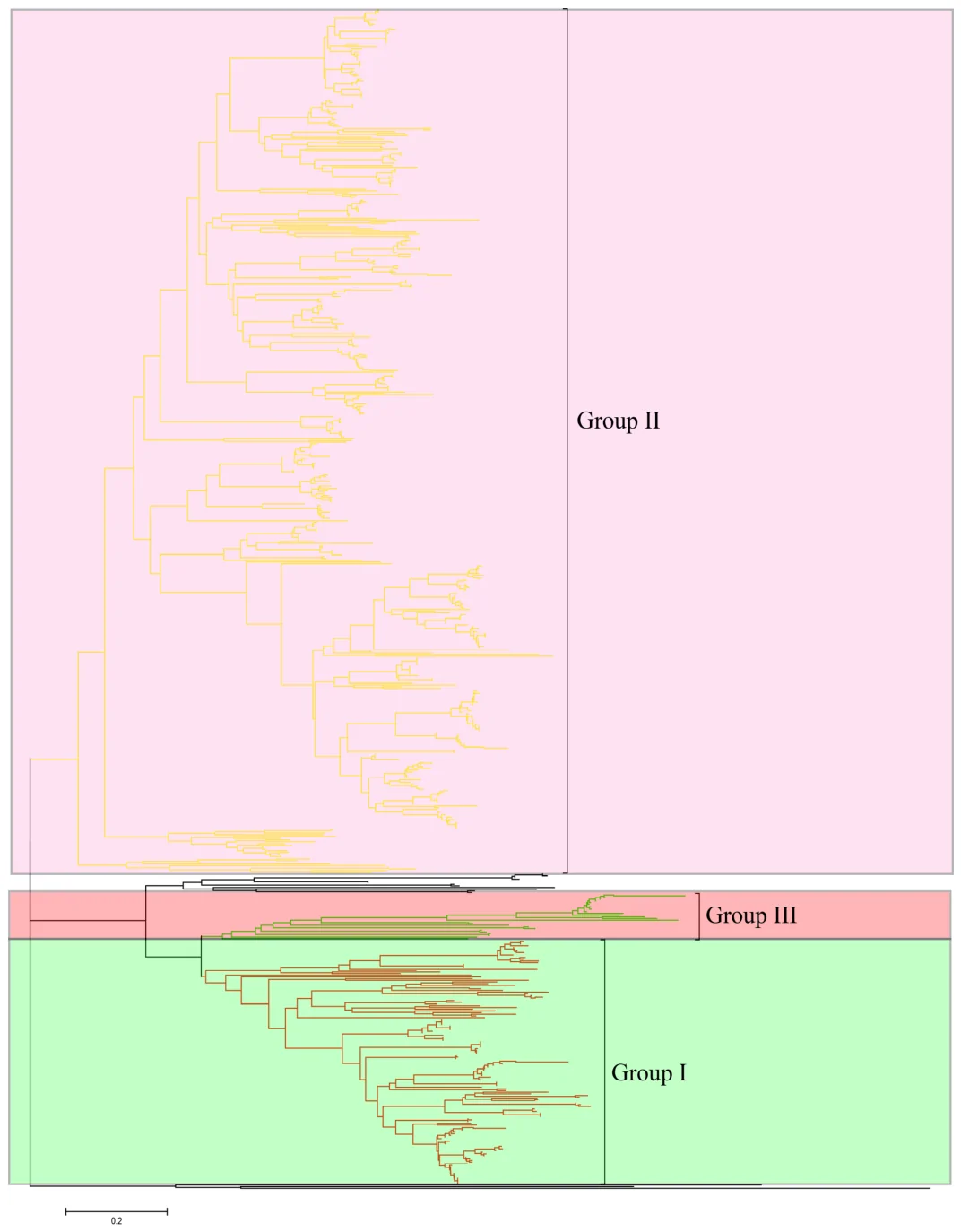
Phylogenetic tree and classification of ASMT proteins in wheat and 14 other plant species. The initial Neighbour-Joining (NJ) phylogenetic tree was constructed using MEGA 11.0 based on the amino acid sequences of the ASMT domain, utilizing the JTT model with 1000 bootstrap replicates. The resulting tree was subsequently analyzed and edited for visualization using MEGA-CC 7.0 software. The ASMT members are clustered into three principal subfamilies (Subfamilies I, II, and III), which are designated with distinct background colors. Comprehensive tree information is available in Supplementary Materials Figure S1.

The classification of *ASMT*s in 15 plant species was based on four kinds of phylogenetic trees (including NJ tree with p-distance model, NJ tree with JTT model, maximum likelihood (ML) tree and Bayesian tree) (Figure 1 and Figure S1). The classification criteria of *ASMT* subfamilies were adopted from a previous study in *Malus domestica* [13] (Table S5). We classified *ASMT*s into three subfamilies (I, II and III) primarily based on the NJ (JTT) tree, and the classifications derived from four independent phylogenetic trees were highly consistent (Table S6). The topology of the phylogenetic tree revealed distinct evolutionary patterns between monocotyledonous and dicotyledonous plants. We observed several clades that were lineage-specific, containing members exclusively from either monocots or dicots. However, other branches comprised a mix of sequences from both groups. Phylogenetic analysis revealed that the *P. patens* sequence (*Pp3c11_12200V3.1*) consistently resolved at the base of subfamily III, suggesting that it represents the most ancestral lineage. In contrast, the sequence from the green alga *C. reinhardtii* failed to resolve into any major subfamily and thus remained unclassified. This lack of clear classification is likely because its gene architecture, particularly its exon–intron structure, predates the establishment of the conserved, ancestral *ASMT* structure observed in land plants.

### 3.2. Evolution and Conserved Exon–Intron Structure of the ASMTs

**Figure 2:**
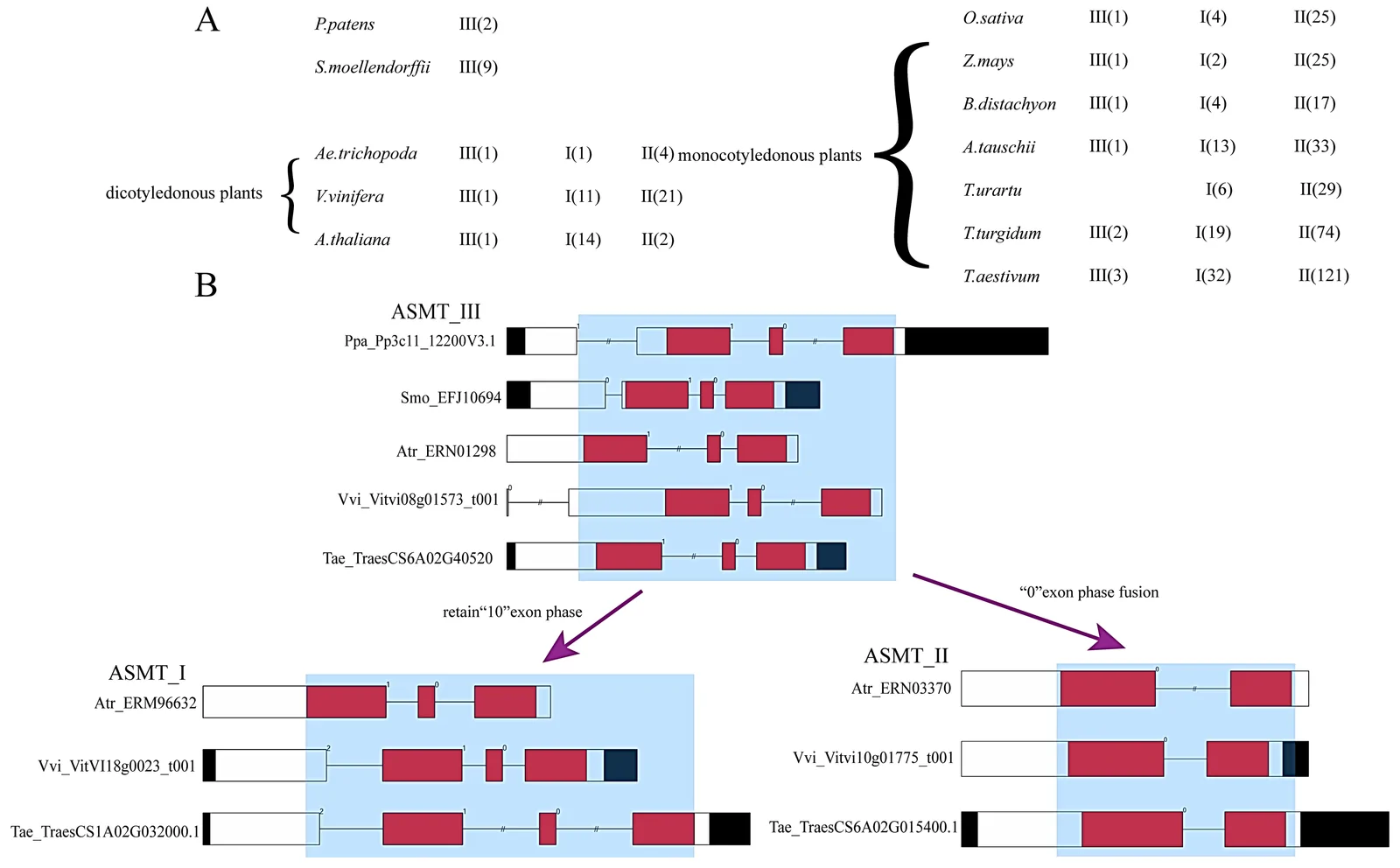
Phylogenetic relationships and conserved exon–intron structures of the *ASMT* gene family. (**A**) Evolutionary history of the *ASMT* subfamilies, showing the expansion patterns across lineages. (**B**) Conserved exon–intron structural organization. Red boxes denote the core O-methyltransferase (ASMT) domains, white boxes indicate other exon regions, black boxes represent untranslated regions (UTRs), and lines indicate introns. The symbol “//” indicates extended introns. Explanation of Exon Phases: The numbers 0, 1, and 2 represent the intron phases. Phase 0 indicates an intron located between two codons. Phase 1 indicates an intron located after the first nucleotide of a codon. Phase 2 indicates an intron located after the second nucleotide. The shared “1-0” intron phase pattern (representing two consecutive introns with splice-phases 1 and 0 at conserved positions) observed in the ancestral Subfamily III and Subfamily I suggests a conserved evolutionary lineage. In contrast, the emergence of the “0” phase in Subfamily II indicates a distinct evolutionary divergence event characterized by exon fusion and subsequent massive expansion in monocots.

In order to obtain the evolutionary information of *ASMT*s, we diagrammed the exon–intron structures within the O-methyltransferase domain (red areas) in 15 plants (Figure S2). The results showed that some members from the same *ASMT* subfamilies had conserved exon–intron structures from *S. moellendorffii* to *Triticum aestivum*. Based on the above results, we summarized an evolutionary model of *ASMT*s involving a highly conserved “1-0” intron phase pattern (Figure 2). Analysis of the *ASMT* gene family across the plant kingdom revealed a history of significant expansion and structural diversification (Figure 2A). The members of *ASMT*s were minimal in basal land plants such as *P. patens* and *S. moellendorffii*, and expanded moderately in dicotyledons. However, *ASMT*s underwent a massive expansion in monocots (such as 30 in *O. sativa* and 156 in *T. aestivum*). Based on the phylogenetic distribution and distinct exon–intron structures, we propose an evolutionary model. *ASMT* subfamily III represented the ancestral lineage. This conclusion was supported by two key observations: (1) The basal plants *P. patens* and *S. moellendorffii* only contained subfamily III *ASMT*s. (2) Furthermore, all *ASMT* genes in these ancient lineages share a single, conserved structural pattern, which is characterized by the insertion of two successive introns with phases 1 and 0 (referred to as the ‘1-0’ pattern) at conserved positions within the core domain (Figure 2B). Our phylogenetic analysis across 15 plant species reveals a clear chronological emergence of the three *ASMT* subfamilies. Subfamily III represents the most ancient lineage, as its members are the only ones found in the basal land plants *P. patens* and *S. moellendorffii*. In contrast, Subfamilies I and II are absent in these foundational lineages and appear to have emerged later in plant evolution, as they are first observed in both dicotyledonous and monocotyledonous plants. This distribution pattern suggests a hypothetical evolutionary timeline where the basal Subfamily III potentially gave rise to the more recently derived Subfamilies I and II. Following their emergence, the three subfamilies experienced markedly different patterns of numerical expansion across plant lineages (Figure 2A).

Subfamily III, after its appearance, has remained a small, low-copy-number subfamily, typically containing only one to three members in any given species. Subfamily II, contrary to being a static, single-copy ancestral group, underwent a massive expansion, particularly within the monocot lineage, increasing from just two members in *A. thaliana* to 121 members in *T. aestivum*. Subfamily I exhibits an intermediate pattern of moderate, lineage-specific expansion, especially within the wheat lineage, reaching 32 members in *T. aestivum*. These distinct expansion trajectories suggest that the three subfamilies have been subjected to different evolutionary pressures following their divergence. The divergence of Subfamilies I and II from their common ancestor, Subfamily III, is underpinned by specific and distinct changes in their gene architecture (Figure 2B). Subfamily I was formed through a relatively simple divergence event, as it retained the ancestral ‘1-0’ intron phase pattern characteristic of Subfamily III. In contrast, the formation of Subfamily II involved a more complex structural change: we postulate a hypothetical exon fusion event that might have merged two ancestral exons, thereby leading to its novel and distinct ‘0’ intron phase.

To study domain distribution, we batch-scanned all *ASMT* genes from the 14 studied plants against the Pfam37 database and plotted the results using Perl and R scripts (Figure S4). The domain distribution analysis highlighted a remarkable degree of structural conservation within the *ASMT* gene family. Across all 14 species, the vast majority of ASMT proteins conformed to a canonical single-domain architecture. Unlike many other large gene families that diversify through domain shuffling and the creation of multi-domain proteins, the *ASMT* family appears to have been maintained under strong purifying selection that preserves its core structural integrity.

### 3.3. Chromosomal Location and Tandem Duplication Events of ASMT in T. aestivum

In order to elucidate the genomic distribution of the wheat *ASMT* gene family, 148 of the 156 identified *TaeASMT* members were mapped on the 21 chromosomes of *Triticum aestivum* (Figure S5; Table S7). The *TaeASMT* genes were unevenly distributed across the wheat genome, with a significantly greater number of genes on the homoeologous chromosome group 1 (1A, 1B, and 1D). The hexaploid bread wheat (*T. aestivum*) genome consists of three subgenomes (A, B, and D), which were involved in three polyploidisation events [42]. This non-random distribution suggests that gene duplication has played a key role in the family’s expansion. To investigate a specific mechanism of localized proliferation, we analyzed tandem duplication events. A total of 17 tandem duplication clusters were identified in the *T. aestivum* genome, widely distributed across 16 different chromosomes within the A, B, and D subgenomes (Table S8; Figure S6). These results indicated that tandem duplication might be a key factor driving the expansion of wheat *ASMT*s.

To investigate the impact of large-scale genomic events, we analyzed segmental duplications caused by polyploidization. Collinearity analysis identified numerous distinct collinear gene pairs widely distributed across the wheat genome. As visualized in the synteny map, these extensive segmental duplications predominantly formed conserved networks linking the homoeologous chromosomes across the A, B, and D subgenomes (such as the distinct triplicated loci observed in chromosome groups 1, 3, and 6), highlighting the widespread retention of these genes following allopolyploidization (Table S9; Figure S7). To estimate the timeline of these duplications, we calculated the synonymous substitution rates (Ks) of these gene pairs. The distribution of Ks values exhibited a prominent peak with a center between 0.20 and 0.35 (Figure S8). This peak’s timing might be tightly coupled with the recent polyploidization events that formed wheat. This result strongly suggests that polyploidization and subsequent segmental duplications were the primary driving factors for the extensive expansion of the *TaeASMT* gene family. These extensive tandem and segmental duplication events have significant functional implications for the *TaeASMT* family. Consistent with findings in the *ASMT* family of *Juglans regia*, this expansion likely provides functional redundancy, contributing to the evolutionary robustness of the gene family [17]. Furthermore, our expression analysis reveals that duplicated paralogs often exhibit distinct transcriptional patterns, with some showing strong stress-inducibility while others maintain basal levels. This divergence suggests potential functional subfunctionalization, hypothetically allowing the expanded *TaeASMT* family to perform both acute stress response and constitutive regulatory roles upon further validation.

**Figure 3:**
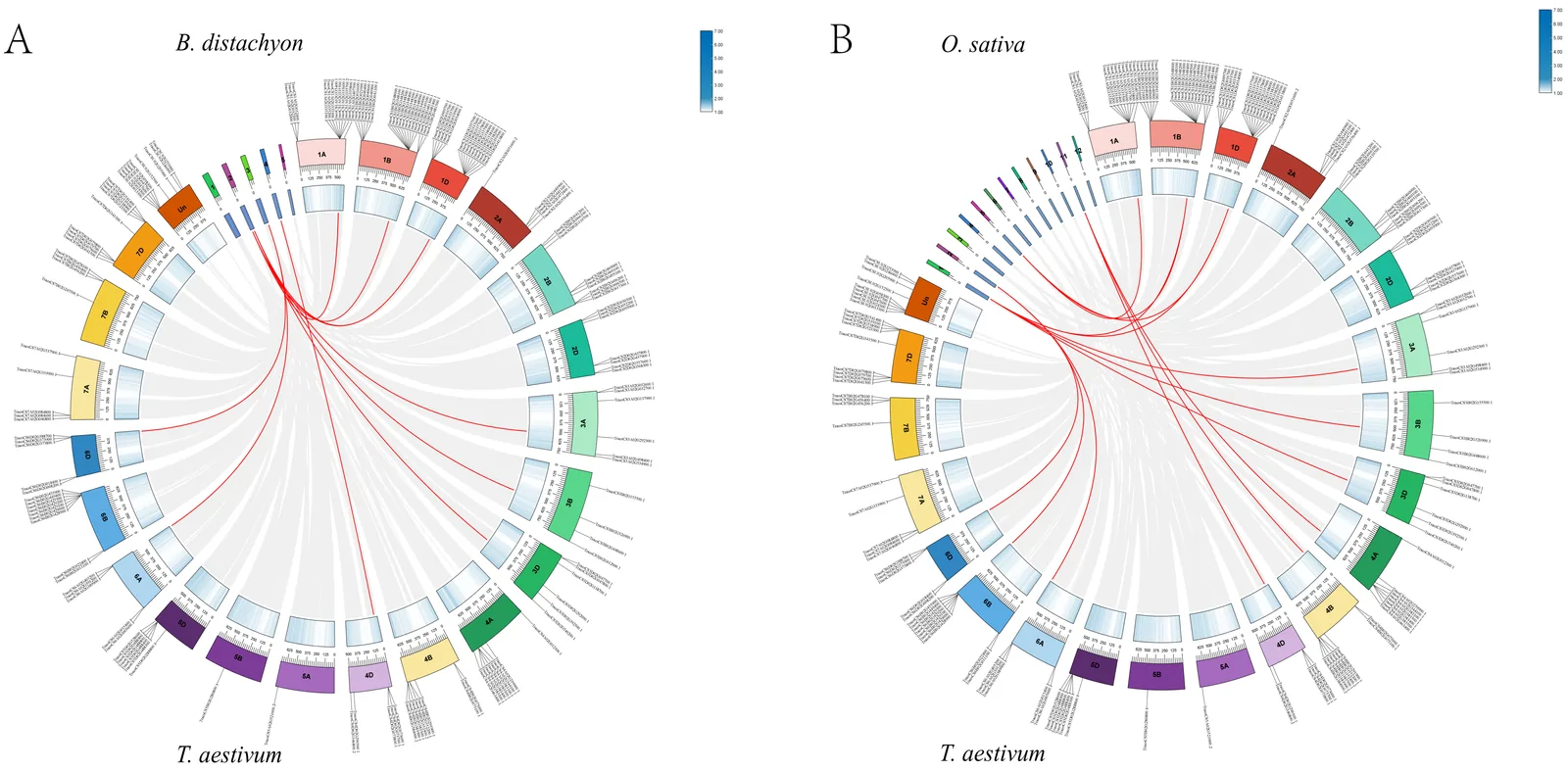
Collinearity analysis of the *ASMT* gene. This figure shows the synteny maps of *T. aestivum* and the two related grass species (*B. distachyon* and *O. sativa*). Syntenic gene pairings between *ASMT*s are shown by red curves, whilst other genes are represented by grey curves. (**A**) Synteny of *ASMT*s in *T. aestivum* and *B. distachyon.* (**B**) Synteny of *ASMT*s in *T. aestivum* and *O. sativa*.

To trace the deeper evolutionary history of the *TaeASMT* family, comparative synteny maps were constructed between *T. aestivum* and two other key grass species (*Oryza sativa* and *Brachypodium distachyon*) (Figure 3). The results indicate that there are 15 syntenic gene pairs between *T. aestivum* and *B. distachyon*, and another 15 pairs between *T. aestivum* and *O. sativa* (Table S10). Notably, several *TaeASMT* genes have formed a direct homologous relationship with members of *T. aestivum* and *B. distachyon*. For example, *TraesCS3A02G292300* shares a syntenic relationship with both *BRADI_2g50111v3* (*B. distachyon*) and *BGIOSGA004473* (*O. sativa*). This conserved synteny among these three species indicated that these orthologous genes originated from a common ancestor before the divergence of the Poaceae lineages.

### 3.4. Expression Pattern of T. aestivum ASMT Under Abiotic Stress

To investigate how the *T. aestivum ASMT* gene family responds to various abiotic stresses, we performed a differential expression analysis on several public RNA-seq datasets. Following a standard bioinformatic pipeline (see Section 2), genes with a |log_2_(FoldChange)| > 1 were identified as potential stress-responsive candidates for further experimental validation (Table S11A).

(1) Drought: The expression profiles of *TaeASMT*s under drought stress were examined using two transcriptome datasets from three wheat cultivars, ‘Zhengmai1860,’ ‘Zhoumai18,’ and ‘Bainong207’ (BioProject IDs: 1077667 and 994277). Our results showed that most *TaeASMT*s exhibited mild upregulation or downregulation under drought treatment. However, we noticed that a few genes such as *II_TraesCS3A02G137900* showed strong upregulation. Some genes, such as *II_TraesCS6D02G373800* and *II_TraesCS2B02G041200*, only exhibited upregulation under melatonin treatment. Interestingly, some genes, such as *II_TraesCS2B02G110700* and *II_TraesCS2D02G093900*, exhibited downregulations under drought treatment, but upregulations under normal treatment. Therefore, these genes might be key responsive candidates in wheat drought stress response. (2) Heat stress: Our results showed that most *TaeASMT*s exhibited mild changes in expression under heat stress. However, we noticed that a few genes, such as *II_TraesCS4A02G239900* and *II_TraesCS7A02G084800*, showed strong upregulation. Interestingly, the expression levels of genes like *II_TraesCS4D02G073600* and *II_TraesCS6D02G018400* decreased under high-temperature treatment but increased under normal treatment. Therefore, these genes might be highly responsive to heat stress. (3) Salt stress: Our results showed that most *TaeASMT*s exhibited mild expression changes under salt stress conditions. However, we noticed that *II_TraesCS2B02G606200* and *II_TraesCS2B02G606500* exhibited strong upregulation. Therefore, these genes may serve as salt-responsive candidates. (4) Flooding: Our results showed that the expression patterns for most *TaeASMT*s were generally mild under flooding conditions. However, we noticed that *I_TraesCS5D02G488800* and *I_TraesCS1D02G359500* showed strong upregulation. Therefore, these genes might exhibit specific responsiveness to the response to anaerobic stress caused by flooding.

**Figure 4:**
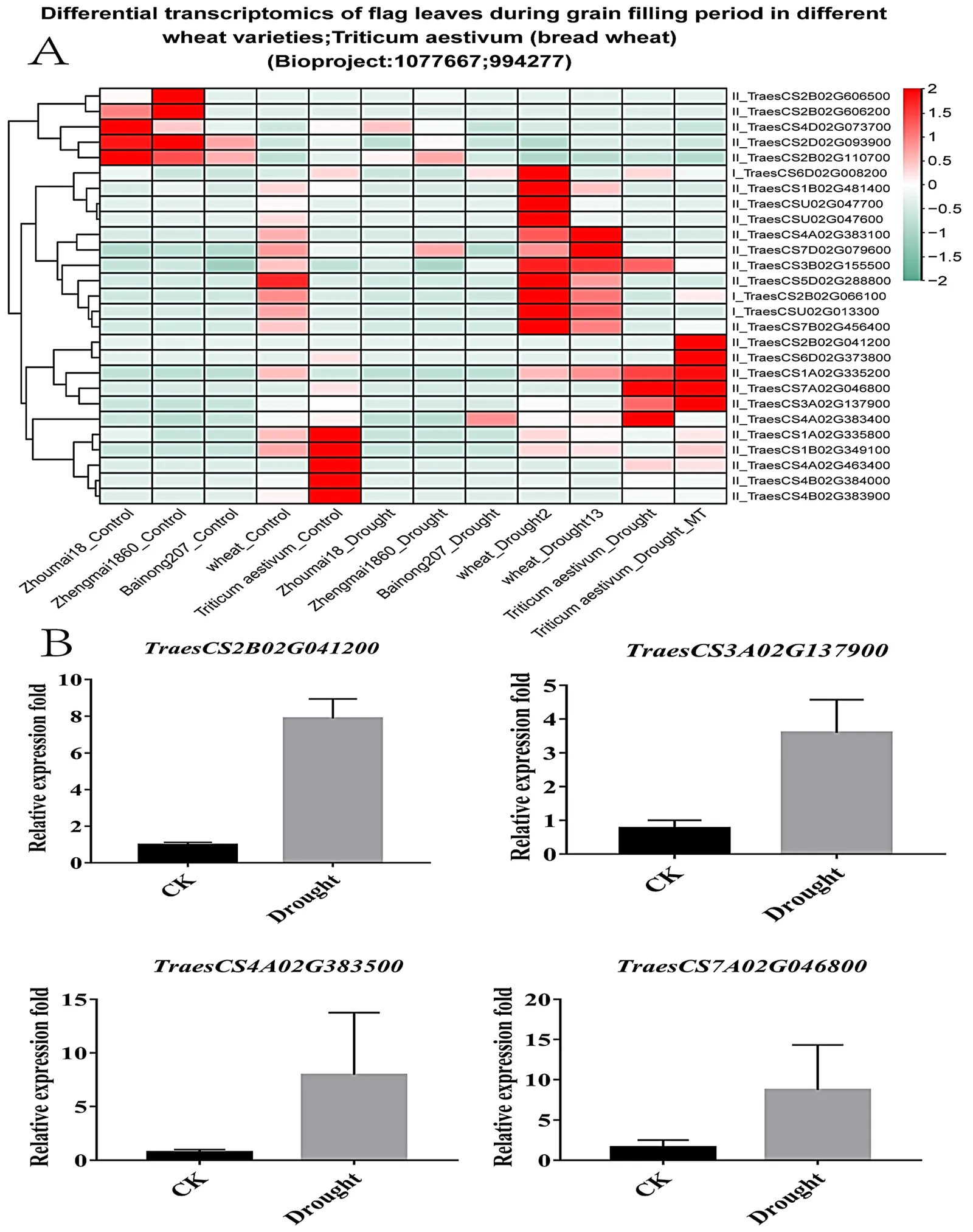
Heat map of wheat *ASMT* and its qRT-PCR results under drought conditions. (**A**) Transcriptome heat map. The color bar represents the relative expression value, with greenish color indicating little or no expression and red indicating high expression. (**B**) qRT-PCR results under drought treatment. Error bars represent the standard deviation (SD) of three independent biological replicates. Statistical significance was evaluated using a Student *t*-test (*p* < 0.05).

To provide independent experimental confirmation of the *TaeASMT* genes in the drought stress response across different cultivars, we selected four candidate genes (*II_TraesCS2B02G041200*, *II_TraesCS3A02G137900*, *II_TraesCS4A02G383500* and *II_TraesCS7A02G046800*) by using qRT-PCR analysis. These specific candidates were selected because they exhibited the strongest upregulation (the highest Log2FC values) in the transcriptomic datasets. The expression trends of qRT-PCR were highly consistent with public transcriptome data, confirming the patterns observed in the RNA-seq analysis (Figure 4A,B). In particular, all four tested genes of transcriptome exhibited significant upregulation under drought treatment, with log_2_(Fold Change) values of 1.48, 1.87, 2.08, and 2.21, respectively. These results independently confirmed the drought responsiveness of these wheat *ASMT* genes.

**Figure 5:**
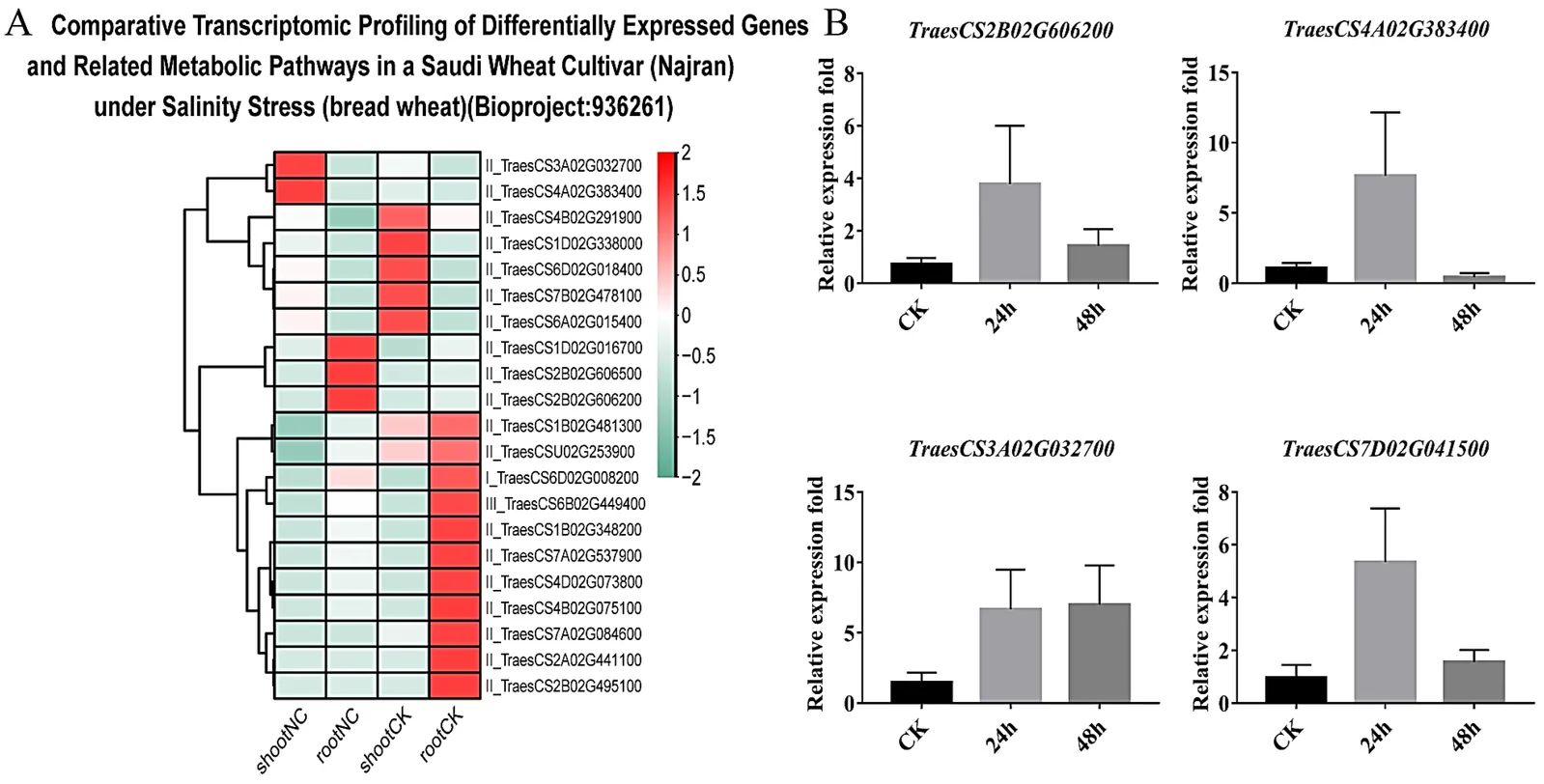
Heatmap of wheat *ASMT* under salt stress treatment and its qRT-PCR results. (**A**) Transcriptome heatmap. The color bar represents the relative expression value, with greenish color indicating little or no expression and red indicating high expression. (**B**) qRT-PCR results under salt stress treatment. Error bars represent the standard deviation (SD) of three independent biological replicates. Statistical significance was evaluated using a Student *t*-test (*p* < 0.05).

To provide independent experimental confirmation of the *TaeASMT* genes in the salt stress response, we selected another four candidate genes (*II_TraesCS2B02G606200*, *II_TraesCS4A02G383400*, *II_TraesCS3A02G032700*, and *II_TraesCS7D02G041500*) and analyzed their expression at 24 and 48 h post-treatment by using qRT-PCR analysis. The expression trends of qRT-PCR were highly consistent with the corresponding public transcriptome data (BioProject: 936261), confirming the patterns observed in the RNA-seq analysis (Figure 5A,B). In particular, all four tested genes exhibited significant upregulation under salt treatment, with log_2_(Fold Change) values of 3.74, 2.96, 1.88, and 1.67, respectively. These results independently confirmed the salt responsiveness of these wheat *ASMT* genes.

### 3.5. Expression Patterns of ASMTs in T. aestivum Under Biotic Stress

To investigate the transcriptional responses of the *T. aestivum ASMT* gene family to various biotic stresses, we analyzed public RNA-seq datasets from the NCBI SRA database (Figure S9, Table S11B). The analytical pipeline involved quality control with FastQC, alignment to the reference genome using STAR, and expression quantification via RSEM. For exploratory trend analysis, differentially expressed gene (DEG) candidates were initially screened using the DESeq2 R package, with a primary fold-change threshold of |log_2_(FoldChange)| > 1. (1) *Fusarium graminearum*: To investigate the response to Fusarium graminearum, the causative agent of Fusarium head blight, we analyzed a transcriptome dataset comparing the wheat varieties ‘Wuyangmai’ and ‘Chinese Spring’ (BioProject ID: 1183537). While most *TaeASMT*s showed only mild changes, we identified several variety-specific responses. Notably, *I_TraesCS3B02G448600* and *II_TraesCS1B02G481400* were strongly upregulated specifically in the ‘Chinese Spring’ variety. Conversely, the expression of genes like *II_TraesCS1B02G348000* decreased post-infection. Furthermore, *II_TraesCS1B02G481500* and *II_TraesCS1B02G481300* were upregulated in both wheat varieties under infection, suggesting these genes are potentially involved in the general response to Fusarium head blight. (2) *Puccinia striiformis* f. sp. *Tritici*: The response to stripe rust infection was assessed using a public RNA-seq dataset (BioProject ID: 1006331). Our analysis of this dataset showed that although most *TaeASMT*s were not significantly affected by the infection, *II_TraesCS5D02G488300* and *II_TraesCS5D02G488400* exhibited strong upregulation, indicating they are strong pathogen-responsive candidate genes. (3) Powdery mildew: For powdery mildew, we analyzed a dataset comparing susceptible and resistant wheat lines from Shandong (BioProject ID: 914331). Most *TaeASMT*s showed only minor expression changes in both lines. However, a key finding was the strong upregulation of two Subfamily I genes, *I_TraesCS2D02G568300* and *I_TraesCS5D02G488800*, exclusively in the powdery mildew-resistant wheat variety. This suggests that they are candidate genes responsive to powdery mildew infection. (4) *Puccinia triticina TBDG*: Finally, the expression patterns under leaf rust infection were investigated using two public datasets (BioProject IDs: 972750 and 782527). Consistent with the findings for stripe rust, most genes were only slightly affected. However, *II_TraesCS5D02G488300* and *II_TraesCS5D02G488400* once again showed strong upregulation, suggesting these two genes are candidate components of a broader transcriptional response against different rust pathogens.

### 3.6. Analysis of Cis-Acting Elements

**Figure 6:**
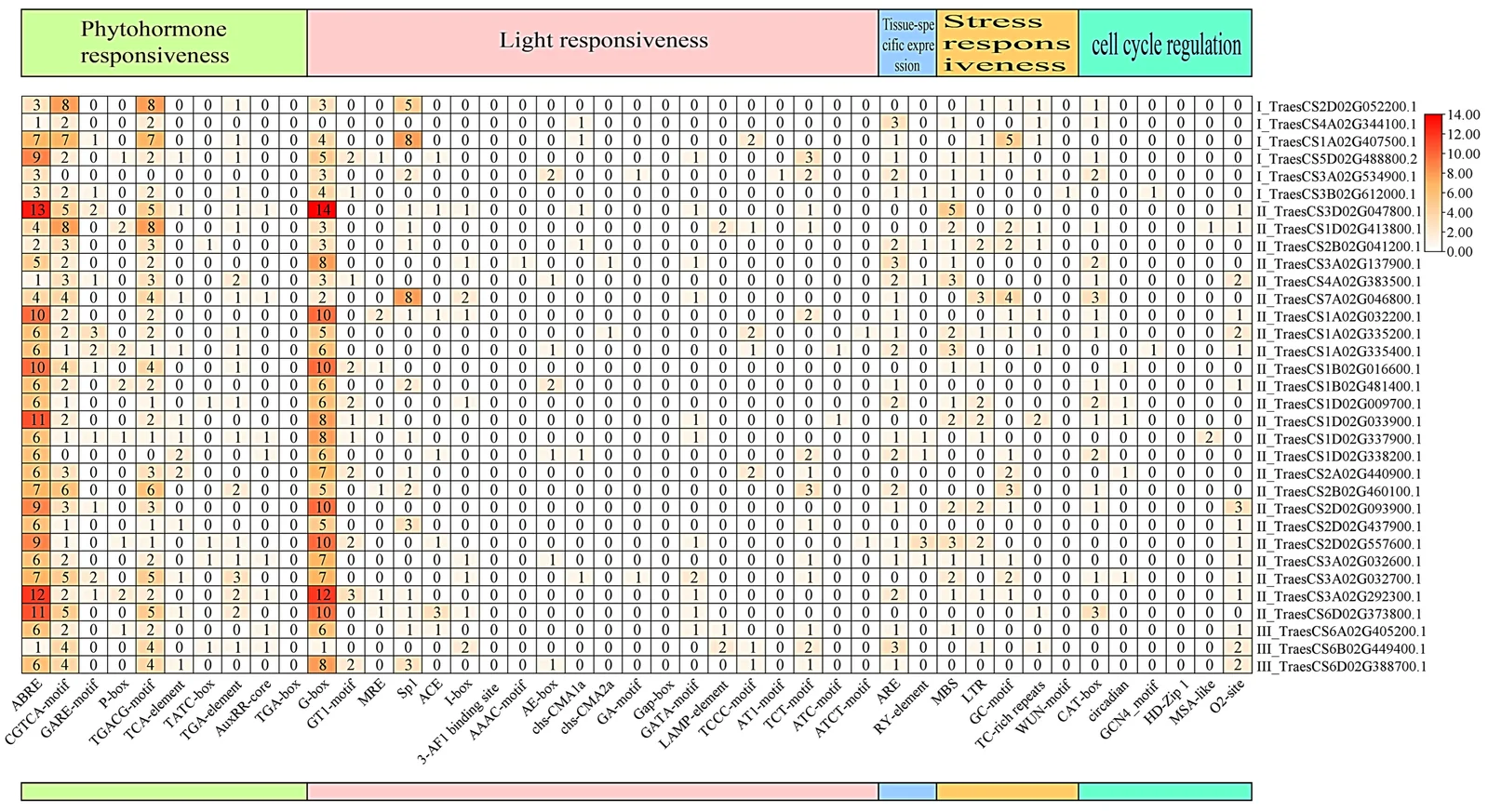
Examination of cis-acting elements of the *ASMT* gene in *T. aestivum*. The horizontal axis denotes various biological processes categorized by distinct cis-acting elements, while the vertical axis indicates gene names; the figures show the quantity of cis-acting elements.

To elucidate the potential regulatory mechanisms governing the wheat *ASMT* gene family, we analyzed the cis-acting regulatory elements (CREs) within their promoter regions. CREs are short DNA sequences that serve as binding sites for transcription factors (TFs), thereby modulating gene expression in response to various developmental and environmental cues. Using the PlantCARE (http://bioinformatics.psb.ugent.be/webtools/plantcare/html/ (accessed on 16 August 2026)), we identified and characterized the CREs in the promoter regions of 156 wheat *ASMT* genes. The identified elements were classified into five major functional categories: hormone-responsive (e.g., ABRE, TGACG-motif), light-responsive (e.g., G-box, MRE), stress-responsive (e.g., LTR, MBS), elements related to cell cycle regulation (e.g., CAT-box, O2-site), and those involved in tissue-specific expression (e.g., ARE, RY-element) (Figure 6). A key finding of our results was the universal presence of hormone- and stress-responsive elements in all analyzed *ASMT* promoters, indicating their potential involvement in these signal pathways. Among all identified CREs, the abscisic acid-responsive element (ABRE) and the light-responsive G-box were the most abundant. In addition, most *ASMT* genes contained at least one LTR (low-temperature responsive), MBS (MYB binding site is involved in drought), or GC-motif element. Overall, the composition and prevalence of these CREs strongly suggest that the wheat *ASMT* gene family is intricately involved in mediating responses to diverse abiotic stresses and is subject to complex regulation by plant hormones.

## 4. Discussion

### 4.1. Expansion, Polyploidization, and Structural Evolution of the Wheat ASMT Gene Family

In recent years, the publication of the wheat genome has enabled numerous gene family investigations; however, this study represents the first comprehensive, genome-wide analysis of the *ASMT* gene family in wheat. ASMT, as an indispensable final catalyst in the synthesis of the antioxidant melatonin, is crucial for plant stress response processes. While the *ASMT* gene family has been characterized in several species, including *Arabidopsis thaliana* (19 members) and *Malus domestica* (37 members), our identification of 156 *TaeASMT* members reveals a remarkable expansion in wheat. According to our analysis, this significant expansion of *TaeASMT*s was related to the two major allopolyploidization events of wheat (*T. aestivum*). The first allopolyploidization occurred approximately 800,000 years ago, leading to tetraploid wheat [43]. The second allopolyploidization occurred around 8500–9000 years ago, resulting in the emergence of wheat through hybridization with *Ae. tauschii* (D subgenome donor) [43]. Our synteny analysis of *TaeASMT*s supported this theory of wheat polyploidization. The homologous *TaeASMT*s were widely distributed across the A, B, and D subgenomes. Furthermore, the Ks values of duplicated gene pairs showed a distinct peak between 0 and 0.35. These results combined indicated that whole-genome duplication (WGD) and segmental duplications were the main driving forces for the expansion of the *TaeASMT* gene family. Our result is consistent with findings in the wheat expansin gene family, where polyploidy was also identified as the principal evolutionary force for family expansion [44]. To further support the direct link between gene expansion and functional diversification, we evaluated the expression divergence of duplicated *TaeASMT* genes. Following gene duplication, paralogs often undergo subfunctionalization or neofunctionalization to avoid genetic redundancy. Our transcriptomic profiling provides observational support for this hypothesis. For instance, within the identified duplicated pairs, *II_TraesCS3B02G155500* was strongly induced under heat stress, whereas its duplicated paralog, *II_TraesCS3D02G138700*, exhibited negligible or entirely divergent expression patterns under the exact same conditions. Such distinct spatial–temporal and stress-responsive expression partitioning among duplicated gene pairs suggests potential subfunctionalization. This suggests that the massive expansion driven by polyploidization and segmental duplication did not merely increase the gene copy number, but likely contributed to the adaptive functional diversification of the *TaeASMT* family in wheat.

In addition, our analysis of gene structures provided an evolutionary model of *TaeASMT*s (Figure 2). Our model indicates that subfamily III might be the ancestral lineage of the *TaeASMT* gene family. This is because moss (*P. patens*) and lycophyte (*S. moellendorffii*) contained only subfamily III ASMTs, but not subfamilies I and II. Additionally, almost all members of Subfamily III *ASMTs* contained a conserved gene structure characterized by the ‘1-0’ intron phase pattern from *P. patens* to *T. aestivum*, suggesting that this specific splicing architecture has potential functional significance in plant growth and development. The principle that gene structure is critical for function and evolution is well-established [45]. Furthermore, our observation of conserved architecture within phylogenetic clades is consistent with findings in both other large gene families, such as the MYBs in oil palm (*Elaeis guineensis* Jacq.) [46], and specifically within the *ASMT* family in apple [13]. Subfamily III represents the ancestral form of the gene family, as exemplified by the gene *Ppa_Pp3c11_12200V3.1* from the basal land plant *P. patens*. This ancestral gene is characterized by its conserved ‘1-0’ intron phase pattern, which serves as the structural blueprint for subsequent diversification. During subsequent evolution, two distinct divergence events occurred. The formation of Subfamily I was a conservative event; its members, such as *Tae_TraesCS1A02G032000.1*, retained the ancestral ‘1-0’ intron phase pattern but likely underwent a terminal exon loss, resulting in a shorter gene architecture. In contrast, the emergence of Subfamily II, which is absent in basal plants, involved a more significant structural innovation. This inferential model is strongly supported by comparative genomic evidence across the 15 analyzed species. The ‘1-0’ pattern and the novel ‘0’ intron phase exhibited strict topological segregation onto distinct phylogenetic clades. Coupled with the chronological appearance of the ‘0’ phase strictly in evolutionarily younger angiosperms, this pattern provides parsimonious evidence for a derived structural shift. This lineage was likely formed through an exon fusion event (i.e., the loss of an internal intron, a well-documented mechanism driving functional divergence in duplicated plant genes), a change that is clearly visible in representative members like *Tae_TraesCS6A02G015400.1* [45]. This fusion of two ancestral exons created the novel and characteristic ‘0’ intron phase that defines this subfamily. These specific molecular events—retention with exon loss versus exon fusion—provided the structural blueprints for the new subfamilies and likely preceded the large-scale numerical expansion observed predominantly in the grass family. The comprehensive nature of our study, which included diploid, tetraploid, and wheat species, provides a comparative genomic basis for this hypothetical model and offers a framework for exploring the genesis and structural evolution of this vital gene family. However, it is crucial to acknowledge the limitations of this structural evolutionary model. Given our restricted taxonomic sampling of 15 representative species and the in silico nature of the annotations, our proposed “1-0” to “0” transition and the postulated exon-fusion event remain largely hypothetical. Alternative evolutionary scenarios, such as multiple independent intron loss events across different lineages or convergent evolution of the gene architecture, cannot be completely ruled out. Future studies incorporating a much broader and denser phylogenetic sampling across diverse plant lineages, coupled with robust experimental functional validation of these ancient sequences, are required to definitively test these alternative evolutionary hypotheses.

### 4.2. Functional Significance of the TaeASMT Genes in Stress Responses

Our integrated analysis of transcriptome data and experimental validation provides significant insights into the functional roles of the expanded *TaeASMT* family, particularly in stress responses. The diverse expression profiles of *TaeASMT*s under various abiotic stresses, including drought, heat, salt, and flooding, suggest a high degree of functional divergence (Figure S9, Table S11A). Under drought, the significant upregulation of *II_TraesCS3A02G137900* is consistent with studies in apple where the *MD01G1229100* gene was also induced by abiotic stress [13]. Furthermore, the upregulation of *II_TraesCS6D02G373800* and *II_TraesCS2B02G041200* specifically in melatonin-treated wheat aligns with research in poplar, where *Potri 009G139800* overexpression enhanced drought resistance by increasing endogenous melatonin levels [16,47,48]. Mechanistically, this enhanced resistance is attributed to the catalytic role of ASMT in promoting endogenous melatonin accumulation, which functions as a potent antioxidant to scavenge reactive oxygen species (ROS) and alleviate oxidative damage under osmotic stress. To independently confirm our transcriptomic observations, we performed qRT-PCR on four key drought-responsive candidate genes. The results were highly consistent with the RNA-seq data, confirming the significant upregulation of all four selected genes and corroborating their identity as drought-responsive candidate genes in wheat (Figure 4). This two-step approach of transcriptome mining followed by independent qRT-PCR confirmation is a standard and effective strategy for identifying functional candidate genes, a methodology also recently employed in the characterization of the *ASMT* gene family in tea plants (*Camellia sinensis*) [10,12]. Similarly, qRT-PCR independently confirmed the strong induction of four key salt-responsive genes, such as *II_TraesCS2B02G606200* (log_2_FC = 3.74), corroborating their strong transcriptional responsiveness to salinity (Figure 5), a function also reported for *GSVIVT01020642001* from grape [49]. While specific *TaeASMT* genes exhibited strong induction, a significant proportion of the family showed only mild transcriptional fluctuations under stress. This pattern likely reflects a complex regulatory strategy involving both functional divergence and post-transcriptional control. Rather than indicating a minor role, these “mildly” responsive genes may serve as “fine-tuners” responsible for maintaining basal melatonin homeostasis, complementing the high-amplitude response of primary stress-inducible paralogs. Furthermore, recent studies in walnut suggest that ASMT transcripts are heavily targeted by small RNAs (sRNAs), such as the stress-responsive miR399, which suppress mRNA levels to prevent ectopic accumulation [17]. Therefore, the observed mild regulation in our transcriptome data may represent a tightly controlled basal state maintained by sRNA-mediated surveillance, ensuring that the plant retains the potential for rapid, “subtle” modulation of stress resistance without constitutive high expression.

In addition to abiotic stress, melatonin accumulation is also a critical defense mechanism against pathogens. For instance, *ASMT* overexpression has been shown to enhance fungal resistance in poplar and *Arabidopsis* [16,50]. In our study, the significant upregulation of *II_TraesCS2D02G437800* and *II_TraesCS1B02G481400* following *Fusarium graminearum* infection suggests that these genes are potentially involved in wheat’s defense against this devastating pathogen.

The molecular basis for the diverse expression patterns observed in this study can be partially explained by the specific cis-acting regulatory elements (CREs) identified in the promoter regions. Similar to recent findings in potato and barley where promoter analysis revealed key hormone-responsive elements linking gene families to stress resilience [51,52], our direct observations reveal a correlation between the transcriptional upregulation of key candidate genes and the enrichment of specific stress-responsive elements. For instance, the strong stress-induced expression of *II_TraesCS3A02G032700* aligns with an enrichment of ABA-responsive elements (ABREs) in its promoter. Extrapolating from findings in perennial ryegrass and tomato [53,54], we hypothesize a regulatory feedback loop: stress-induced ABA activates ABA-responsive transcription factors (e.g., AREB/ABFs) that bind to these ABREs, upregulating *ASMT* expression for melatonin biosynthesis. The resulting melatonin may subsequently fine-tune ABA levels to prevent excessive senescence. While this study provides a comprehensive genome-wide characterization of the wheat *ASMT* family, several limitations must be explicitly acknowledged. First, the bioinformatic identification is based on in silico sequence homology and conserved domains; definitive confirmation of *ASMT* identity requires future biochemical validation of actual enzymatic activity. Second, while the public transcriptomic datasets offer valuable broad-scale exploratory insights, their inherent variability across different wheat cultivars and independent experimental setups necessitates caution. Third, although our independent qRT-PCR experiments effectively confirm the transcriptional responsiveness of selected candidates in the ‘SuMai 3’ cultivar, an increase in transcript abundance does not directly equate to physiological stress tolerance or an increase in endogenous melatonin concentrations. Consequently, our proposed evolutionary trajectories, regulatory feedback loops, and potential sub-functionalization models remain hypotheses. Future studies incorporating transgenic functional validation, enzymatic assays, and targeted metabolomics are essential to definitively establish the biological roles of these candidate genes in wheat.

Similarly, the abundance of JA-responsive elements (TGACG motifs) in the promoters of specific candidates like *II_TraesCS3A02G032700* suggests a mechanistic link to JA signaling. Because melatonin application increases endogenous JA content in drought-stressed soybean [55], we hypothesize that these *TaeASMT*s respond to JA signals, forming a co-regulatory feedback loop for plant defense. Future experimental validation will confirm whether these candidate genes actively integrate into broader hormone signaling networks. Finally, a schematic workflow summarizing the comprehensive analytical pipeline and research logic of this study is provided in Figure S10.

## 5. Conclusions

Through a comprehensive genome-wide analysis, this study identified 156 *ASMT* gene family members in wheat and elucidated their evolutionary history, revealing that polyploidization and segmental duplication were the primary drivers of family expansion. Our structural analysis proposed a hypothetical evolutionary model postulating that the basal Subfamily III might have diverged into Subfamilies I and II via specific exon fusion events. Functionally, this study identifies specific *TaeASMTs* as strong candidate genes responsive to abiotic stress. Transcriptomic profiling and independent qRT-PCR confirmation identified specific candidate genes that are strongly induced by drought and salt stress. We further revealed that this responsiveness correlates with the enrichment of hormone-responsive elements in their promoters, suggesting a hypothetical feedback loop between melatonin and ABA signaling that warrants future investigation.

Looking forward, while keeping the aforementioned experimental limitations in mind, these findings provide initial candidate genes for genetic improvement in wheat breeding. The high-confidence stress-responsive candidates identified here hold great potential for future utilization in Marker-Assisted Selection (MAS) programs to screen for superior alleles in wheat germplasm. Furthermore, genetic engineering approaches, such as CRISPR/Cas9-mediated editing to optimize promoter activity or the overexpression of these key *ASMT* genes, offer a promising strategy to enhance endogenous melatonin biosynthesis, thereby boosting antioxidant capacity and plant stress tolerance. Ultimately, this research bridges the gap between genomic identification and functional application, providing critical genetic resources for developing climate-resilient wheat varieties in the face of global environmental changes.

## Figures and Tables

**Table 1:** The number of ASMT gene family in 15 plants.

Species	Number	Atypical ASMT
*Chlamydomonas reinhardtii*	3	0
*Physcomitrium patens*	4	1
*Selaginella moellendorffii*	22	11
*Amborella trichopoda*	10	7
*Vitis vinifera*	35	7
*Arabidopsis thaliana*	17	0
*Oryza sativa*	36	8
*Zea mays*	29	0
*Brachypodium distachyon*	22	0
*Aegilops tauschii*	47	2
*Triticum urartu*	37	4
*Triticum dicoccoides*	77	12
*Triticum turgidum*	58	4
*Triticum spelta*	159	7
*Triticum aestivum*	156	12

## Data Availability

The genomes, proteomes and GFF files of the investigated plants are available in Ensembl Plants 59-release (http://plants.ensembl.org/). The accession numbers of plants are *T. aestivum* (IWGSC), *T. spelta* (PGSBv2.0), *T. turgidum* (Svevo.v1), *T. dicoccoides* (WEWSeq_v.1.0), *T. urartu* (IGDB), *Ae. tauschii* (Aet_v4.0), *B. distachyon* (v3.0), *Z. mays* (Zm-B73-REFERENCE-NAM-5.0), *O. sativa* (ASM465v1), *A. thaliana* (TAIR10), *V. vinifera* (ASM3070453v1), *A. trichopoda* (AMTR1.0), *S. moellendorffii* (v1.0), *P. patens* (Phypa_V3) and *C. reinhardtii* (v5.5). *Public wheat* (T. aestivum), *Ae. tauschii* and *B. distachyon* transcriptome expression datasets were retrieved from the SRA database of NCBI. The SRA accession numbers of transcriptomes are 1077667, 994277, 971860, 936261, 1097929, 1183537, 1006331, 914331, 972750 and 782527.

## Supplementary Materials

The following supporting information can be downloaded at https://www.mdpi.com/article/10.3390/biology15161430/s1, Figure S1. Phylogenetic classification of *ASMT*s from 15 representative plants (*C. reinhardtii*, *P. patens*, *S*. *moellendorffii*, *A. trichopoda*, *V. vinifera*, *A. thaliana*, *O. sativa*, *Z. mays*, *B. distachyon*, *Ae. tauschii*, *T. urartu*, *T. dicoccoides*, *T. turgidum*, *T. spelta* and *T. aestivum*) by using the four methods. (A) NJ (JTT); (B) NJ (p-distance); (C) ML (LG + I + G + F); (D) Bayes. Figure S2. Exon–intron and domain diagrams of *ASMT*s in 15 plants. The descriptions of the domain and exon phases are the same as those in Figure 2. The lengths of the boxes and lines are scaled based on the lengths of the genes. Figure S3. Conserved exon–intron and domain diagrams of *ASMT*s in *T. aestivum*, *B. distachyon*, *V. vinifera*, *A. trichopoda*, *S. moellendorffii* and *P. patens*. The descriptions of the domain and exon phases are the same as those in Figure 2. The lengths of the boxes and lines are scaled based on the lengths of the genes. Figure S4. Domain diagrams of *ASMT*s in 15 plants. Figure S5. Chromosome locations of *ASMT*s in *T. aestivum*. Chromosomal locations of *T. aestivum ASMT*s. Yellow boxes denote *T. aestivum ASMT* genes. Figure S6. Chromosomal locations of the tandemly arrayed *T. aestivum* ASMT genes. Figure S7. Collinearity events of *T. aestivum ASMT* genes. Collinearity events with Ks values of 0–0.35. Figure S8. Collinearity (Ks values) of *T. aestivum ASMT* genes. Collinearity events of duplicated *ASMT*s in the *T. aestivum* genome. The red bars denote the collinearity events contributed by polyploidizations (Ks values 0–0.35). The blue bars denote the other collinearity events. Figure S9. Heatmap of the expression patterns of individual *T. aestivum ASMT* genes under abiotic and biotic stress treatments. (A) drought stress; (B) Heat stress; (C) salinity stress; (D) waterlogging stress; (E) *F. graminearum* treatment; (F) stripe rust treatment; (G) powdery mildew treatment and (H) *Puccinia triticina* treatment. Figure S10. Schematic workflow of the systematic analysis of the *ASMT* gene family in wheat. The pipeline is organized into four major phases: (1) Genome-wide identification and rigorous quality control across 15 plant species; (2) Evolutionary and structural characterization including phylogenetic clustering and exon–intron phase analysis; (3) Expression profiling using transcriptomic data under various abiotic and biotic stresses; and (4) Functional candidate identification and regulatory mechanism prediction through independent qRT-PCR confirmation and promoter cis-element analysis. Table S1. Public wheat (*T. aestivum*) RNA-seq expression data for use. Table S2. Primers used in the qRT-PCR analysis. Table S3. *ASMT* members containing 1-2 *ASMT* domains in 15 plants. Table S4. Atypical *ASMT* members containing 1-2 *ASMT* domains in 15 plants. Table S5. The *ASMT* subfamily classification in *Arabidopsis thaliana*. Table S6. The classification of *ASMT* gene family in 15 plants. Table S7. Chromosome locations of *T. aestivum ASMT* genes. Table S8. Chromosome locations of *T. aestivum* tandem duplication *ASMT*s. Table S9. Collinearity events and Ka/Ks values of *T. aestivum ASMT* genes. Table S10. Collinearity events and Ka/Ks values of *ASMT*s among *T. aestivum, B. distachyon* and *O. sativa*. Sheets 1 show the Ka/Ks values of collinearity events from *ASMT*s and all genes between *T. aestivum* and *O. sativa*. Similarly, sheet 2 showed *T. aestivum* and *B. distachyon*. Table S11A. Normalized gene expression log2-fold change (treatment vs. control) values of T. aestivum *ASMT* genes from transcriptomes under abiotic stress treatments. Sheet 1 was about drought stress. Sheet 2 was about heat stress. Sheet 3 was about salinity stress. Sheet 4 was about waterlogging stress. Table S11B. Normalized gene expression log2-fold fold change (treatment vs. control) values of *T. aestivum ASMT* genes from transcriptomes under biotic stress treatments. Sheet 1 was about *F. graminearum* treatment. Sheet 2 was about stripe rust. Sheet 3 was about powdery mildew treatment. Sheet 4 was about *Puccinia triticina* treatment.

## Abbreviations

The following abbreviations are used in this manuscript:

ASMT	Acetylserotonin O-methyltransferase
HMM	Hidden Markov model
ABRE	Abscisic acid response element
LTR	Low-temperature responsive
qRT-PCR	Quantitative real-time PCR
SNAT	Serotonin N-acetyltransferase
TDC	Tryptophan decarboxylase
TPH	Tryptophan hydroxylase
T5H	Tryptamine 5-hydroxylase
ROS	Reactive oxygen species
ABA	Abscisic acid
CREs	Cis-regulatory elements
NJ	Neighbour-joining
JTT	Jones–Taylor–Thorton
ML	Maximum likelihood
AIC	Akaike information criterion
MCMC	Markov Chain Monte Carlo
GFF	General feature format
Ka	Non-synonymous substitution
Ks	Synonymous substitution
SRA	Sequence Read Archives
NCBI	National Center for Biotechnology Information
STAR	Spliced transcripts alignment to a reference
RSEM	RNA-Seq by expectation maximization
TaeASMT	T. aestivum ASMT
DEGs	Differentially expressed genes
TFs	Transcription factors
ARE	Anaerobic responsive element
MBS	MYB binding site
WGD	Whole-genome duplication
JA	Jasmonic acid
