# Supplementary material for: Genome-Wide Identification of the ASMT Gene Family and Expression Analysis of Wheat ASMTs Under Abiotic and Biotic Stress
Source: Biology (Basel). 2026 Aug 19;15(16):1430. doi: 10.3390/biology15161430 (PMC13509220; doi:10.3390/biology15161430)

A

NJ(JTT)

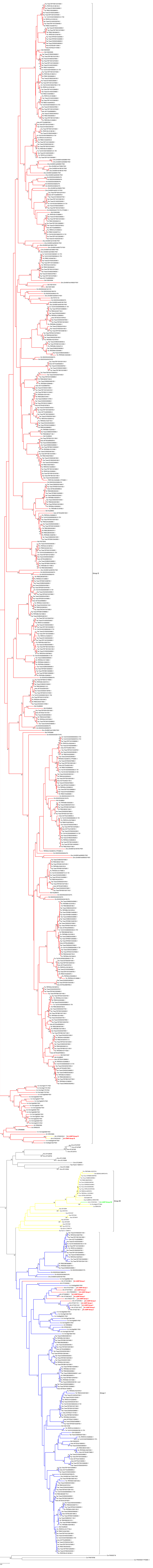

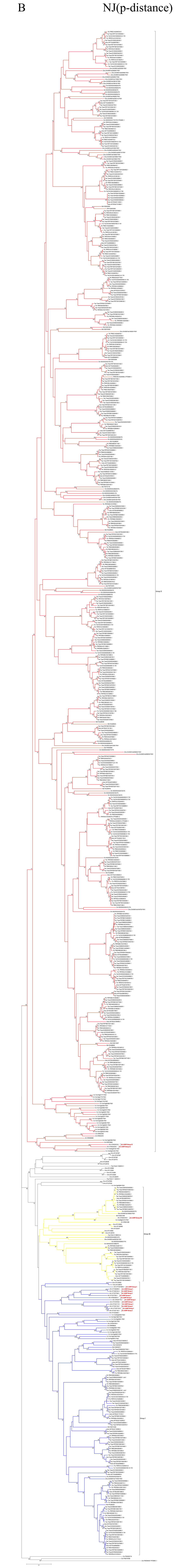
B
NJ(p-distance)

C

ML(JTT)

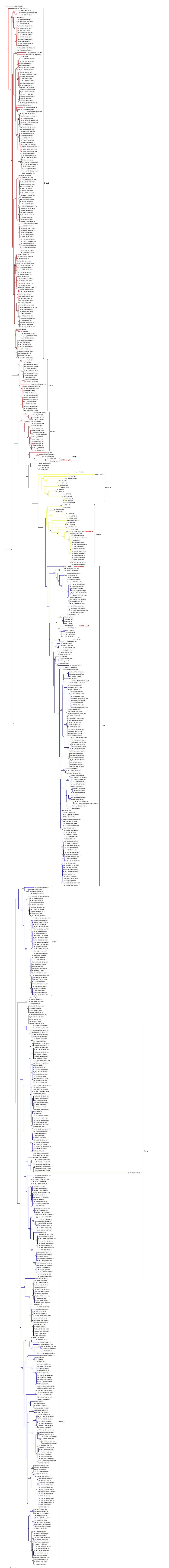

D

Bayes

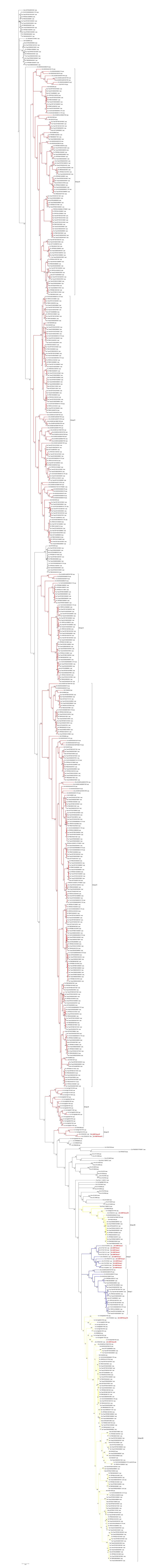

Supplement: Supplementary file 1 [file biology-15-01430-s001.zip › Figure/Figure S1.pdf]
