# Supplementary material for: Genome-Wide Identification of the ASMT Gene Family and Expression Analysis of Wheat ASMTs Under Abiotic and Biotic Stress
Source: Biology (Basel). 2026 Aug 19;15(16):1430. doi: 10.3390/biology15161430 (PMC13509220; doi:10.3390/biology15161430)
Supplement: Supplementary file 1 [file biology-15-01430-s001.zip › Figure/Figure S10.pdf]

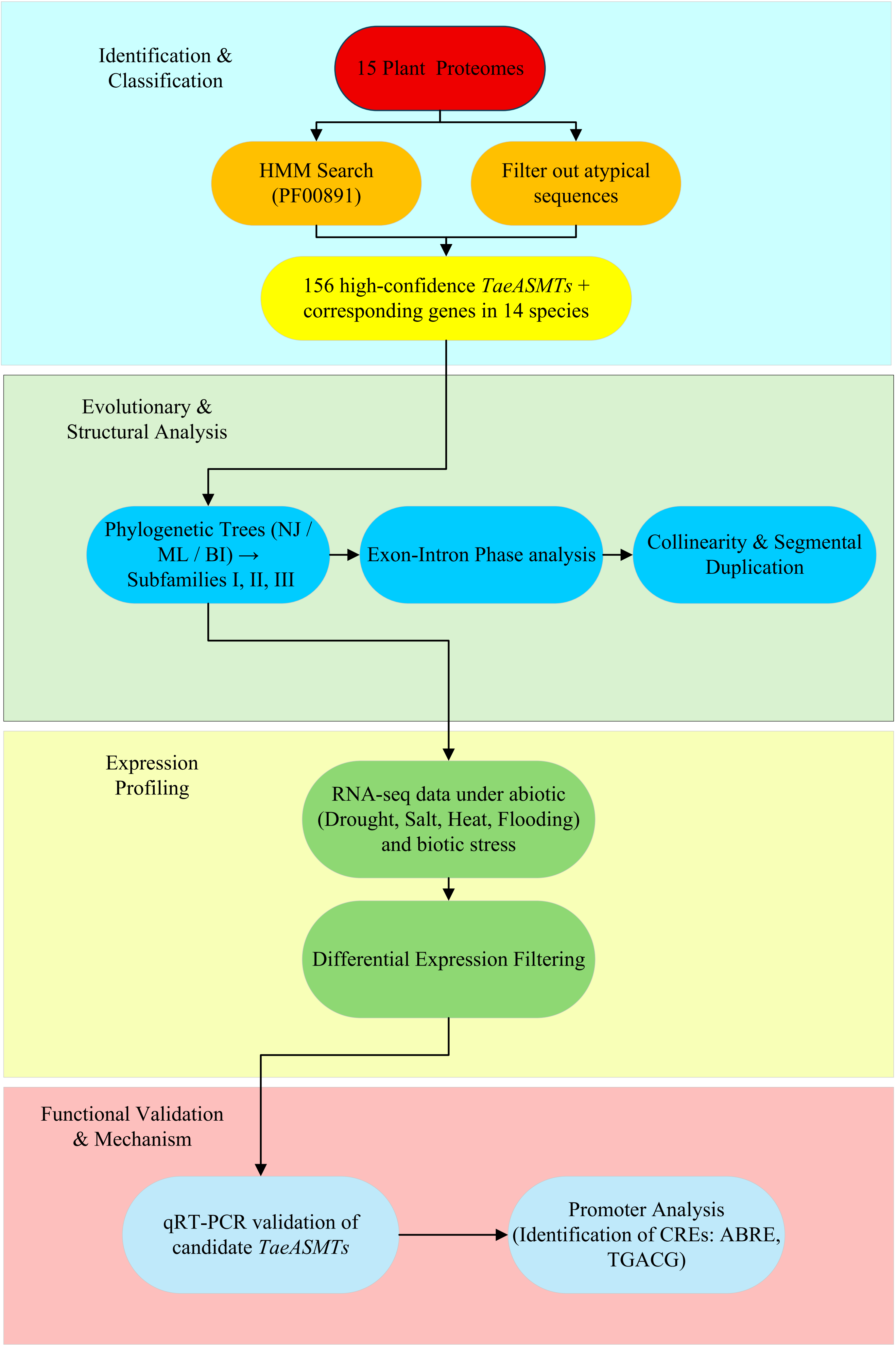
Identification & Classification
15 Plant Proteomes
HMM Search (PF00891)
Filter out atypical sequences
156 high-confidence *TaeASMTs* + corresponding genes in 14 species
Evolutionary & Structural Analysis
Phylogenetic Trees (NJ / ML / BI) → Subfamilies I, II, III
Exon-Intron Phase analysis
Collinearity & Segmental Duplication
Expression Profiling
RNA-seq data under abiotic (Drought, Salt, Heat, Flooding) and biotic stress
Differential Expression Filtering
Functional Validation & Mechanism
qRT-PCR validation of candidate *TaeASMTs*
Promoter Analysis (Identification of CREs: ABRE, TGACG)
