# Supplementary material for: Genome-Wide Identification of the ASMT Gene Family and Expression Analysis of Wheat ASMTs Under Abiotic and Biotic Stress
Source: Biology (Basel). 2026 Aug 19;15(16):1430. doi: 10.3390/biology15161430 (PMC13509220; doi:10.3390/biology15161430)

T.ae ASMT I subfamily exon−intron and kinase domain diagram (part 1)

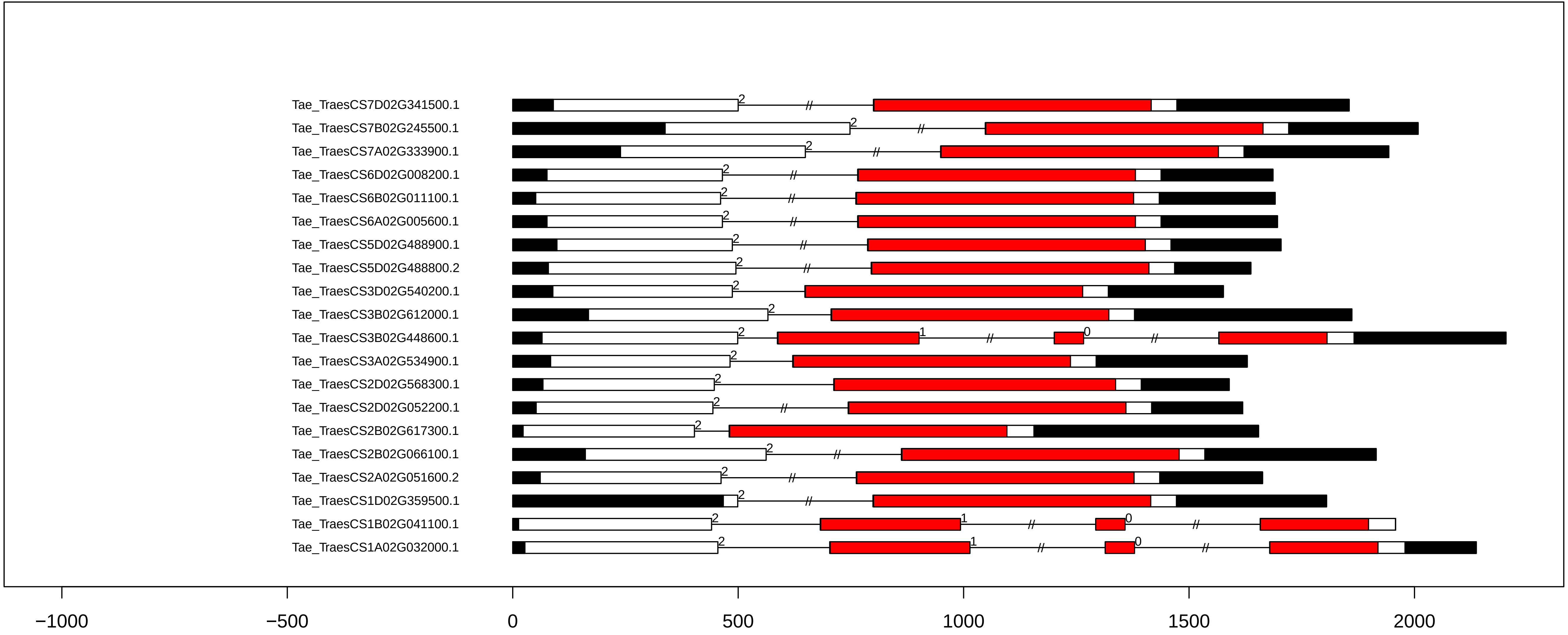

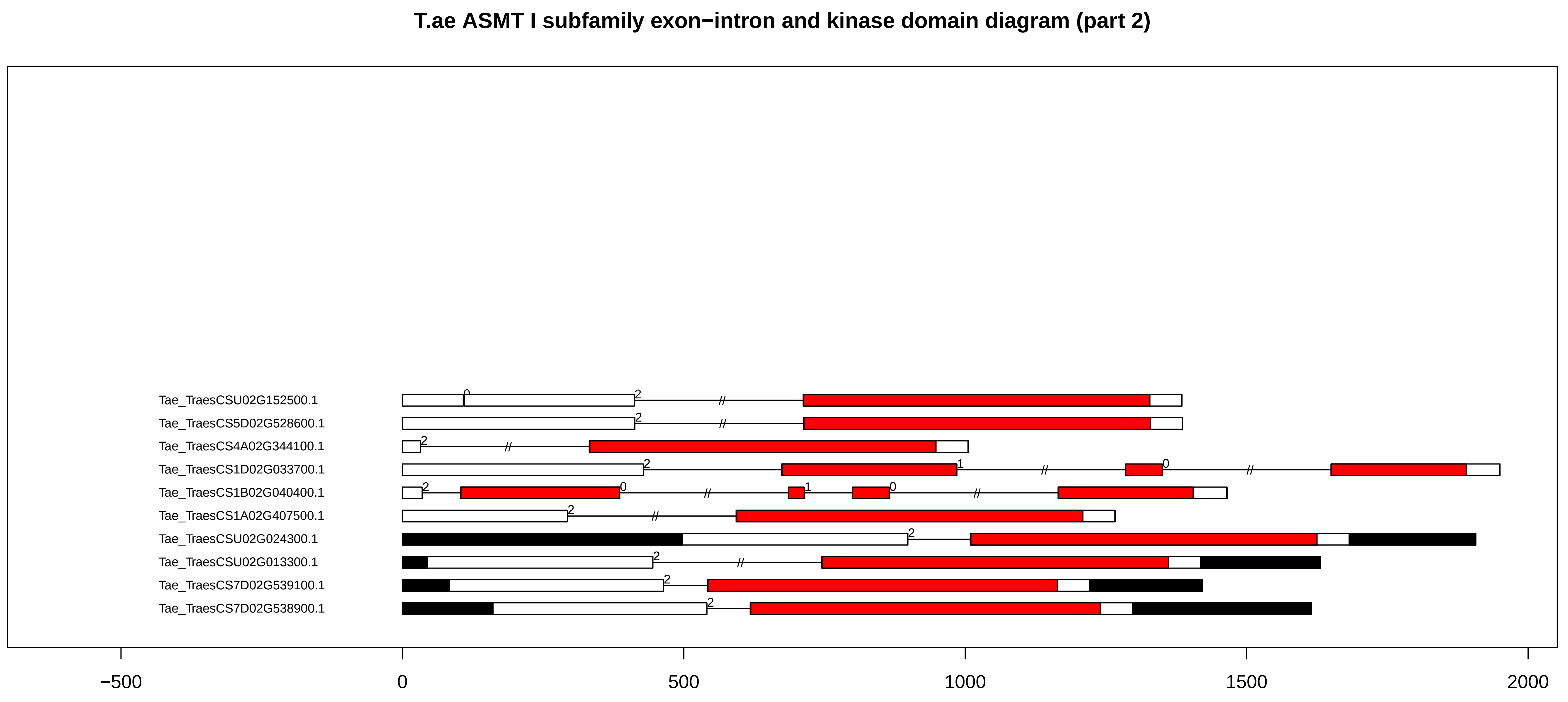

T.ae ASMT I subfamily exon−intron and kinase domain diagram (part 2)
Tae_TraesCSU02G152500.1
Tae_TraesCS5D02G528600.1
Tae_TraesCS4A02G344100.1
Tae_TraesCS1D02G033700.1
Tae_TraesCS1B02G040400.1
Tae_TraesCS1A02G407500.1
Tae_TraesCSU02G024300.1
Tae_TraesCSU02G013300.1
Tae_TraesCS7D02G539100.1
Tae_TraesCS7D02G538900.1
−500
0
500
1000
1500
2000

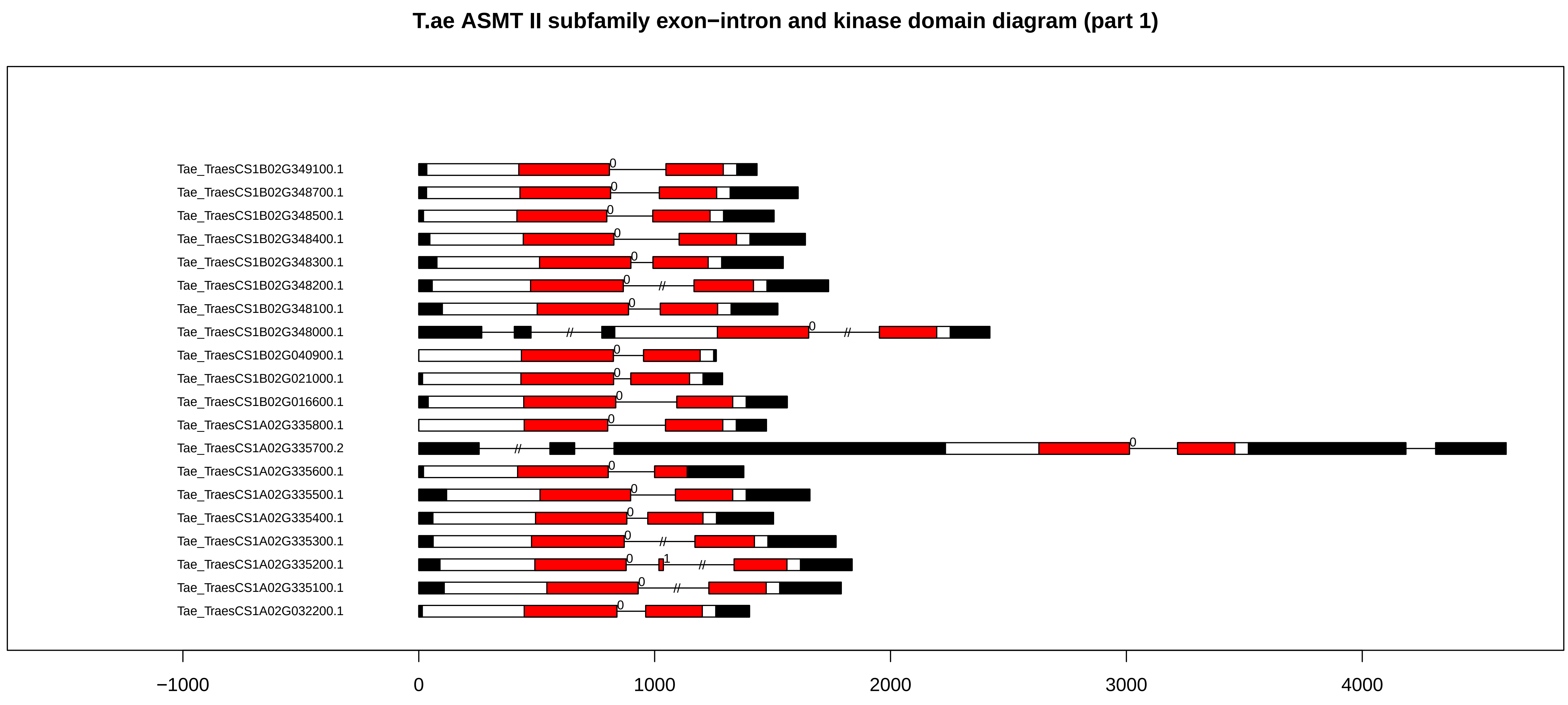

T.ae ASMT II subfamily exon−intron and kinase domain diagram (part 1)
Tae_TraesCS1B02G349100.1
Tae_TraesCS1B02G348700.1
Tae_TraesCS1B02G348500.1
Tae_TraesCS1B02G348400.1
Tae_TraesCS1B02G348300.1
Tae_TraesCS1B02G348200.1
Tae_TraesCS1B02G348100.1
Tae_TraesCS1B02G348000.1
Tae_TraesCS1B02G040900.1
Tae_TraesCS1B02G021000.1
Tae_TraesCS1B02G016600.1
Tae_TraesCS1A02G335800.1
Tae_TraesCS1A02G335700.2
Tae_TraesCS1A02G335600.1
Tae_TraesCS1A02G335500.1
Tae_TraesCS1A02G335400.1
Tae_TraesCS1A02G335300.1
Tae_TraesCS1A02G335200.1
Tae_TraesCS1A02G335100.1
Tae_TraesCS1A02G032200.1
−1000
0
1000
2000
3000
4000

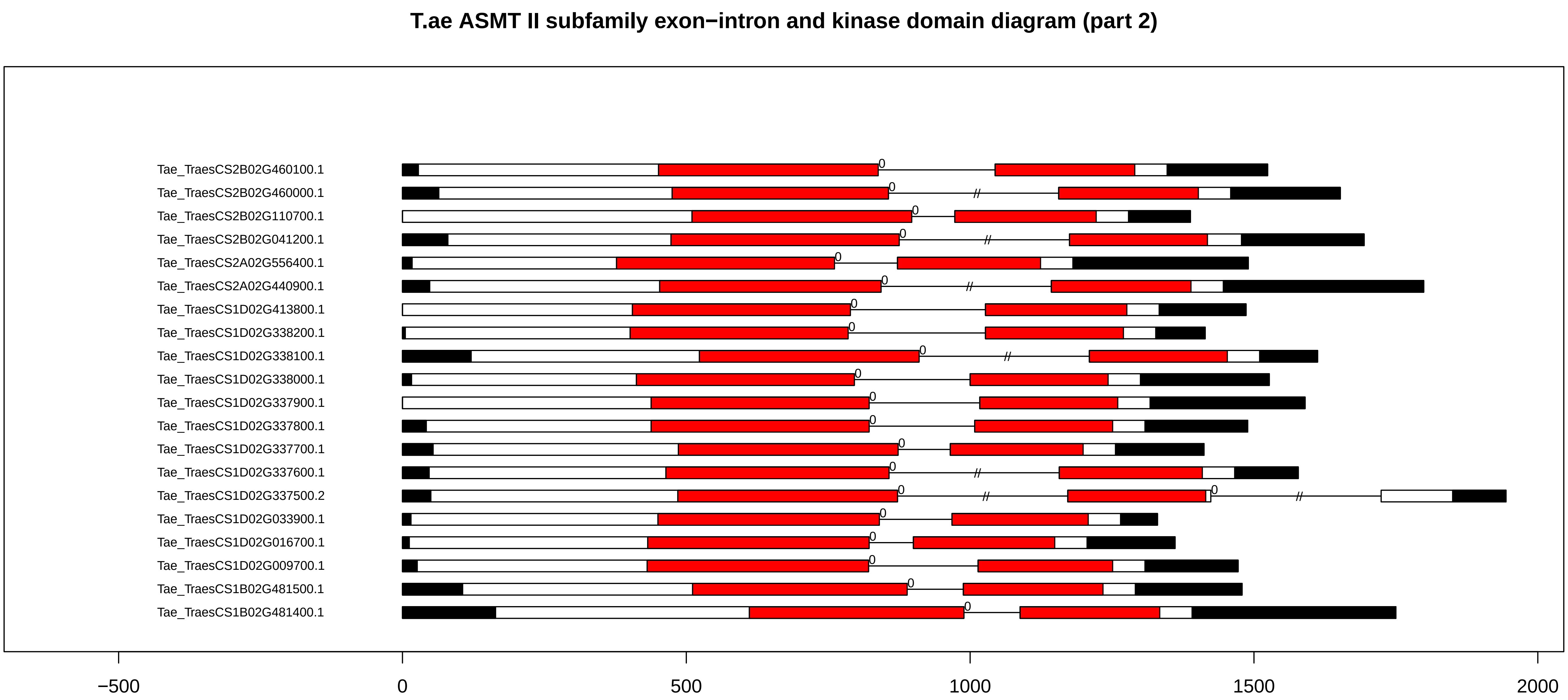
T.ae ASMT II subfamily exon−intron and kinase domain diagram (part 2)
Tae_TraesCS2B02G460100.1
Tae_TraesCS2B02G460000.1
Tae_TraesCS2B02G110700.1
Tae_TraesCS2B02G041200.1
Tae_TraesCS2A02G556400.1
Tae_TraesCS2A02G440900.1
Tae_TraesCS1D02G413800.1
Tae_TraesCS1D02G338200.1
Tae_TraesCS1D02G338100.1
Tae_TraesCS1D02G338000.1
Tae_TraesCS1D02G337900.1
Tae_TraesCS1D02G337800.1
Tae_TraesCS1D02G337700.1
Tae_TraesCS1D02G337600.1
Tae_TraesCS1D02G337500.2
Tae_TraesCS1D02G033900.1
Tae_TraesCS1D02G016700.1
Tae_TraesCS1D02G009700.1
Tae_TraesCS1B02G481500.1
Tae_TraesCS1B02G481400.1
−500
0
500
1000
1500
2000

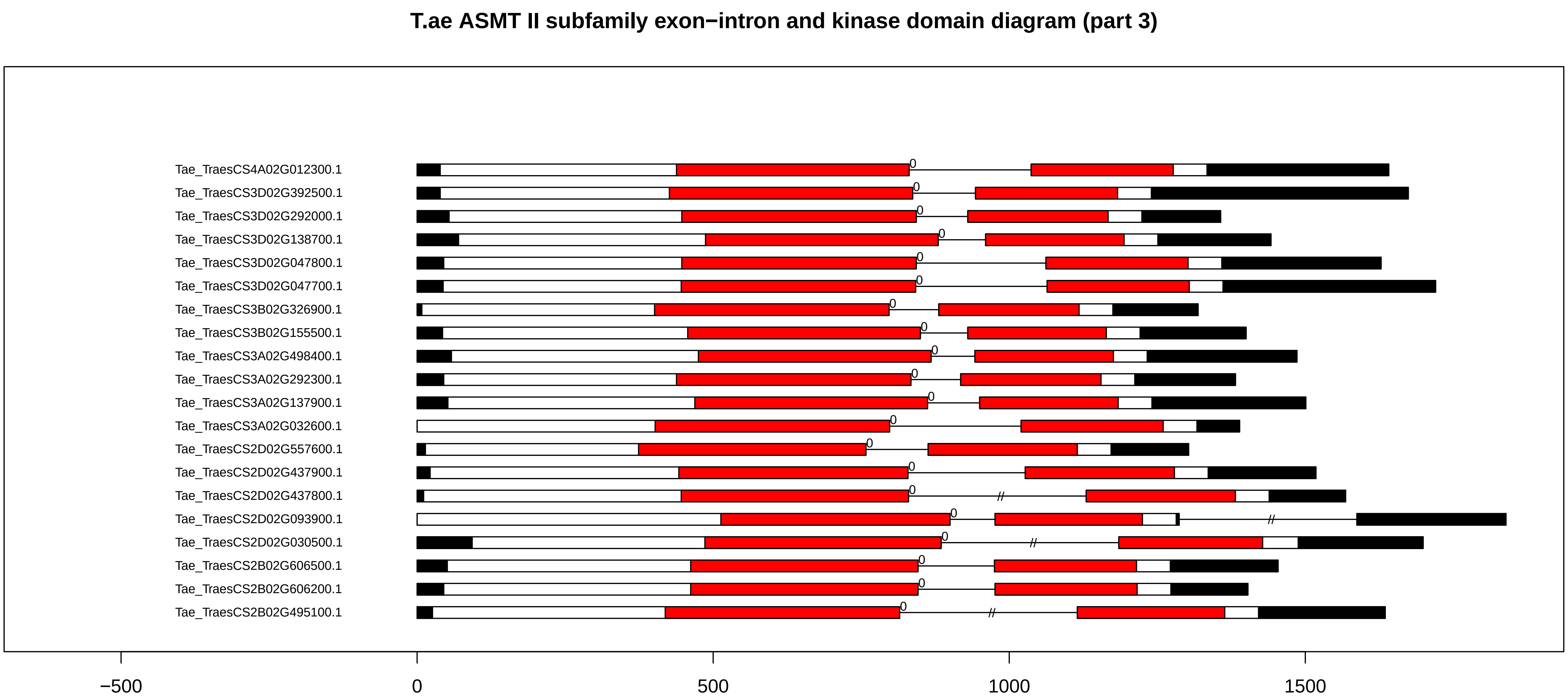
T.ae ASMT II subfamily exon−intron and kinase domain diagram (part 3)
Tae_TraesCS4A02G012300.1
Tae_TraesCS3D02G392500.1
Tae_TraesCS3D02G292000.1
Tae_TraesCS3D02G138700.1
Tae_TraesCS3D02G047800.1
Tae_TraesCS3D02G047700.1
Tae_TraesCS3B02G326900.1
Tae_TraesCS3B02G155500.1
Tae_TraesCS3A02G498400.1
Tae_TraesCS3A02G292300.1
Tae_TraesCS3A02G137900.1
Tae_TraesCS3A02G032600.1
Tae_TraesCS2D02G557600.1
Tae_TraesCS2D02G437900.1
Tae_TraesCS2D02G437800.1
Tae_TraesCS2D02G093900.1
Tae_TraesCS2D02G030500.1
Tae_TraesCS2B02G606500.1
Tae_TraesCS2B02G606200.1
Tae_TraesCS2B02G495100.1
−500
0
500
1000
1500

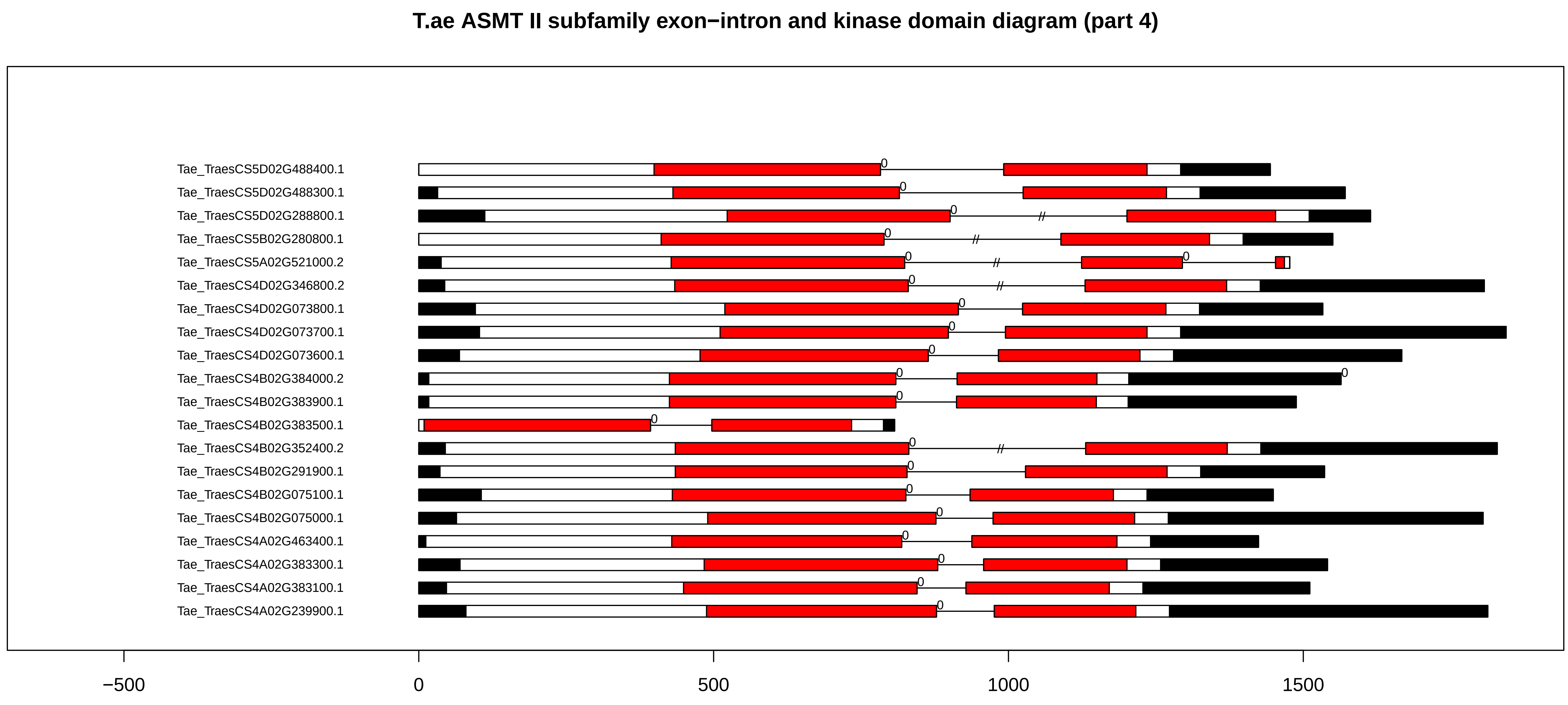

T.ae ASMT II subfamily exon−intron and kinase domain diagram (part 4)
Tae_TraesCS5D02G488400.1
Tae_TraesCS5D02G488300.1
Tae_TraesCS5D02G288800.1
Tae_TraesCS5B02G280800.1
Tae_TraesCS5A02G521000.2
Tae_TraesCS4D02G346800.2
Tae_TraesCS4D02G073800.1
Tae_TraesCS4D02G073700.1
Tae_TraesCS4D02G073600.1
Tae_TraesCS4B02G384000.2
Tae_TraesCS4B02G383900.1
Tae_TraesCS4B02G383500.1
Tae_TraesCS4B02G352400.2
Tae_TraesCS4B02G291900.1
Tae_TraesCS4B02G075100.1
Tae_TraesCS4B02G075000.1
Tae_TraesCS4A02G463400.1
Tae_TraesCS4A02G383300.1
Tae_TraesCS4A02G383100.1
Tae_TraesCS4A02G239900.1
−500
0
500
1000
1500

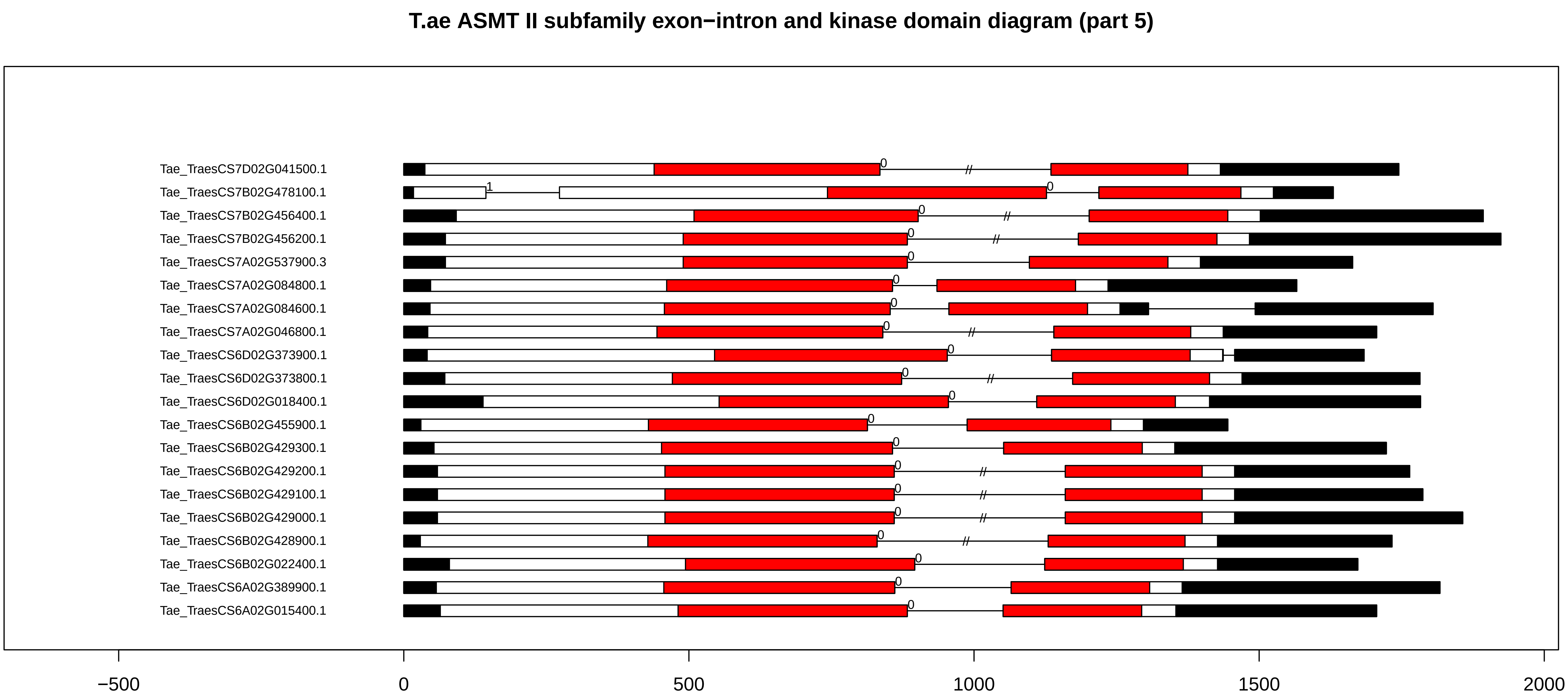
T.ae ASMT II subfamily exon−intron and kinase domain diagram (part 5)
Tae_TraesCS7D02G041500.1
Tae_TraesCS7B02G478100.1
Tae_TraesCS7B02G456400.1
Tae_TraesCS7B02G456200.1
Tae_TraesCS7A02G537900.3
Tae_TraesCS7A02G084800.1
Tae_TraesCS7A02G084600.1
Tae_TraesCS7A02G046800.1
Tae_TraesCS6D02G373900.1
Tae_TraesCS6D02G373800.1
Tae_TraesCS6D02G018400.1
Tae_TraesCS6B02G455900.1
Tae_TraesCS6B02G429300.1
Tae_TraesCS6B02G429200.1
Tae_TraesCS6B02G429100.1
Tae_TraesCS6B02G429000.1
Tae_TraesCS6B02G428900.1
Tae_TraesCS6B02G022400.1
Tae_TraesCS6A02G389900.1
Tae_TraesCS6A02G015400.1
−500
0
500
1000
1500
2000

T.ae ASMT II subfamily exon−intron and kinase domain diagram (part 6)

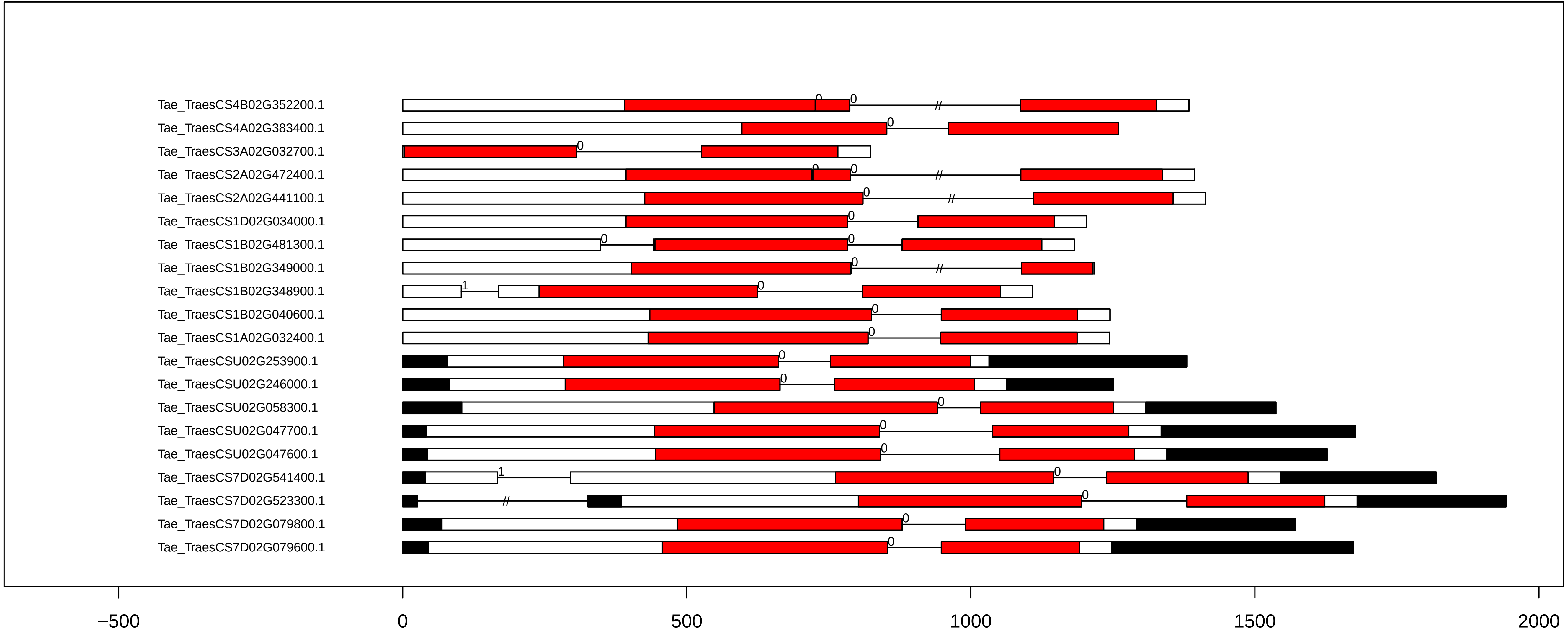

T.ae ASMT II subfamily exon−intron and kinase domain diagram (part 7)

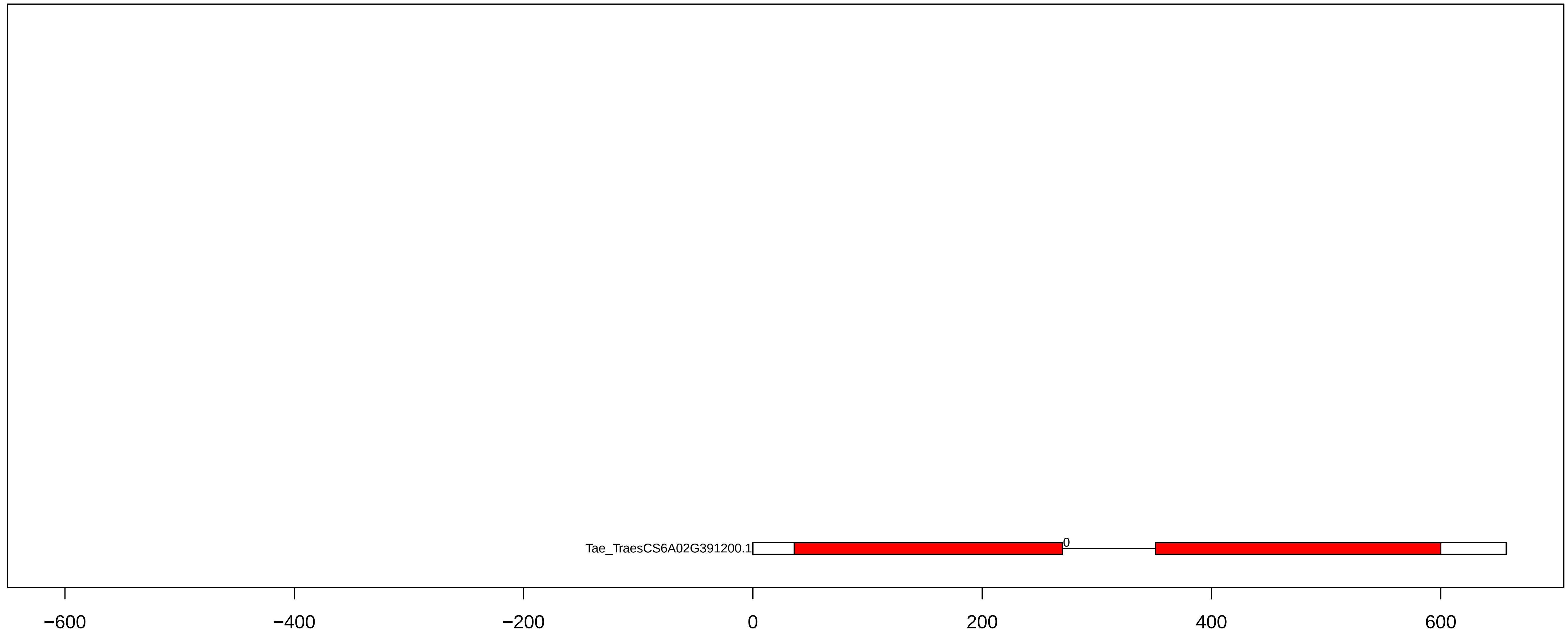

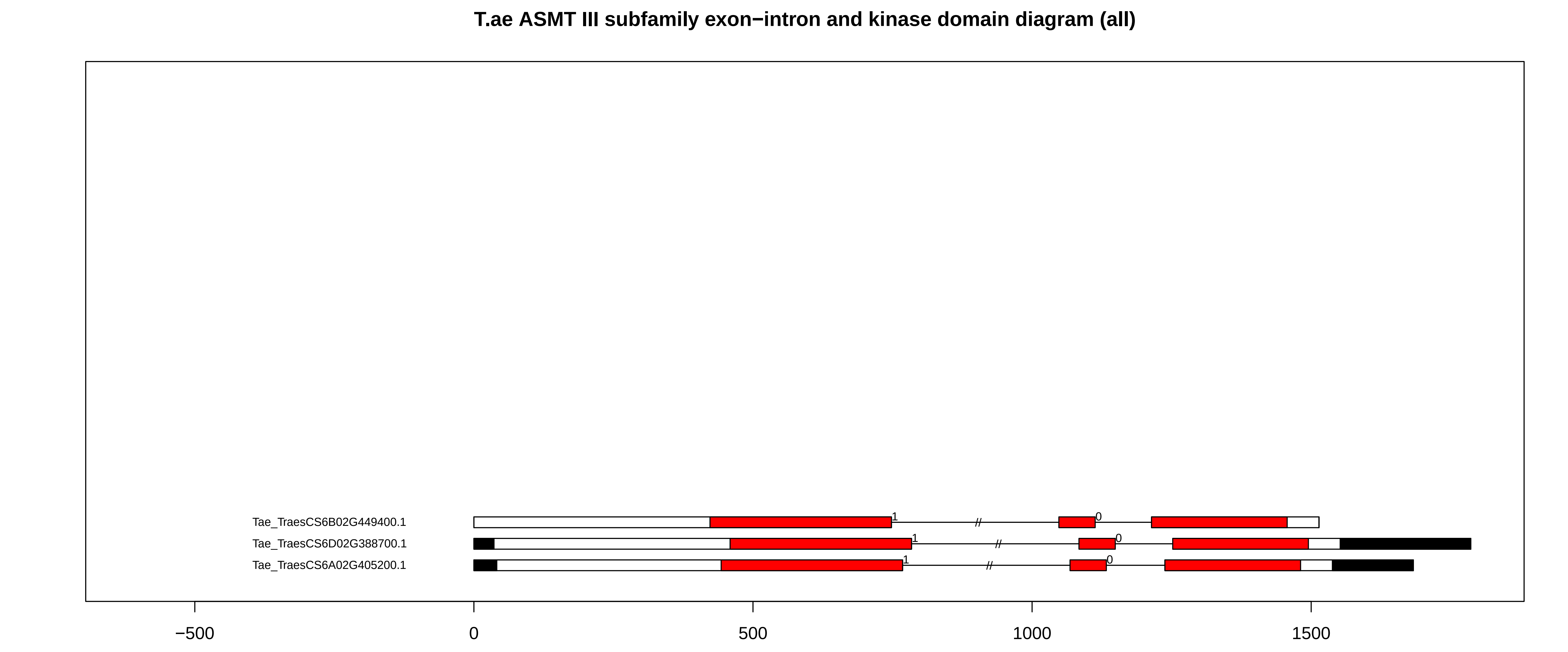
T.ae ASMT III subfamily exon−intron and kinase domain diagram (all)
Tae_TraesCS6B02G449400.1
Tae_TraesCS6D02G388700.1
Tae_TraesCS6A02G405200.1
1
0
1
0
1
0
−500
0
500
1000
1500

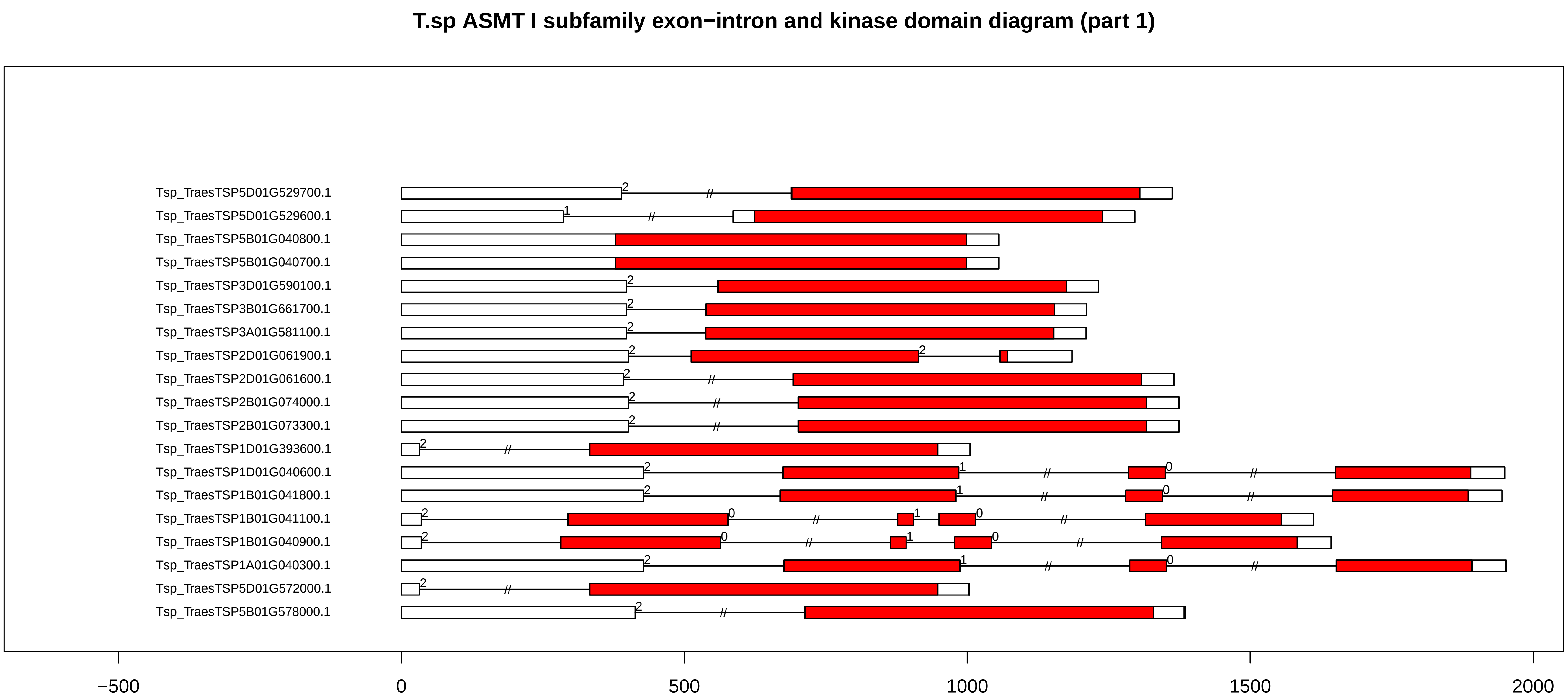
T.sp ASMT I subfamily exon−intron and kinase domain diagram (part 1)
Tsp_TraesTSP5D01G529700.1
Tsp_TraesTSP5D01G529600.1
Tsp_TraesTSP5B01G040800.1
Tsp_TraesTSP5B01G040700.1
Tsp_TraesTSP3D01G590100.1
Tsp_TraesTSP3B01G661700.1
Tsp_TraesTSP3A01G581100.1
Tsp_TraesTSP2D01G061900.1
Tsp_TraesTSP2D01G061600.1
Tsp_TraesTSP2B01G074000.1
Tsp_TraesTSP2B01G073300.1
Tsp_TraesTSP1D01G393600.1
Tsp_TraesTSP1D01G040600.1
Tsp_TraesTSP1B01G041800.1
Tsp_TraesTSP1B01G041100.1
Tsp_TraesTSP1B01G040900.1
Tsp_TraesTSP1A01G040300.1
Tsp_TraesTSP5D01G572000.1
Tsp_TraesTSP5B01G578000.1
−500
0
500
1000
1500
2000

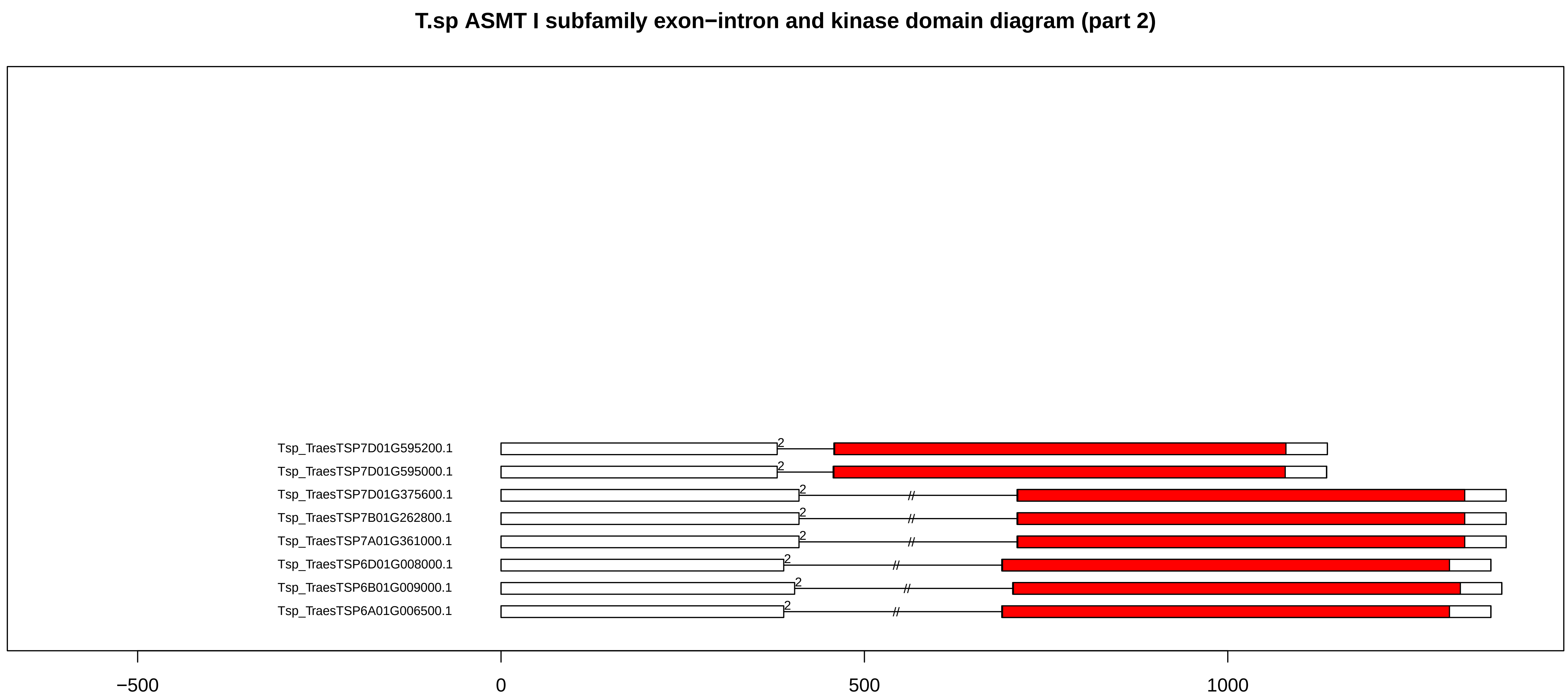

T.sp ASMT I subfamily exon−intron and kinase domain diagram (part 2)
Tsp_TraesTSP7D01G595200.1
Tsp_TraesTSP7D01G595000.1
Tsp_TraesTSP7D01G375600.1
Tsp_TraesTSP7B01G262800.1
Tsp_TraesTSP7A01G361000.1
Tsp_TraesTSP6D01G008000.1
Tsp_TraesTSP6B01G009000.1
Tsp_TraesTSP6A01G006500.1
−500
0
500
1000

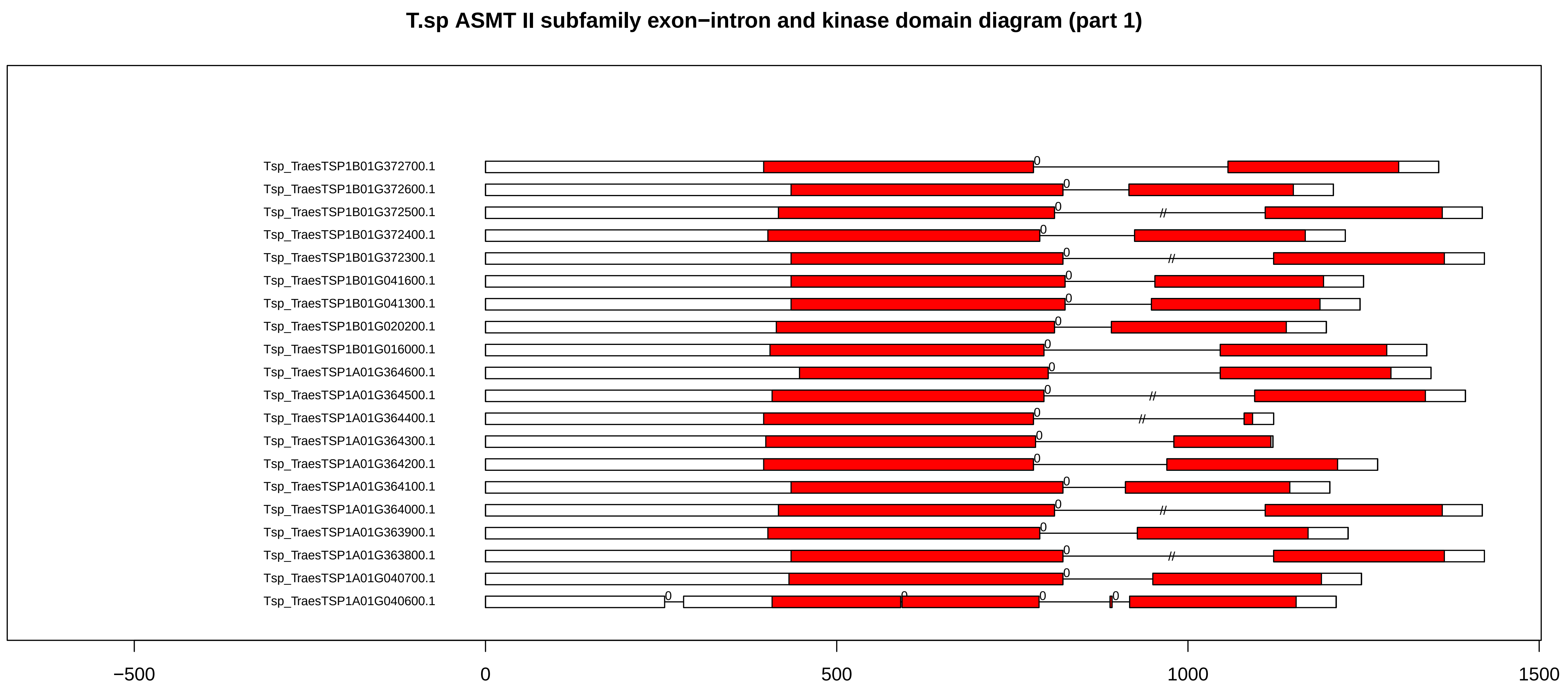
T.sp ASMT II subfamily exon−intron and kinase domain diagram (part 1)
Tsp_TraesTSP1B01G372700.1
Tsp_TraesTSP1B01G372600.1
Tsp_TraesTSP1B01G372500.1
Tsp_TraesTSP1B01G372400.1
Tsp_TraesTSP1B01G372300.1
Tsp_TraesTSP1B01G041600.1
Tsp_TraesTSP1B01G041300.1
Tsp_TraesTSP1B01G020200.1
Tsp_TraesTSP1B01G016000.1
Tsp_TraesTSP1A01G364600.1
Tsp_TraesTSP1A01G364500.1
Tsp_TraesTSP1A01G364400.1
Tsp_TraesTSP1A01G364300.1
Tsp_TraesTSP1A01G364200.1
Tsp_TraesTSP1A01G364100.1
Tsp_TraesTSP1A01G364000.1
Tsp_TraesTSP1A01G363900.1
Tsp_TraesTSP1A01G363800.1
Tsp_TraesTSP1A01G040700.1
Tsp_TraesTSP1A01G040600.1
−500
0
500
1000
1500

T.sp ASMT II subfamily exon−intron and kinase domain diagram (part 2)

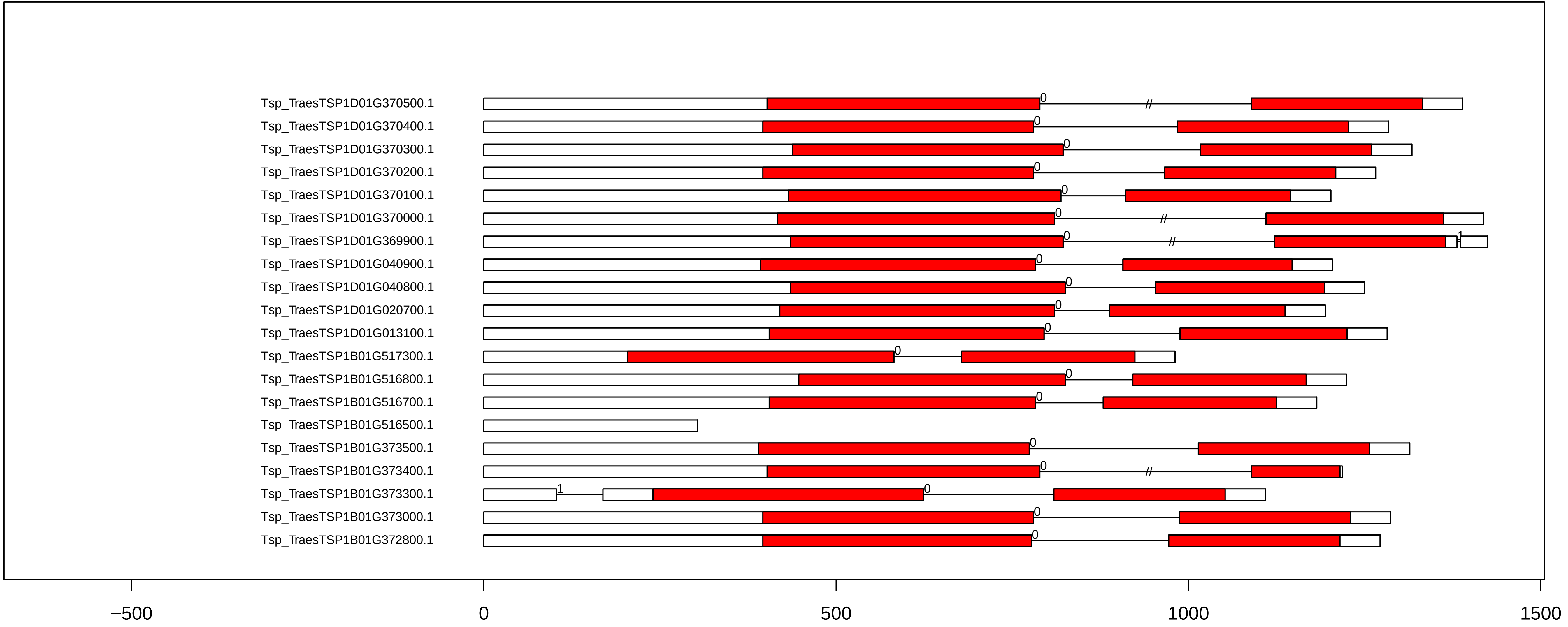

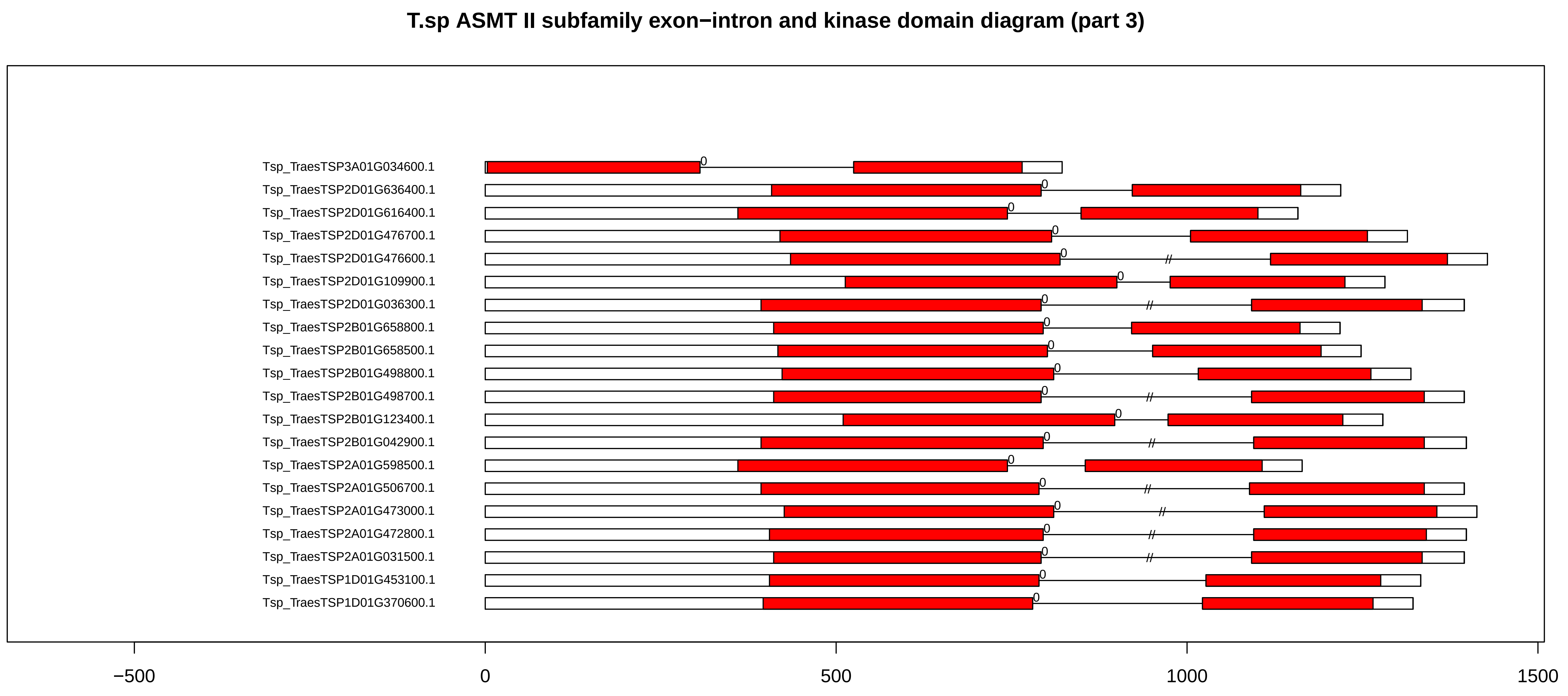

T.sp ASMT II subfamily exon−intron and kinase domain diagram (part 3)
Tsp_TraesTSP3A01G034600.1
Tsp_TraesTSP2D01G636400.1
Tsp_TraesTSP2D01G616400.1
Tsp_TraesTSP2D01G476700.1
Tsp_TraesTSP2D01G476600.1
Tsp_TraesTSP2D01G109900.1
Tsp_TraesTSP2D01G036300.1
Tsp_TraesTSP2B01G658800.1
Tsp_TraesTSP2B01G658500.1
Tsp_TraesTSP2B01G498800.1
Tsp_TraesTSP2B01G498700.1
Tsp_TraesTSP2B01G123400.1
Tsp_TraesTSP2B01G042900.1
Tsp_TraesTSP2A01G598500.1
Tsp_TraesTSP2A01G506700.1
Tsp_TraesTSP2A01G473000.1
Tsp_TraesTSP2A01G472800.1
Tsp_TraesTSP2A01G031500.1
Tsp_TraesTSP1D01G453100.1
Tsp_TraesTSP1D01G370600.1
−500
0
500
1000
1500

T.sp ASMT II subfamily exon−intron and kinase domain diagram (part 4)

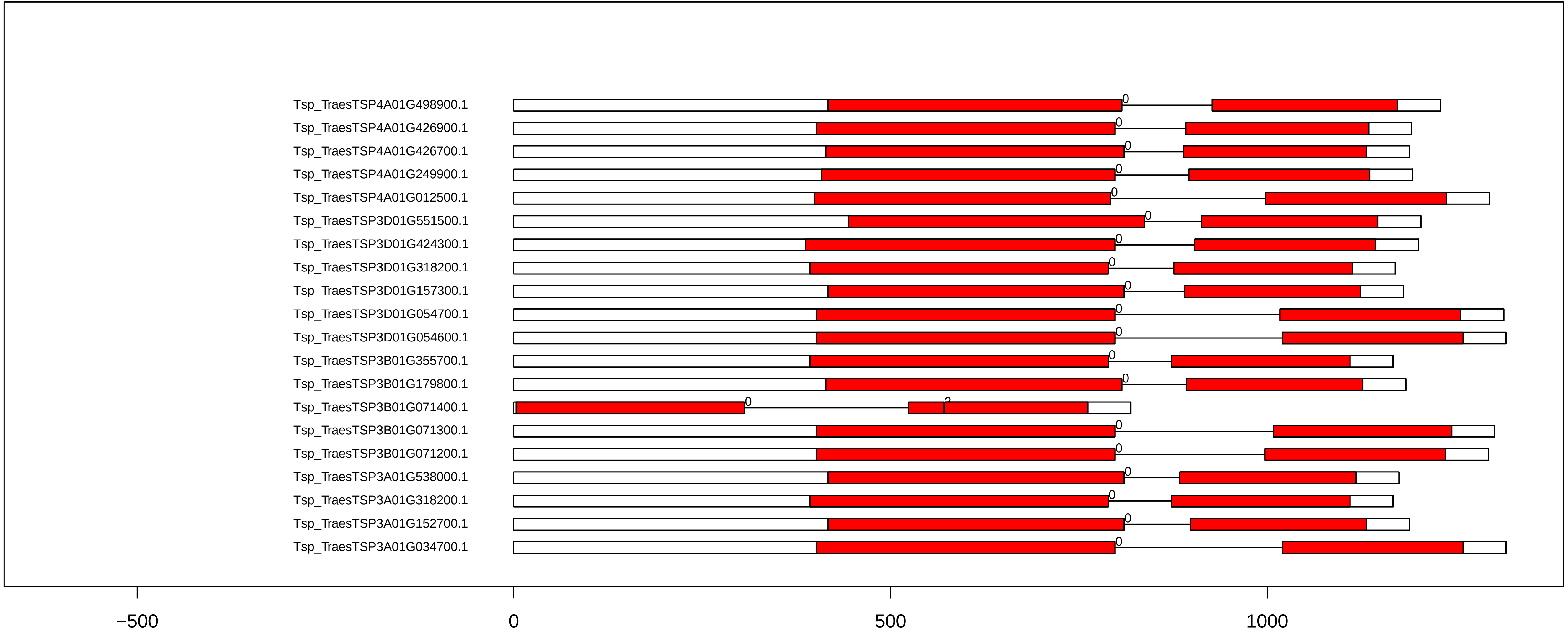

T.sp ASMT II subfamily exon−intron and kinase domain diagram (part 5)

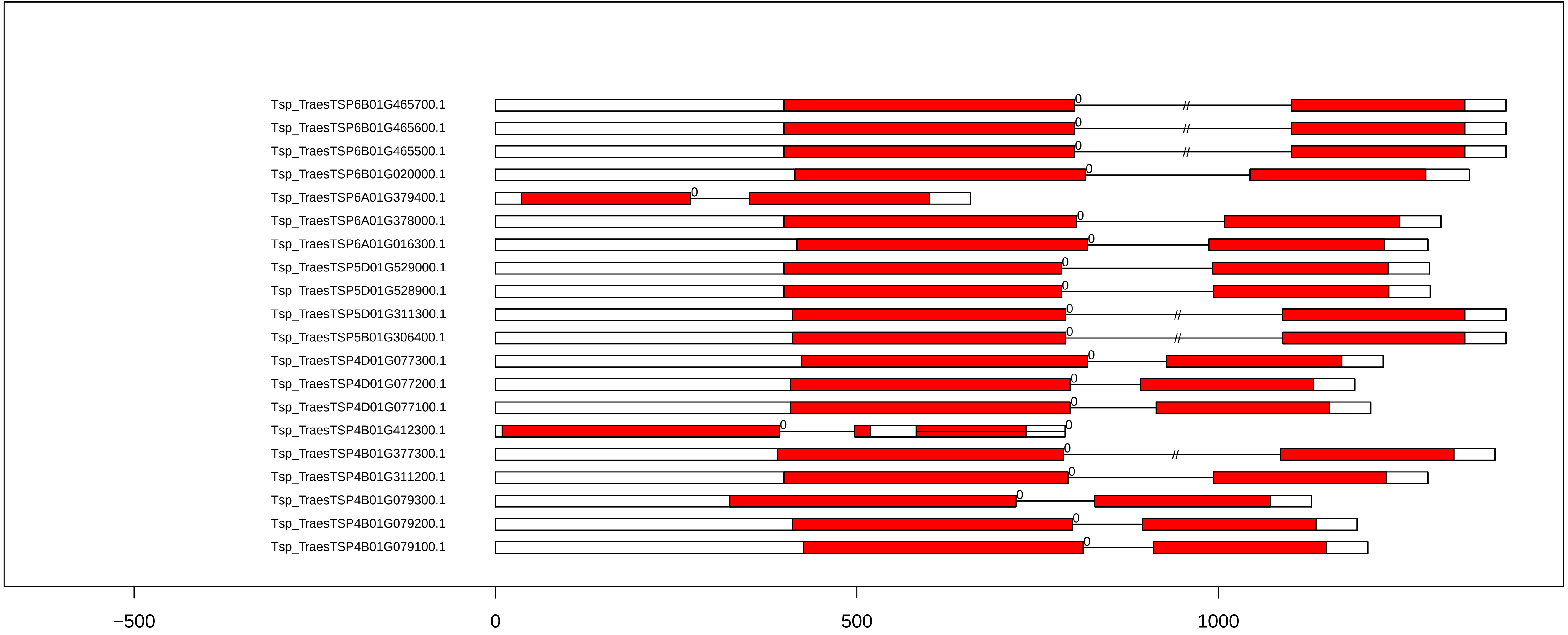

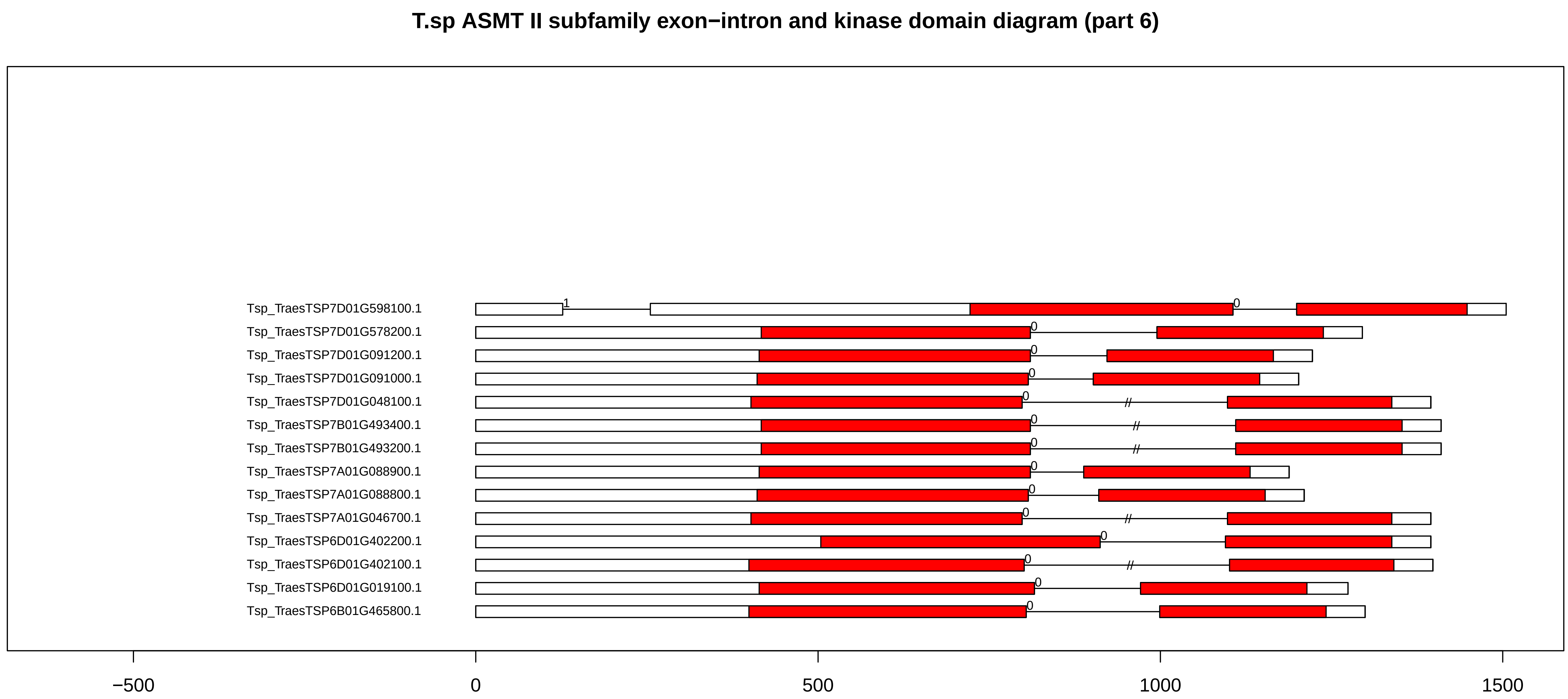
T.sp ASMT II subfamily exon−intron and kinase domain diagram (part 6)
Tsp_TraesTSP7D01G598100.1
Tsp_TraesTSP7D01G578200.1
Tsp_TraesTSP7D01G091200.1
Tsp_TraesTSP7D01G091000.1
Tsp_TraesTSP7D01G048100.1
Tsp_TraesTSP7B01G493400.1
Tsp_TraesTSP7B01G493200.1
Tsp_TraesTSP7A01G088900.1
Tsp_TraesTSP7A01G088800.1
Tsp_TraesTSP7A01G046700.1
Tsp_TraesTSP6D01G402200.1
Tsp_TraesTSP6D01G402100.1
Tsp_TraesTSP6D01G019100.1
Tsp_TraesTSP6B01G465800.1
−500
0
500
1000
1500

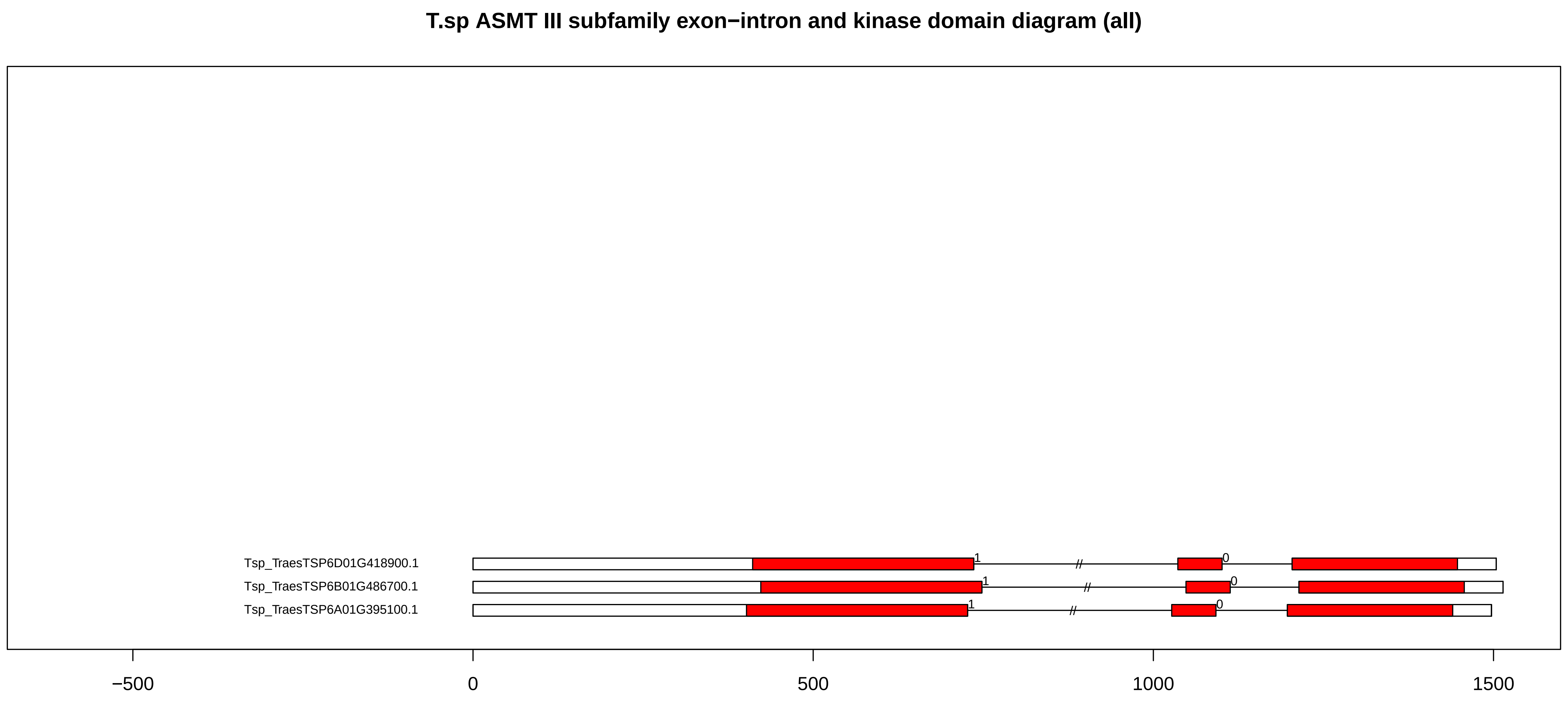
T.sp ASMT III subfamily exon−intron and kinase domain diagram (all)
Tsp_TraesTSP6D01G418900.1
Tsp_TraesTSP6B01G486700.1
Tsp_TraesTSP6A01G395100.1
1
0
1
0
1
0
−500
0
500
1000
1500

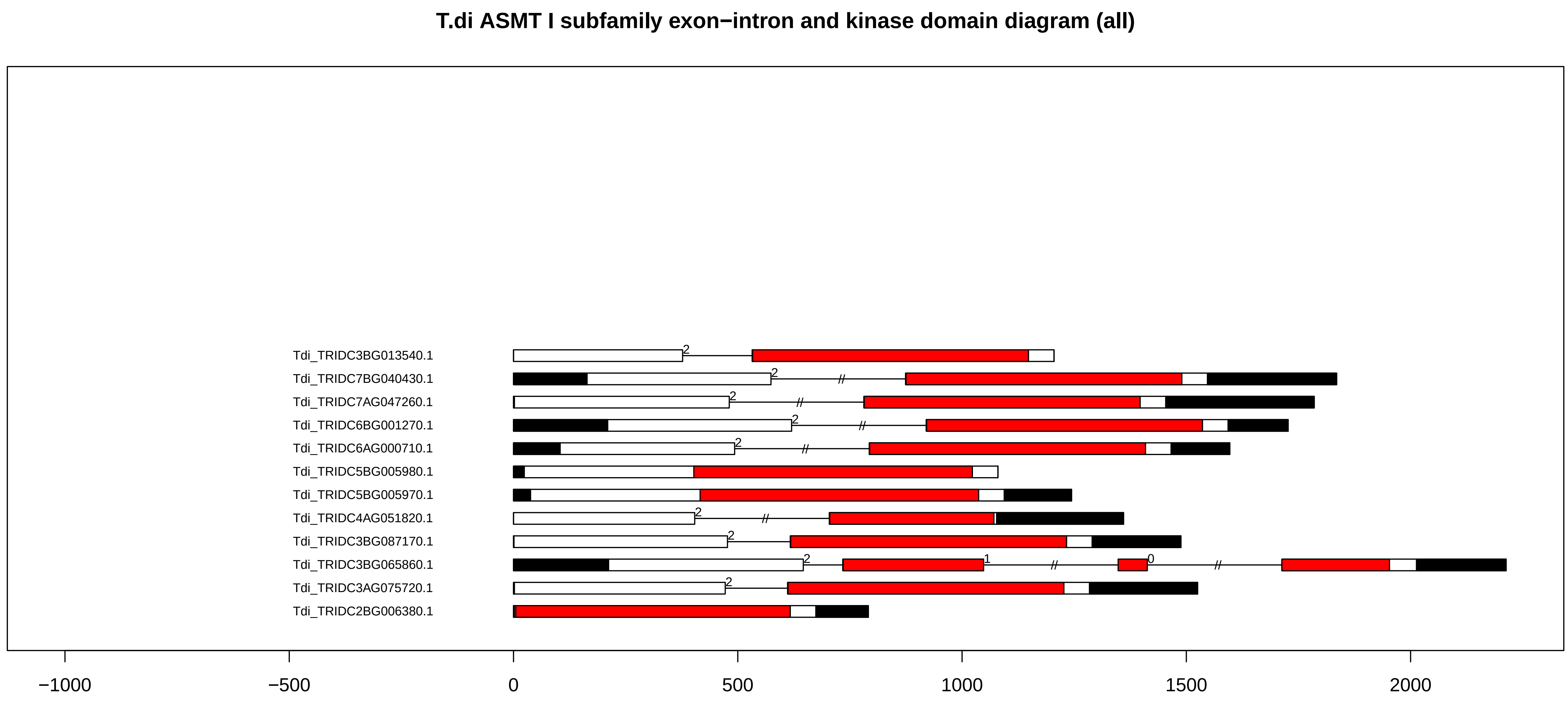

T.di ASMT I subfamily exon−intron and kinase domain diagram (all)
Tdi_TRIDC3BG013540.1
Tdi_TRIDC7BG040430.1
Tdi_TRIDC7AG047260.1
Tdi_TRIDC6BG001270.1
Tdi_TRIDC6AG000710.1
Tdi_TRIDC5BG005980.1
Tdi_TRIDC5BG005970.1
Tdi_TRIDC4AG051820.1
Tdi_TRIDC3BG087170.1
Tdi_TRIDC3BG065860.1
Tdi_TRIDC3AG075720.1
Tdi_TRIDC2BG006380.1
−1000
−500
0
500
1000
1500
2000

T.di ASMT II subfamily exon−intron and kinase domain diagram (part 1)

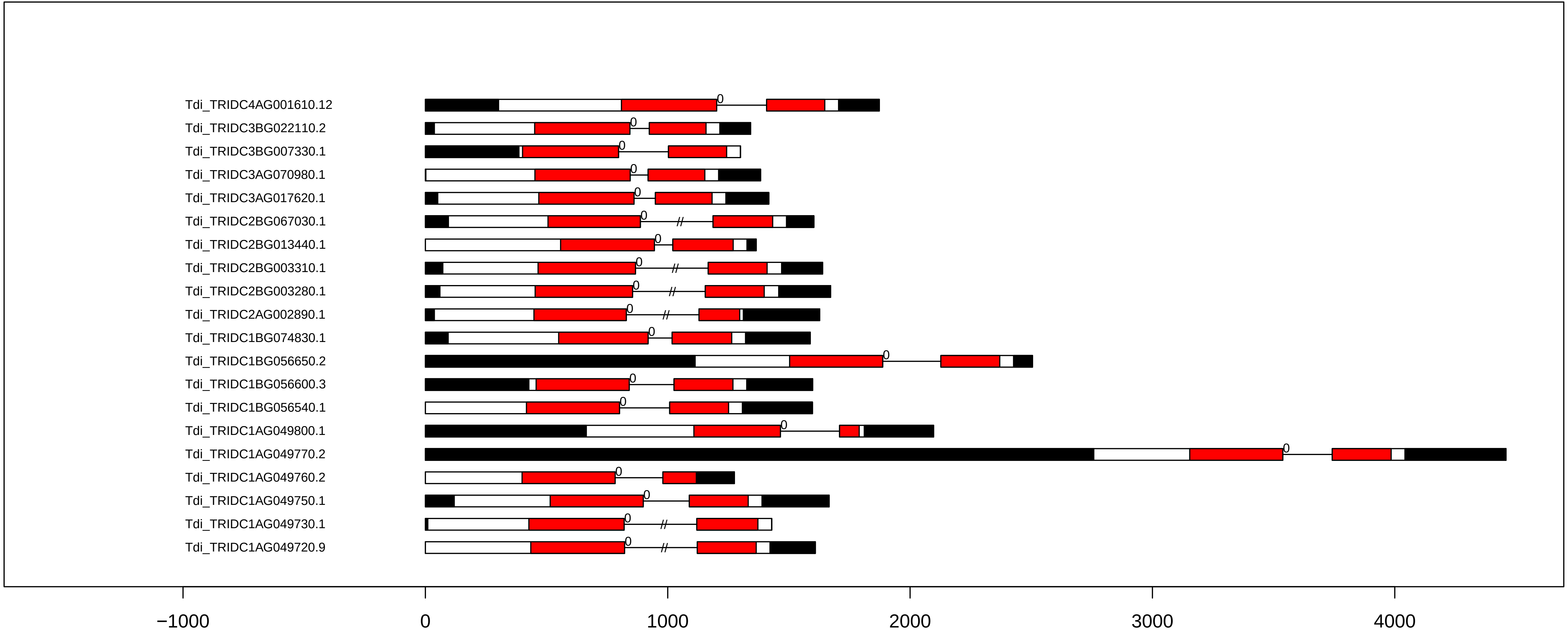

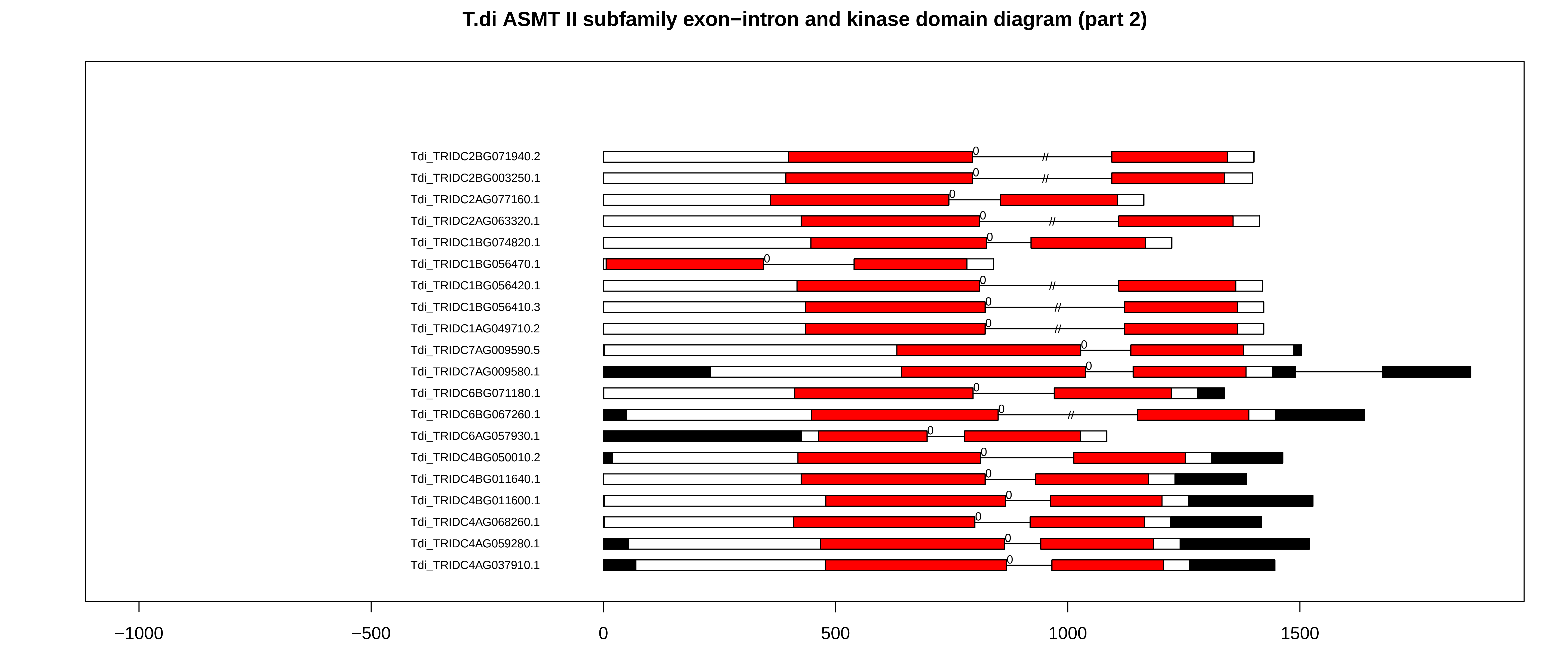

T.di ASMT II subfamily exon−intron and kinase domain diagram (part 2)
Tdi_TRIDC2BG071940.2
Tdi_TRIDC2BG003250.1
Tdi_TRIDC2AG077160.1
Tdi_TRIDC2AG063320.1
Tdi_TRIDC1BG074820.1
Tdi_TRIDC1BG056470.1
Tdi_TRIDC1BG056420.1
Tdi_TRIDC1BG056410.3
Tdi_TRIDC1AG049710.2
Tdi_TRIDC7AG009590.5
Tdi_TRIDC7AG009580.1
Tdi_TRIDC6BG071180.1
Tdi_TRIDC6BG067260.1
Tdi_TRIDC6AG057930.1
Tdi_TRIDC4BG050010.2
Tdi_TRIDC4BG011640.1
Tdi_TRIDC4BG011600.1
Tdi_TRIDC4AG068260.1
Tdi_TRIDC4AG059280.1
Tdi_TRIDC4AG037910.1
−1000
−500
0
500
1000
1500

T.di ASMT II subfamily exon−intron and kinase domain diagram (part 3)

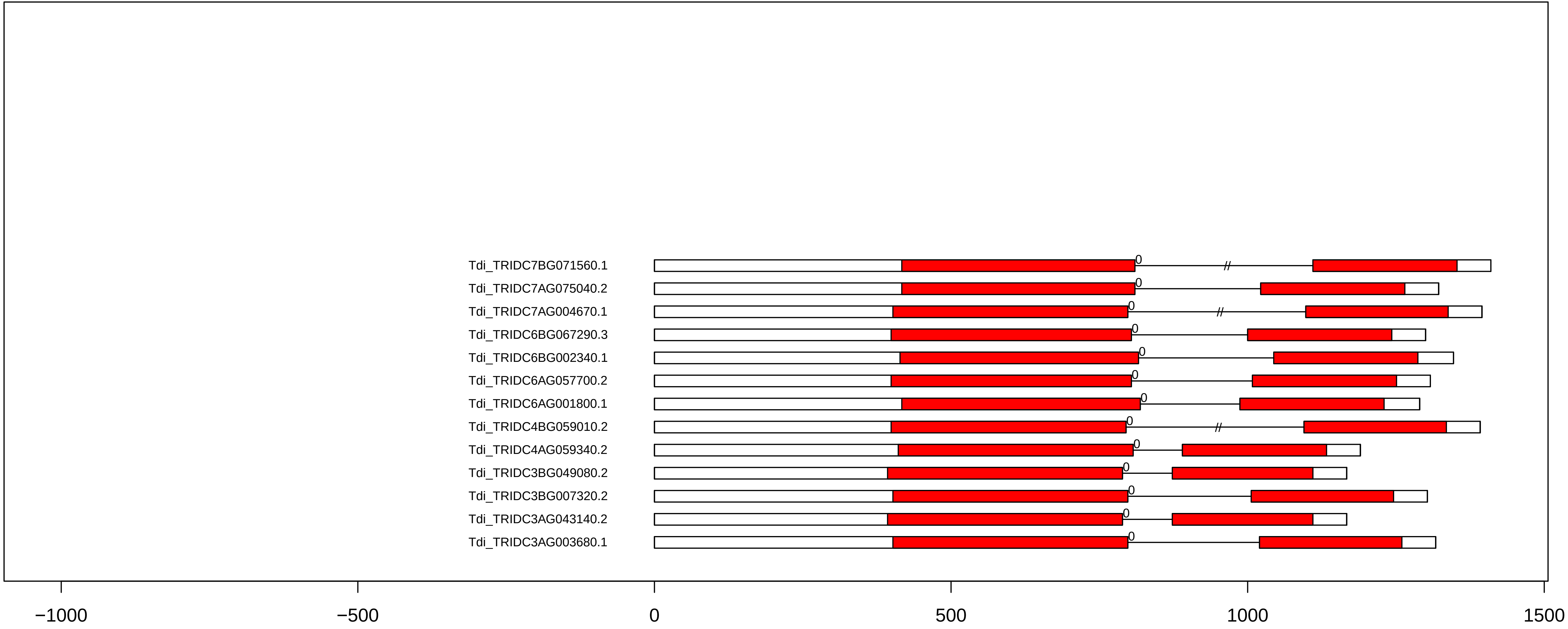

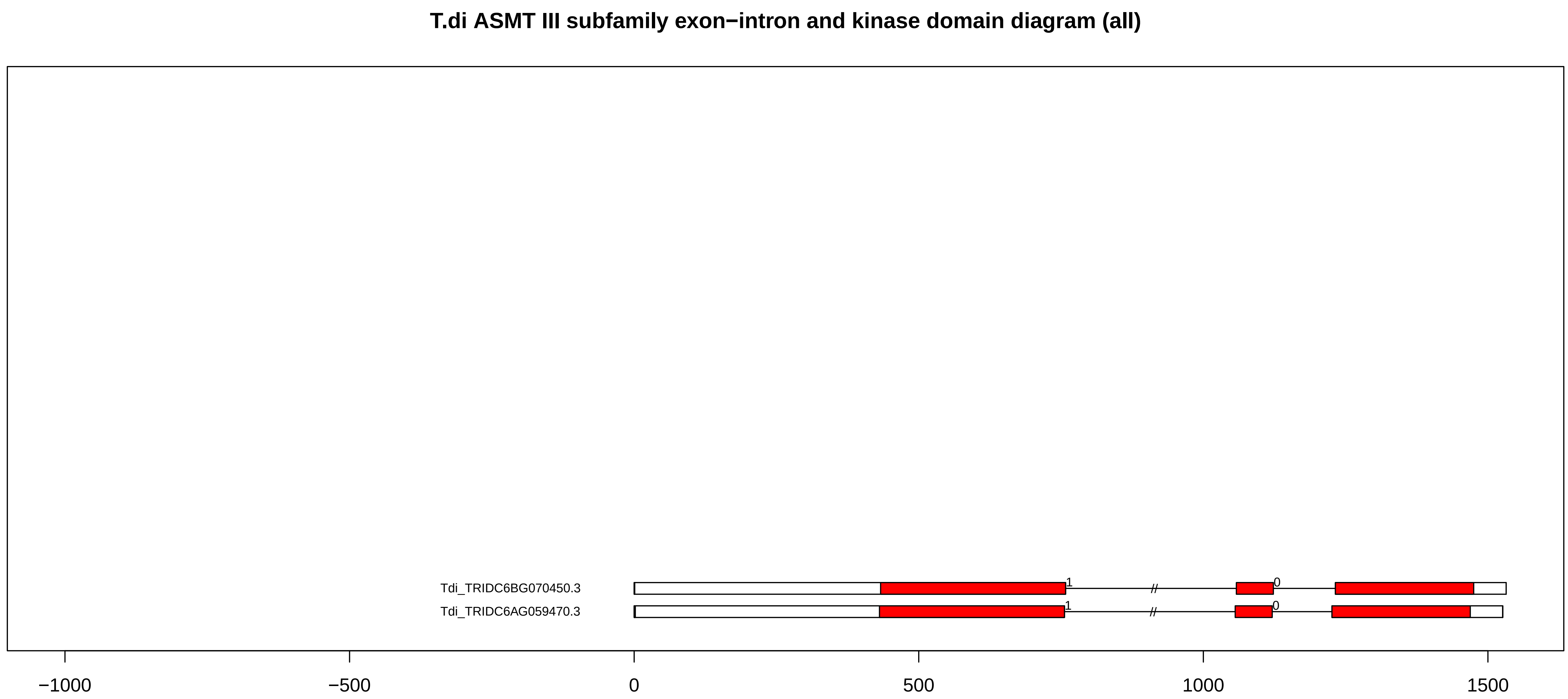

T.di ASMT III subfamily exon−intron and kinase domain diagram (all)
Tdi_TRIDC6BG070450.3
Tdi_TRIDC6AG059470.3
1
0
1
0
−1000
−500
0
500
1000
1500

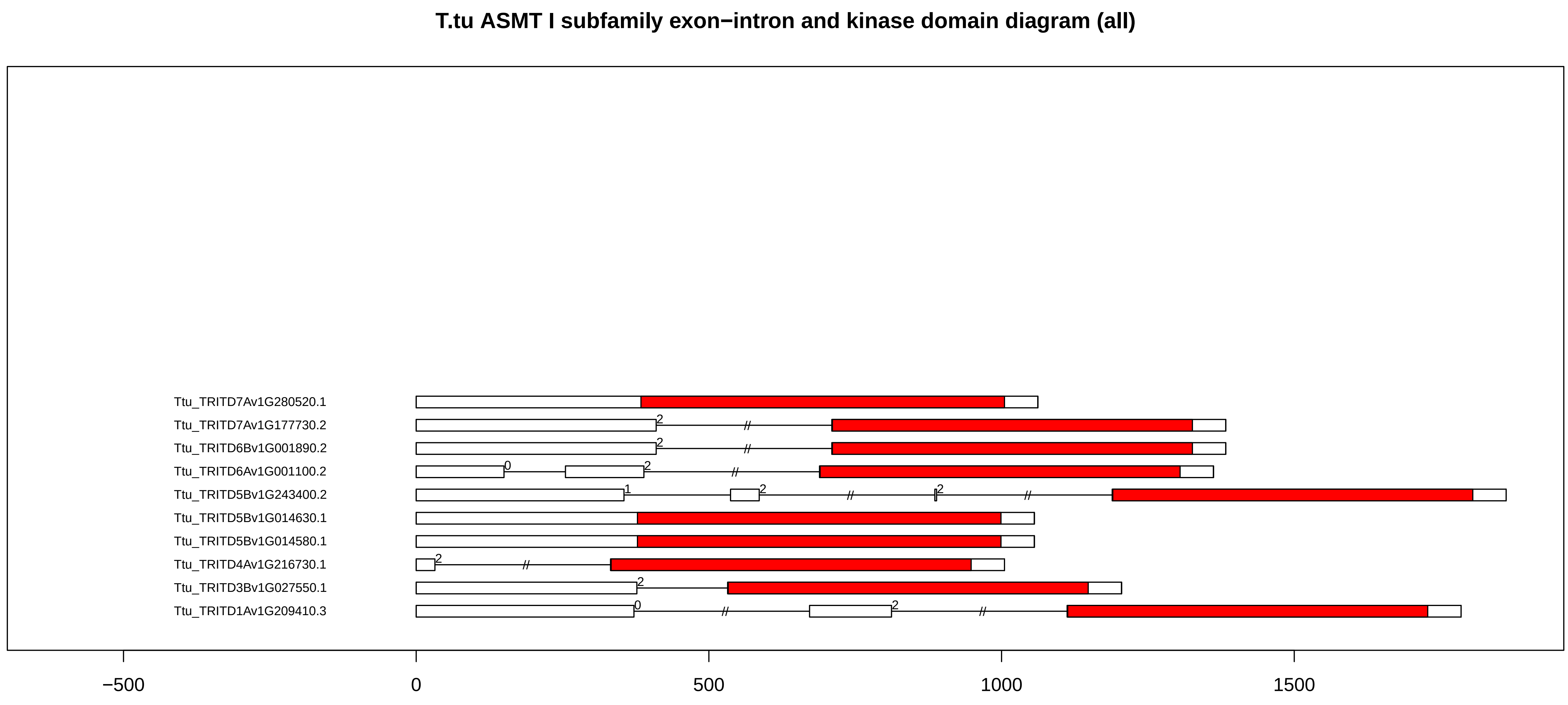
T.tu ASMT I subfamily exon−intron and kinase domain diagram (all)
Ttu_TRITD7Av1G280520.1
Ttu_TRITD7Av1G177730.2
Ttu_TRITD6Bv1G001890.2
Ttu_TRITD6Av1G001100.2
Ttu_TRITD5Bv1G243400.2
Ttu_TRITD5Bv1G014630.1
Ttu_TRITD5Bv1G014580.1
Ttu_TRITD4Av1G216730.1
Ttu_TRITD3Bv1G027550.1
Ttu_TRITD1Av1G209410.3
−500
0
500
1000
1500

T.tu ASMT II subfamily exon−intron and kinase domain diagram (part 1)

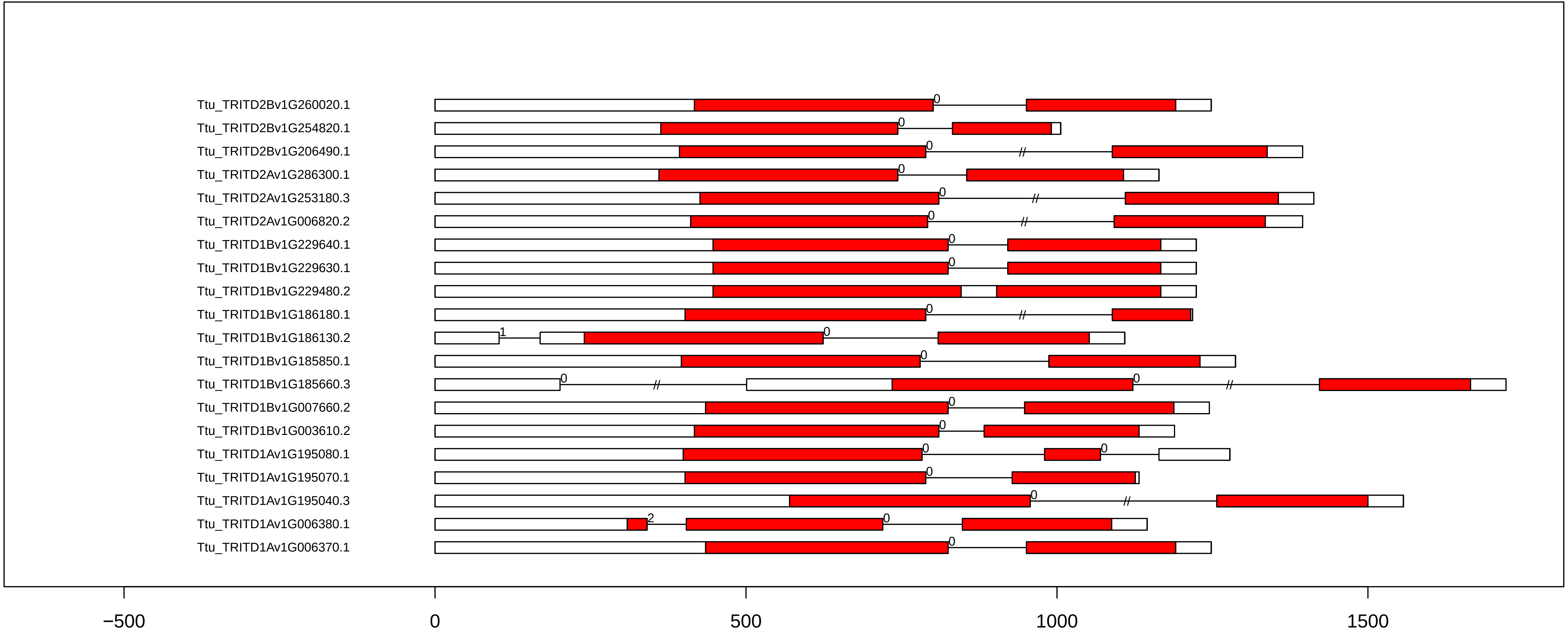

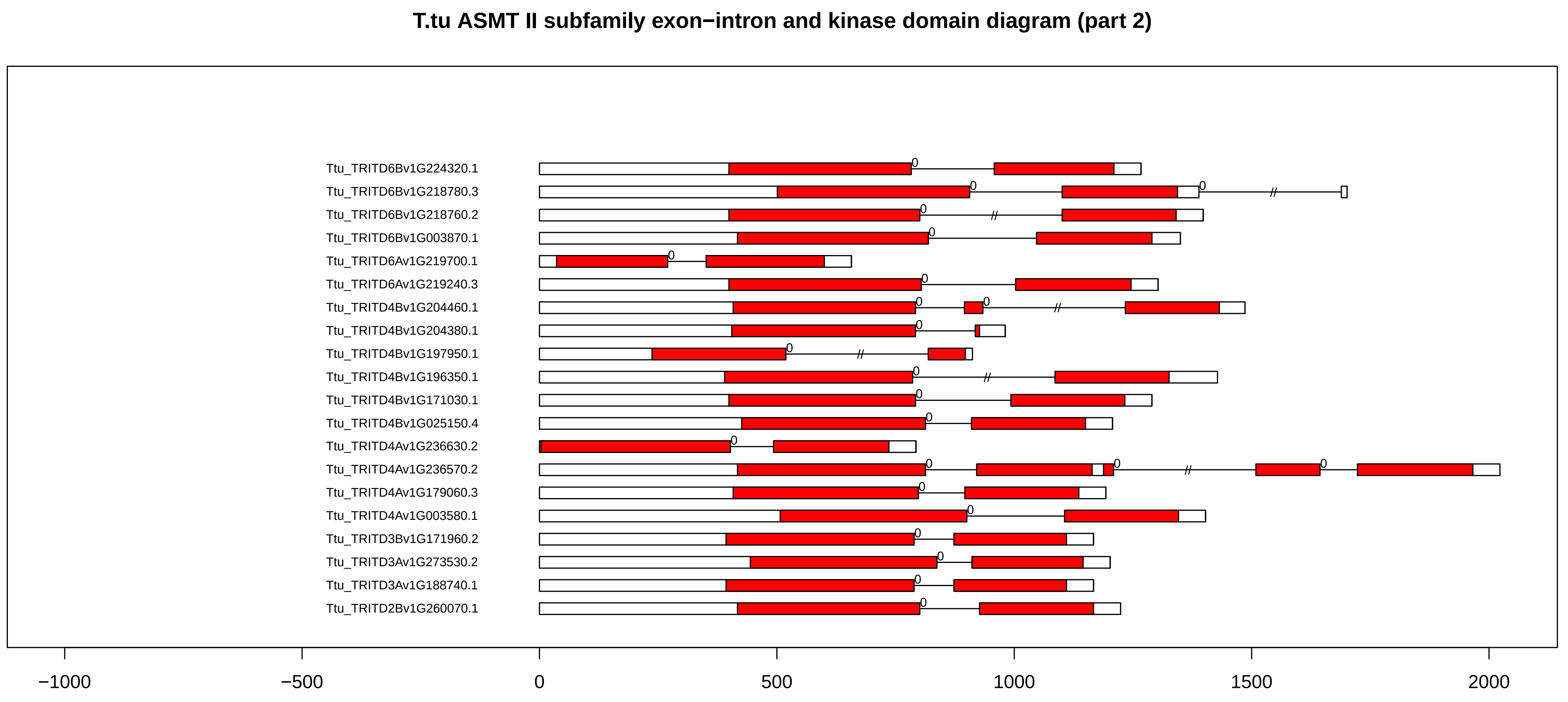
T.tu ASMT II subfamily exon−intron and kinase domain diagram (part 2)
Ttu_TRITD6Bv1G224320.1
Ttu_TRITD6Bv1G218780.3
Ttu_TRITD6Bv1G218760.2
Ttu_TRITD6Bv1G003870.1
Ttu_TRITD6Av1G219700.1
Ttu_TRITD6Av1G219240.3
Ttu_TRITD4Bv1G204460.1
Ttu_TRITD4Bv1G204380.1
Ttu_TRITD4Bv1G197950.1
Ttu_TRITD4Bv1G196350.1
Ttu_TRITD4Bv1G171030.1
Ttu_TRITD4Bv1G025150.4
Ttu_TRITD4Av1G236630.2
Ttu_TRITD4Av1G236570.2
Ttu_TRITD4Av1G179060.3
Ttu_TRITD4Av1G003580.1
Ttu_TRITD3Bv1G171960.2
Ttu_TRITD3Av1G273530.2
Ttu_TRITD3Av1G188740.1
Ttu_TRITD2Bv1G260070.1
−1000
−500
0
500
1000
1500
2000

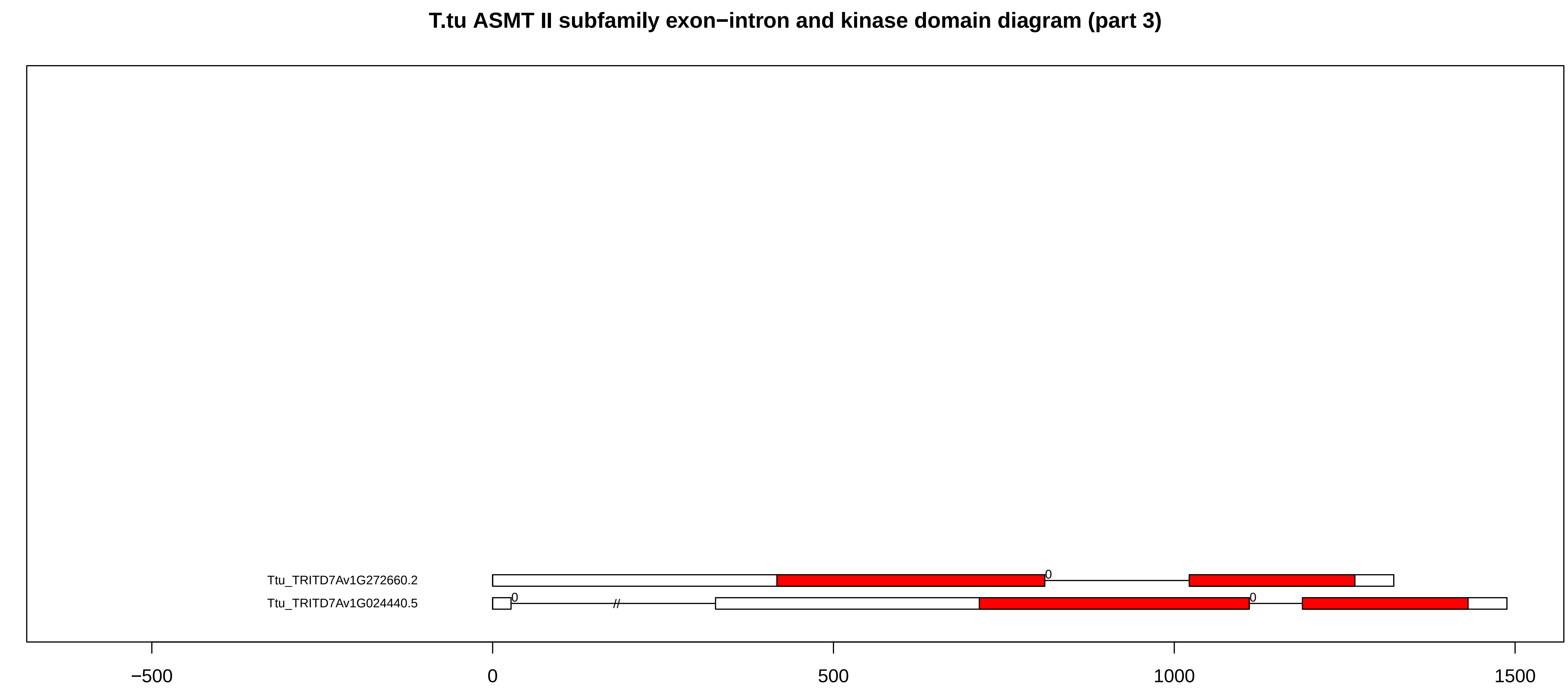
T.tu ASMT II subfamily exon−intron and kinase domain diagram (part 3)
Ttu_TRITD7Av1G272660.2
Ttu_TRITD7Av1G024440.5
0
0
0
−500
0
500
1000
1500

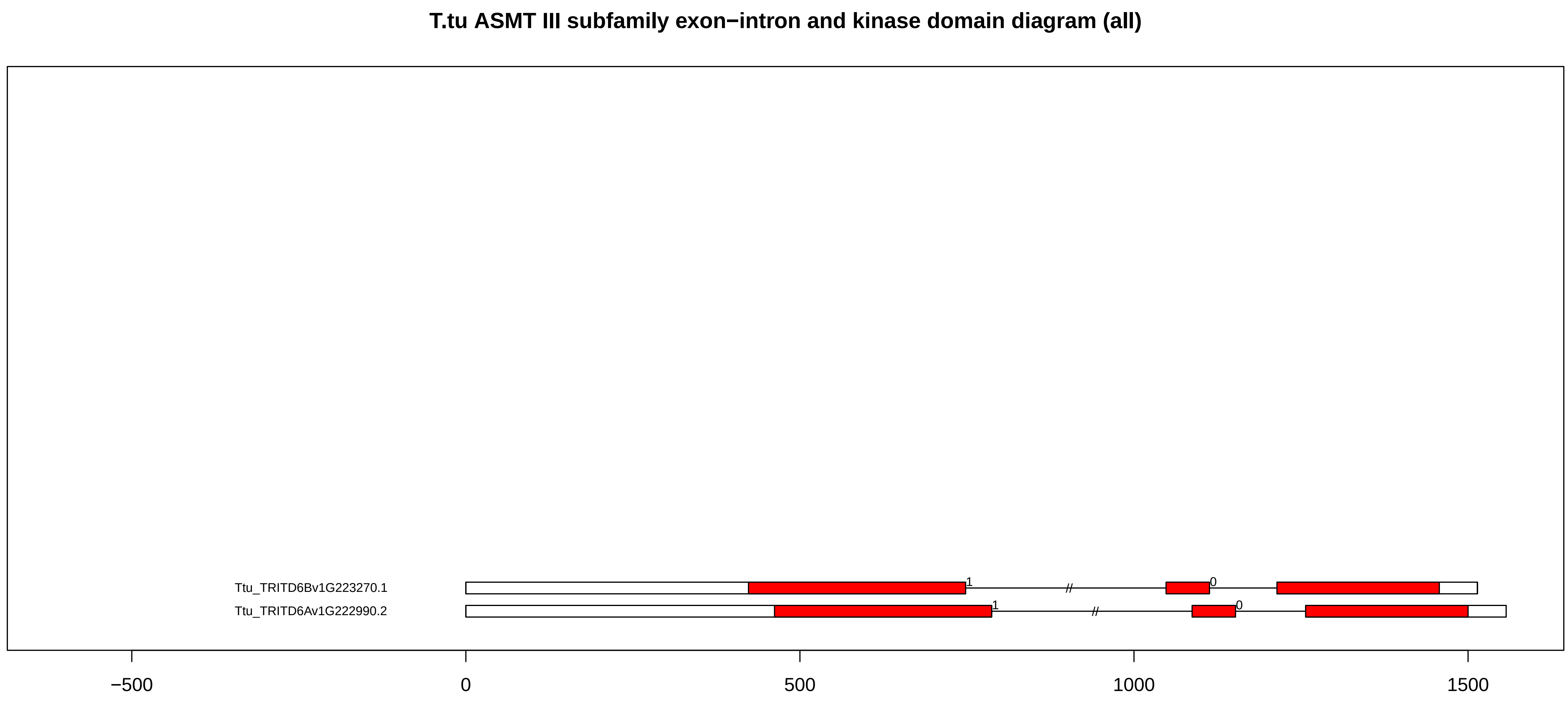
T.tu ASMT III subfamily exon−intron and kinase domain diagram (all)
Ttu_TRITD6Bv1G223270.1
Ttu_TRITD6Av1G222990.2
1
0
1
0
−500
0
500
1000
1500

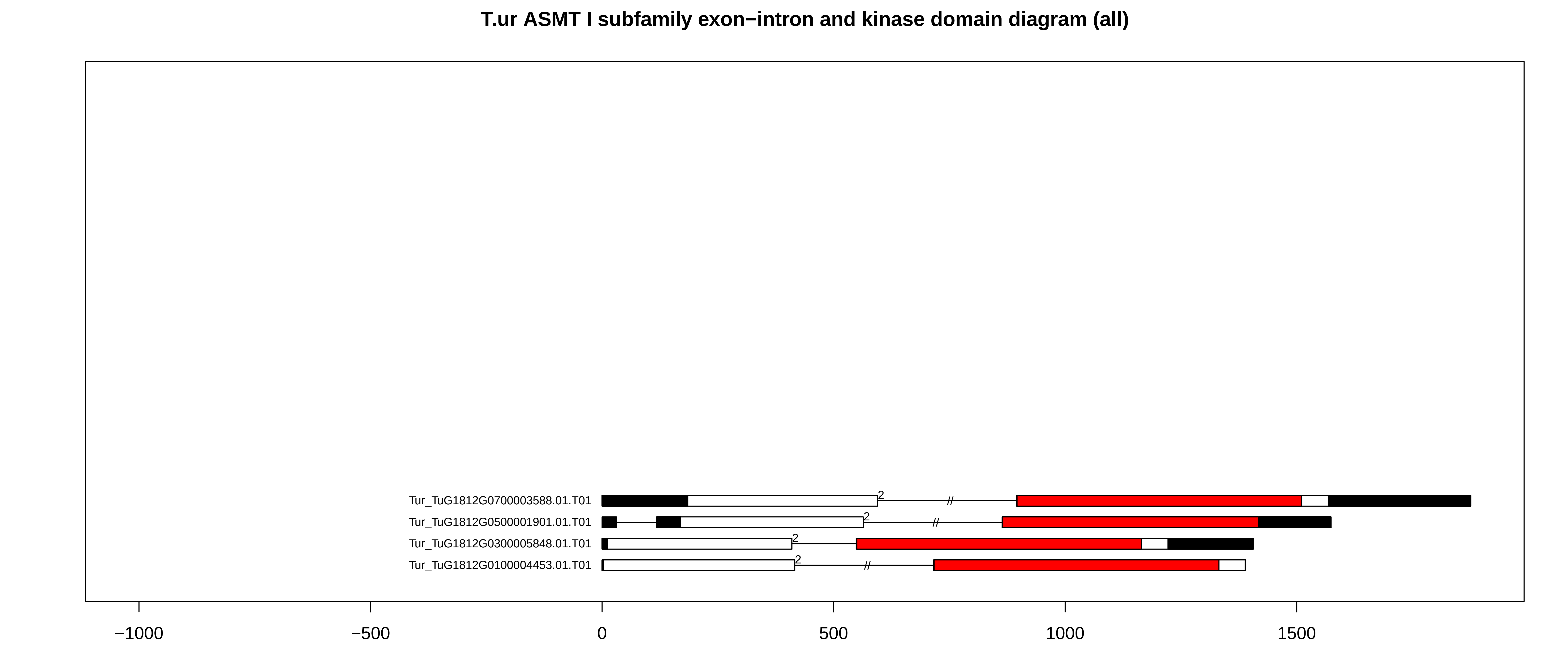

T.ur ASMT I subfamily exon−intron and kinase domain diagram (all)
Tur_TuG1812G0700003588.01.T01
Tur_TuG1812G0500001901.01.T01
Tur_TuG1812G0300005848.01.T01
Tur_TuG1812G0100004453.01.T01
2
2
2
2
−1000
−500
0
500
1000
1500

T.ur ASMT II subfamily exon−intron and kinase domain diagram (part 1)

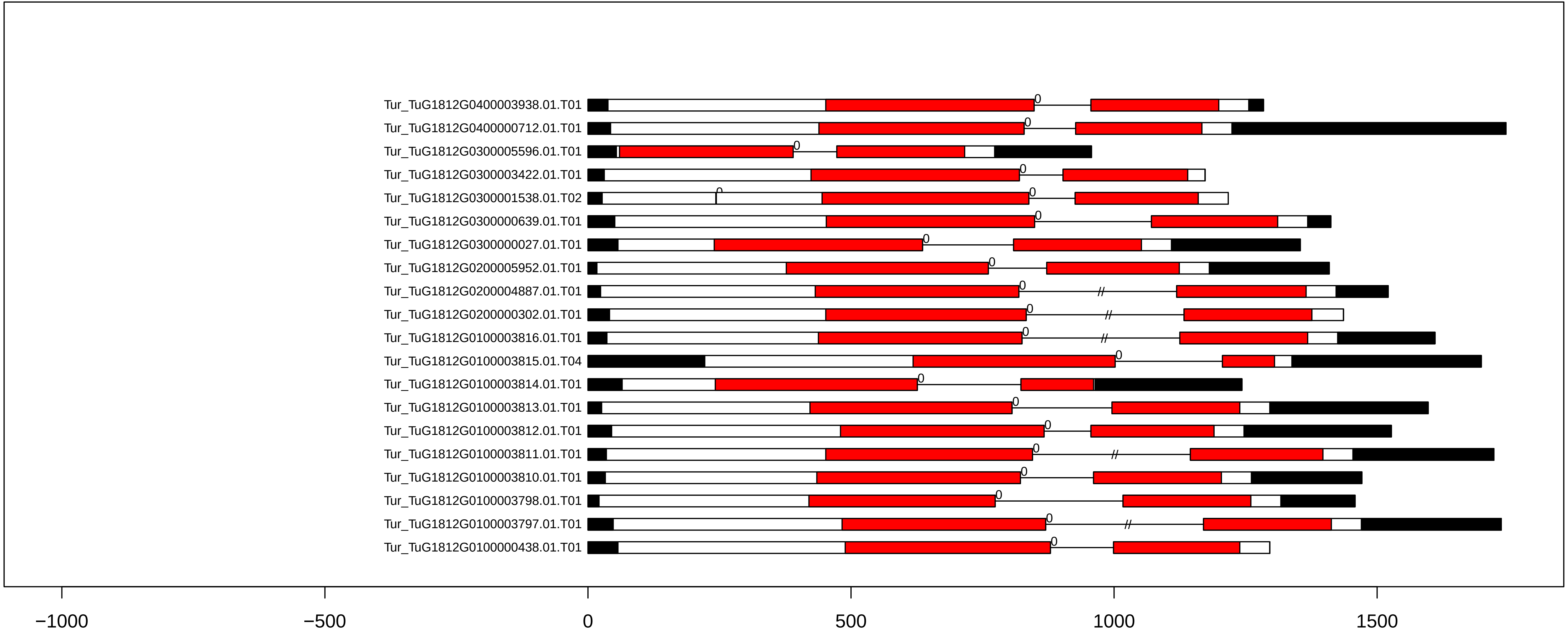

T.ur ASMT II subfamily exon−intron and kinase domain diagram (part 2)

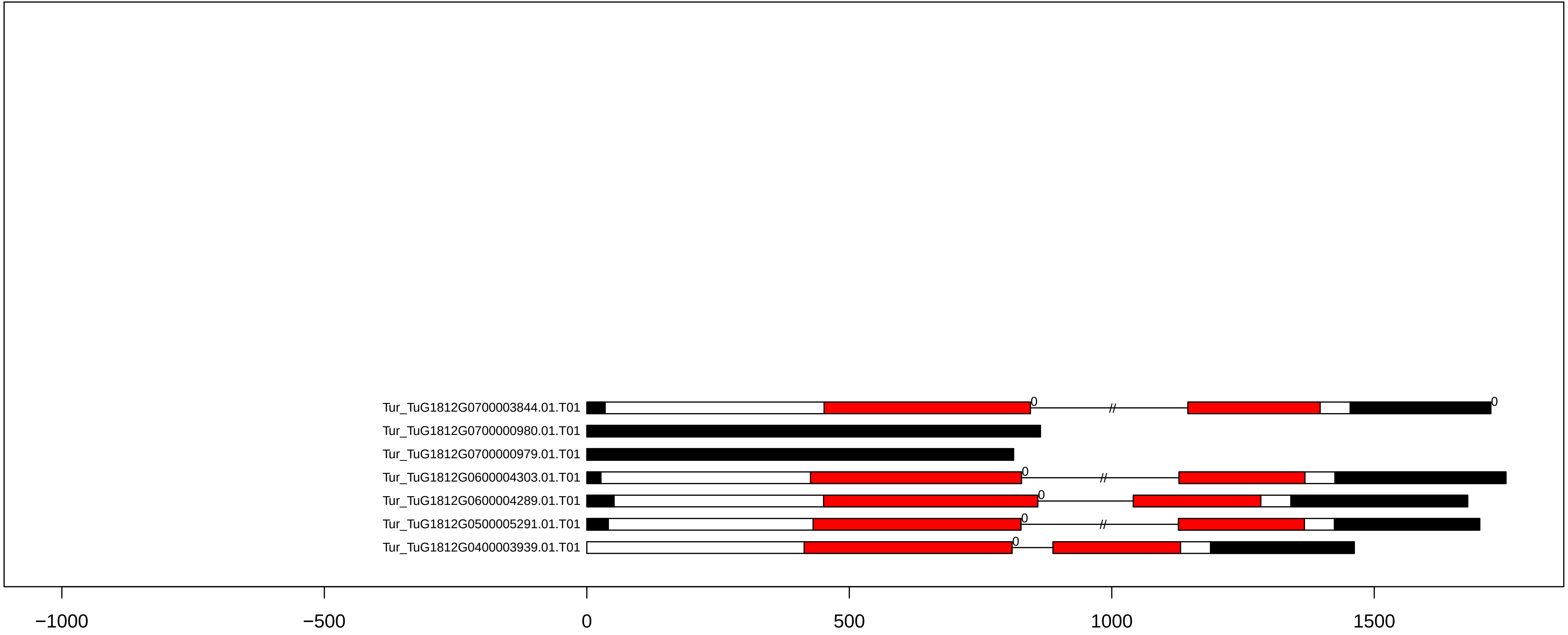

Ae.ta ASMT I subfamily exon−intron and kinase domain diagram (all)

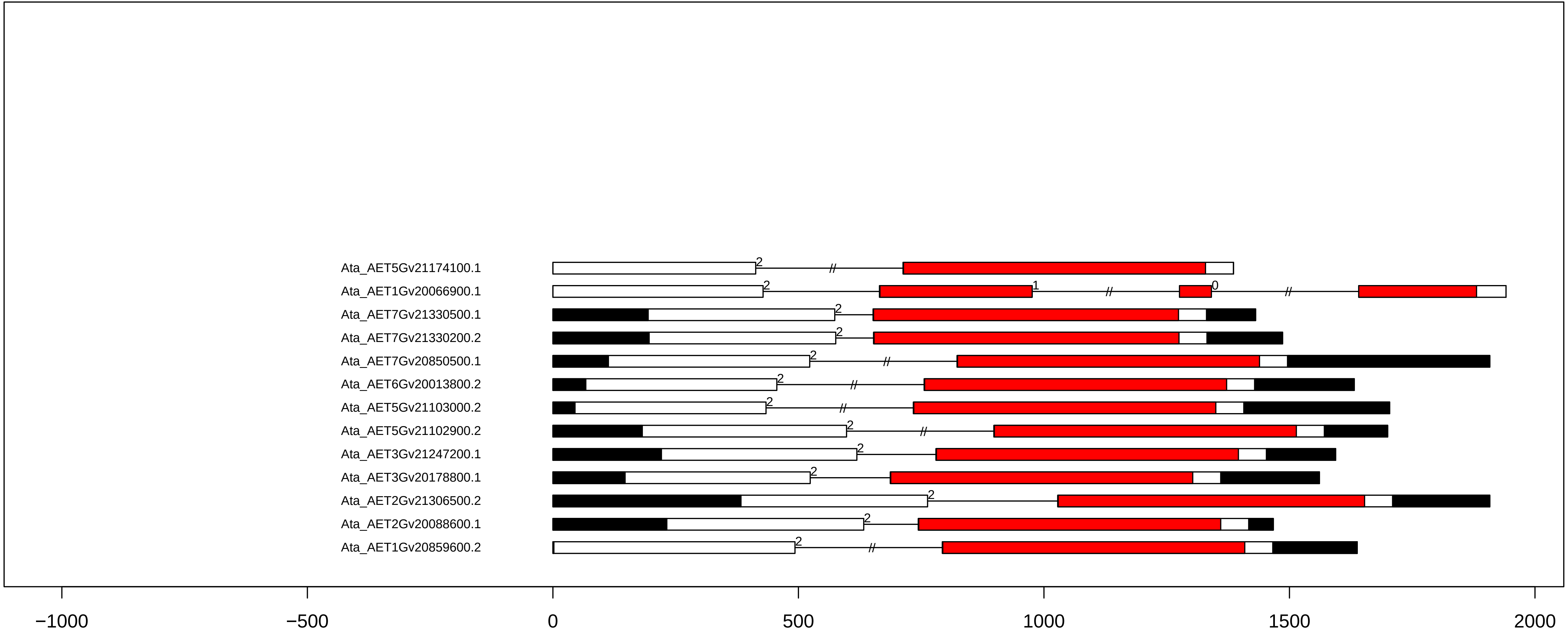

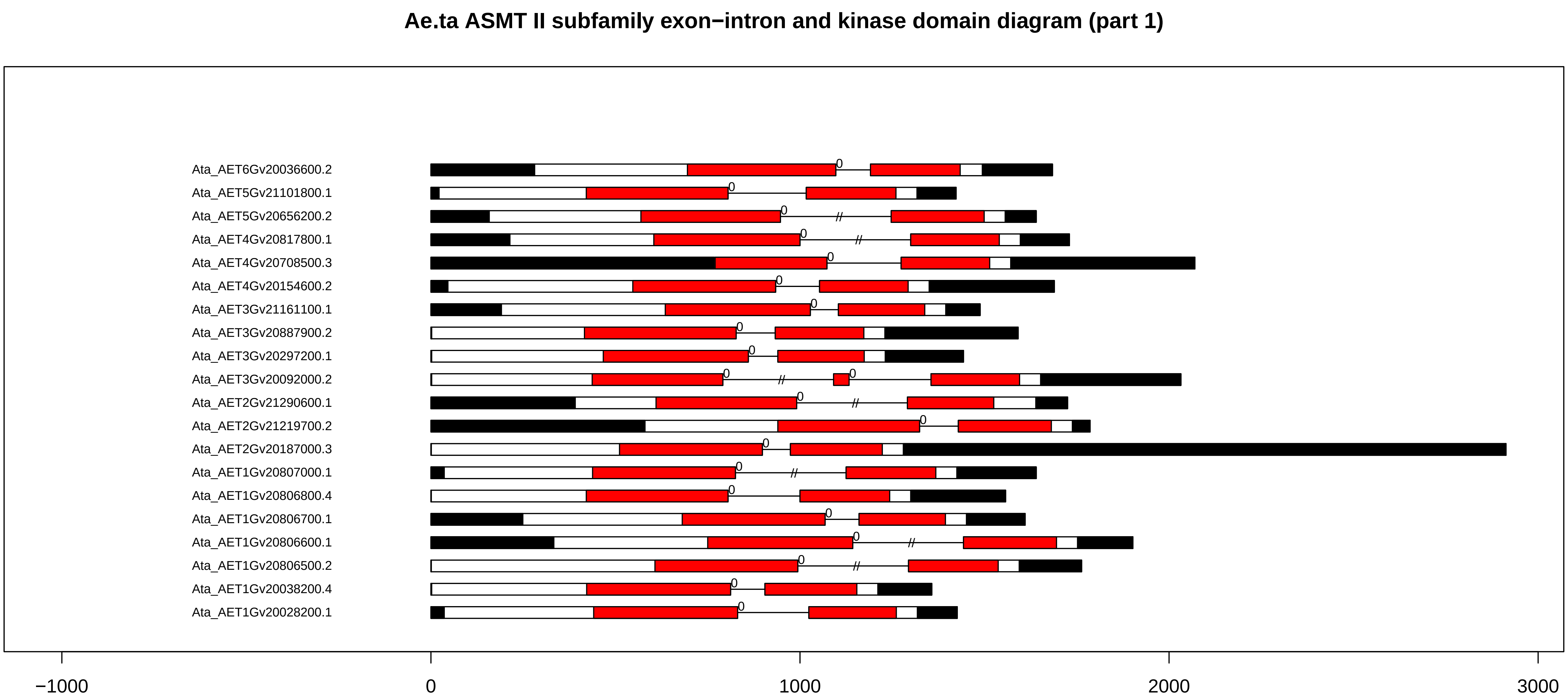

Ae.ta ASMT II subfamily exon−intron and kinase domain diagram (part 1)
Ata_AET6Gv20036600.2
Ata_AET5Gv21101800.1
Ata_AET5Gv20656200.2
Ata_AET4Gv20817800.1
Ata_AET4Gv20708500.3
Ata_AET4Gv20154600.2
Ata_AET3Gv21161100.1
Ata_AET3Gv20887900.2
Ata_AET3Gv20297200.1
Ata_AET3Gv20092000.2
Ata_AET2Gv21290600.1
Ata_AET2Gv21219700.2
Ata_AET2Gv20187000.3
Ata_AET1Gv20807000.1
Ata_AET1Gv20806800.4
Ata_AET1Gv20806700.1
Ata_AET1Gv20806600.1
Ata_AET1Gv20806500.2
Ata_AET1Gv20038200.4
Ata_AET1Gv20028200.1
−1000
0
1000
2000
3000

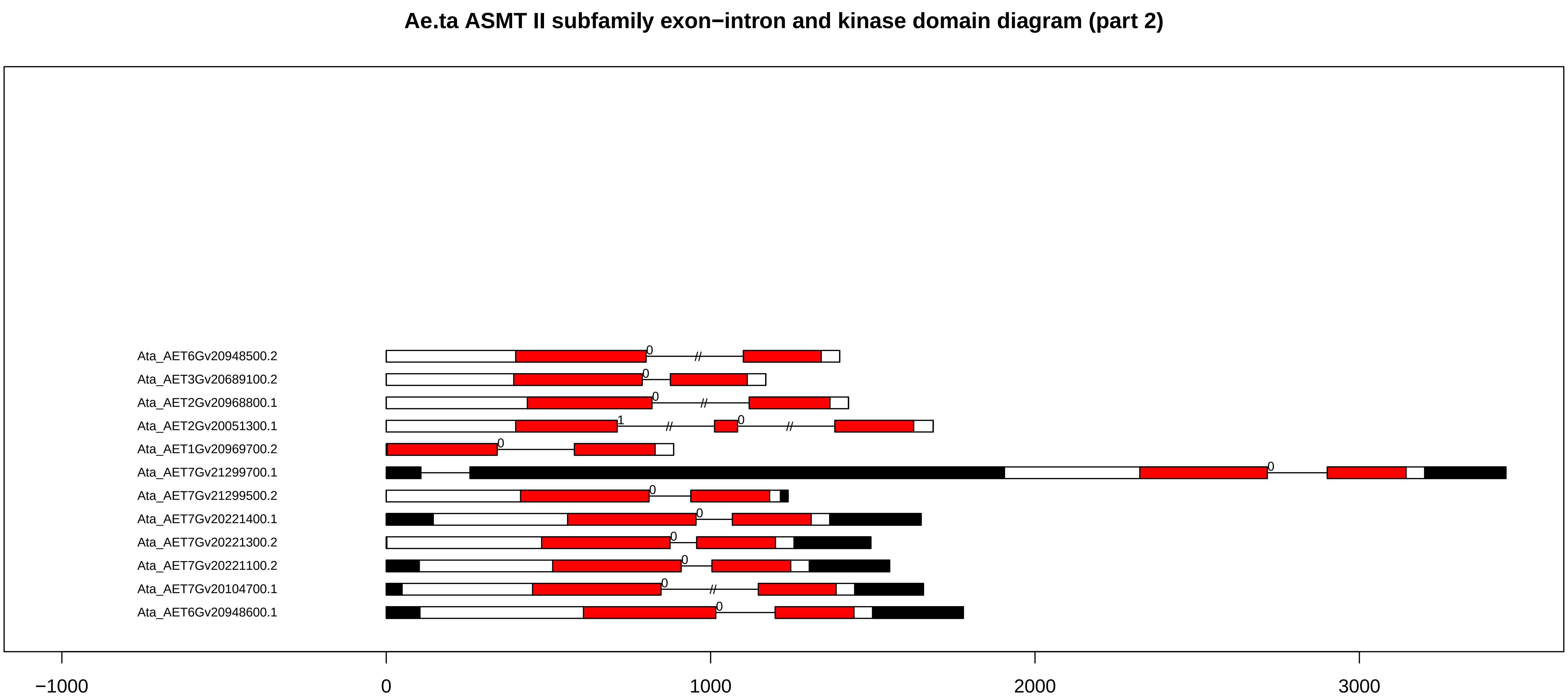
Ae.ta ASMT II subfamily exon−intron and kinase domain diagram (part 2)
Ata_AET6Gv20948500.2
Ata_AET3Gv20689100.2
Ata_AET2Gv20968800.1
Ata_AET2Gv20051300.1
Ata_AET1Gv20969700.2
Ata_AET7Gv21299700.1
Ata_AET7Gv21299500.2
Ata_AET7Gv20221400.1
Ata_AET7Gv20221300.2
Ata_AET7Gv20221100.2
Ata_AET7Gv20104700.1
Ata_AET6Gv20948600.1
−1000
0
1000
2000
3000

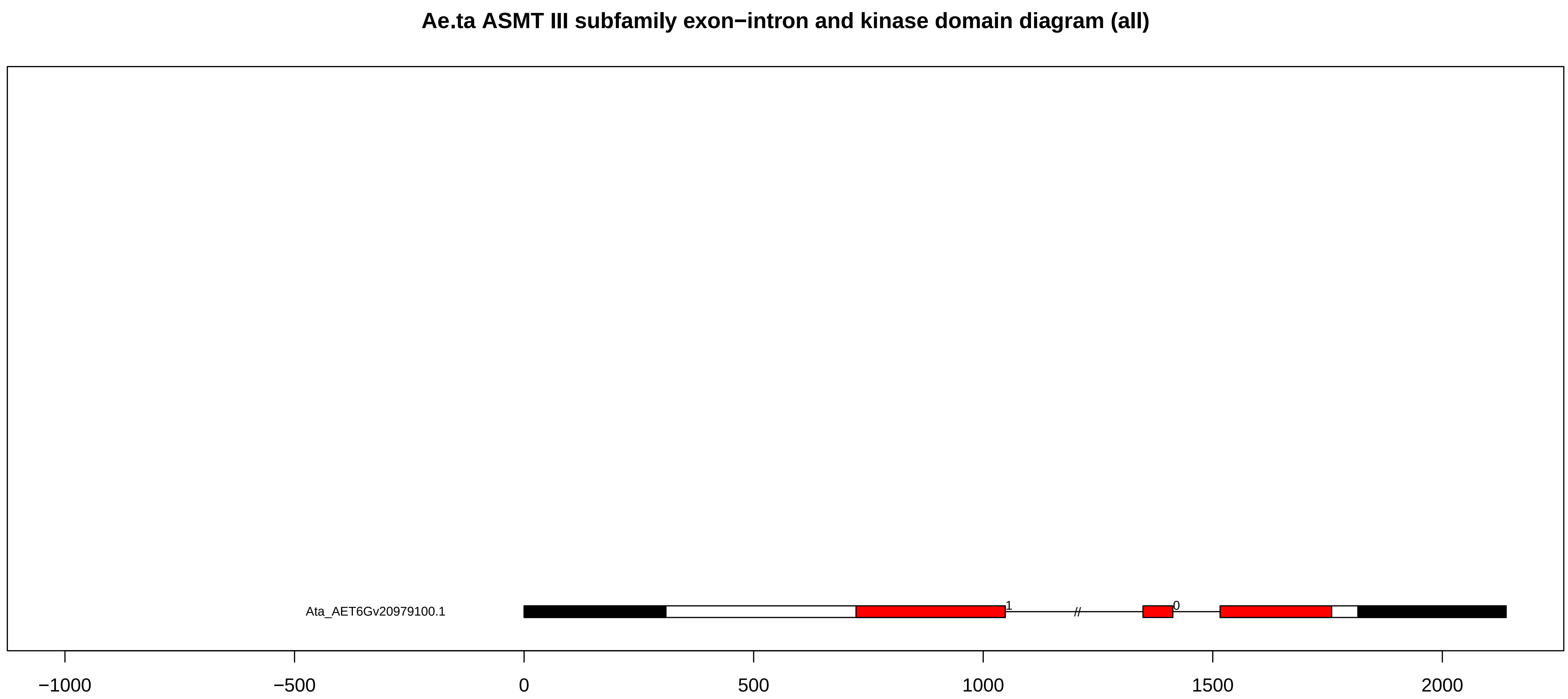
Ae.ta ASMT III subfamily exon−intron and kinase domain diagram (all)
Ata_AET6Gv20979100.1
1
0
−1000
−500
0
500
1000
1500
2000

B.di ASMT I subfamily exon−intron and kinase domain diagram (all)

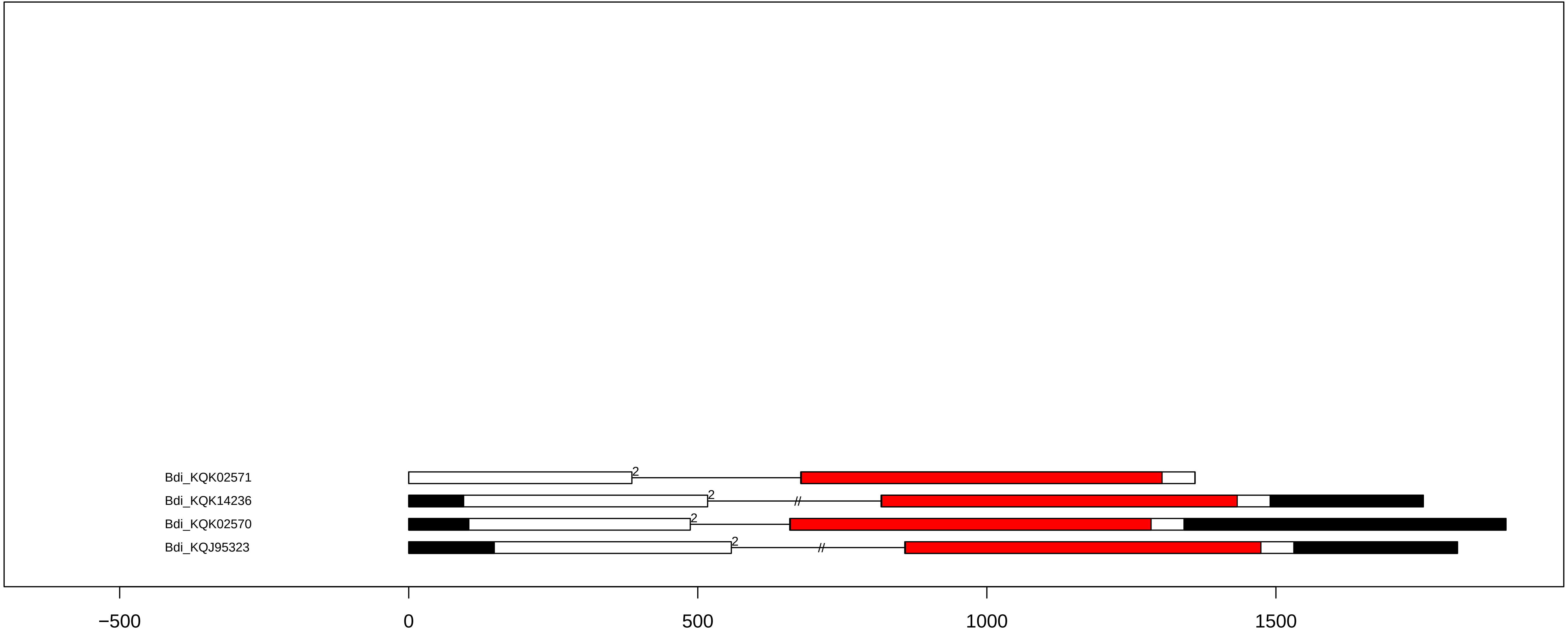

B.di ASMT II subfamily exon−intron and kinase domain diagram (all)

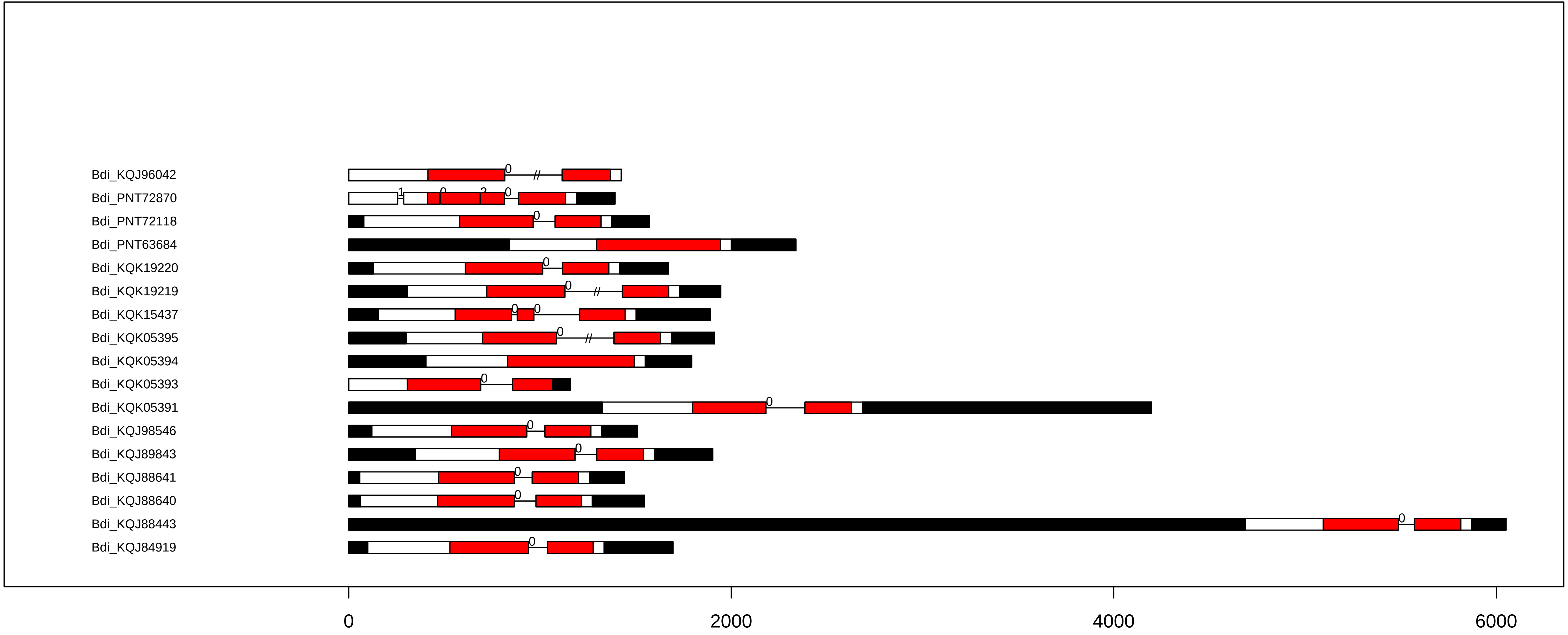

B.di ASMT III subfamily exon−intron and kinase domain diagram (all)

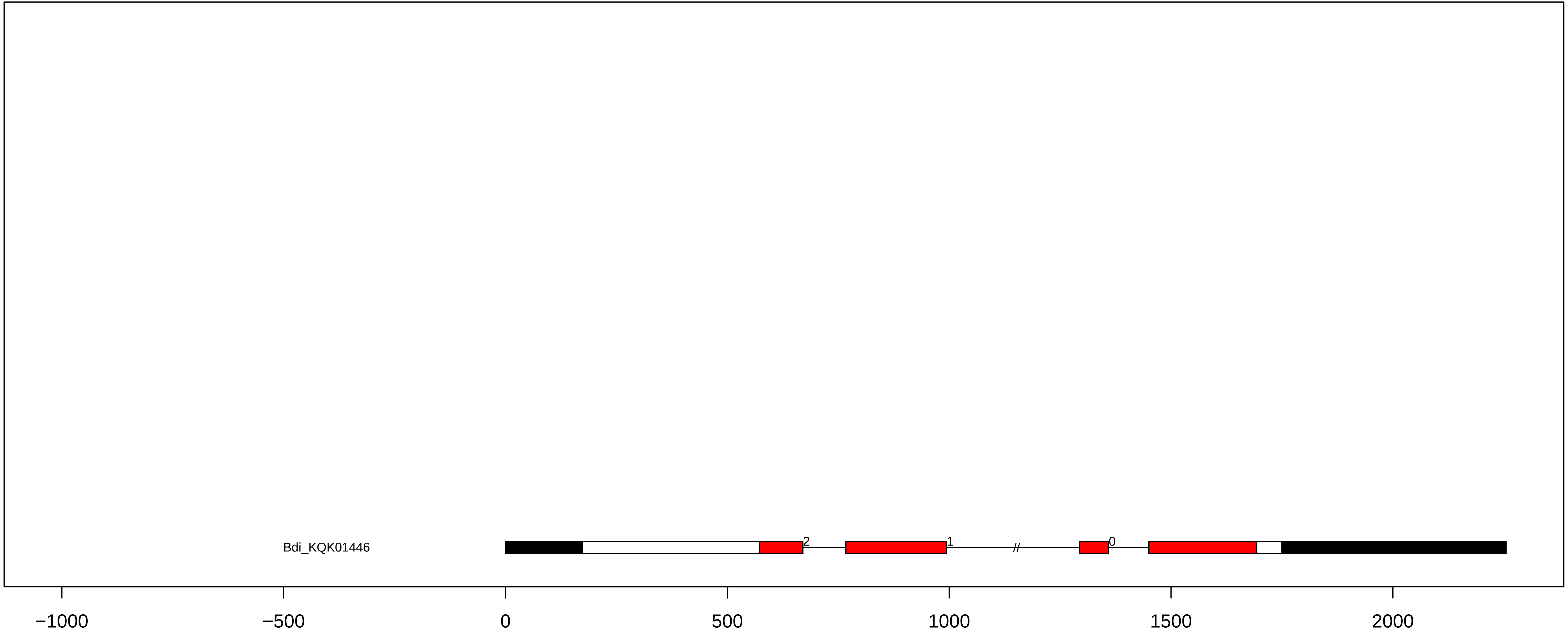

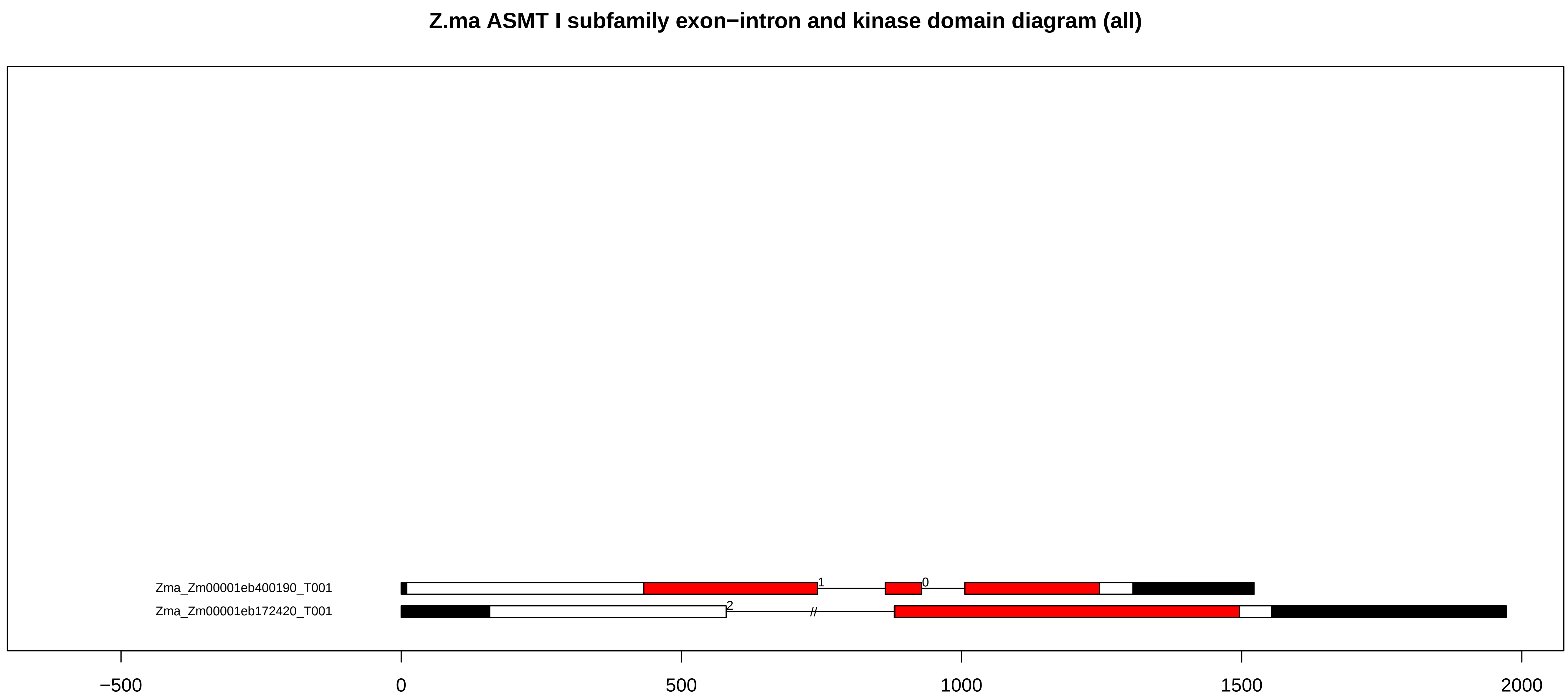

Z.ma ASMT I subfamily exon−intron and kinase domain diagram (all)
Zma_Zm00001eb400190_T001
Zma_Zm00001eb172420_T001
1
0
2
−500
0
500
1000
1500
2000

Z.ma ASMT II subfamily exon−intron and kinase domain diagram (part 1)

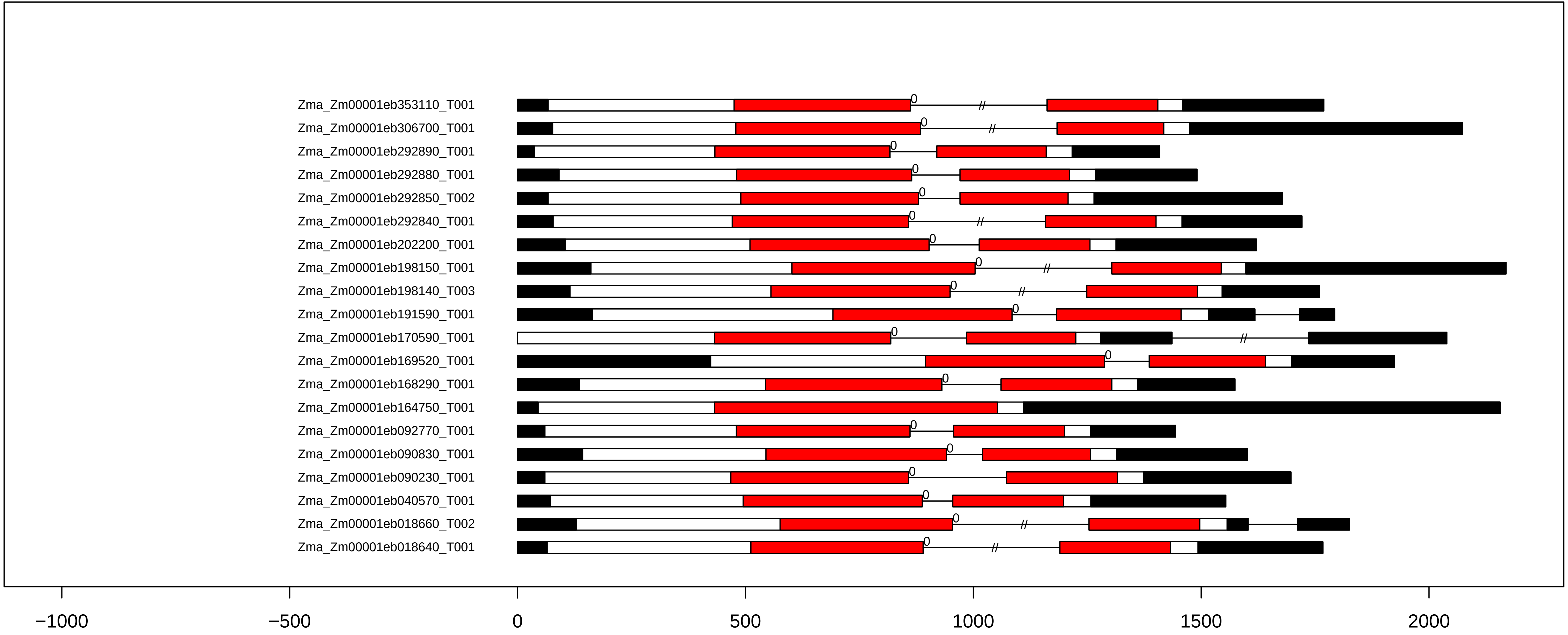

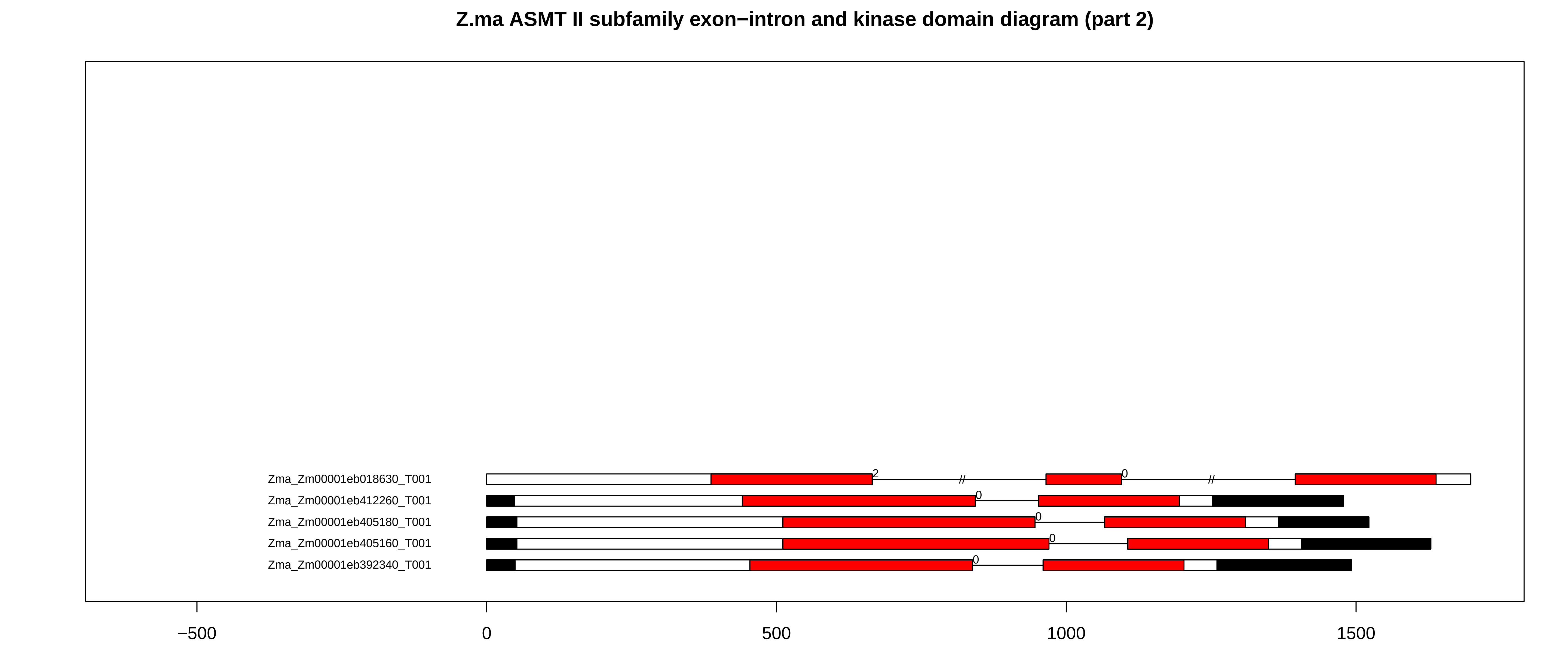
Z.ma ASMT II subfamily exon−intron and kinase domain diagram (part 2)
Zma_Zm00001eb018630_T001
Zma_Zm00001eb412260_T001
Zma_Zm00001eb405180_T001
Zma_Zm00001eb405160_T001
Zma_Zm00001eb392340_T001
2
0
0
0
0
0
−500
0
500
1000
1500

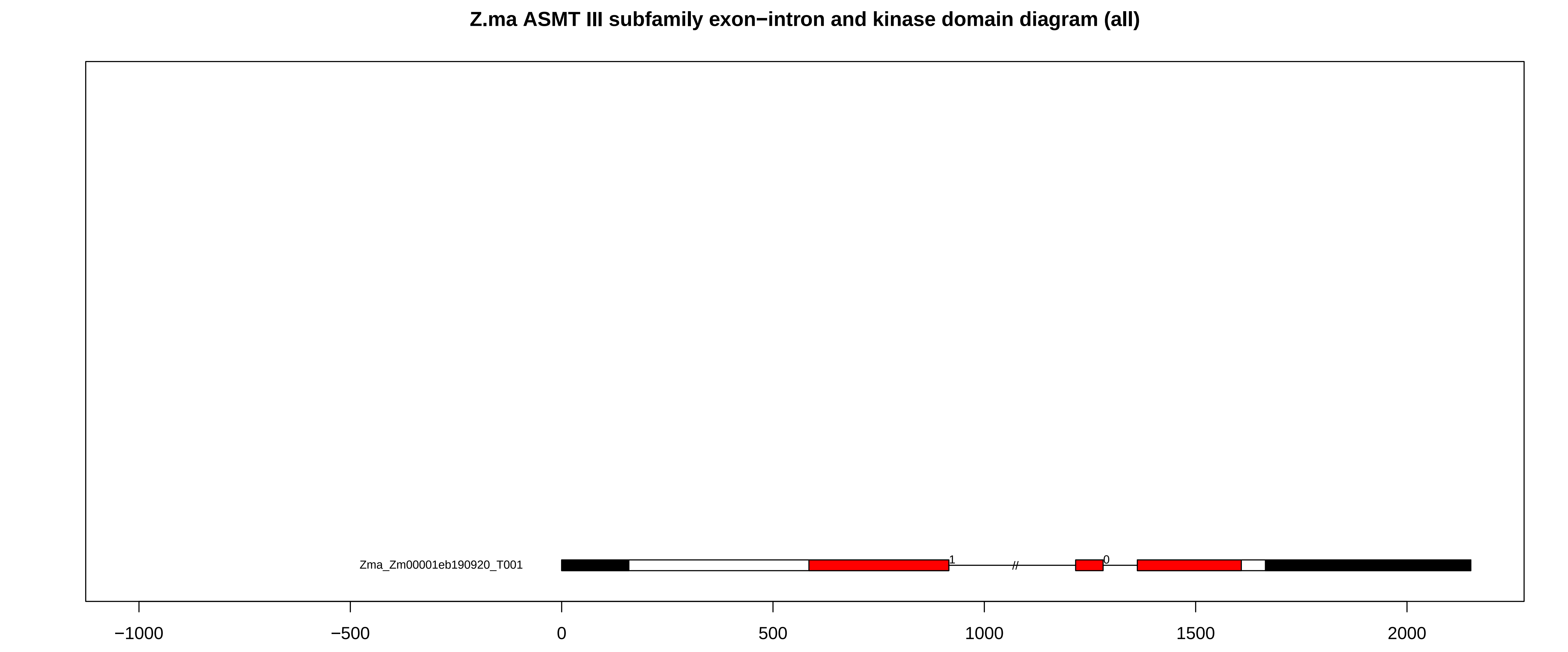
Z.ma ASMT III subfamily exon−intron and kinase domain diagram (all)
Zma_Zm00001eb190920_T001
1
0
−1000
−500
0
500
1000
1500
2000

O.sa ASMT I subfamily exon−intron and kinase domain diagram (all)

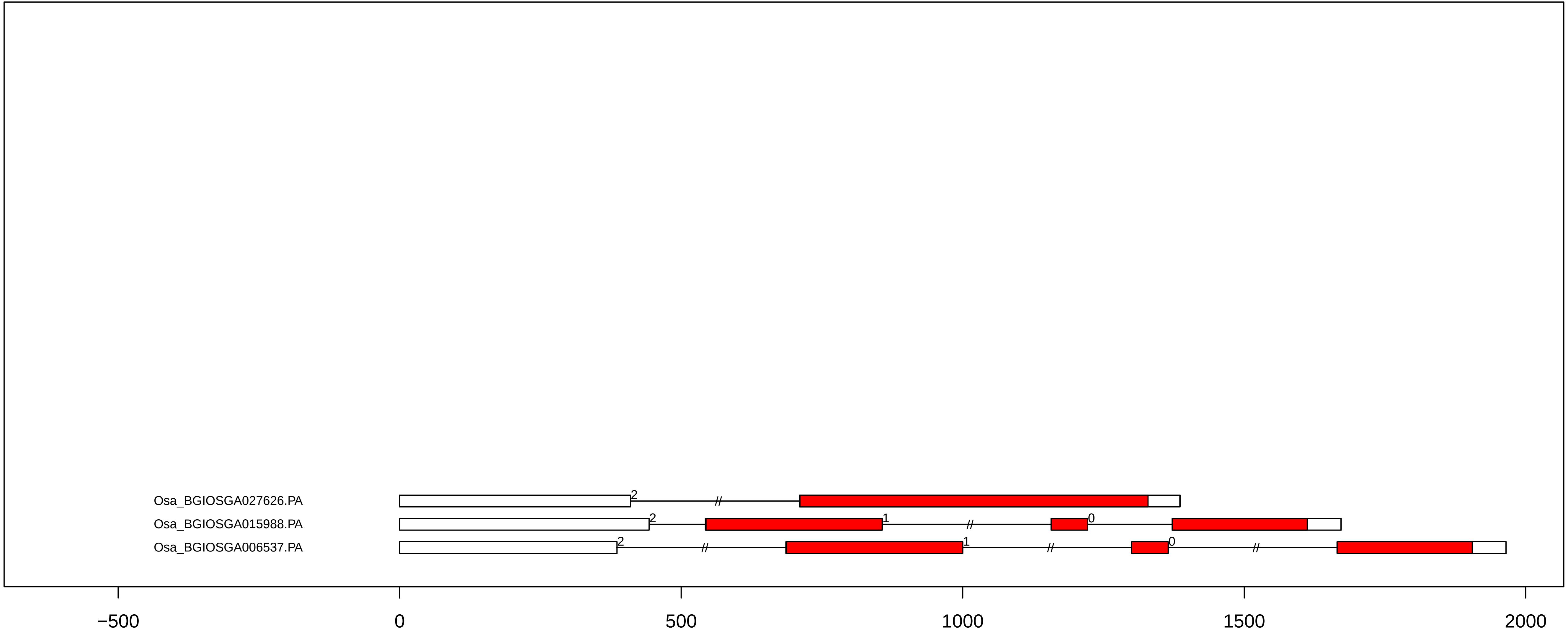

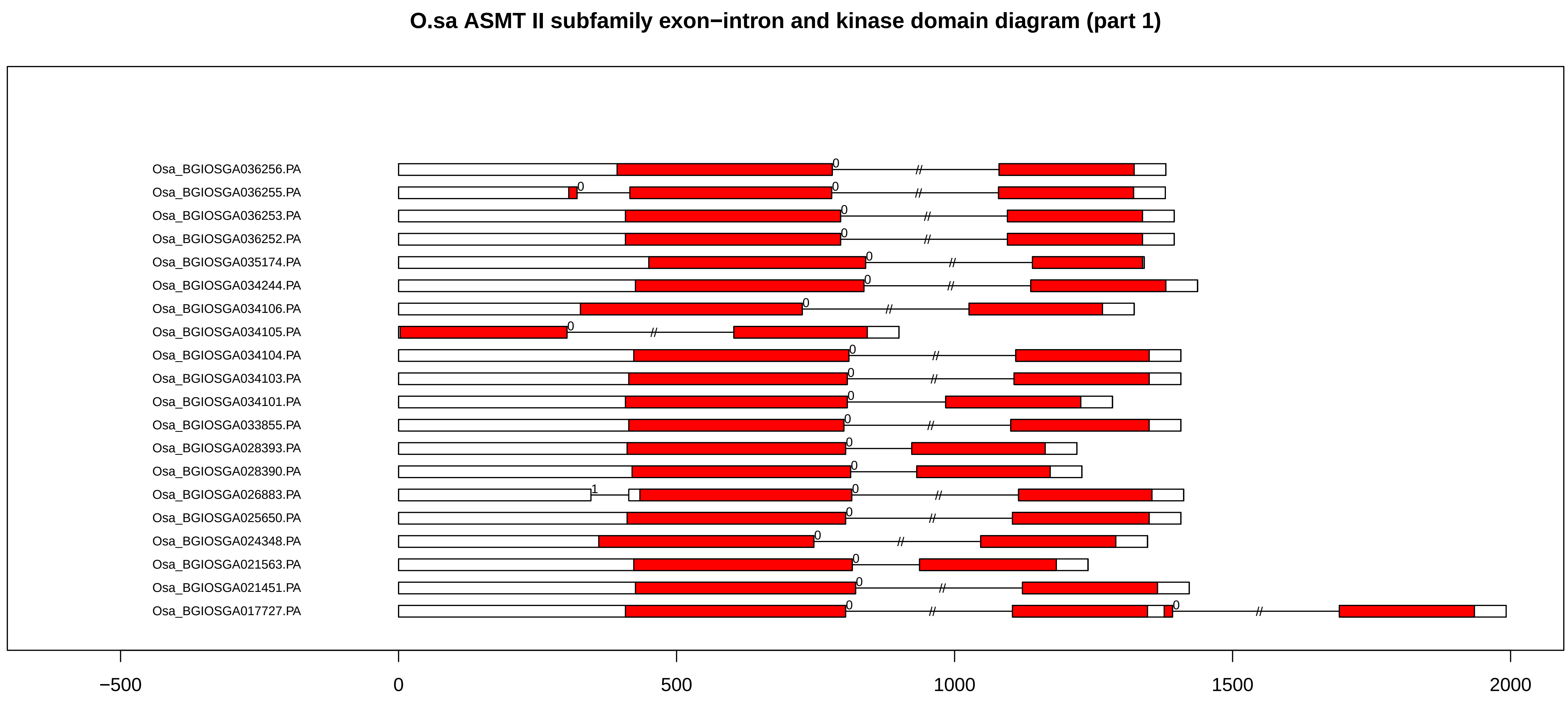

O.sa ASMT II subfamily exon−intron and kinase domain diagram (part 1)
Osa_BGIOSGA036256.PA
Osa_BGIOSGA036255.PA
Osa_BGIOSGA036253.PA
Osa_BGIOSGA036252.PA
Osa_BGIOSGA035174.PA
Osa_BGIOSGA034244.PA
Osa_BGIOSGA034106.PA
Osa_BGIOSGA034105.PA
Osa_BGIOSGA034104.PA
Osa_BGIOSGA034103.PA
Osa_BGIOSGA034101.PA
Osa_BGIOSGA033855.PA
Osa_BGIOSGA028393.PA
Osa_BGIOSGA028390.PA
Osa_BGIOSGA026883.PA
Osa_BGIOSGA025650.PA
Osa_BGIOSGA024348.PA
Osa_BGIOSGA021563.PA
Osa_BGIOSGA021451.PA
Osa_BGIOSGA017727.PA
−500
0
500
1000
1500
2000

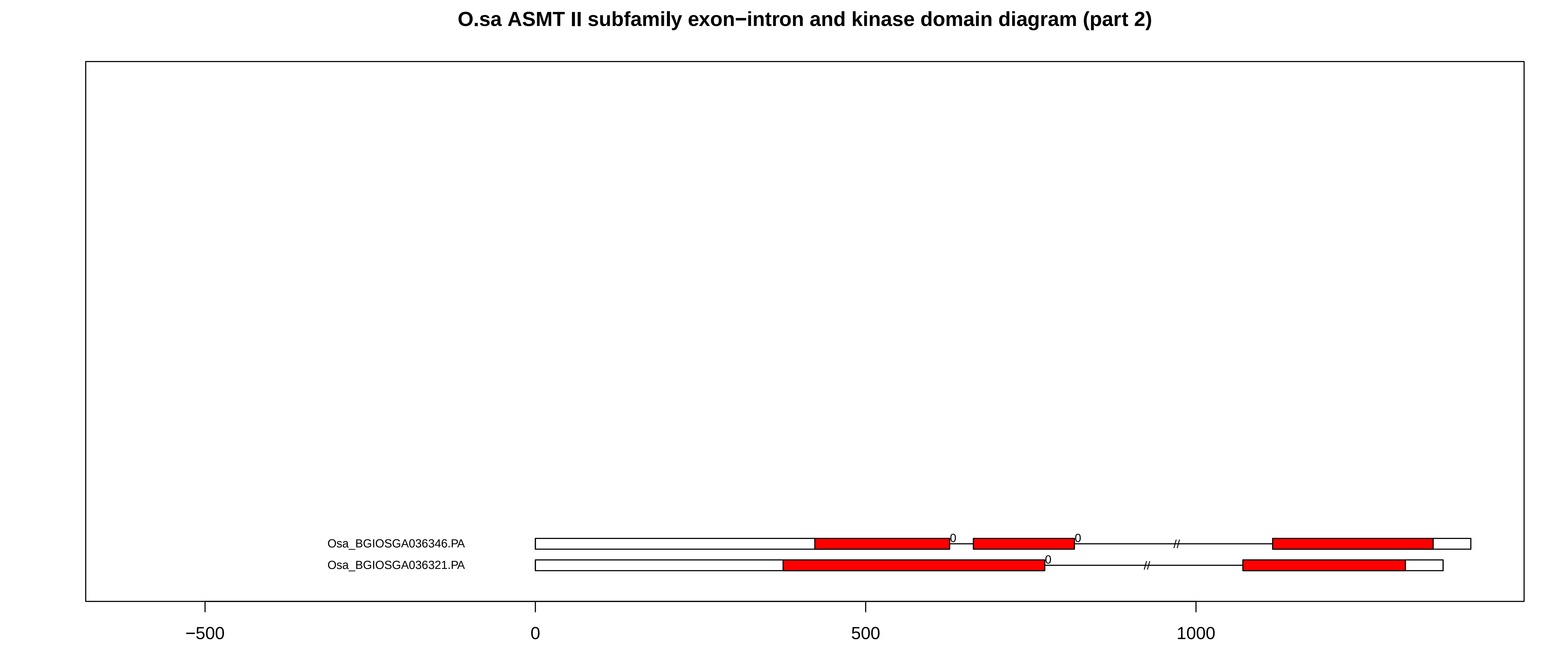
O.sa ASMT II subfamily exon−intron and kinase domain diagram (part 2)
Osa_BGIOSGA036346.PA
Osa_BGIOSGA036321.PA
0
0
0
−500
0
500
1000

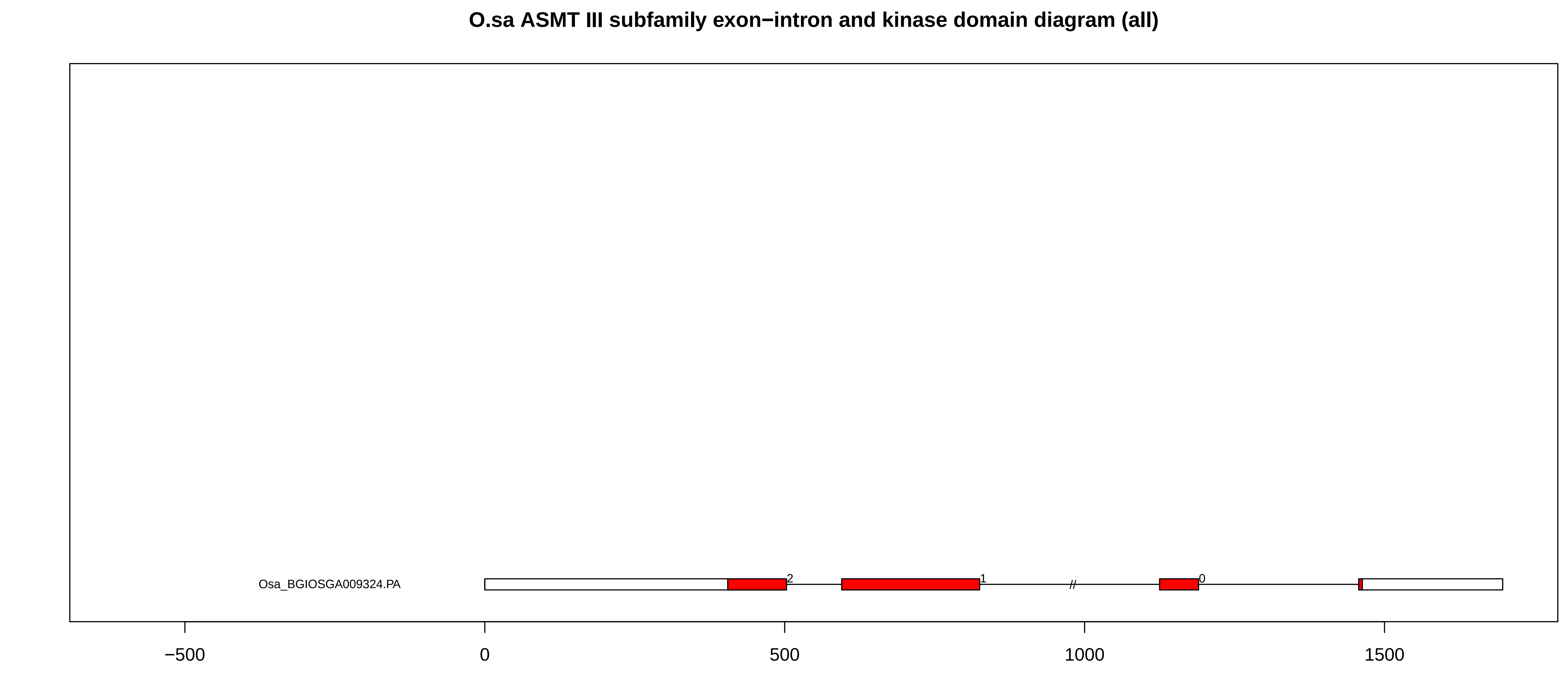
O.sa ASMT III subfamily exon−intron and kinase domain diagram (all)
Osa_BGIOSGA009324.PA
2
1
0
//
−500
0
500
1000
1500

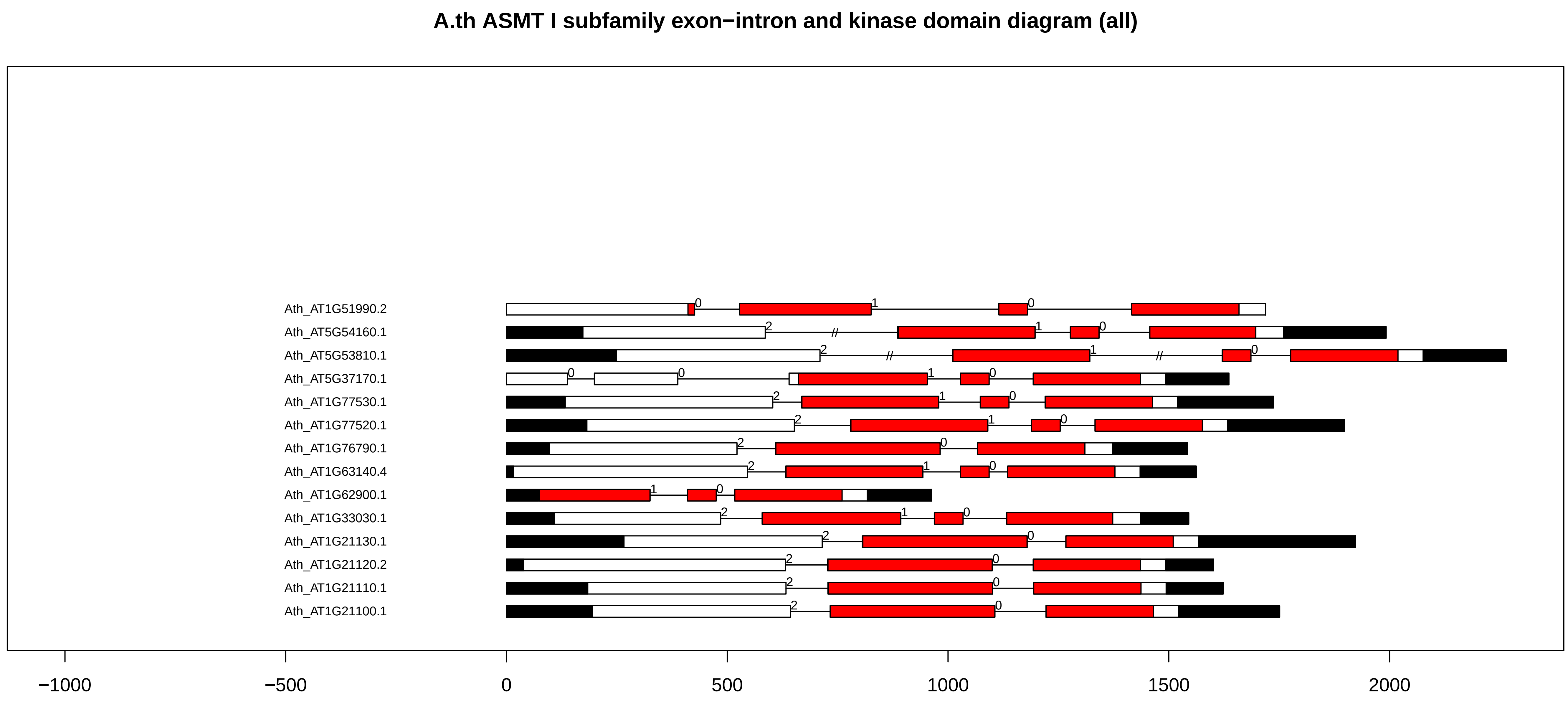
A.th ASMT I subfamily exon−intron and kinase domain diagram (all)
Ath_AT1G51990.2
Ath_AT5G54160.1
Ath_AT5G53810.1
Ath_AT5G37170.1
Ath_AT1G77530.1
Ath_AT1G77520.1
Ath_AT1G76790.1
Ath_AT1G63140.4
Ath_AT1G62900.1
Ath_AT1G33030.1
Ath_AT1G21130.1
Ath_AT1G21120.2
Ath_AT1G21110.1
Ath_AT1G21100.1
−1000
−500
0
500
1000
1500
2000

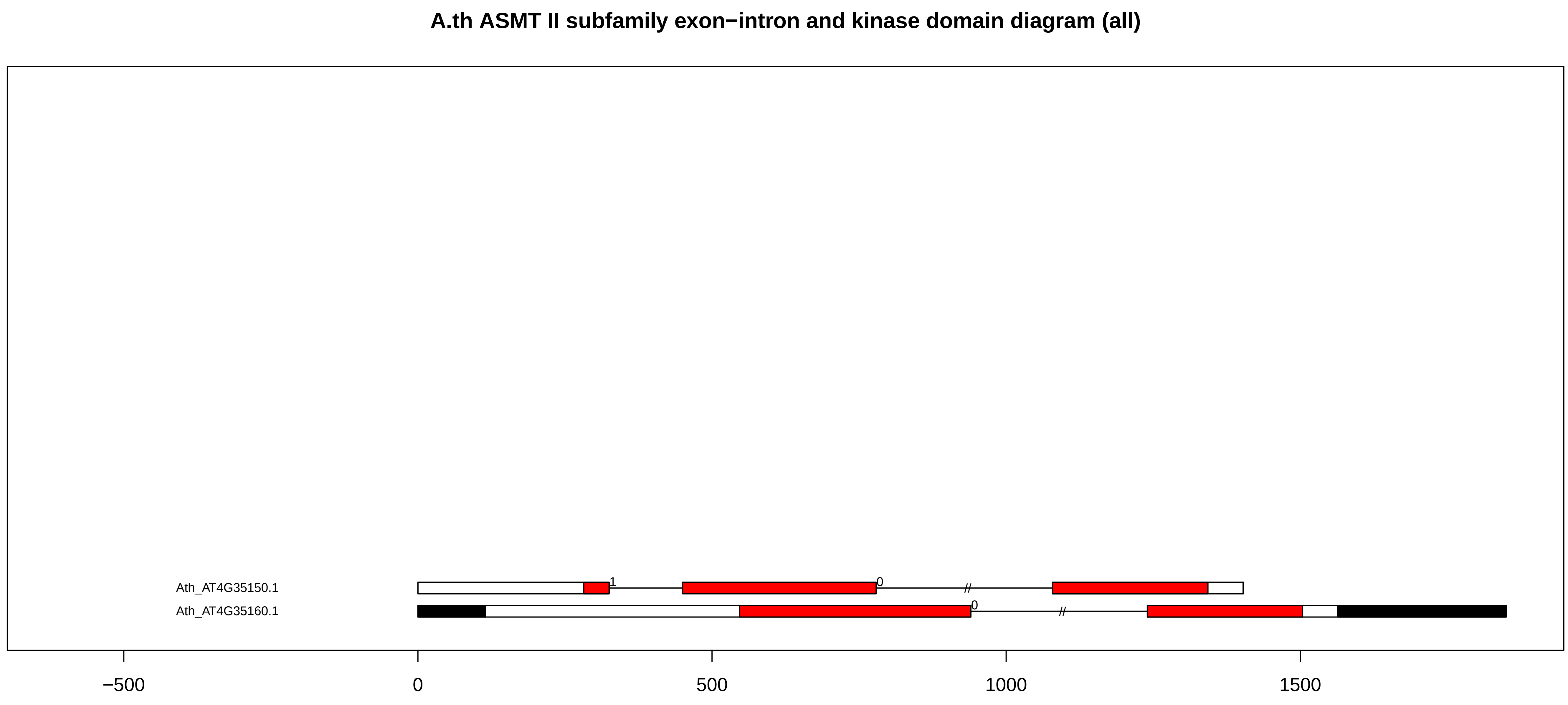
A.th ASMT II subfamily exon−intron and kinase domain diagram (all)
Ath_AT4G35150.1
Ath_AT4G35160.1
1
0
0
−500
0
500
1000
1500

A.th ASMT III subfamily exon−intron and kinase domain diagram (all)

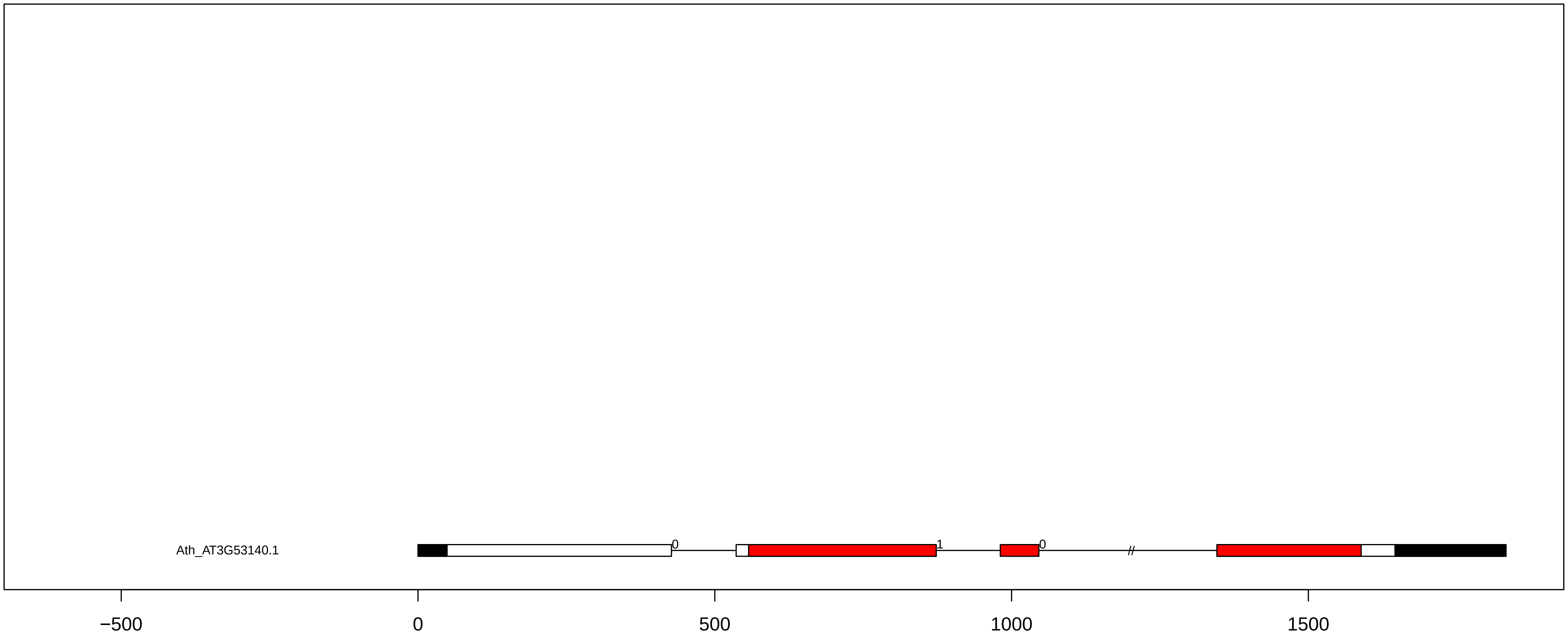

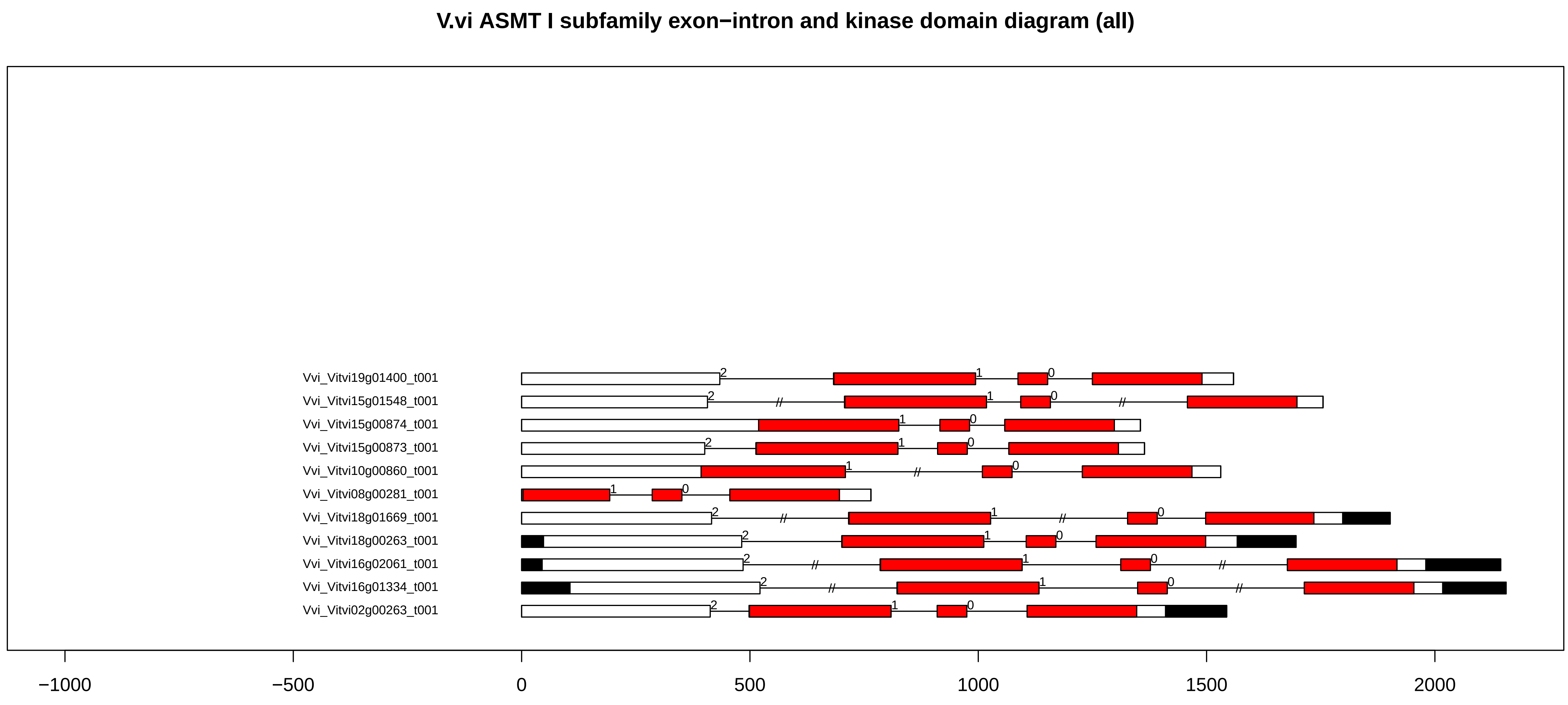
V.vi ASMT I subfamily exon−intron and kinase domain diagram (all)
Vvi_Vitvi19g01400_t001
Vvi_Vitvi15g01548_t001
Vvi_Vitvi15g00874_t001
Vvi_Vitvi15g00873_t001
Vvi_Vitvi10g00860_t001
Vvi_Vitvi08g00281_t001
Vvi_Vitvi18g01669_t001
Vvi_Vitvi18g00263_t001
Vvi_Vitvi16g02061_t001
Vvi_Vitvi16g01334_t001
Vvi_Vitvi02g00263_t001
−1000
−500
0
500
1000
1500
2000

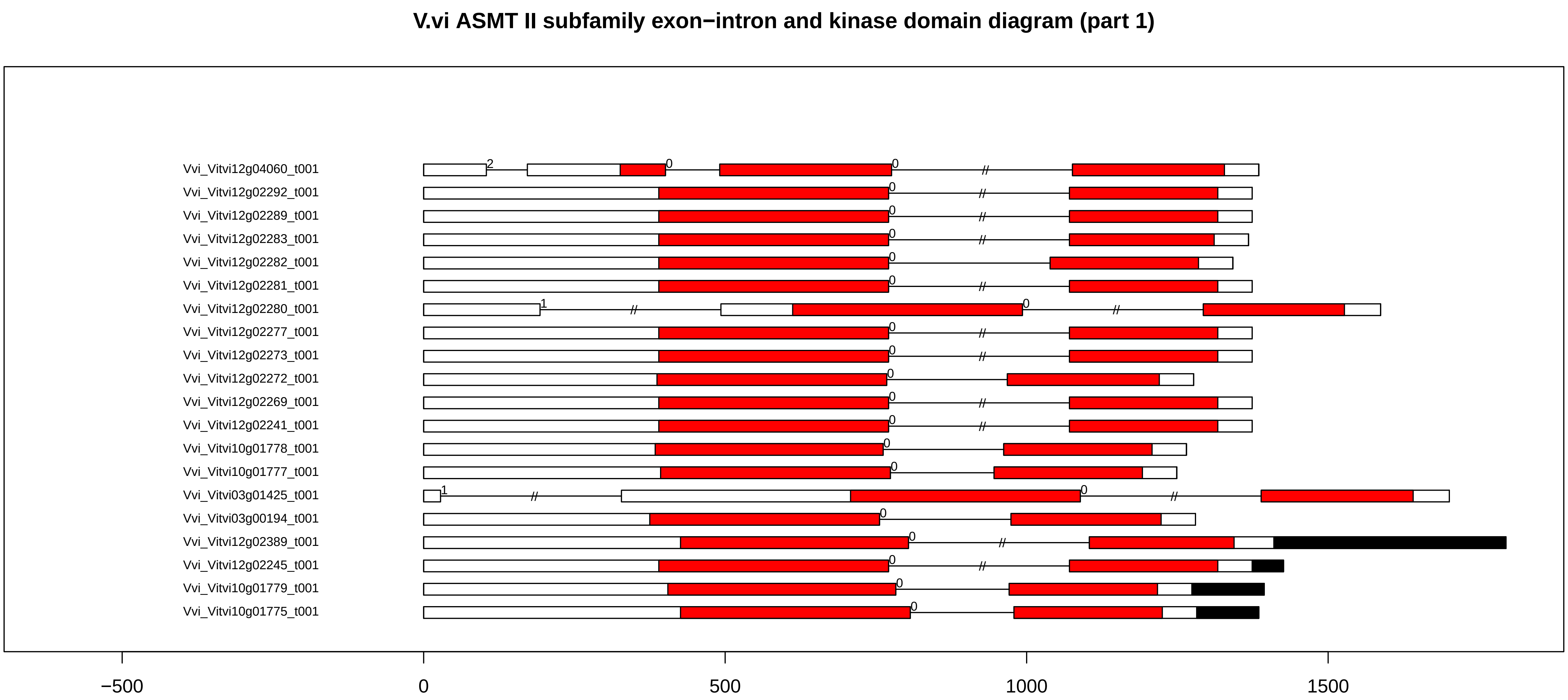

V.vi ASMT II subfamily exon−intron and kinase domain diagram (part 1)
Vvi_Vitvi12g04060_t001
Vvi_Vitvi12g02292_t001
Vvi_Vitvi12g02289_t001
Vvi_Vitvi12g02283_t001
Vvi_Vitvi12g02282_t001
Vvi_Vitvi12g02281_t001
Vvi_Vitvi12g02280_t001
Vvi_Vitvi12g02277_t001
Vvi_Vitvi12g02273_t001
Vvi_Vitvi12g02272_t001
Vvi_Vitvi12g02269_t001
Vvi_Vitvi12g02241_t001
Vvi_Vitvi10g01778_t001
Vvi_Vitvi10g01777_t001
Vvi_Vitvi03g01425_t001
Vvi_Vitvi03g00194_t001
Vvi_Vitvi12g02389_t001
Vvi_Vitvi12g02245_t001
Vvi_Vitvi10g01779_t001
Vvi_Vitvi10g01775_t001
−500
0
500
1000
1500

V.vi ASMT II subfamily exon−intron and kinase domain diagram (part 2)

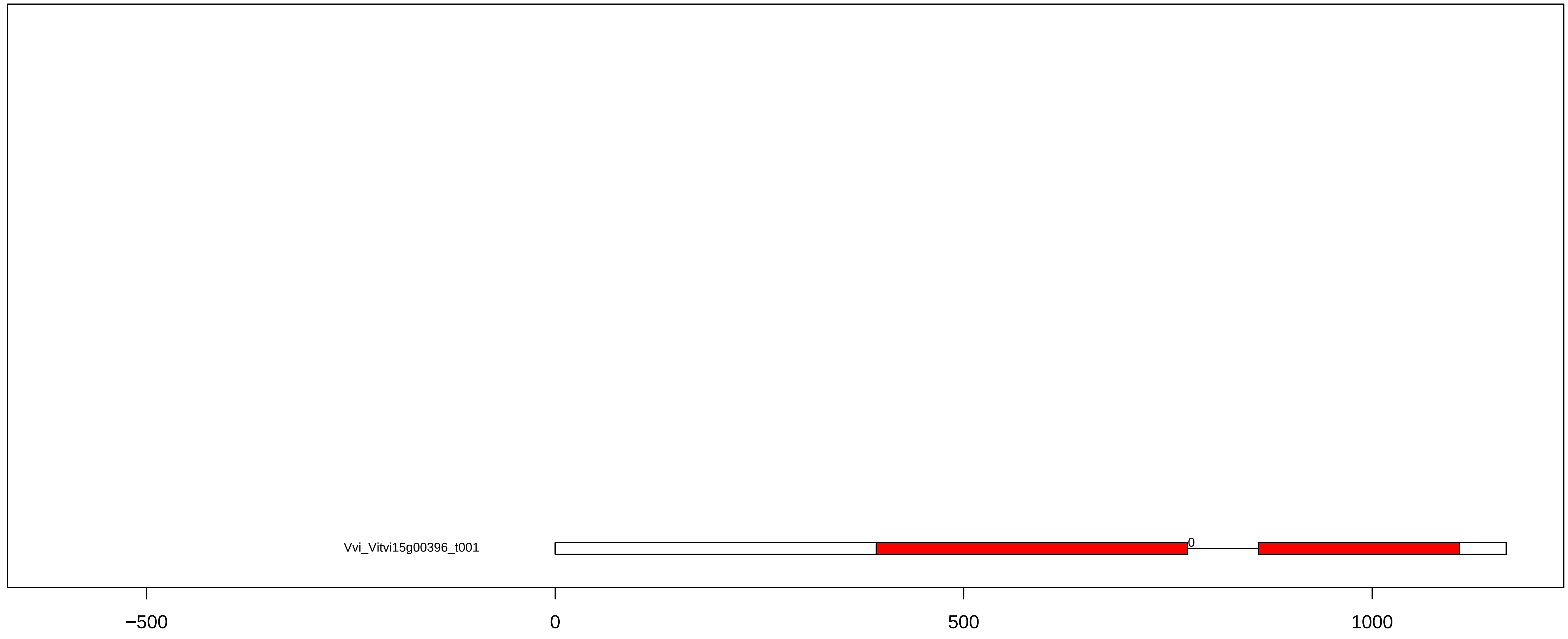

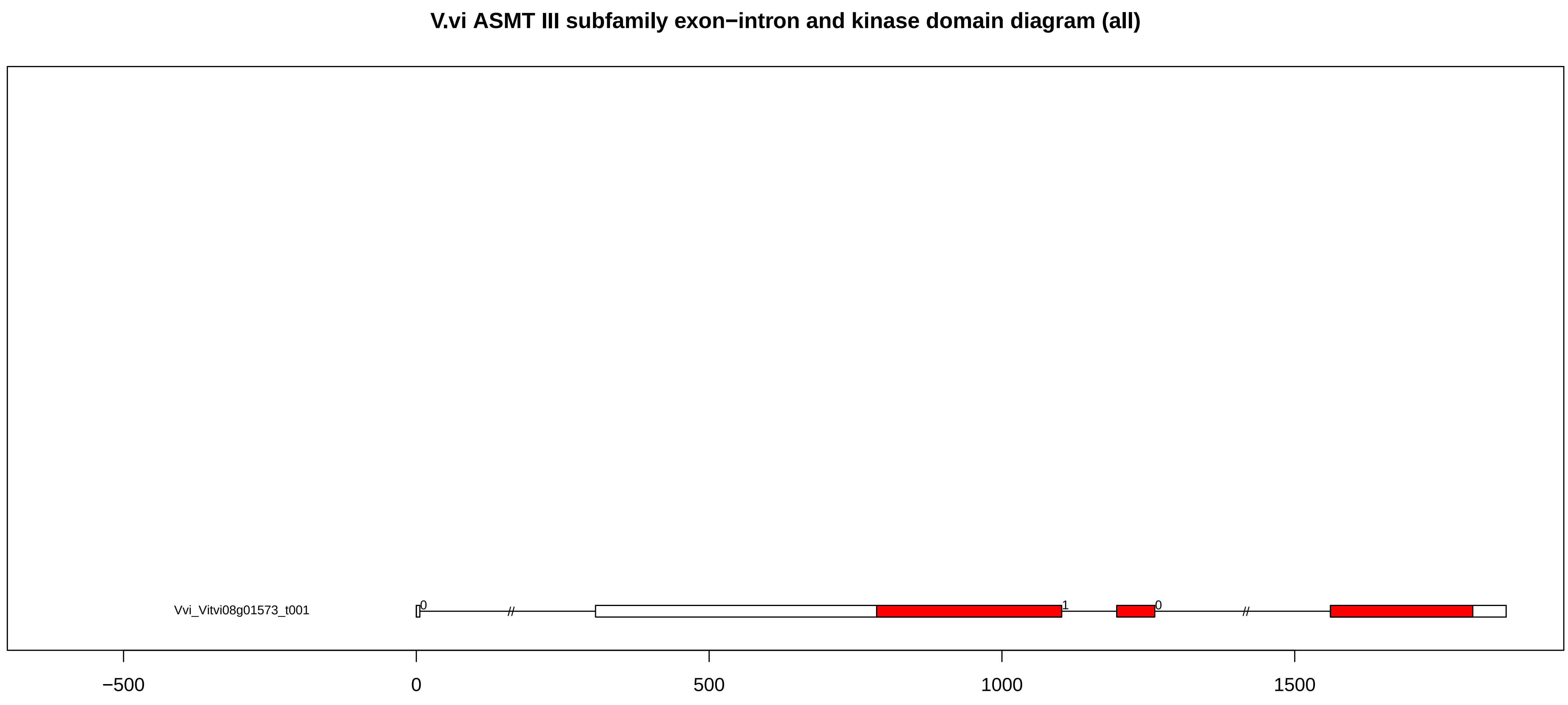
V.vi ASMT III subfamily exon−intron and kinase domain diagram (all)
Vvi_Vitvi08g01573_t001
0
1
0
−500
0
500
1000
1500

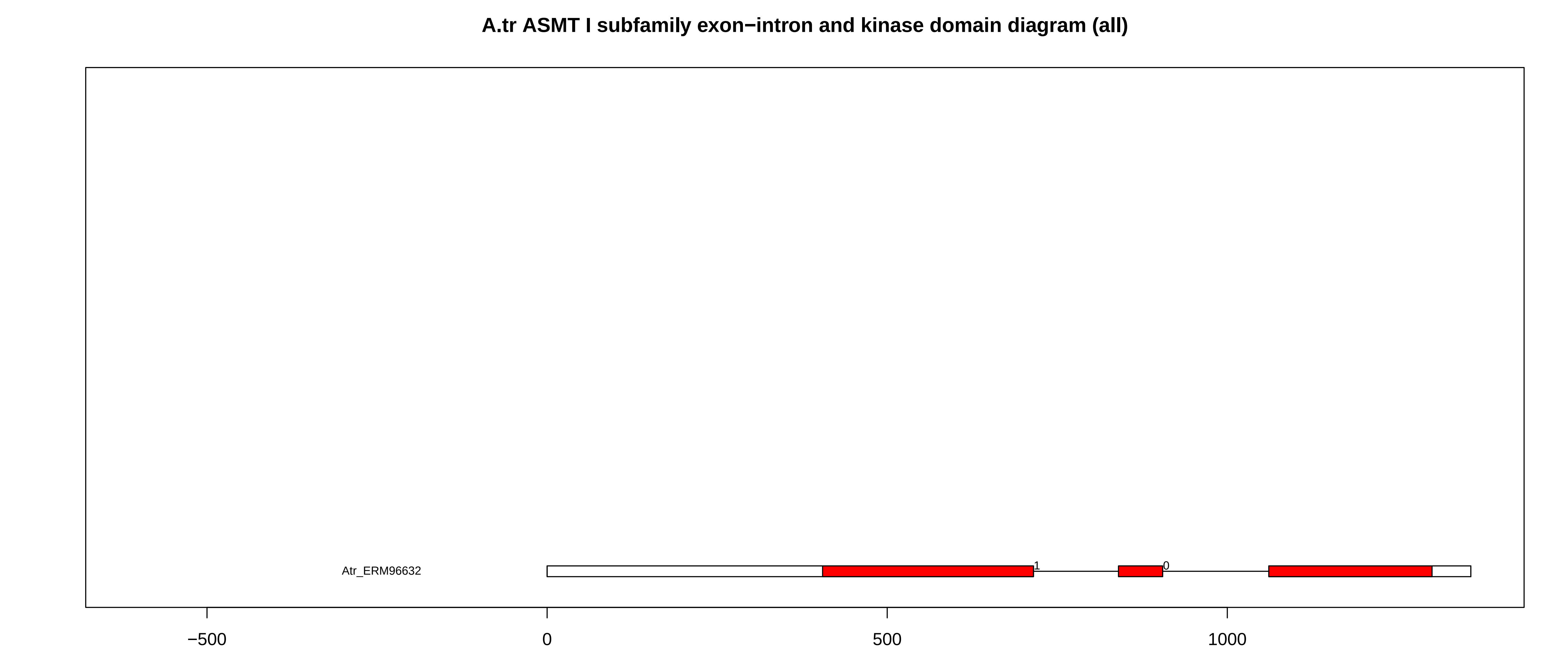

A.tr ASMT I subfamily exon−intron and kinase domain diagram (all)
Atr_ERM96632
1
0
−500
0
500
1000

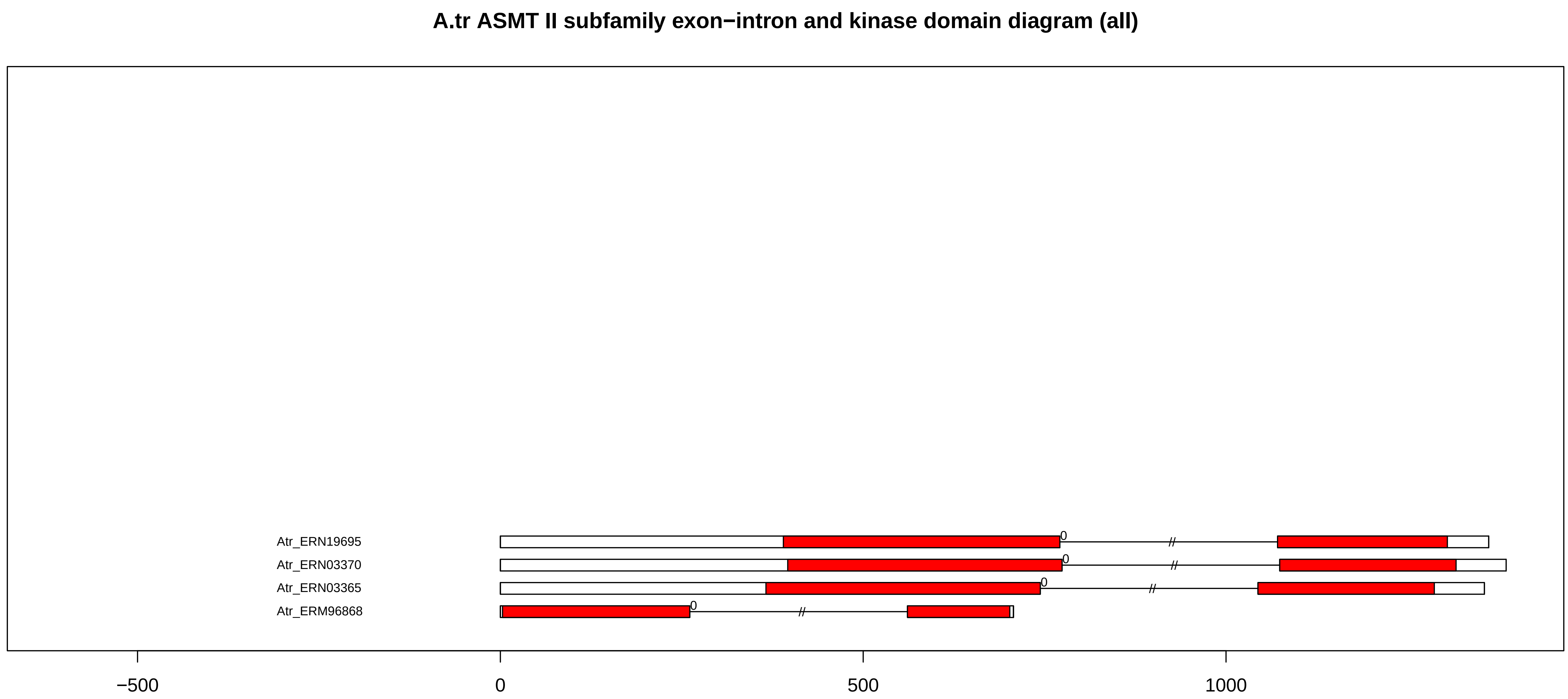
A.tr ASMT II subfamily exon−intron and kinase domain diagram (all)
Atr_ERN19695
Atr_ERN03370
Atr_ERN03365
Atr_ERM96868
0
0
0
0
−500
0
500
1000

A.tr ASMT III subfamily exon−intron and kinase domain diagram (all)

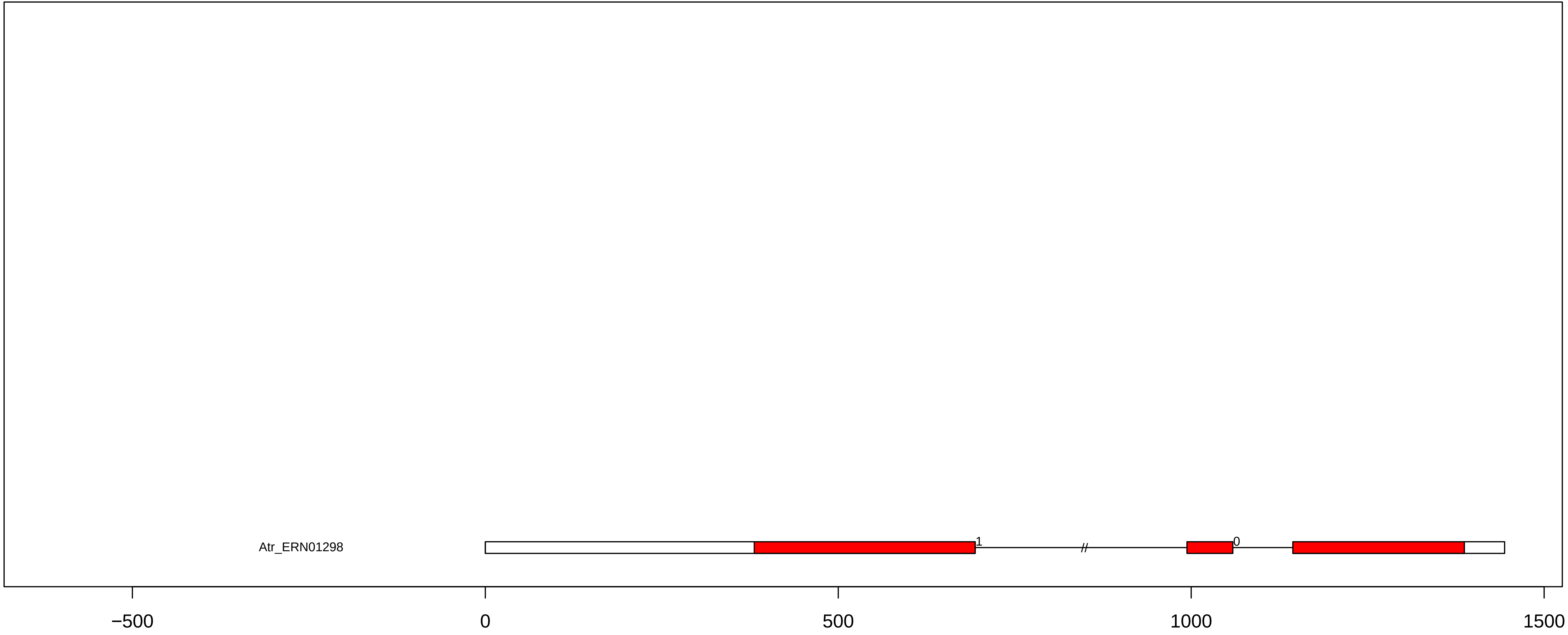

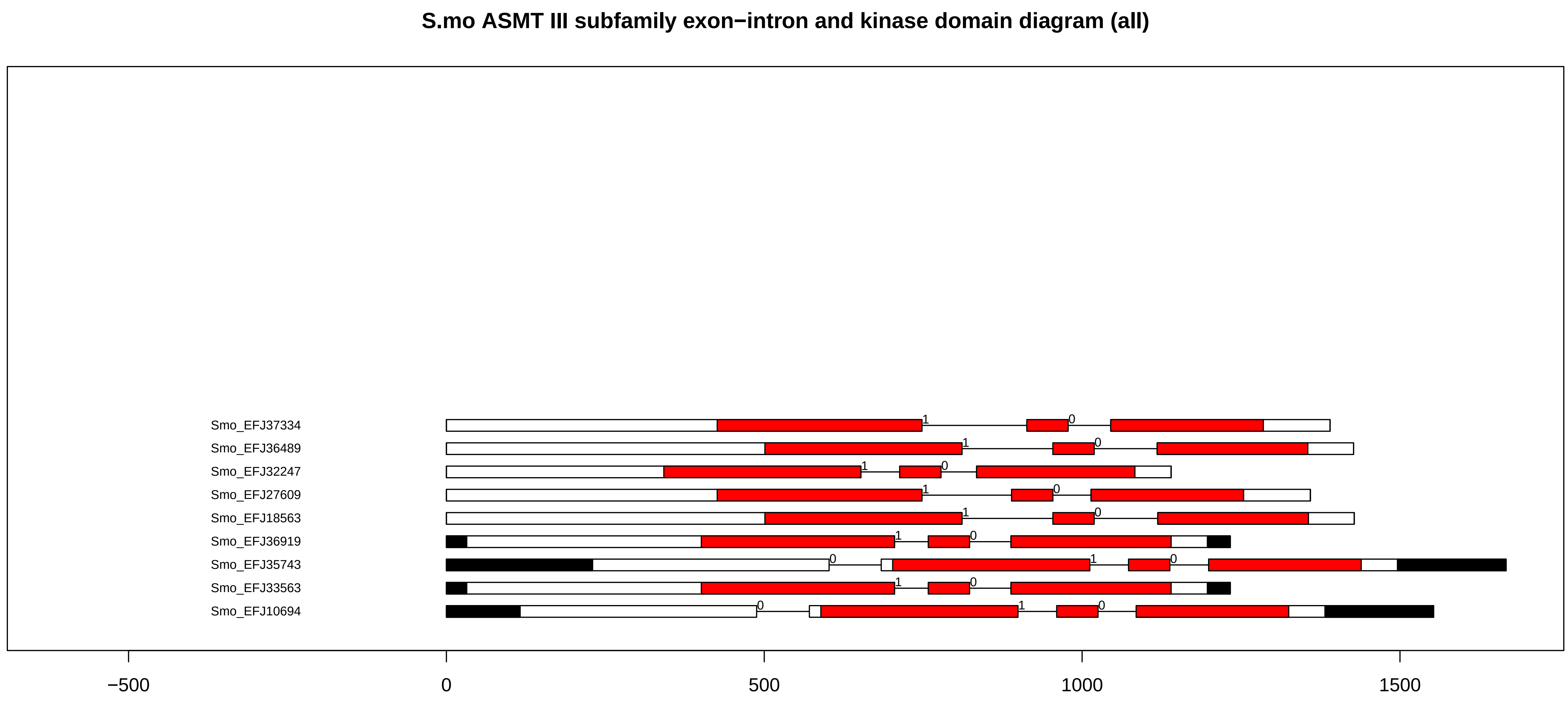
S.mo ASMT III subfamily exon−intron and kinase domain diagram (all)
Smo_EFJ37334
Smo_EFJ36489
Smo_EFJ32247
Smo_EFJ27609
Smo_EFJ18563
Smo_EFJ36919
Smo_EFJ35743
Smo_EFJ33563
Smo_EFJ10694
−500
0
500
1000
1500

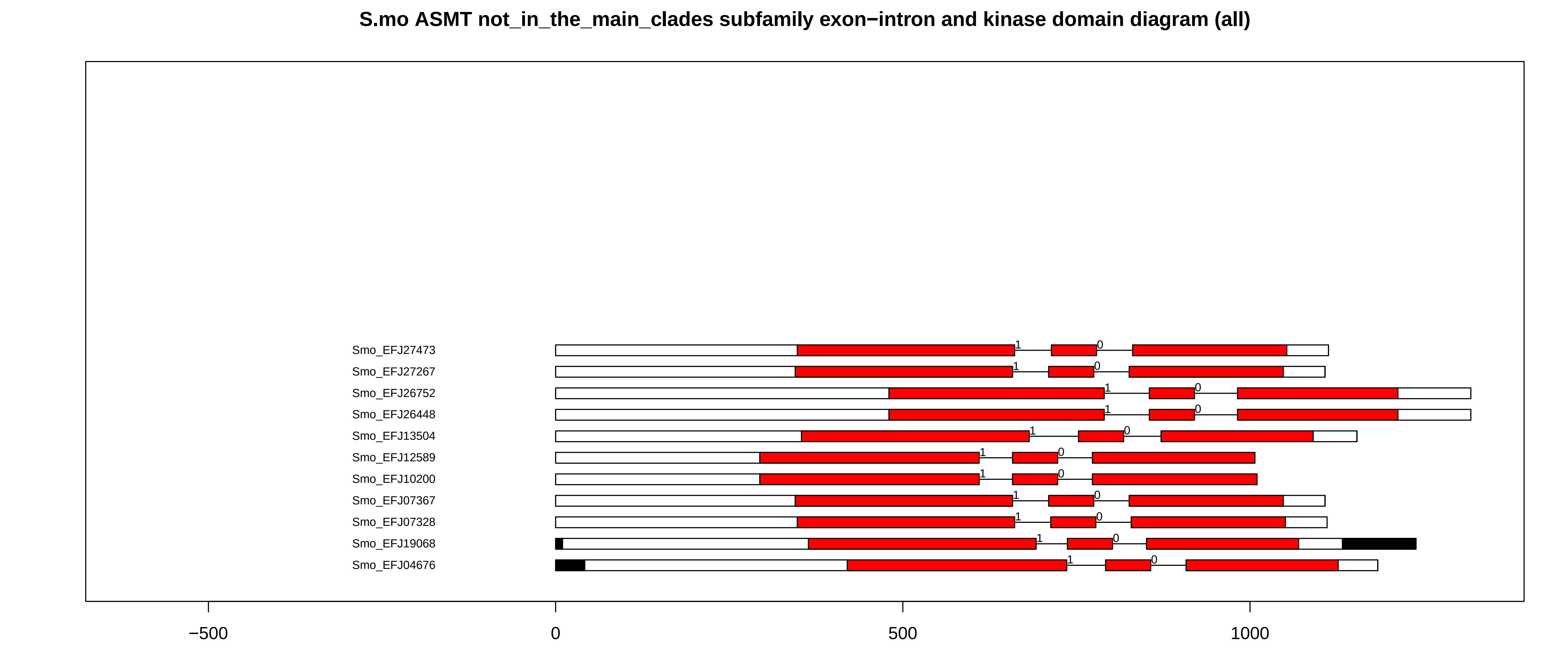
S.mo ASMT not_in_the_main_clades subfamily exon−intron and kinase domain diagram (all)
Smo_EFJ27473
Smo_EFJ27267
Smo_EFJ26752
Smo_EFJ26448
Smo_EFJ13504
Smo_EFJ12589
Smo_EFJ10200
Smo_EFJ07367
Smo_EFJ07328
Smo_EFJ19068
Smo_EFJ04676
−500
0
500
1000

P.pa ASMT III subfamily exon−intron and kinase domain diagram (all)

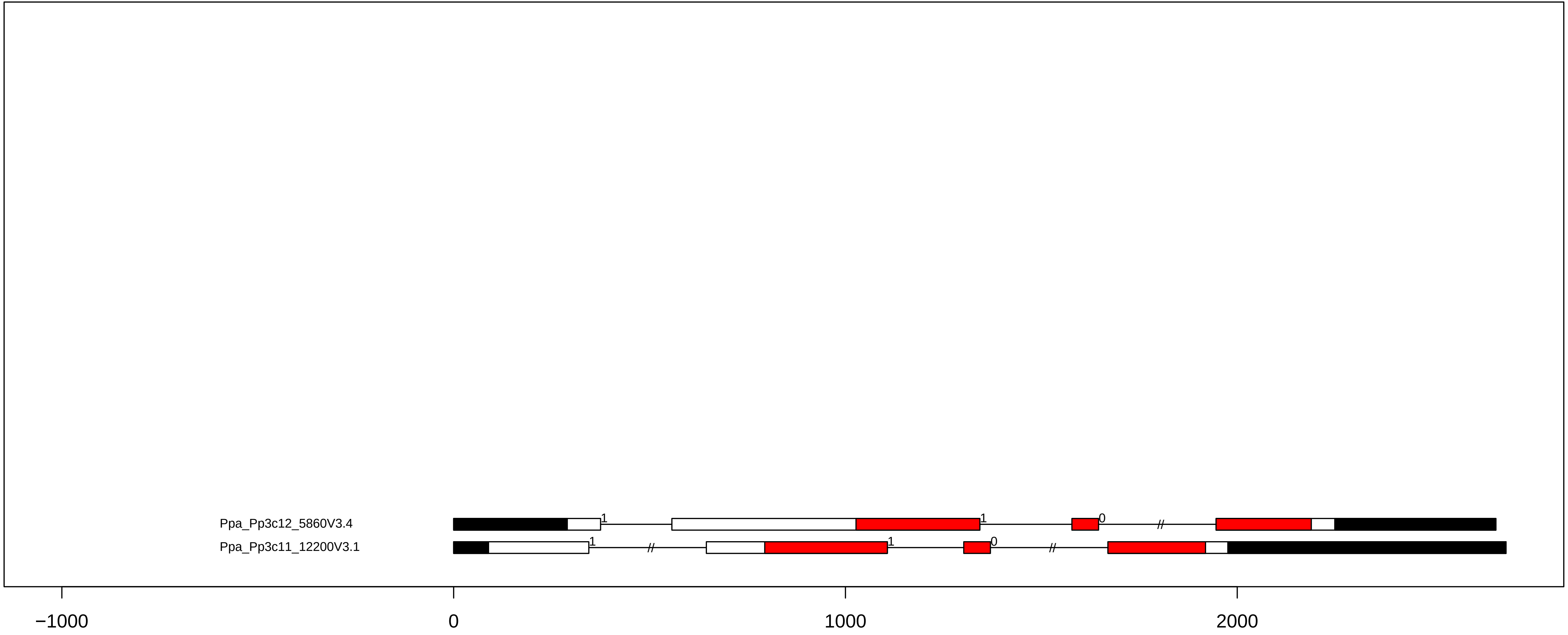

P.pa ASMT not_in_the_main_clades subfamily exon−intron and kinase domain diagram (all)

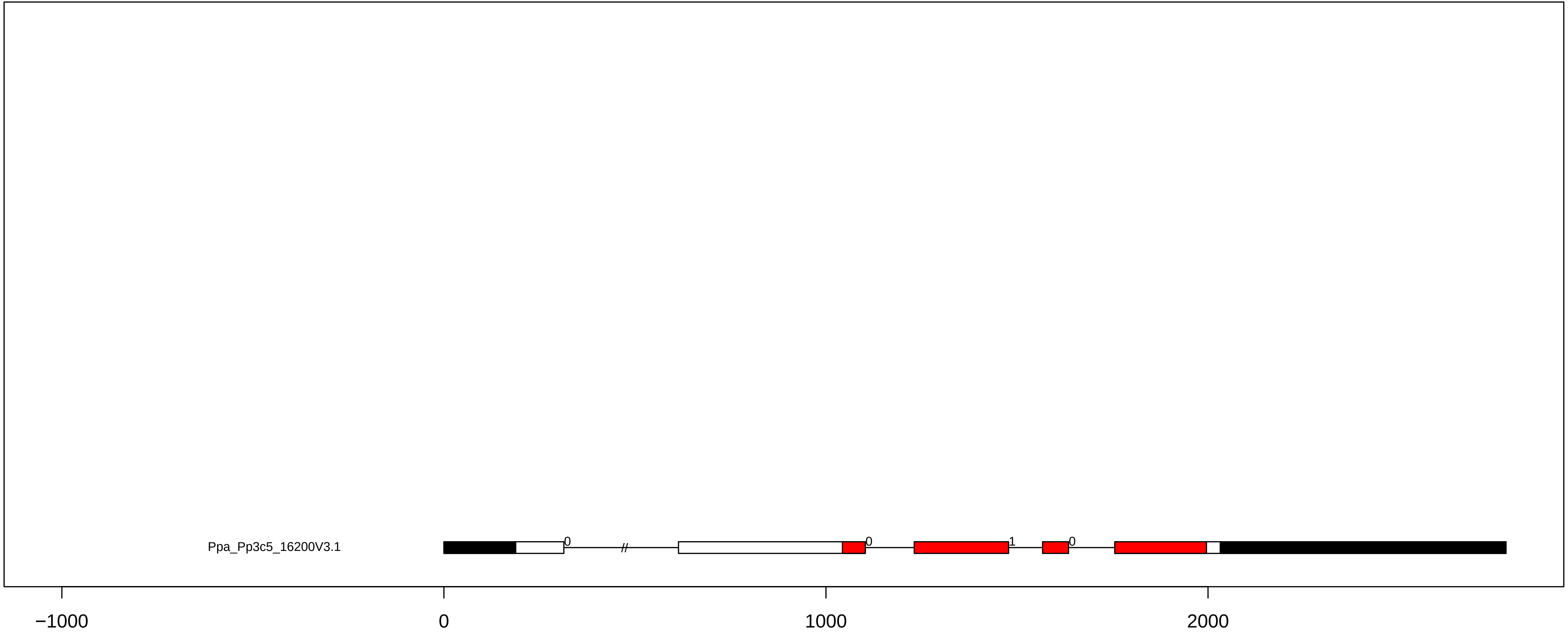

Supplement: Supplementary file 1 [file biology-15-01430-s001.zip › Figure/Figure S2.pdf]
