# Supplementary material for: Genome-Wide Identification of the ASMT Gene Family and Expression Analysis of Wheat ASMTs Under Abiotic and Biotic Stress
Source: Biology (Basel). 2026 Aug 19;15(16):1430. doi: 10.3390/biology15161430 (PMC13509220; doi:10.3390/biology15161430)
Supplement: Supplementary file 1 [file biology-15-01430-s001.zip › Figure/Figure S3.pdf]

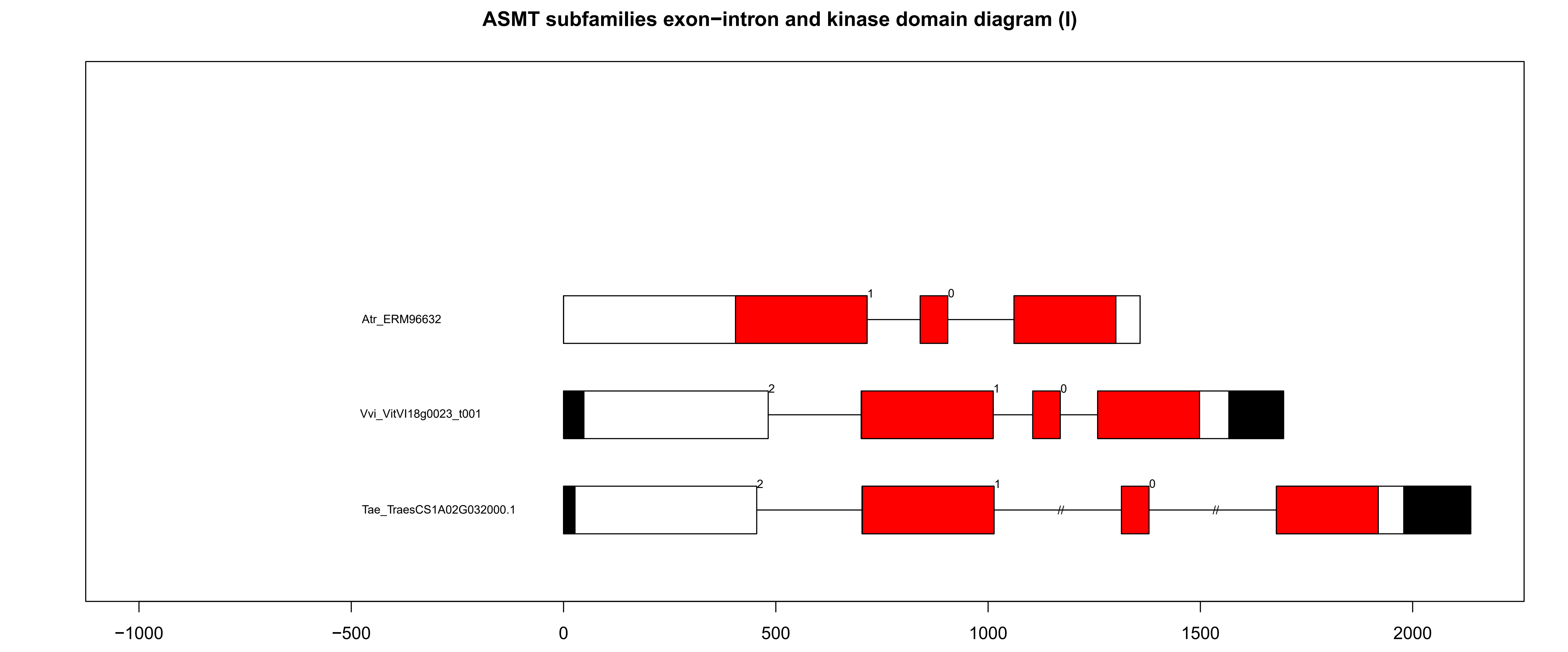
ASMT subfamilies exon−intron and kinase domain diagram (I)
Atr_ERM96632
Vvi_VitVI18g0023_t001
Tae_TraesCS1A02G032000.1
1
0
2
1
0
2
1
0
−1000
−500
0
500
1000
1500
2000

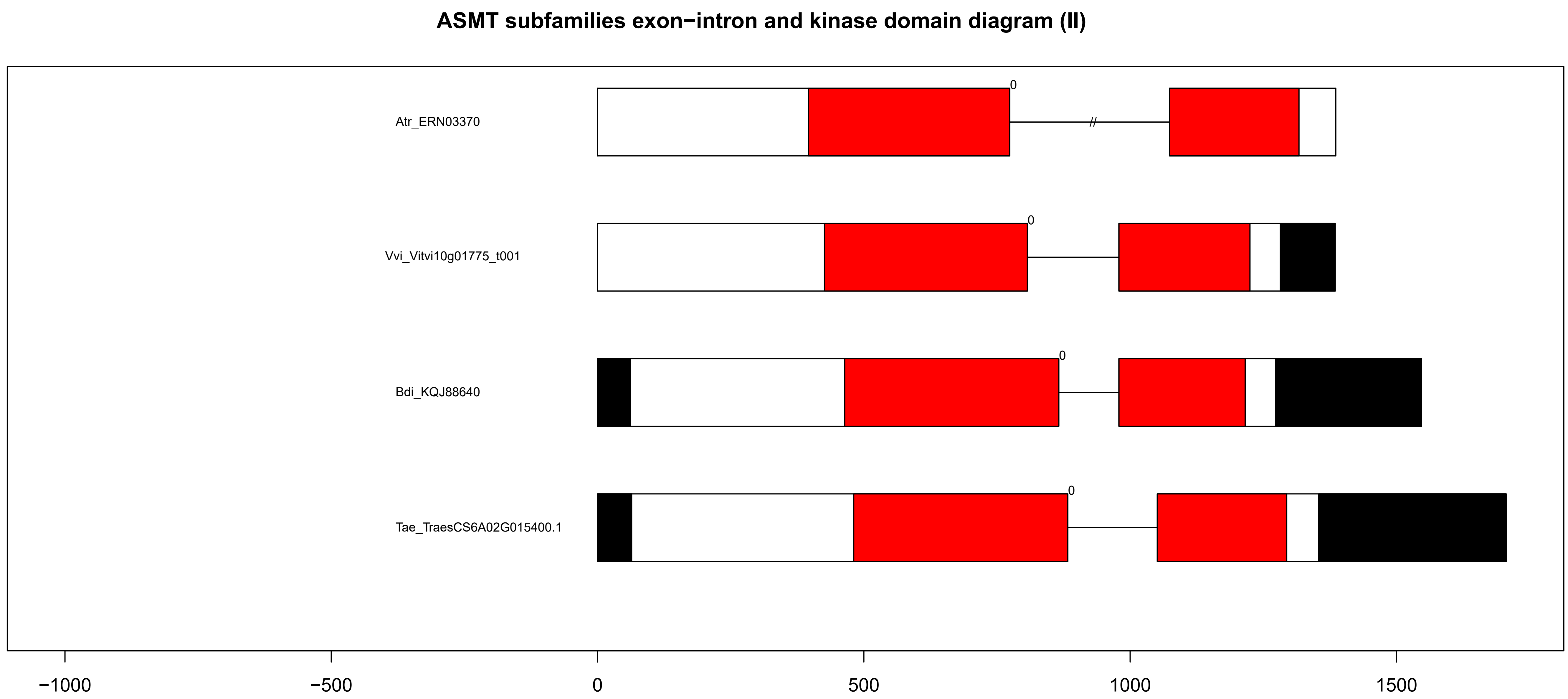
ASMT subfamilies exon−intron and kinase domain diagram (II)
Atr_ERN03370
Vvi_Vitvi10g01775_t001
Bdi_KQJ88640
Tae_TraesCS6A02G015400.1
0
0
0
0
−1000
−500
0
500
1000
1500

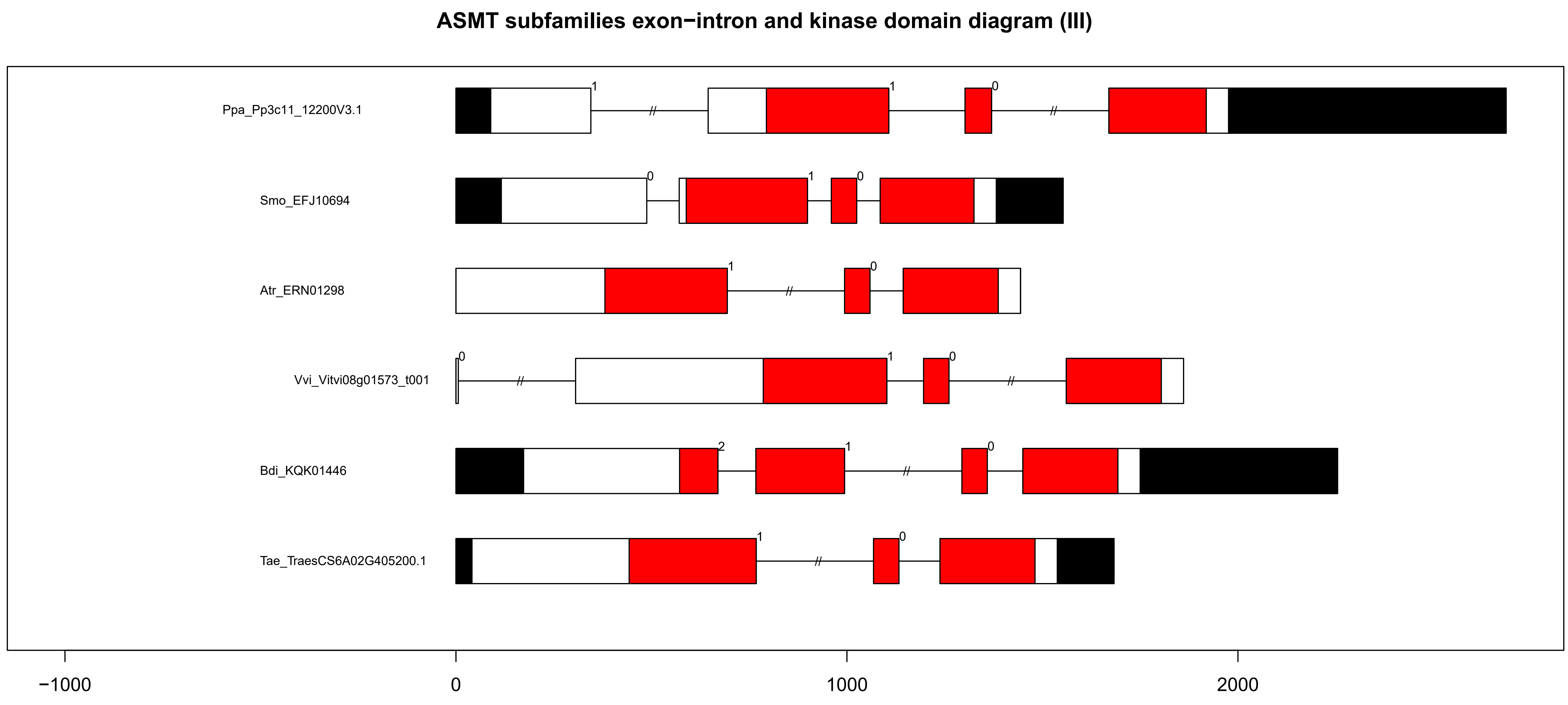

ASMT subfamilies exon−intron and kinase domain diagram (III)
Ppa_Pp3c11_12200V3.1
Smo_EFJ10694
Atr_ERN01298
Vvi_Vitvi08g01573_t001
Bdi_KQK01446
Tae_TraesCS6A02G405200.1
1
1
0
0
1
0
1
0
0
1
0
2
1
0
1
0
−1000
0
1000
2000
