# Supplementary material for: Genome-Wide Identification of the ASMT Gene Family and Expression Analysis of Wheat ASMTs Under Abiotic and Biotic Stress
Source: Biology (Basel). 2026 Aug 19;15(16):1430. doi: 10.3390/biology15161430 (PMC13509220; doi:10.3390/biology15161430)
Supplement: Supplementary file 1 [file biology-15-01430-s001.zip › Figure/Figure S4.pdf]

T.ae ASMT I subfamily domain diagram (part 1)

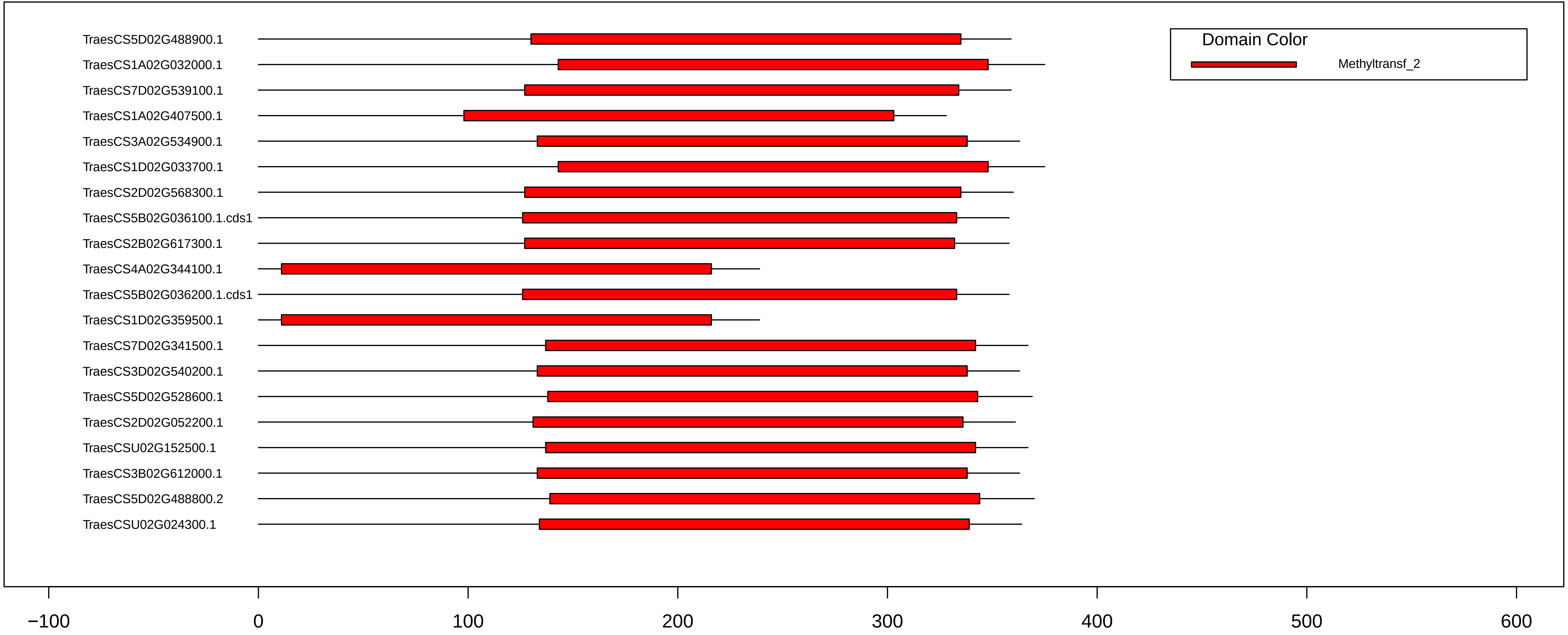

T.ae ASMT I subfamily domain diagram (part 2)

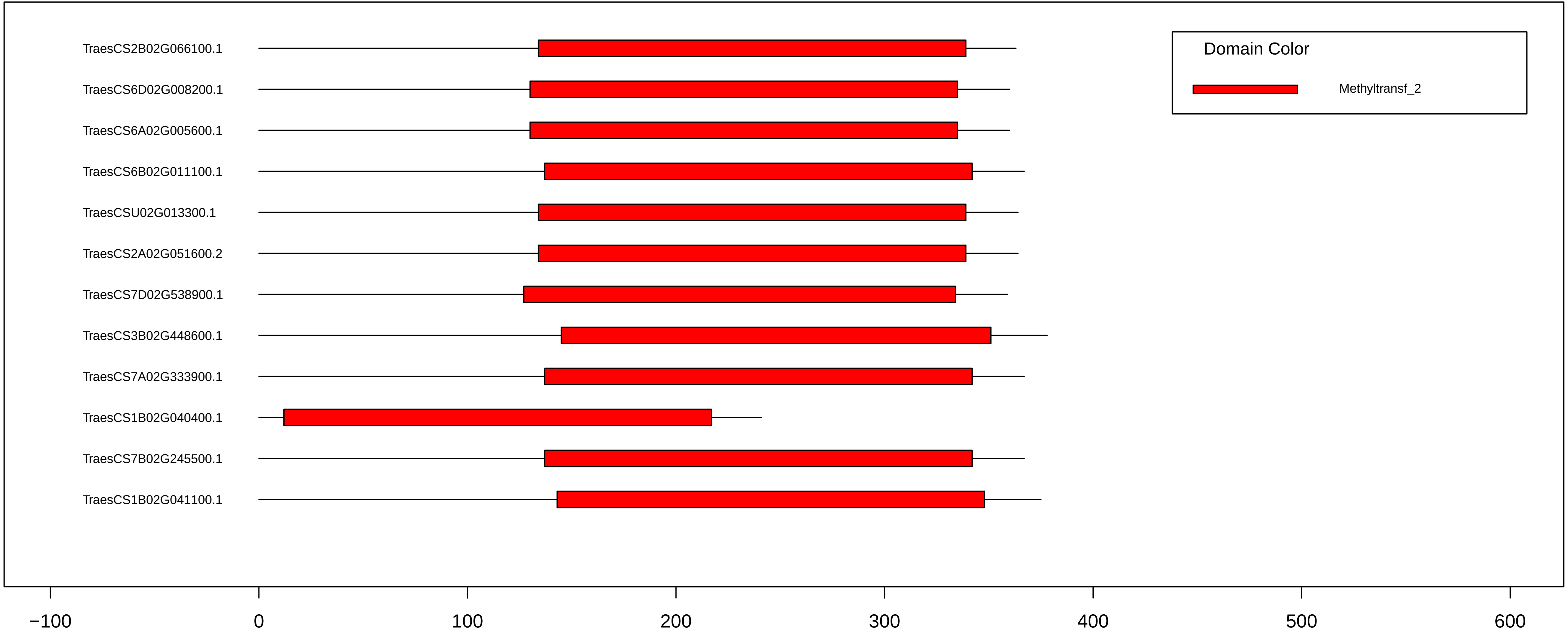

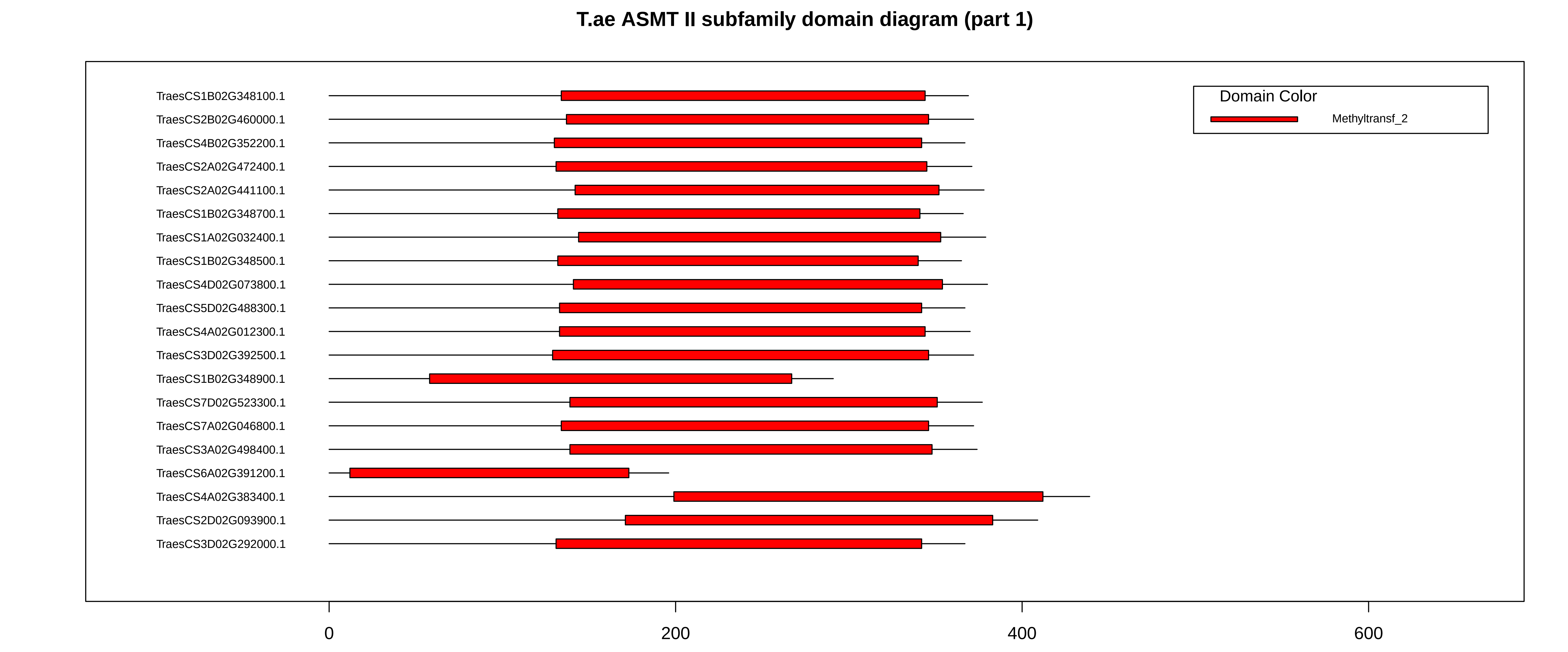
T.ae ASMT II subfamily domain diagram (part 1)
TraesCS1B02G348100.1
TraesCS2B02G460000.1
TraesCS4B02G352200.1
TraesCS2A02G472400.1
TraesCS2A02G441100.1
TraesCS1B02G348700.1
TraesCS1A02G032400.1
TraesCS1B02G348500.1
TraesCS4D02G073800.1
TraesCS5D02G488300.1
TraesCS4A02G012300.1
TraesCS3D02G392500.1
TraesCS1B02G348900.1
TraesCS7D02G523300.1
TraesCS7A02G046800.1
TraesCS3A02G498400.1
TraesCS6A02G391200.1
TraesCS4A02G383400.1
TraesCS2D02G093900.1
TraesCS3D02G292000.1
Domain Color
Methyltransf_2
0
200
400
600

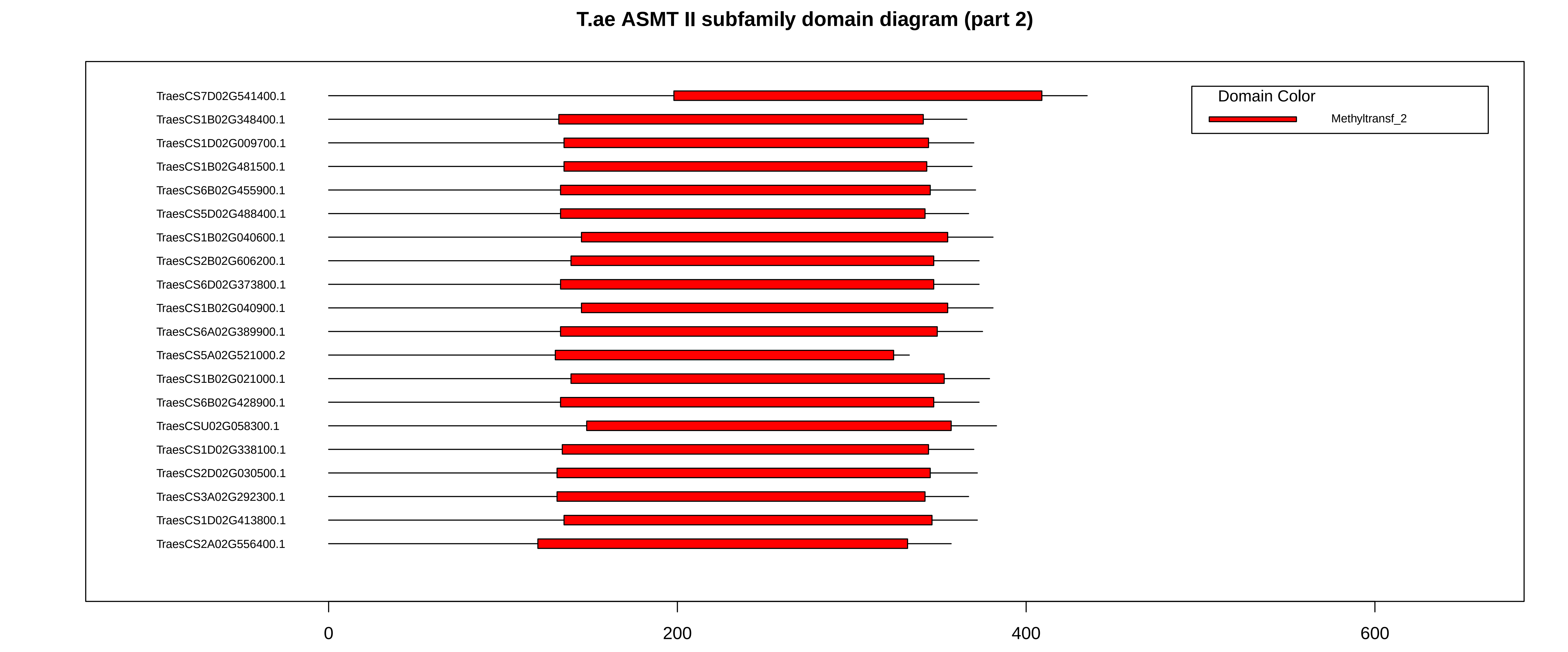
T.ae ASMT II subfamily domain diagram (part 2)
Domain Color
Methyltransf_2
TraesCS7D02G541400.1
TraesCS1B02G348400.1
TraesCS1D02G009700.1
TraesCS1B02G481500.1
TraesCS6B02G455900.1
TraesCS5D02G488400.1
TraesCS1B02G040600.1
TraesCS2B02G606200.1
TraesCS6D02G373800.1
TraesCS1B02G040900.1
TraesCS6A02G389900.1
TraesCS5A02G521000.2
TraesCS1B02G021000.1
TraesCS6B02G428900.1
TraesCSU02G058300.1
TraesCS1D02G338100.1
TraesCS2D02G030500.1
TraesCS3A02G292300.1
TraesCS1D02G413800.1
TraesCS2A02G556400.1
0
200
400
600

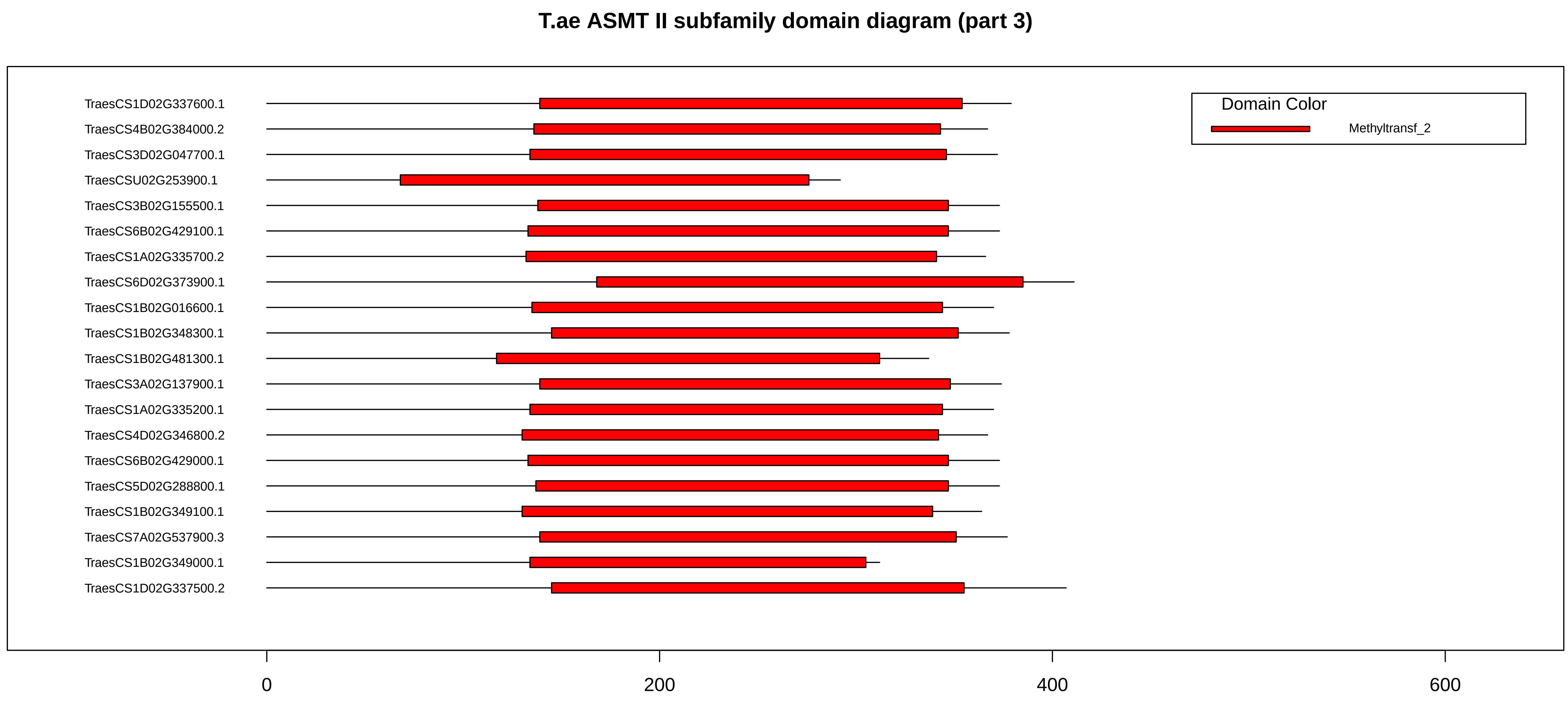
T.ae ASMT II subfamily domain diagram (part 3)
TraesCS1D02G337600.1
TraesCS4B02G384000.2
TraesCS3D02G047700.1
TraesCSU02G253900.1
TraesCS3B02G155500.1
TraesCS6B02G429100.1
TraesCS1A02G335700.2
TraesCS6D02G373900.1
TraesCS1B02G016600.1
TraesCS1B02G348300.1
TraesCS1B02G481300.1
TraesCS3A02G137900.1
TraesCS1A02G335200.1
TraesCS4D02G346800.2
TraesCS6B02G429000.1
TraesCS5D02G288800.1
TraesCS1B02G349100.1
TraesCS7A02G537900.3
TraesCS1B02G349000.1
TraesCS1D02G337500.2
Domain Color
Methyltransf_2
0
200
400
600

T.ae ASMT II subfamily domain diagram (part 4)

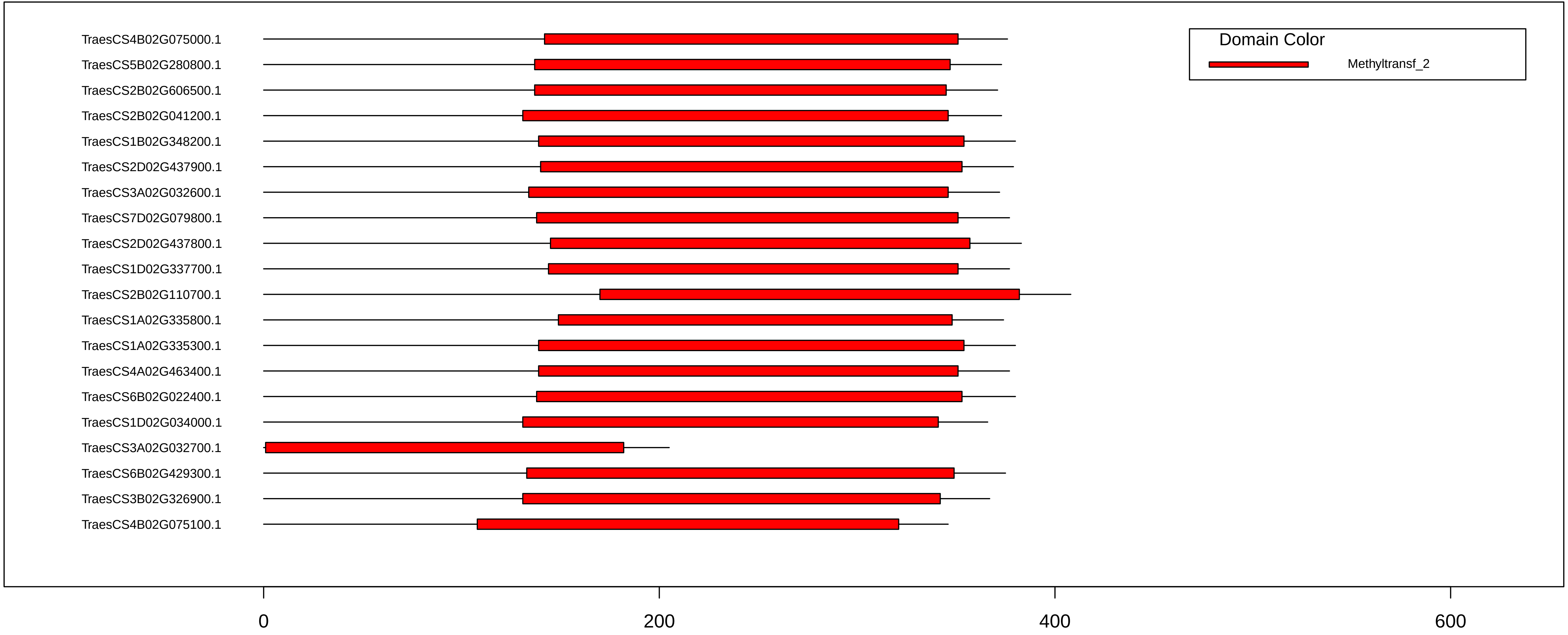

T.ae ASMT II subfamily domain diagram (part 5)

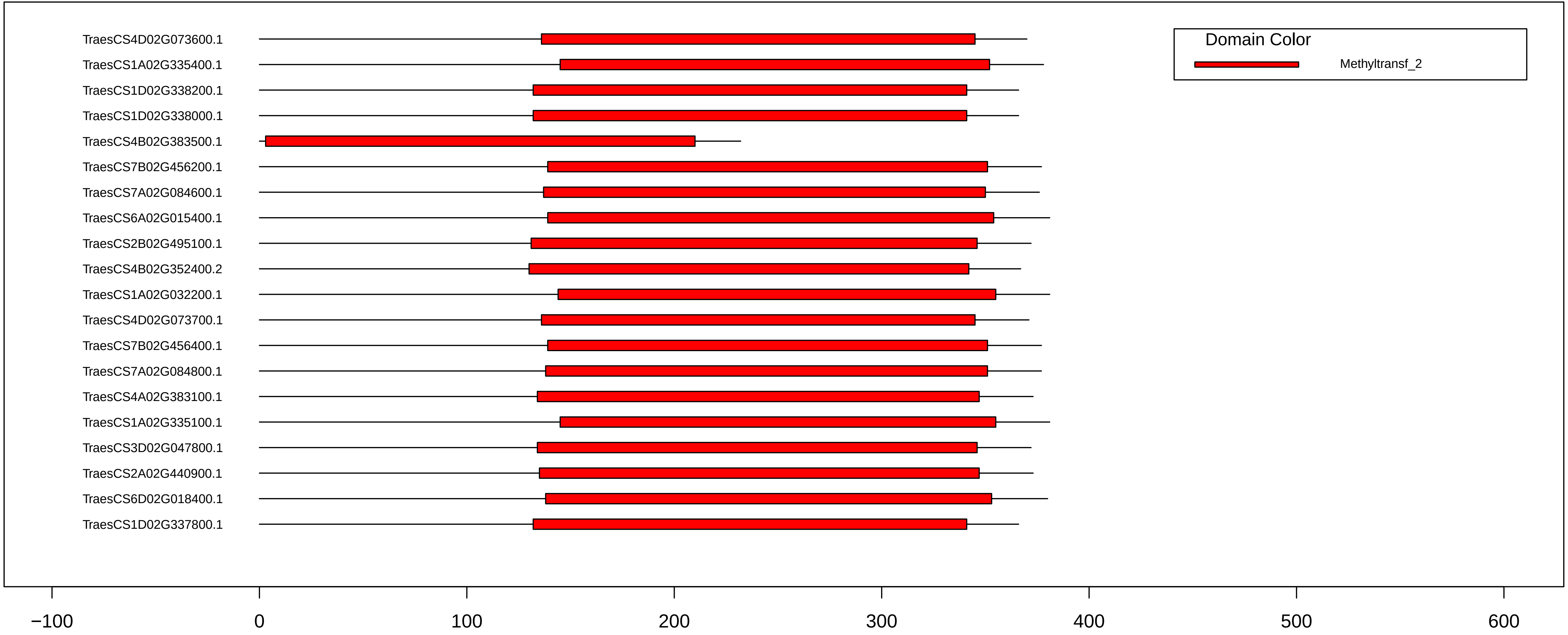

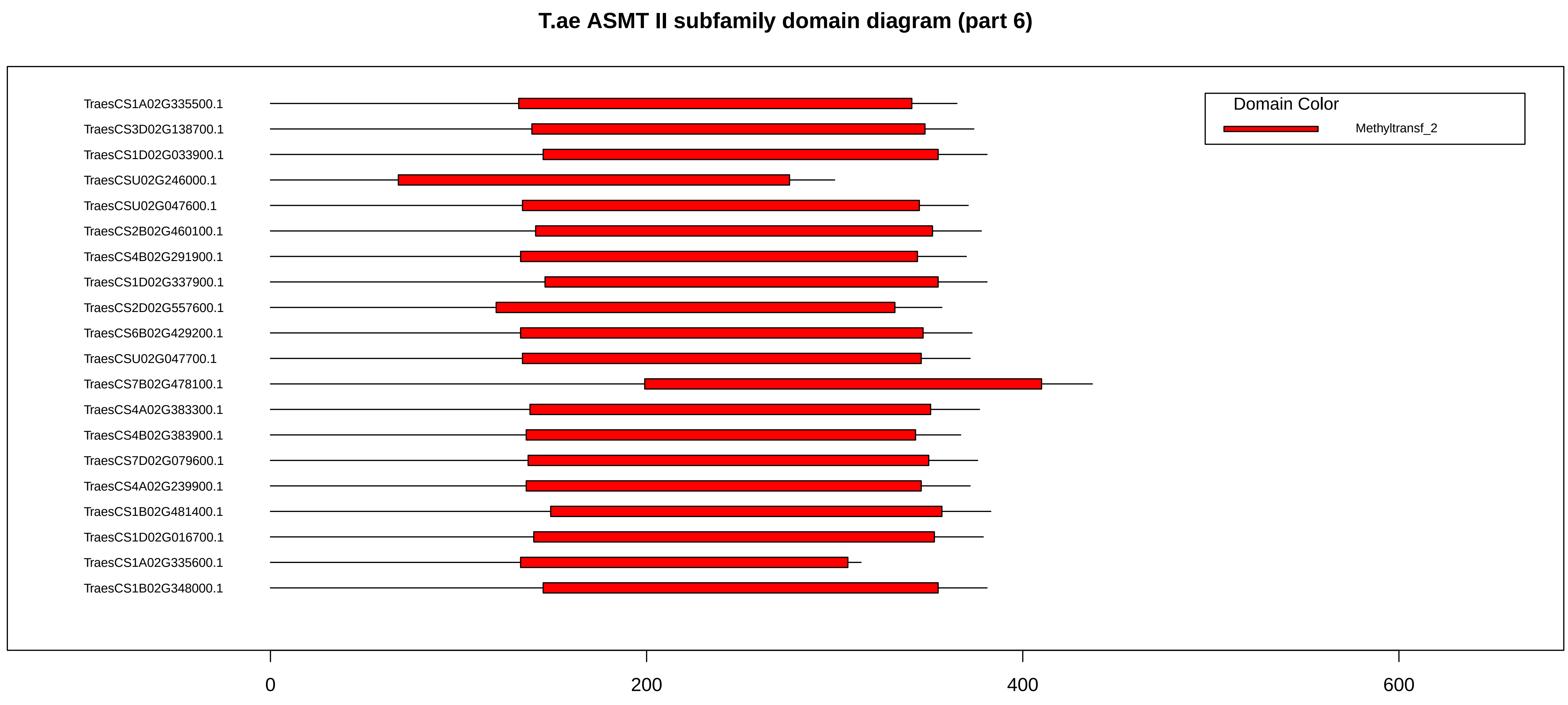
T.ae ASMT II subfamily domain diagram (part 6)
TraesCS1A02G335500.1
TraesCS3D02G138700.1
TraesCS1D02G033900.1
TraesCSU02G246000.1
TraesCSU02G047600.1
TraesCS2B02G460100.1
TraesCS4B02G291900.1
TraesCS1D02G337900.1
TraesCS2D02G557600.1
TraesCS6B02G429200.1
TraesCSU02G047700.1
TraesCS7B02G478100.1
TraesCS4A02G383300.1
TraesCS4B02G383900.1
TraesCS7D02G079600.1
TraesCS4A02G239900.1
TraesCS1B02G481400.1
TraesCS1D02G016700.1
TraesCS1A02G335600.1
TraesCS1B02G348000.1
Domain Color
Methyltransf_2
0
200
400
600

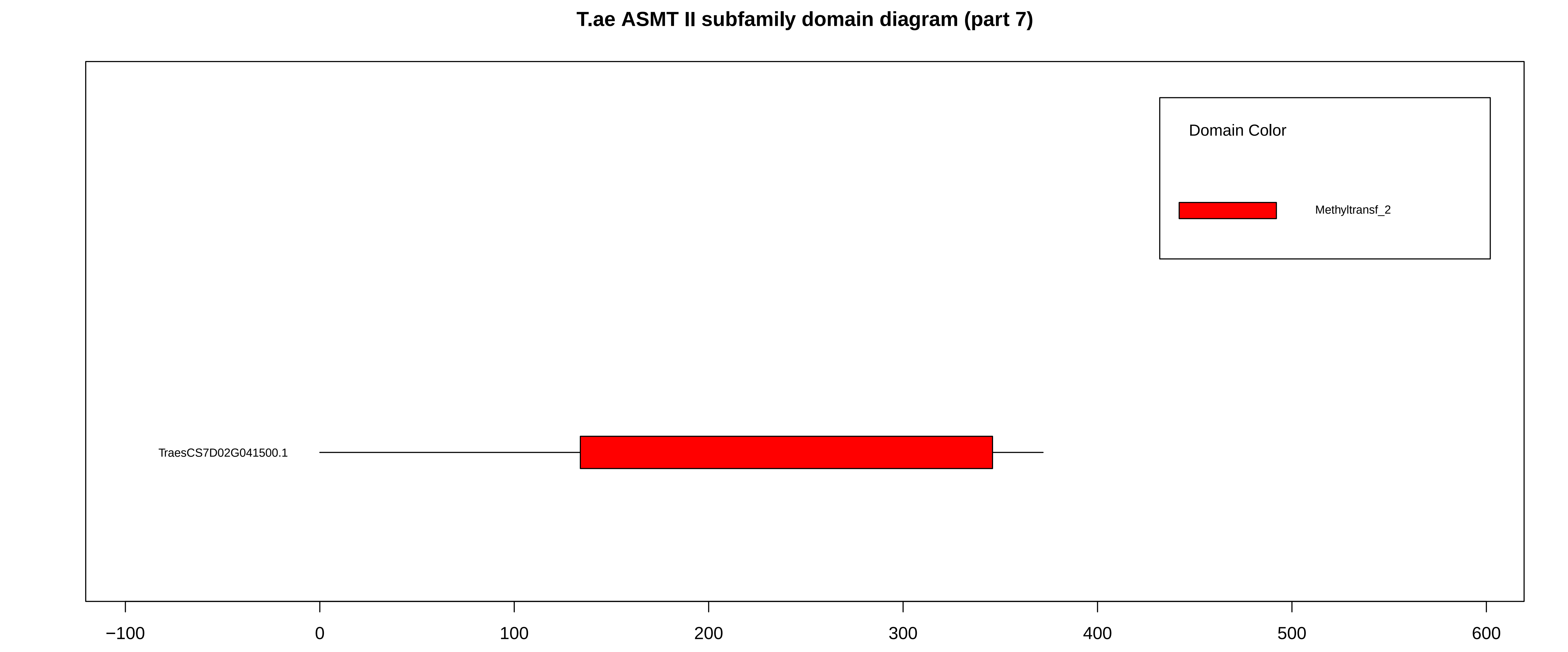
T.ae ASMT II subfamily domain diagram (part 7)
Domain Color
Methyltransf_2
TraesCS7D02G041500.1
−100
0
100
200
300
400
500
600

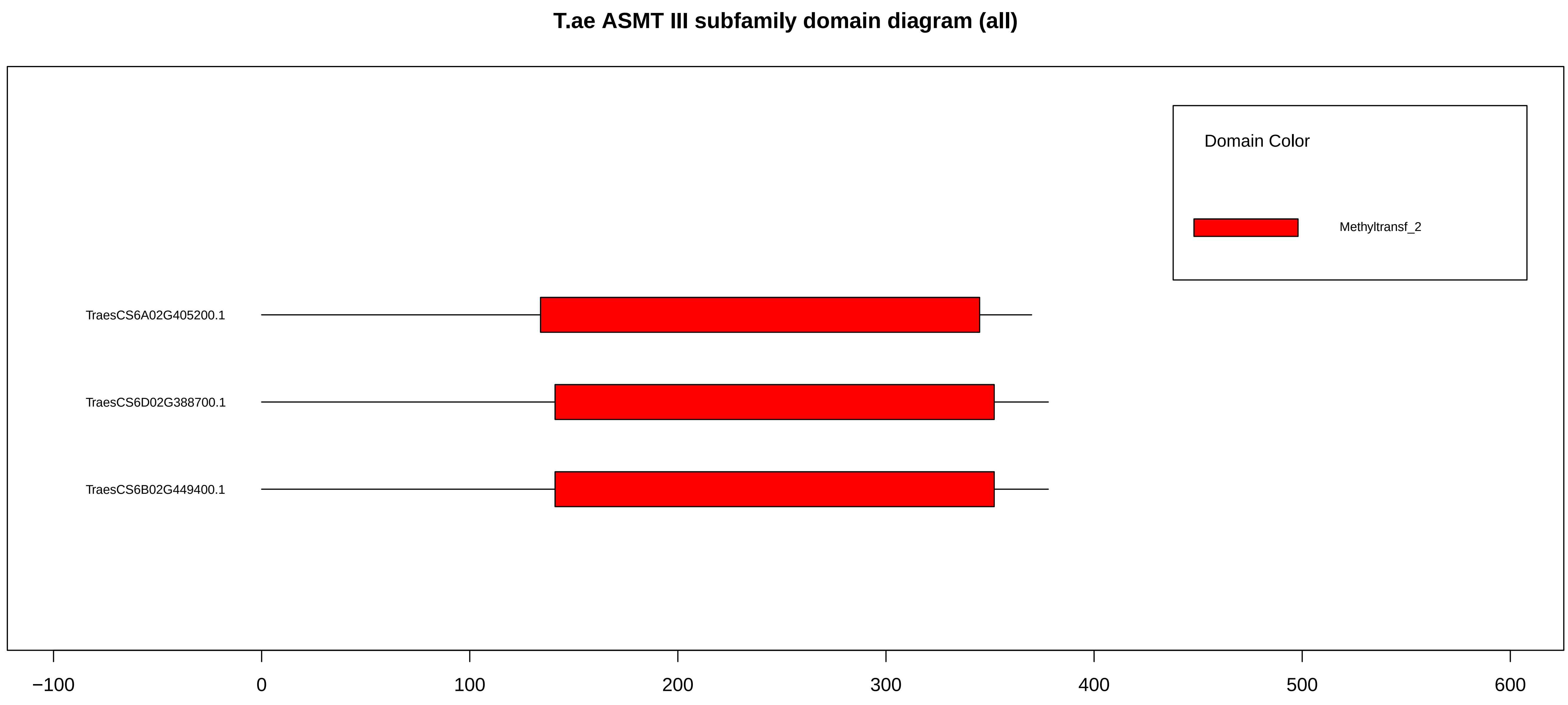

T.ae ASMT III subfamily domain diagram (all)
Domain Color
Methyltransf_2
TraesCS6A02G405200.1
TraesCS6D02G388700.1
TraesCS6B02G449400.1
−100
0
100
200
300
400
500
600

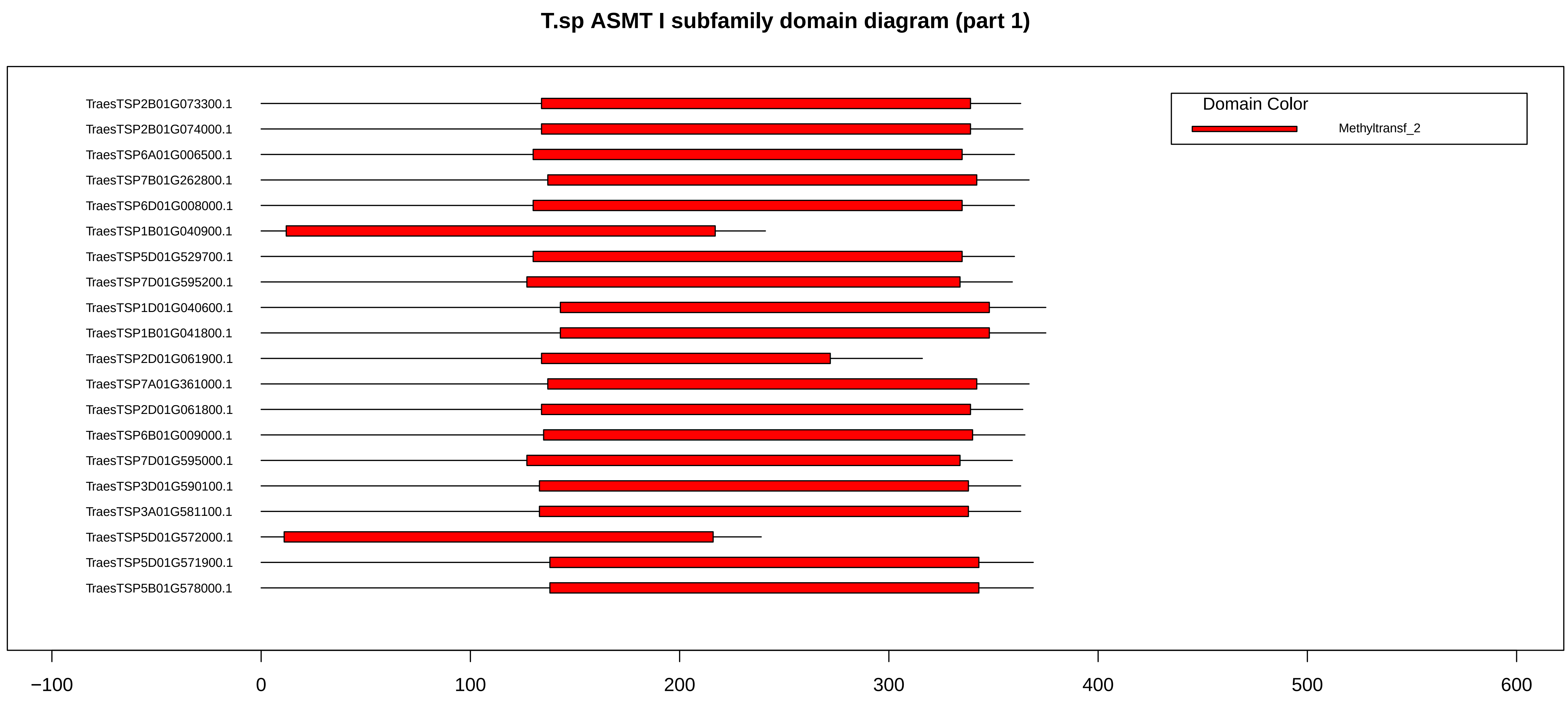
T.sp ASMT I subfamily domain diagram (part 1)
Domain Color
Methyltransf_2
TraesTSP2B01G073300.1
TraesTSP2B01G074000.1
TraesTSP6A01G006500.1
TraesTSP7B01G262800.1
TraesTSP6D01G008000.1
TraesTSP1B01G040900.1
TraesTSP5D01G529700.1
TraesTSP7D01G595200.1
TraesTSP1D01G040600.1
TraesTSP1B01G041800.1
TraesTSP2D01G061900.1
TraesTSP7A01G361000.1
TraesTSP2D01G061800.1
TraesTSP6B01G009000.1
TraesTSP7D01G595000.1
TraesTSP3D01G590100.1
TraesTSP3A01G581100.1
TraesTSP5D01G572000.1
TraesTSP5D01G571900.1
TraesTSP5B01G578000.1
−100
0
100
200
300
400
500
600

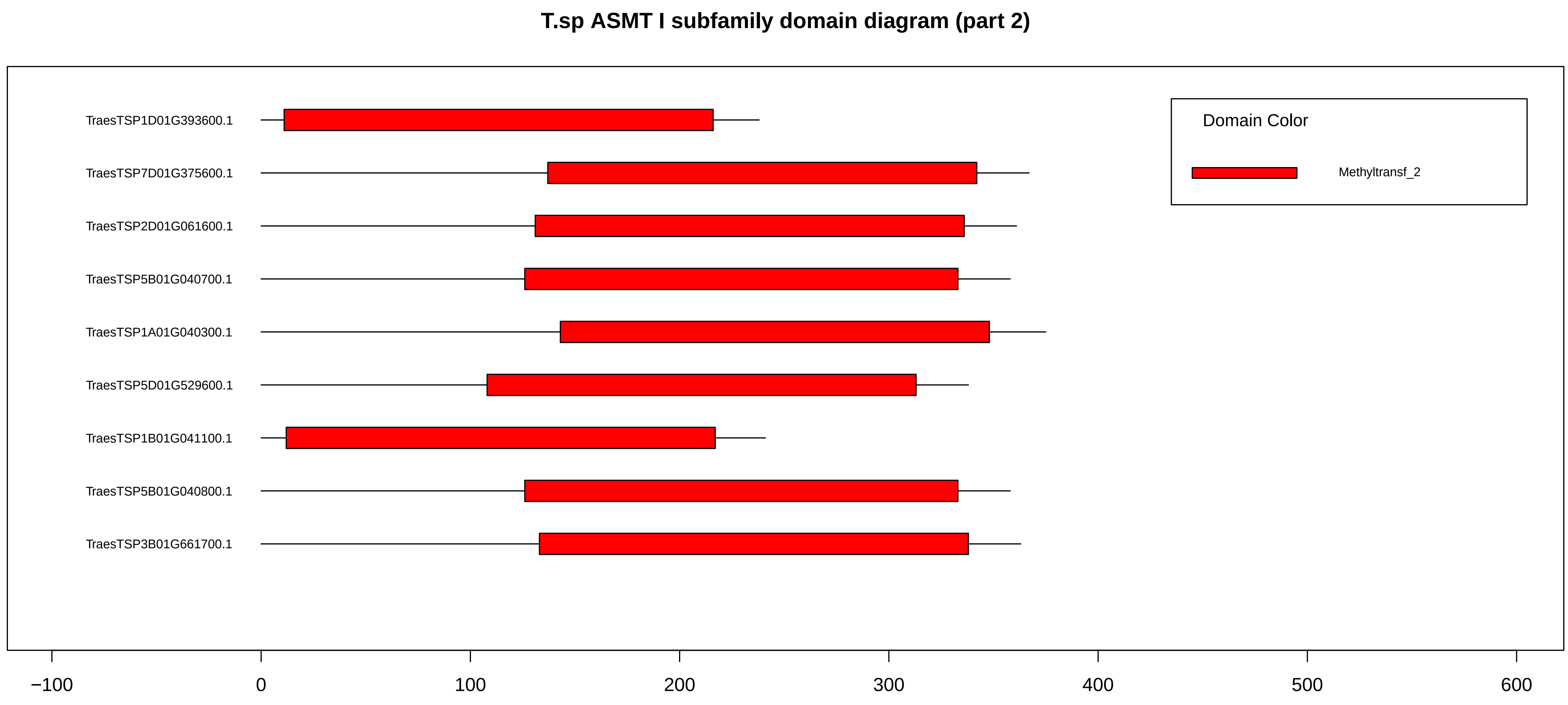
T.sp ASMT I subfamily domain diagram (part 2)
TraesTSP1D01G393600.1
TraesTSP7D01G375600.1
TraesTSP2D01G061600.1
TraesTSP5B01G040700.1
TraesTSP1A01G040300.1
TraesTSP5D01G529600.1
TraesTSP1B01G041100.1
TraesTSP5B01G040800.1
TraesTSP3B01G661700.1
Domain Color
Methyltransf_2
−100
0
100
200
300
400
500
600

T.sp ASMT II subfamily domain diagram (part 1)

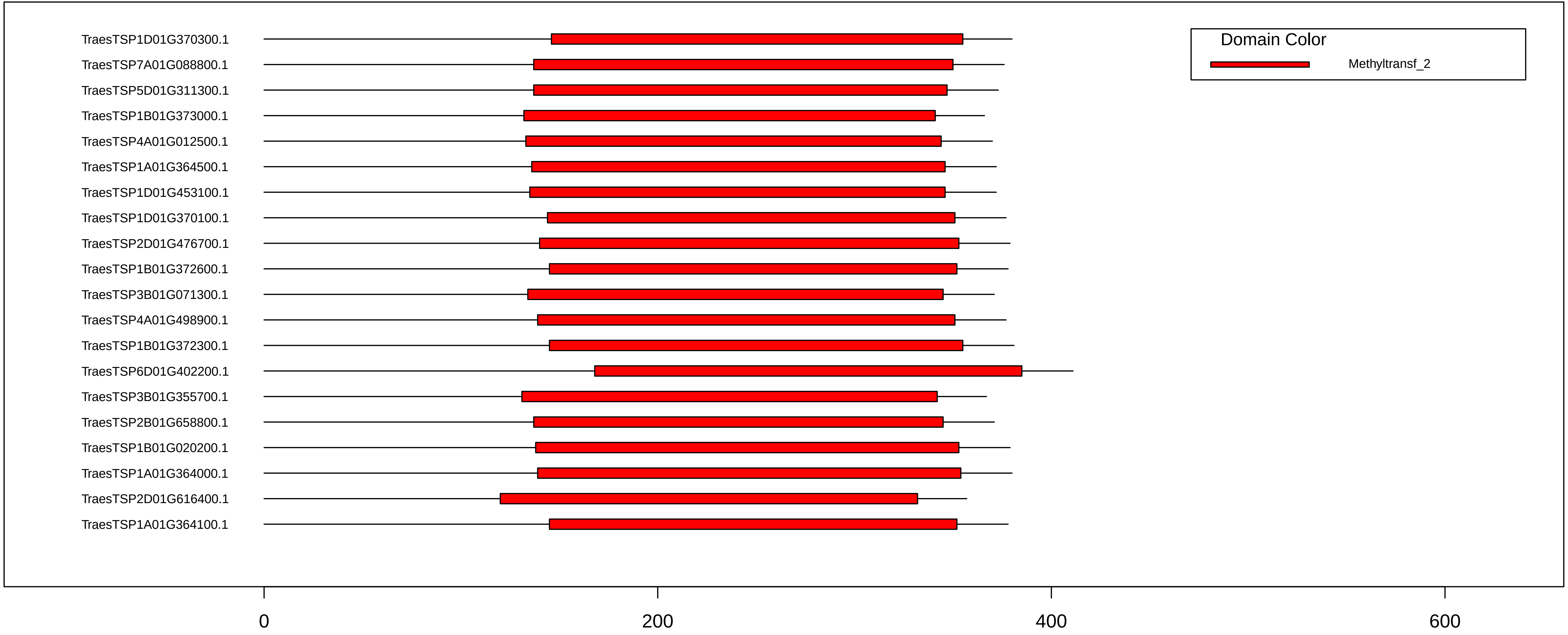

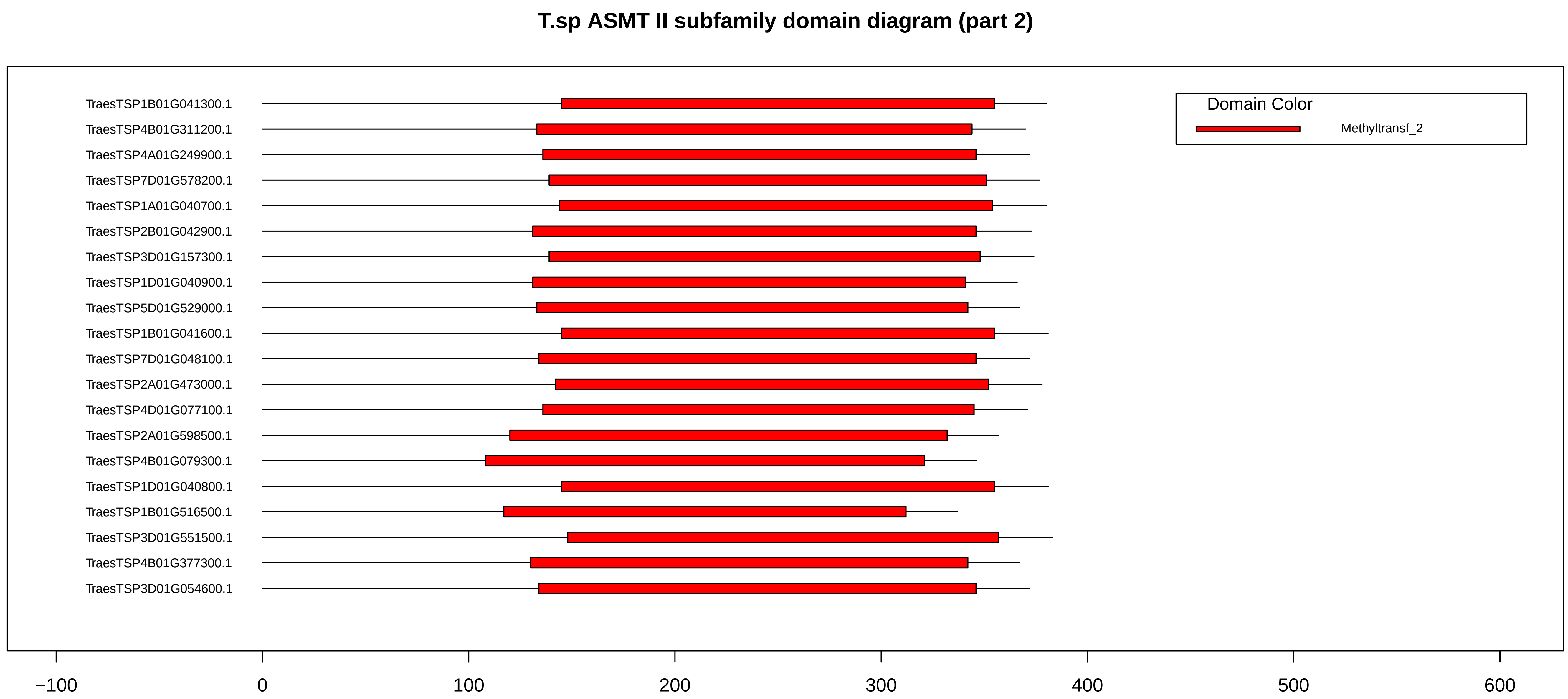
T.sp ASMT II subfamily domain diagram (part 2)
TraesTSP1B01G041300.1
TraesTSP4B01G311200.1
TraesTSP4A01G249900.1
TraesTSP7D01G578200.1
TraesTSP1A01G040700.1
TraesTSP2B01G042900.1
TraesTSP3D01G157300.1
TraesTSP1D01G040900.1
TraesTSP5D01G529000.1
TraesTSP1B01G041600.1
TraesTSP7D01G048100.1
TraesTSP2A01G473000.1
TraesTSP4D01G077100.1
TraesTSP2A01G598500.1
TraesTSP4B01G079300.1
TraesTSP1D01G040800.1
TraesTSP1B01G516500.1
TraesTSP3D01G551500.1
TraesTSP4B01G377300.1
TraesTSP3D01G054600.1
Domain Color
Methyltransf_2
−100
0
100
200
300
400
500
600

T.sp ASMT II subfamily domain diagram (part 3)

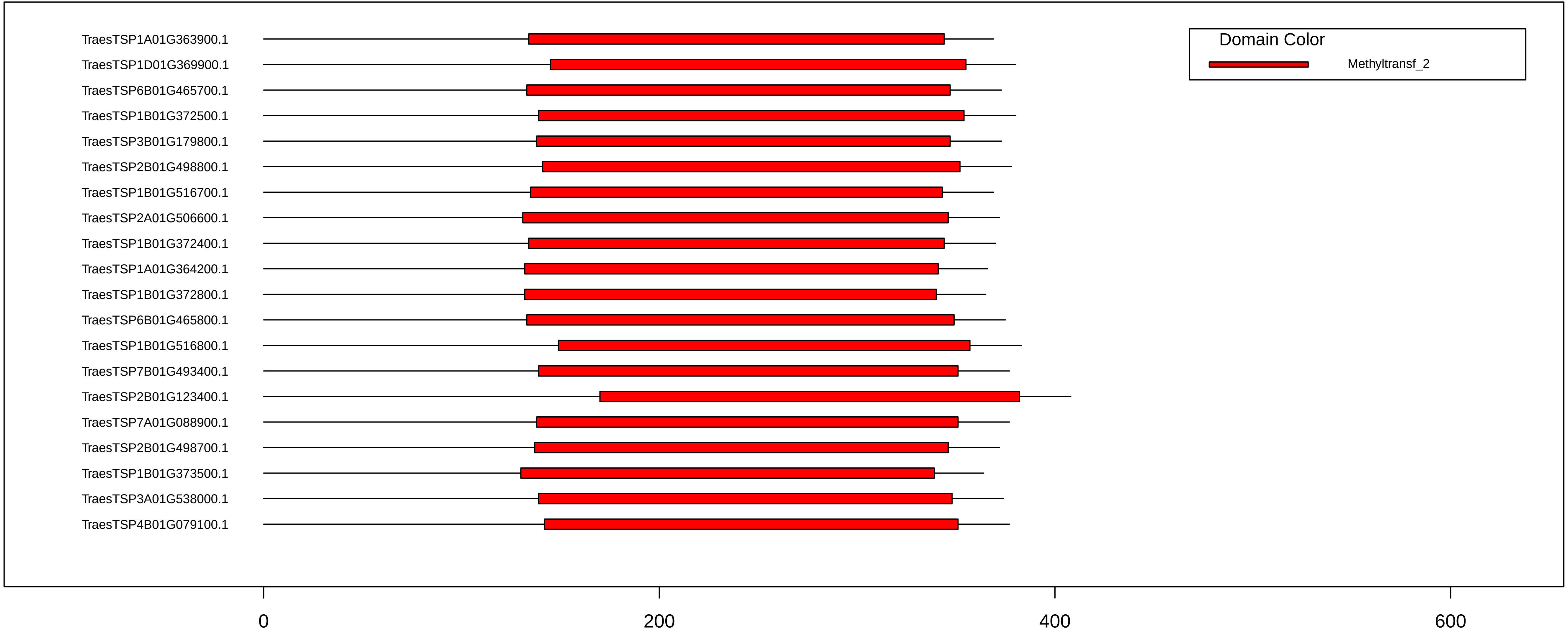

T.sp ASMT II subfamily domain diagram (part 4)

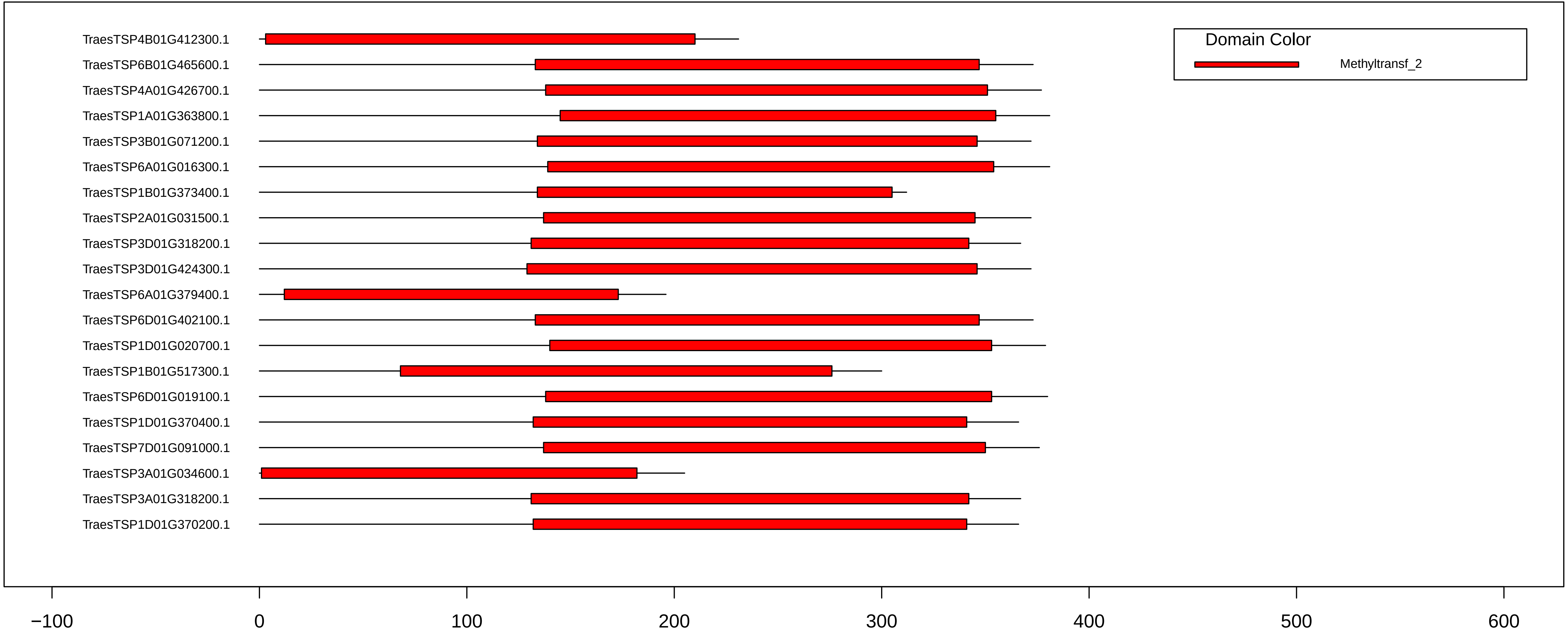

T.sp ASMT II subfamily domain diagram (part 5)

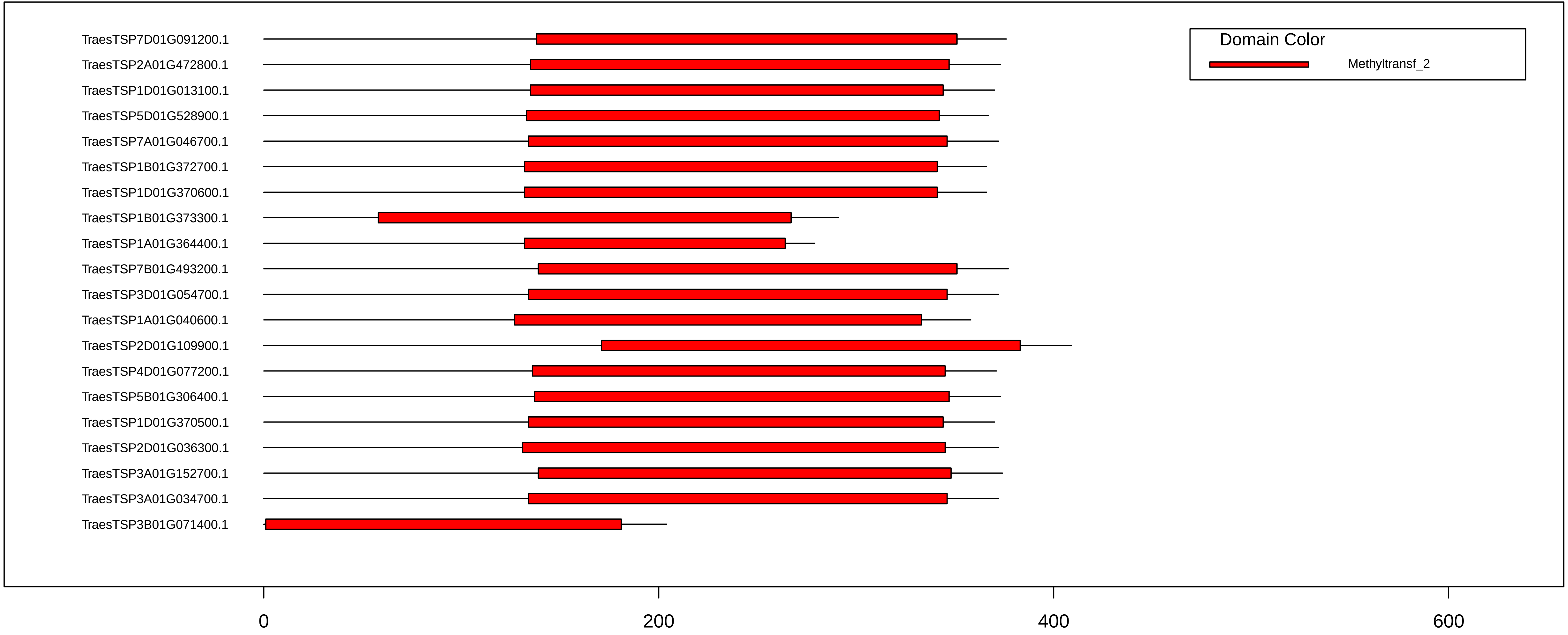

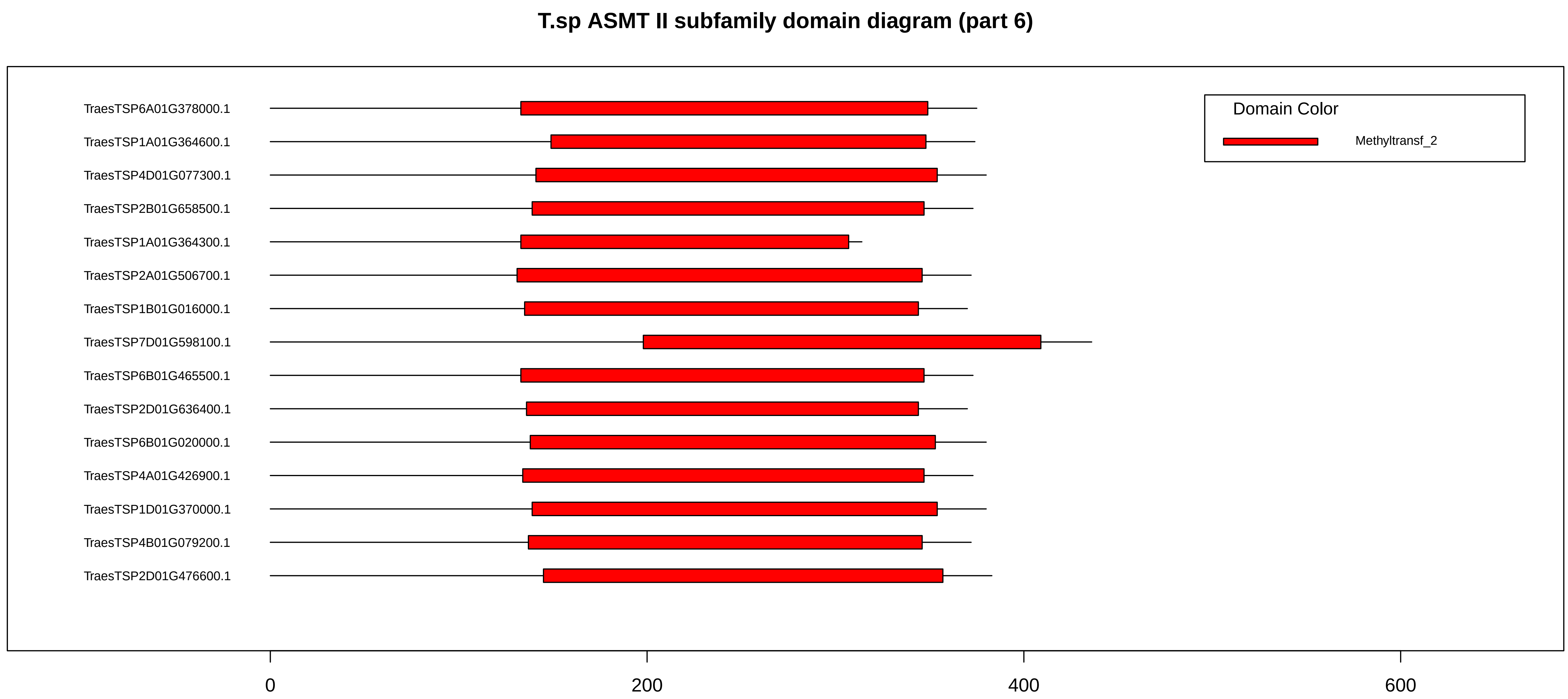
T.sp ASMT II subfamily domain diagram (part 6)
TraesTSP6A01G378000.1
TraesTSP1A01G364600.1
TraesTSP4D01G077300.1
TraesTSP2B01G658500.1
TraesTSP1A01G364300.1
TraesTSP2A01G506700.1
TraesTSP1B01G016000.1
TraesTSP7D01G598100.1
TraesTSP6B01G465500.1
TraesTSP2D01G636400.1
TraesTSP6B01G020000.1
TraesTSP4A01G426900.1
TraesTSP1D01G370000.1
TraesTSP4B01G079200.1
TraesTSP2D01G476600.1
Domain Color
Methyltransf_2
0
200
400
600

T.sp ASMT III subfamily domain diagram (all)

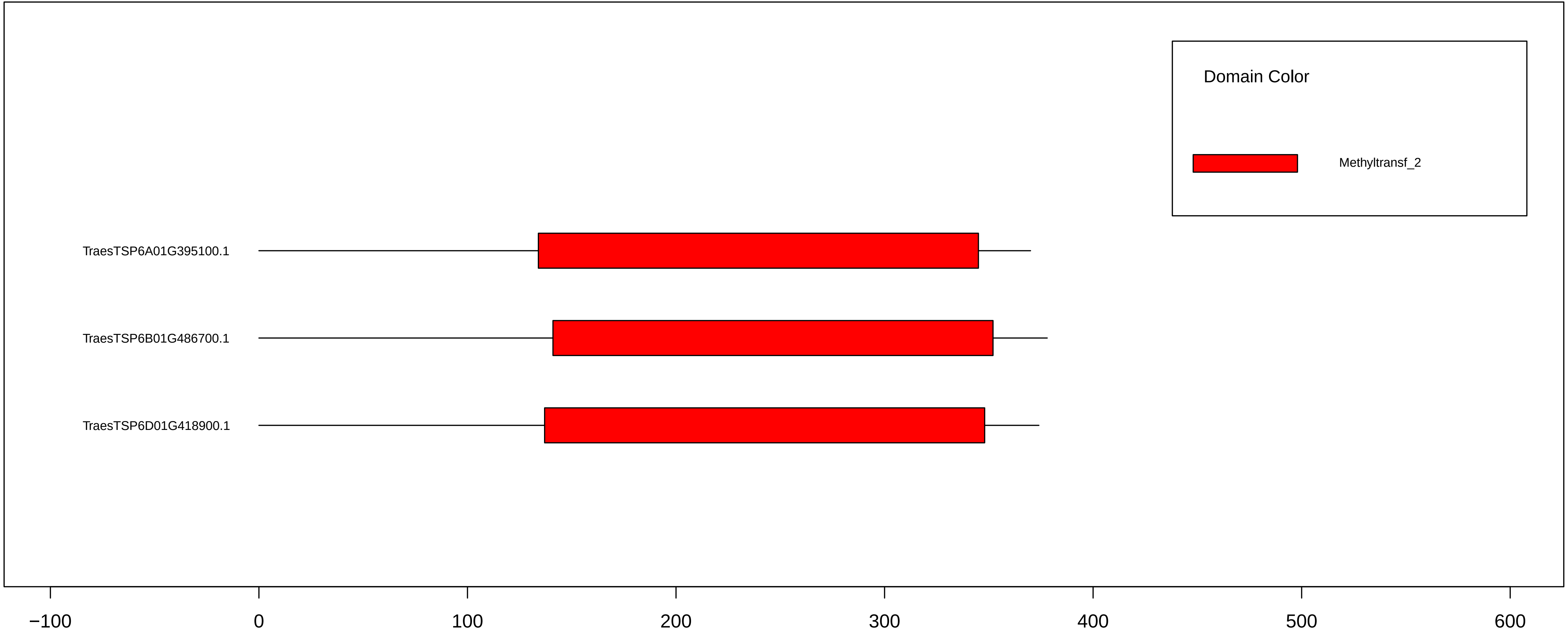

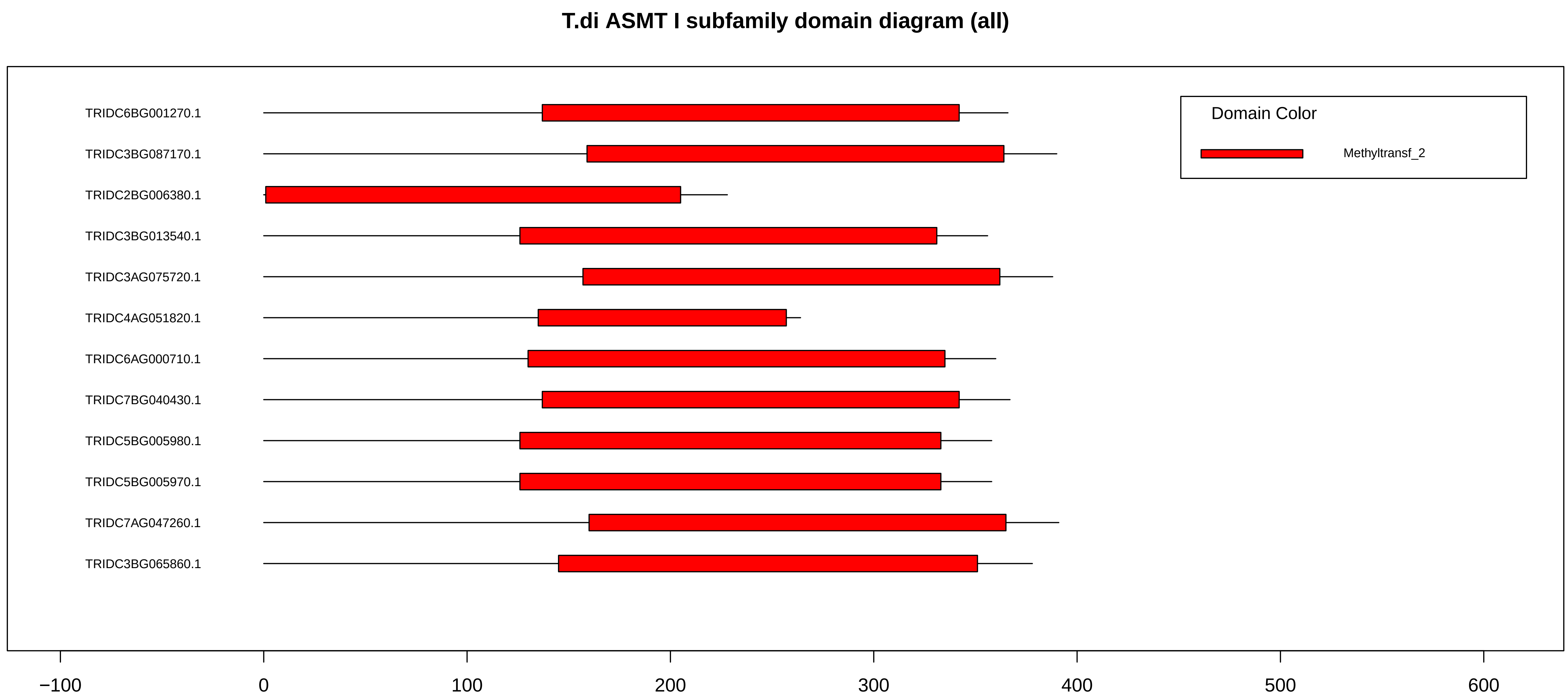
T.di ASMT I subfamily domain diagram (all)
TRIDC6BG001270.1
TRIDC3BG087170.1
TRIDC2BG006380.1
TRIDC3BG013540.1
TRIDC3AG075720.1
TRIDC4AG051820.1
TRIDC6AG000710.1
TRIDC7BG040430.1
TRIDC5BG005980.1
TRIDC5BG005970.1
TRIDC7AG047260.1
TRIDC3BG065860.1
Domain Color
Methyltransf_2
−100
0
100
200
300
400
500
600

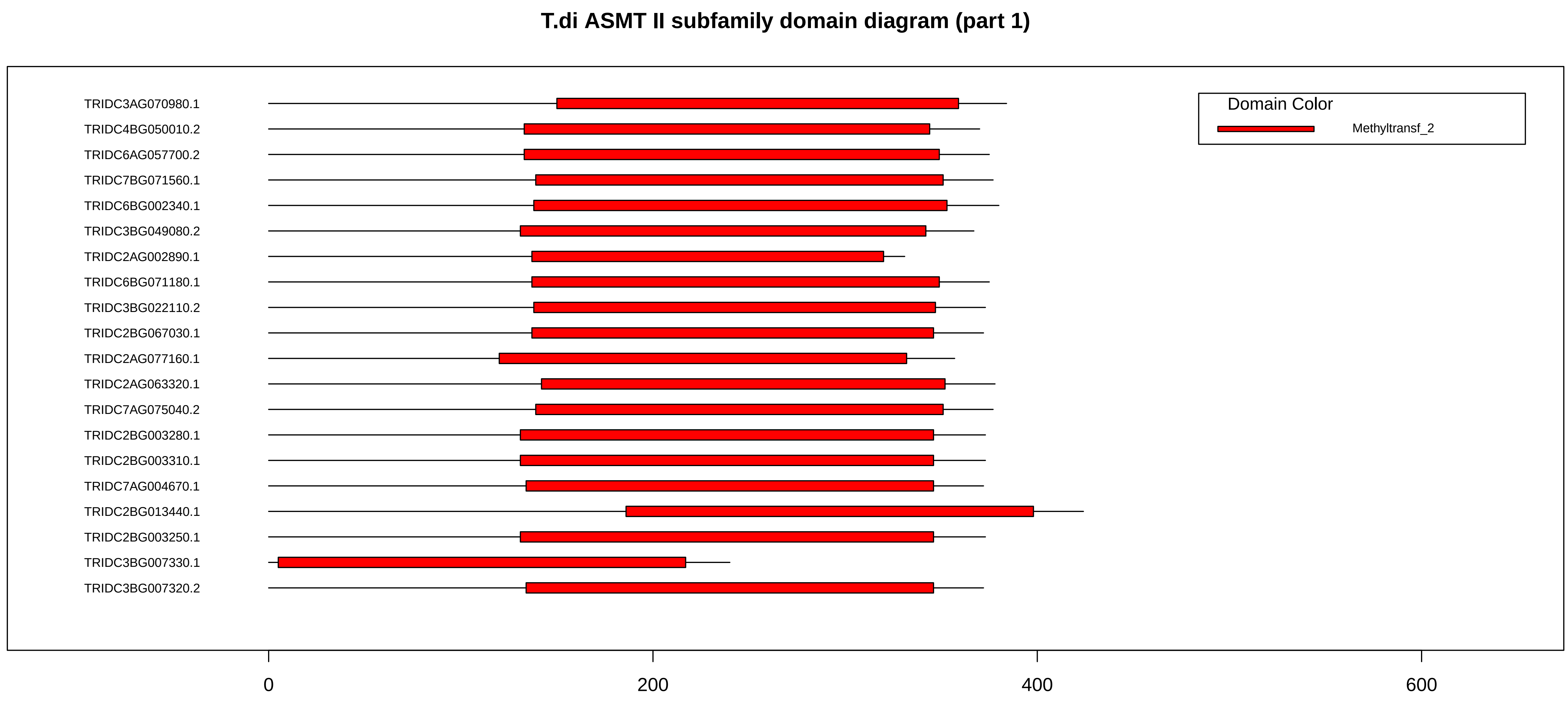

T.di ASMT II subfamily domain diagram (part 1)
TRIDC3AG070980.1
TRIDC4BG050010.2
TRIDC6AG057700.2
TRIDC7BG071560.1
TRIDC6BG002340.1
TRIDC3BG049080.2
TRIDC2AG002890.1
TRIDC6BG071180.1
TRIDC3BG022110.2
TRIDC2BG067030.1
TRIDC2AG077160.1
TRIDC2AG063320.1
TRIDC7AG075040.2
TRIDC2BG003280.1
TRIDC2BG003310.1
TRIDC7AG004670.1
TRIDC2BG013440.1
TRIDC2BG003250.1
TRIDC3BG007330.1
TRIDC3BG007320.2
Domain Color
Methyltransf_2
0
200
400
600

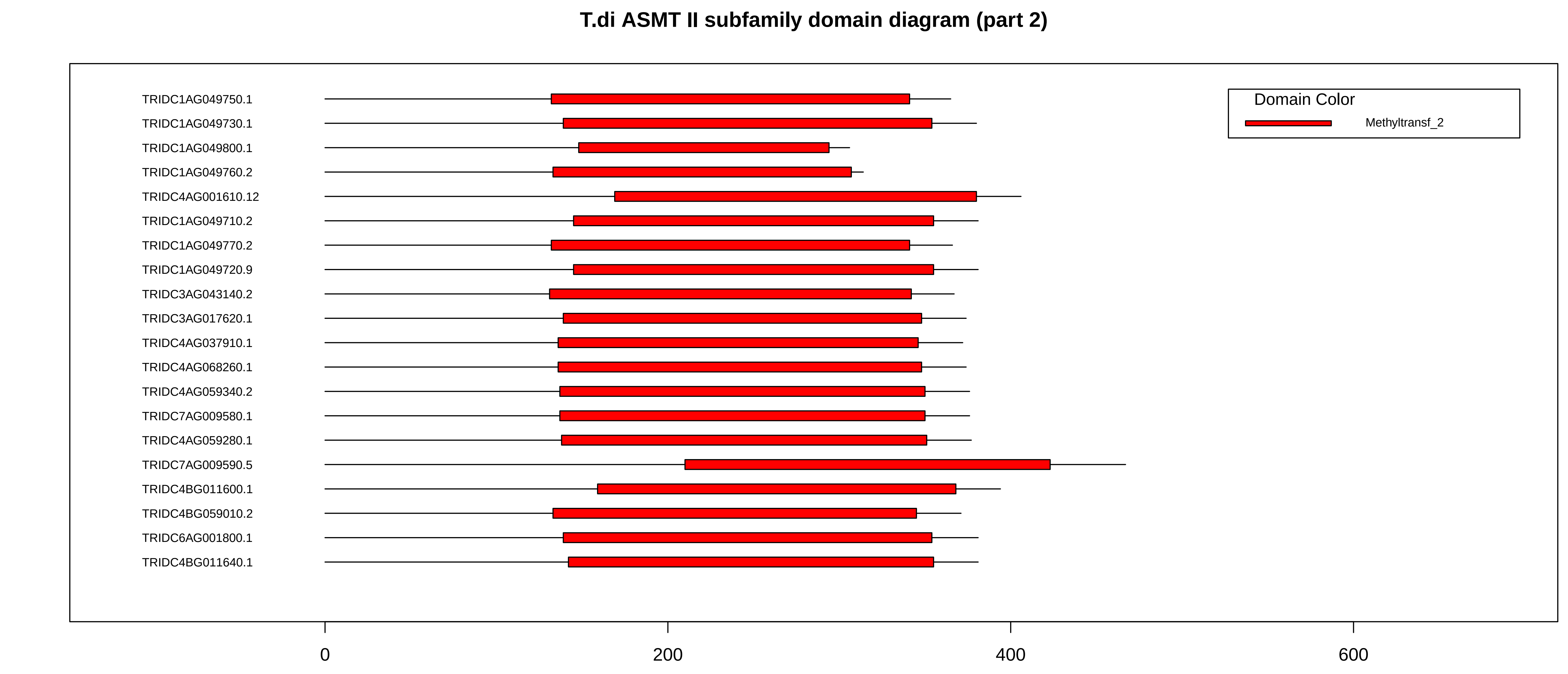
T.di ASMT II subfamily domain diagram (part 2)
TRIDC1AG049750.1
TRIDC1AG049730.1
TRIDC1AG049800.1
TRIDC1AG049760.2
TRIDC4AG001610.12
TRIDC1AG049710.2
TRIDC1AG049770.2
TRIDC1AG049720.9
TRIDC3AG043140.2
TRIDC3AG017620.1
TRIDC4AG037910.1
TRIDC4AG068260.1
TRIDC4AG059340.2
TRIDC7AG009580.1
TRIDC4AG059280.1
TRIDC7AG009590.5
TRIDC4BG011600.1
TRIDC4BG059010.2
TRIDC6AG001800.1
TRIDC4BG011640.1
Domain Color
Methyltransf_2
0
200
400
600

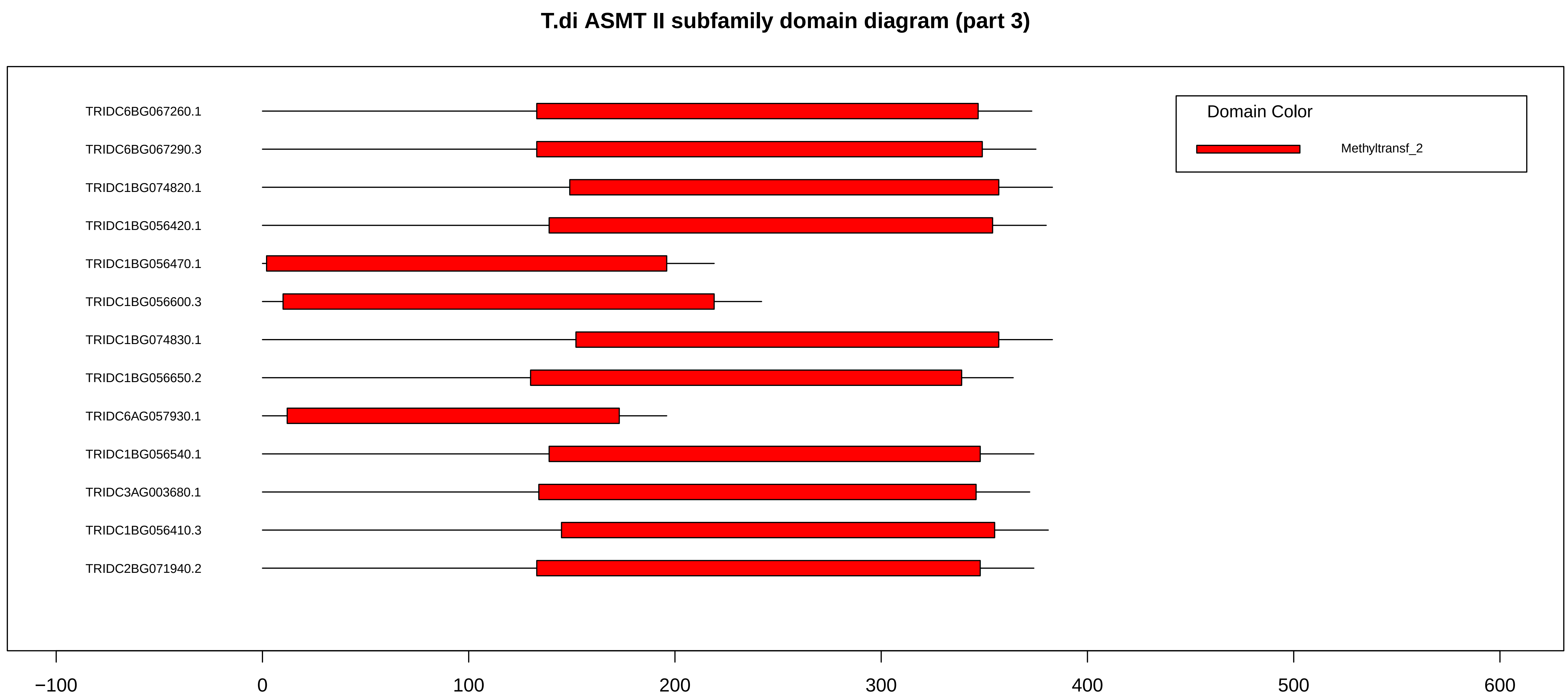

T.di ASMT II subfamily domain diagram (part 3)
TRIDC6BG067260.1
TRIDC6BG067290.3
TRIDC1BG074820.1
TRIDC1BG056420.1
TRIDC1BG056470.1
TRIDC1BG056600.3
TRIDC1BG074830.1
TRIDC1BG056650.2
TRIDC6AG057930.1
TRIDC1BG056540.1
TRIDC3AG003680.1
TRIDC1BG056410.3
TRIDC2BG071940.2
Domain Color
Methyltransf_2
−100
0
100
200
300
400
500
600

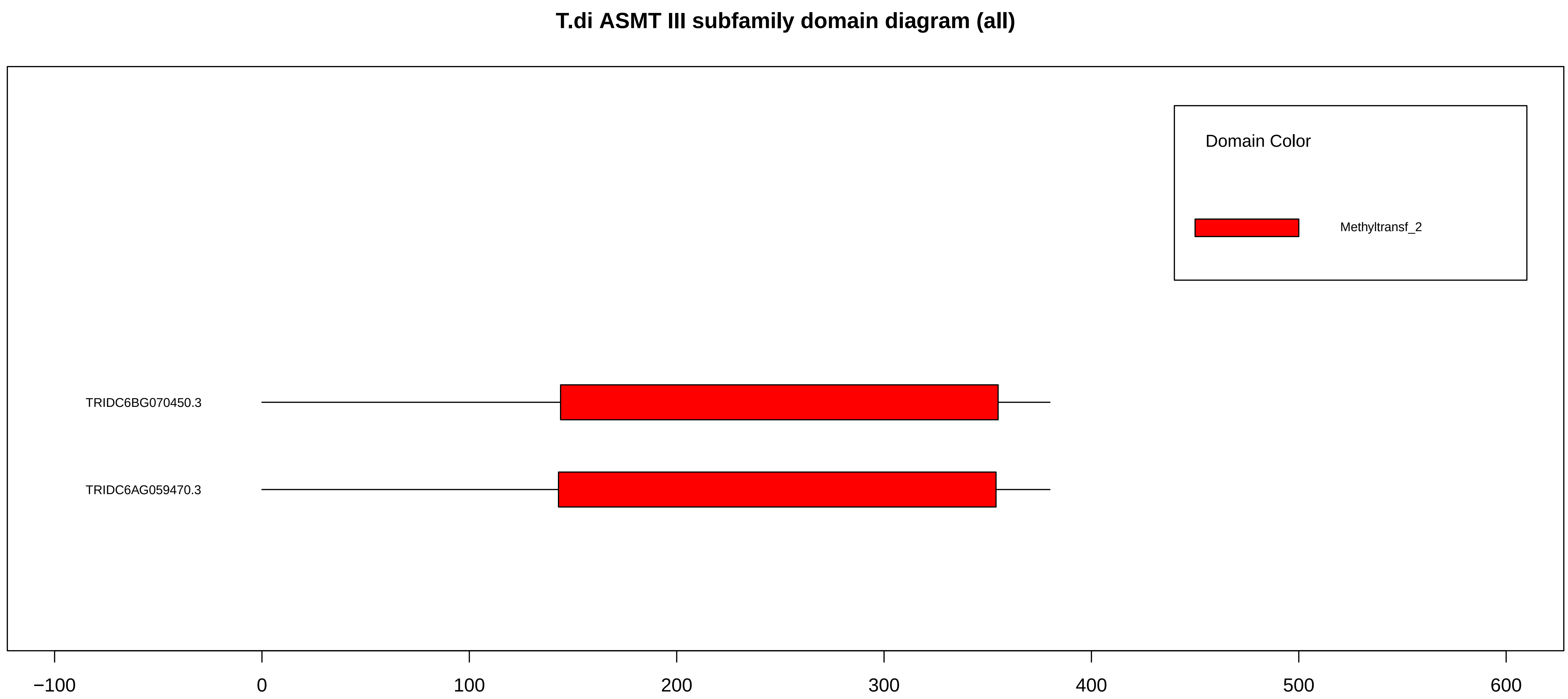
T.di ASMT III subfamily domain diagram (all)
Domain Color
Methyltransf_2
TRIDC6BG070450.3
TRIDC6AG059470.3
−100
0
100
200
300
400
500
600

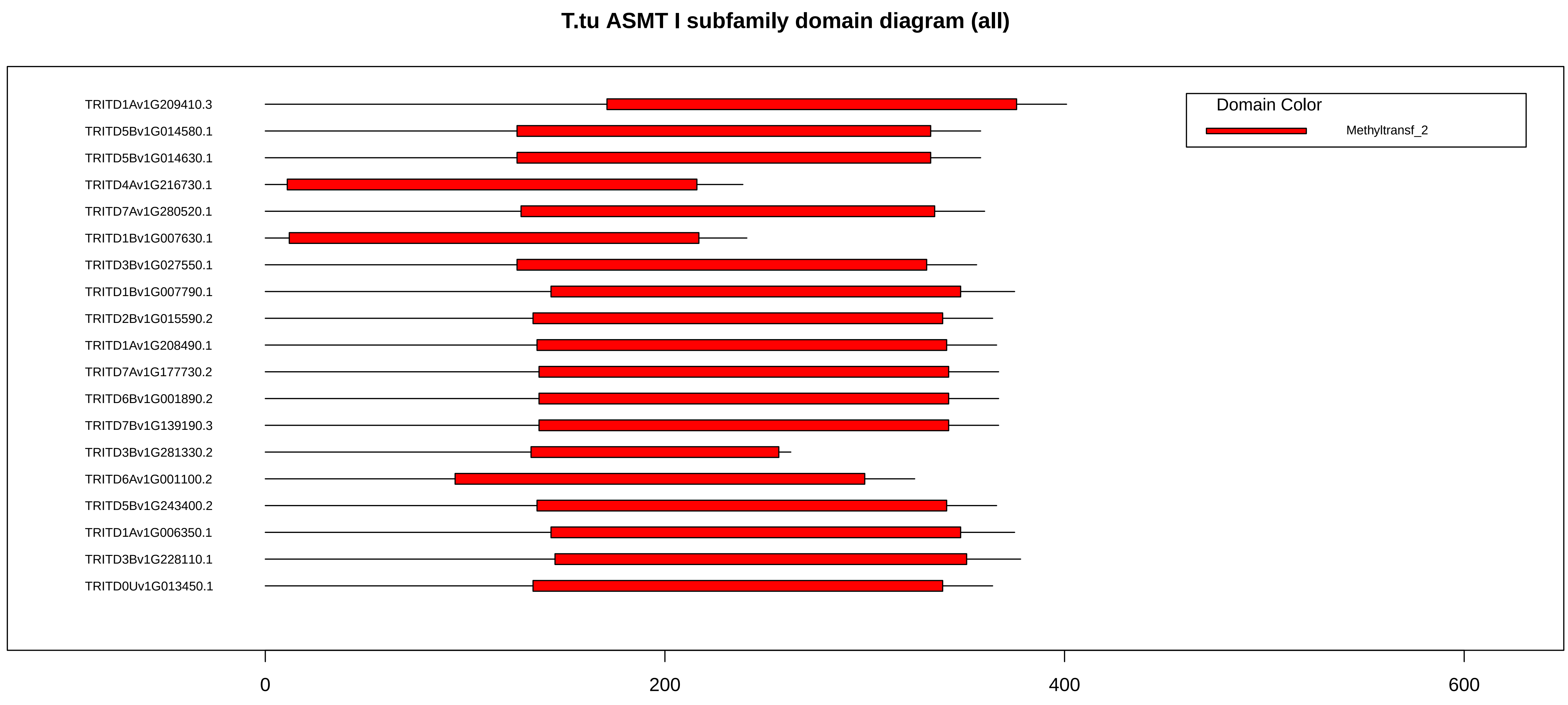
T.tu ASMT I subfamily domain diagram (all)
TRITD1Av1G209410.3
TRITD5Bv1G014580.1
TRITD5Bv1G014630.1
TRITD4Av1G216730.1
TRITD7Av1G280520.1
TRITD1Bv1G007630.1
TRITD3Bv1G027550.1
TRITD1Bv1G007790.1
TRITD2Bv1G015590.2
TRITD1Av1G208490.1
TRITD7Av1G177730.2
TRITD6Bv1G001890.2
TRITD7Bv1G139190.3
TRITD3Bv1G281330.2
TRITD6Av1G001100.2
TRITD5Bv1G243400.2
TRITD1Av1G006350.1
TRITD3Bv1G228110.1
TRITD0Uv1G013450.1
Domain Color
Methyltransf_2
0
200
400
600

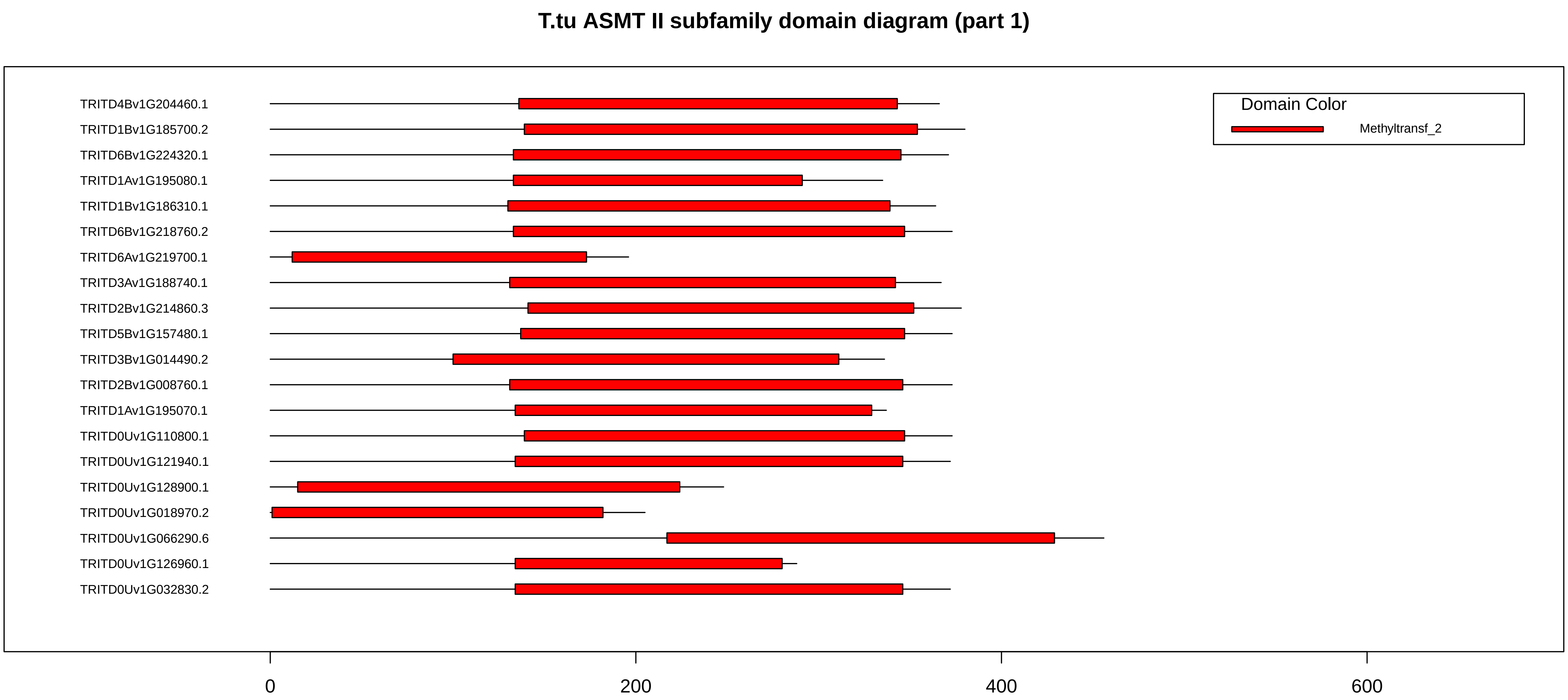
T.tu ASMT II subfamily domain diagram (part 1)
TRITD4Bv1G204460.1
TRITD1Bv1G185700.2
TRITD6Bv1G224320.1
TRITD1Av1G195080.1
TRITD1Bv1G186310.1
TRITD6Bv1G218760.2
TRITD6Av1G219700.1
TRITD3Av1G188740.1
TRITD2Bv1G214860.3
TRITD5Bv1G157480.1
TRITD3Bv1G014490.2
TRITD2Bv1G008760.1
TRITD1Av1G195070.1
TRITD0Uv1G110800.1
TRITD0Uv1G121940.1
TRITD0Uv1G128900.1
TRITD0Uv1G018970.2
TRITD0Uv1G066290.6
TRITD0Uv1G126960.1
TRITD0Uv1G032830.2
Domain Color
Methyltransf_2
0
200
400
600

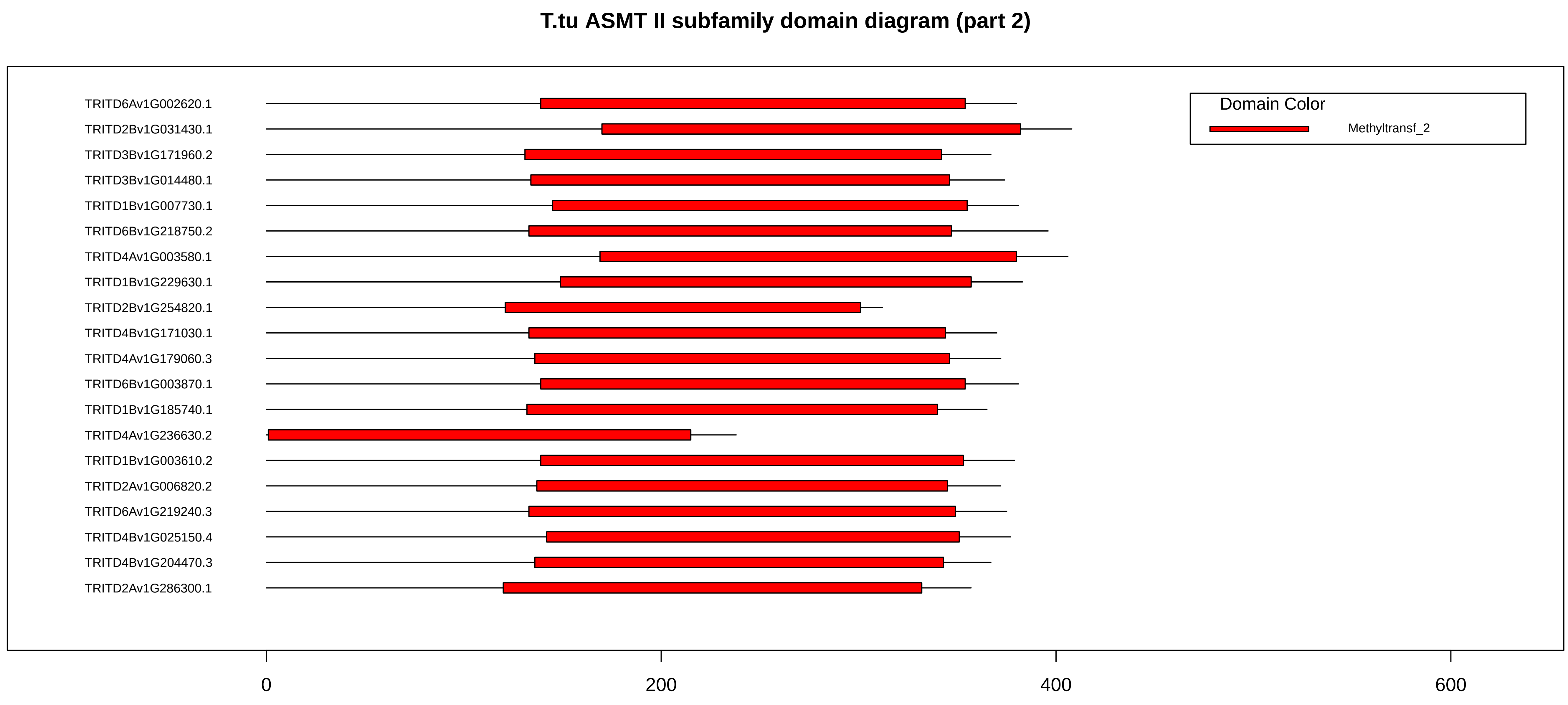

T.tu ASMT II subfamily domain diagram (part 2)
TRITD6Av1G002620.1
TRITD2Bv1G031430.1
TRITD3Bv1G171960.2
TRITD3Bv1G014480.1
TRITD1Bv1G007730.1
TRITD6Bv1G218750.2
TRITD4Av1G003580.1
TRITD1Bv1G229630.1
TRITD2Bv1G254820.1
TRITD4Bv1G171030.1
TRITD4Av1G179060.3
TRITD6Bv1G003870.1
TRITD1Bv1G185740.1
TRITD4Av1G236630.2
TRITD1Bv1G003610.2
TRITD2Av1G006820.2
TRITD6Av1G219240.3
TRITD4Bv1G025150.4
TRITD4Bv1G204470.3
TRITD2Av1G286300.1
Domain Color
Methyltransf_2
0
200
400
600

T.tu ASMT II subfamily domain diagram (part 3)

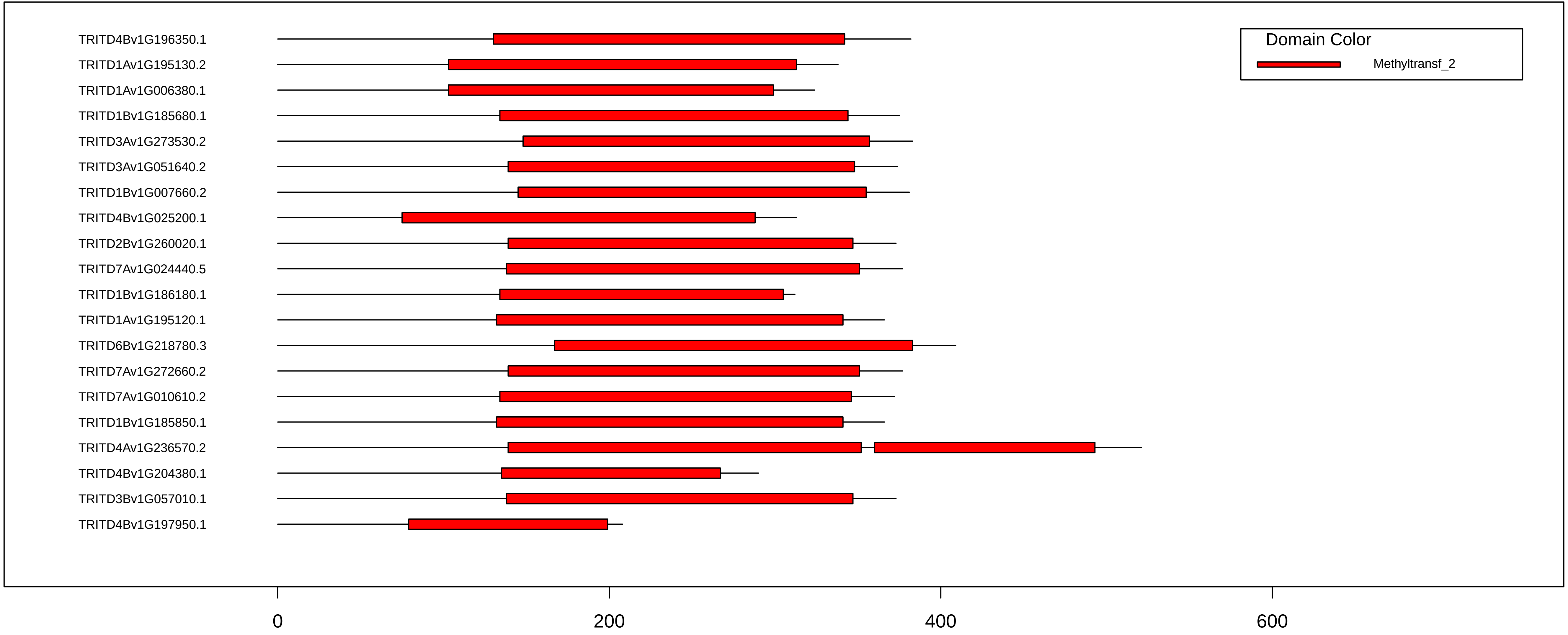

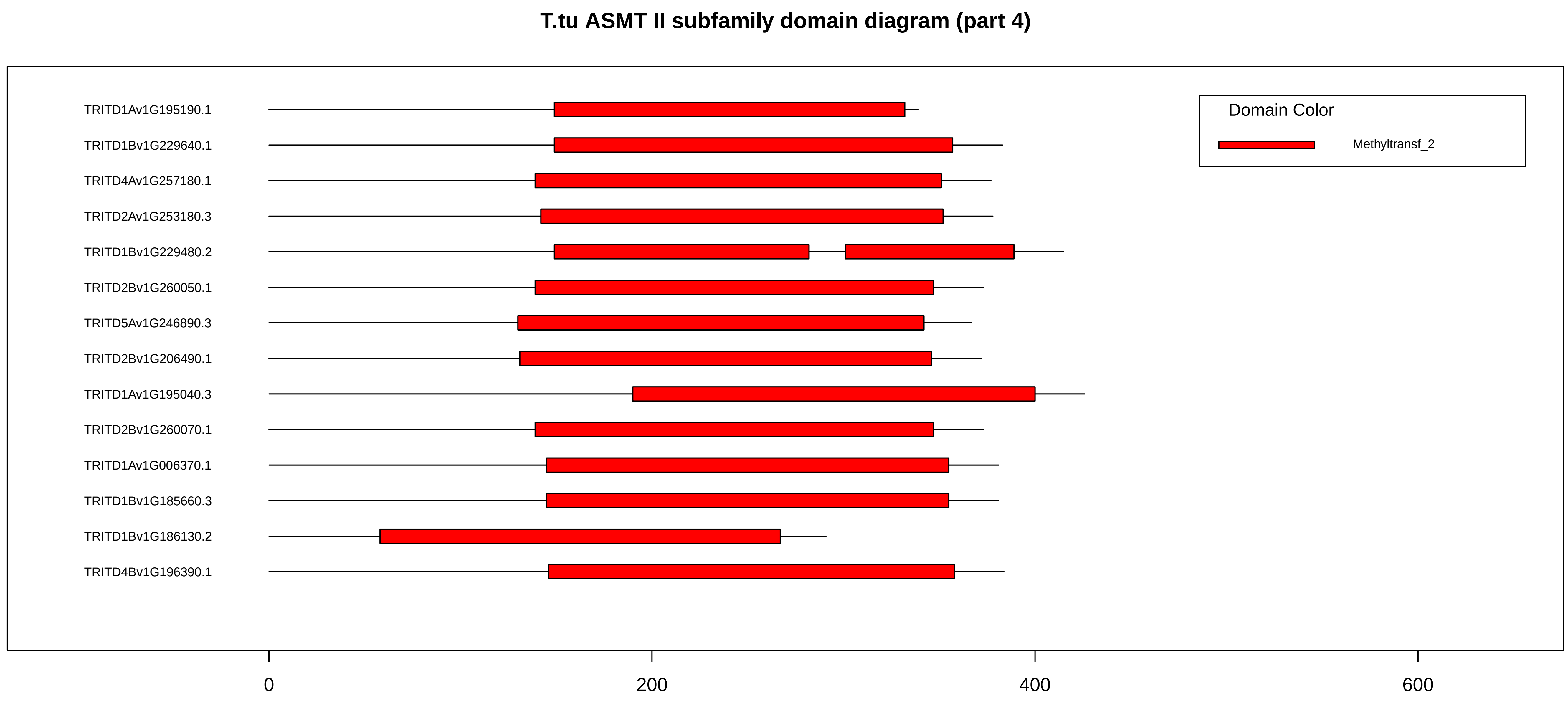
T.tu ASMT II subfamily domain diagram (part 4)
TRITD1Av1G195190.1
TRITD1Bv1G229640.1
TRITD4Av1G257180.1
TRITD2Av1G253180.3
TRITD1Bv1G229480.2
TRITD2Bv1G260050.1
TRITD5Av1G246890.3
TRITD2Bv1G206490.1
TRITD1Av1G195040.3
TRITD2Bv1G260070.1
TRITD1Av1G006370.1
TRITD1Bv1G185660.3
TRITD1Bv1G186130.2
TRITD4Bv1G196390.1
Domain Color
Methyltransf_2
0
200
400
600

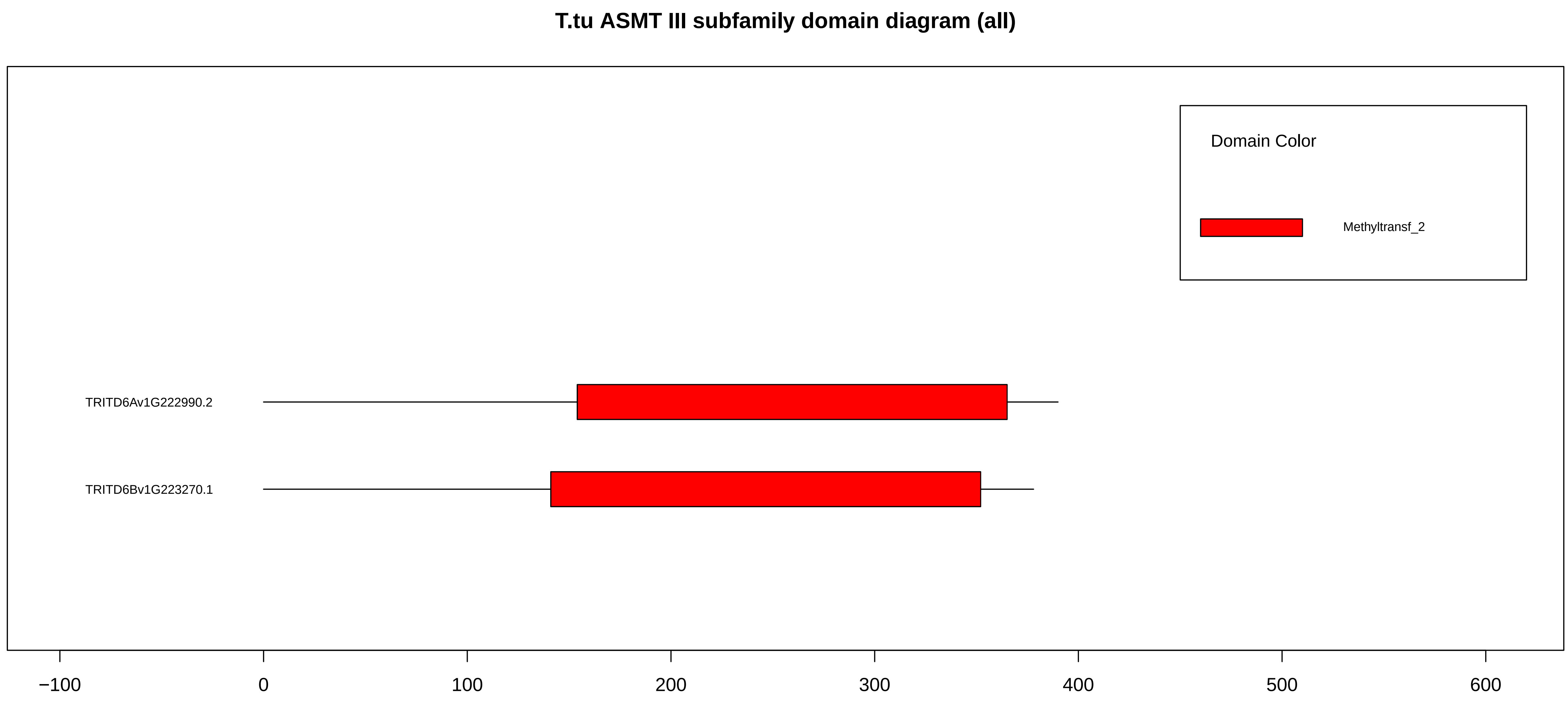
T.tu ASMT III subfamily domain diagram (all)
Domain Color
Methyltransf_2
TRITD6Av1G222990.2
TRITD6Bv1G223270.1
−100
0
100
200
300
400
500
600

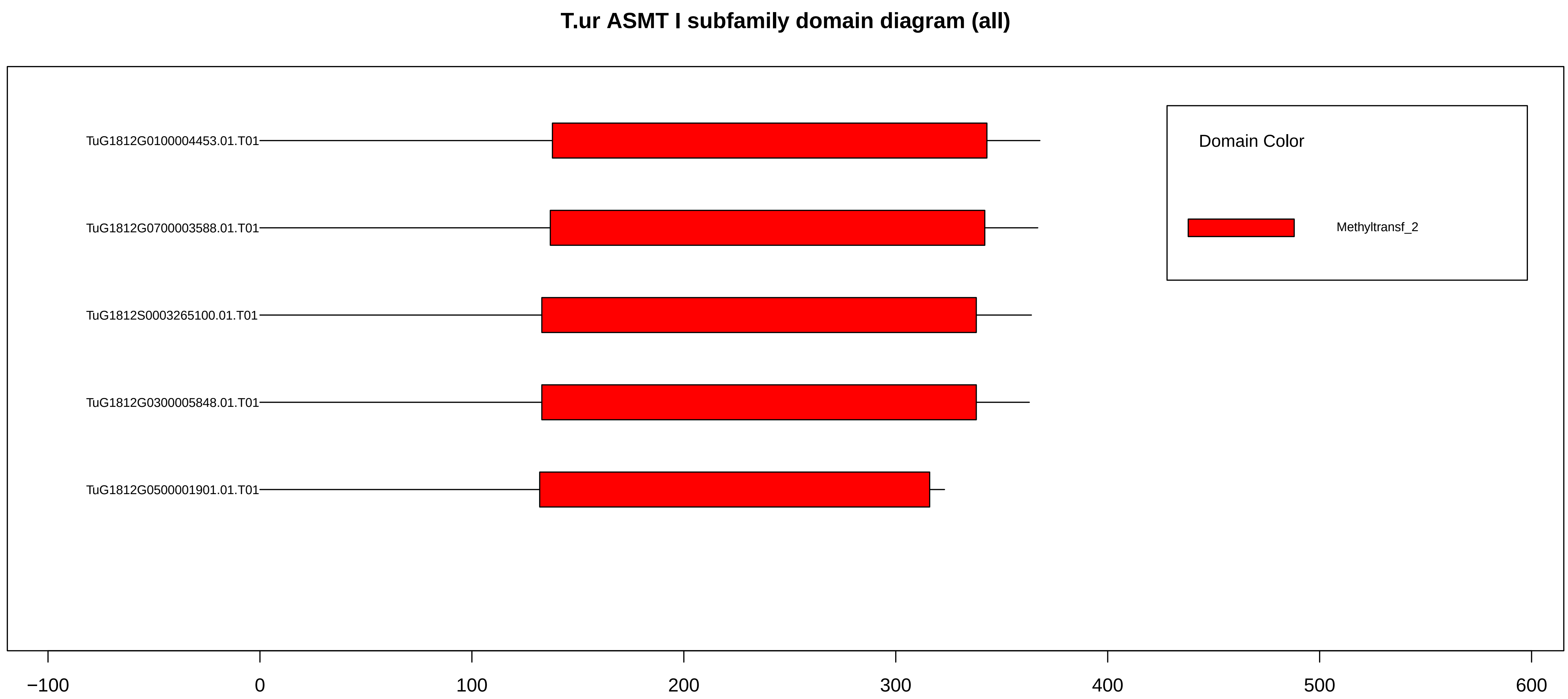
T.ur ASMT I subfamily domain diagram (all)
TuG1812G0100004453.01.T01
TuG1812G0700003588.01.T01
TuG1812S0003265100.01.T01
TuG1812G0300005848.01.T01
TuG1812G0500001901.01.T01
Domain Color
Methyltransf_2
−100
0
100
200
300
400
500
600

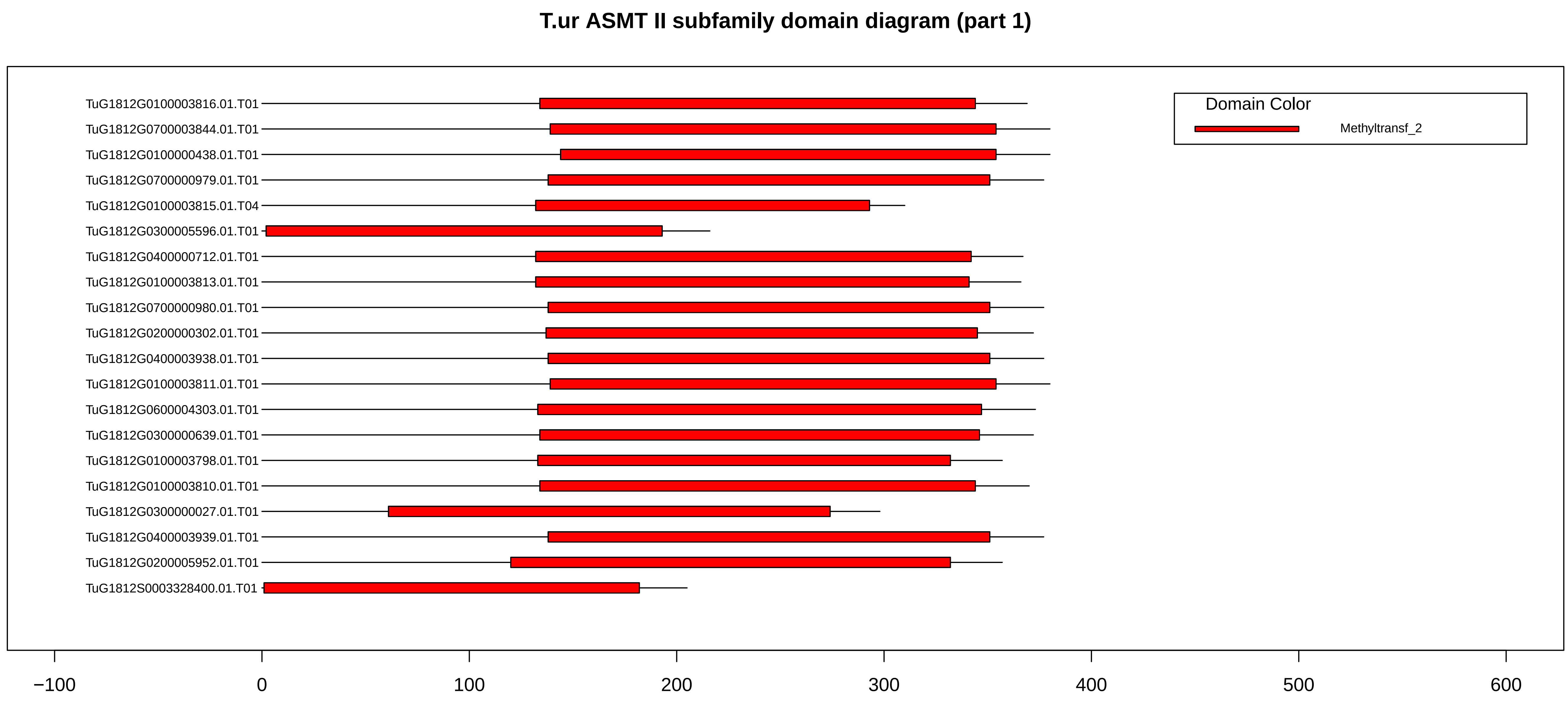

T.ur ASMT II subfamily domain diagram (part 1)
Domain Color
Methyltransf_2
TuG1812G0100003816.01.T01
TuG1812G0700003844.01.T01
TuG1812G0100000438.01.T01
TuG1812G0700000979.01.T01
TuG1812G0100003815.01.T04
TuG1812G0300005596.01.T01
TuG1812G0400000712.01.T01
TuG1812G0100003813.01.T01
TuG1812G0700000980.01.T01
TuG1812G0200000302.01.T01
TuG1812G0400003938.01.T01
TuG1812G0100003811.01.T01
TuG1812G0600004303.01.T01
TuG1812G0300000639.01.T01
TuG1812G0100003798.01.T01
TuG1812G0100003810.01.T01
TuG1812G0300000027.01.T01
TuG1812G0400003939.01.T01
TuG1812G0200005952.01.T01
TuG1812S0003328400.01.T01
−100
0
100
200
300
400
500
600

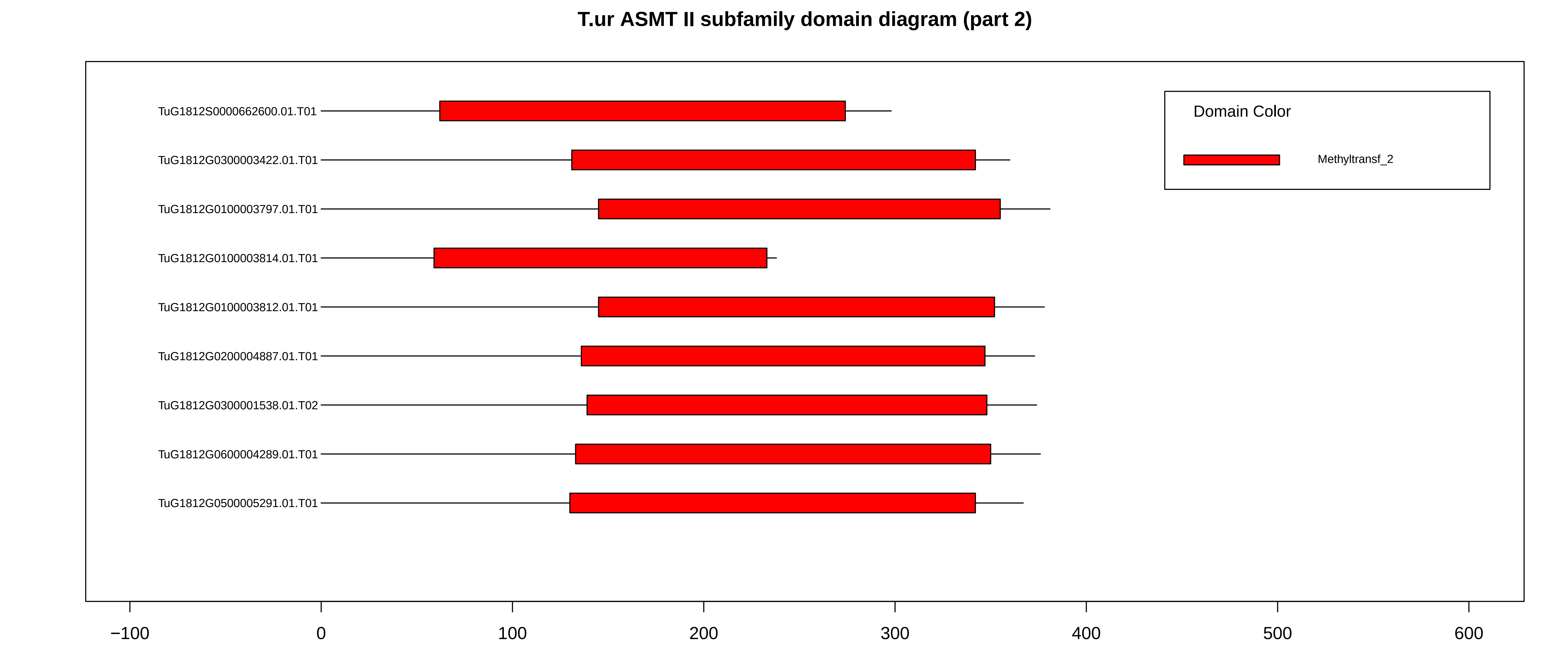

T.ur ASMT II subfamily domain diagram (part 2)
TuG1812S0000662600.01.T01
TuG1812G0300003422.01.T01
TuG1812G0100003797.01.T01
TuG1812G0100003814.01.T01
TuG1812G0100003812.01.T01
TuG1812G0200004887.01.T01
TuG1812G0300001538.01.T02
TuG1812G0600004289.01.T01
TuG1812G0500005291.01.T01
Domain Color
Methyltransf_2
−100
0
100
200
300
400
500
600

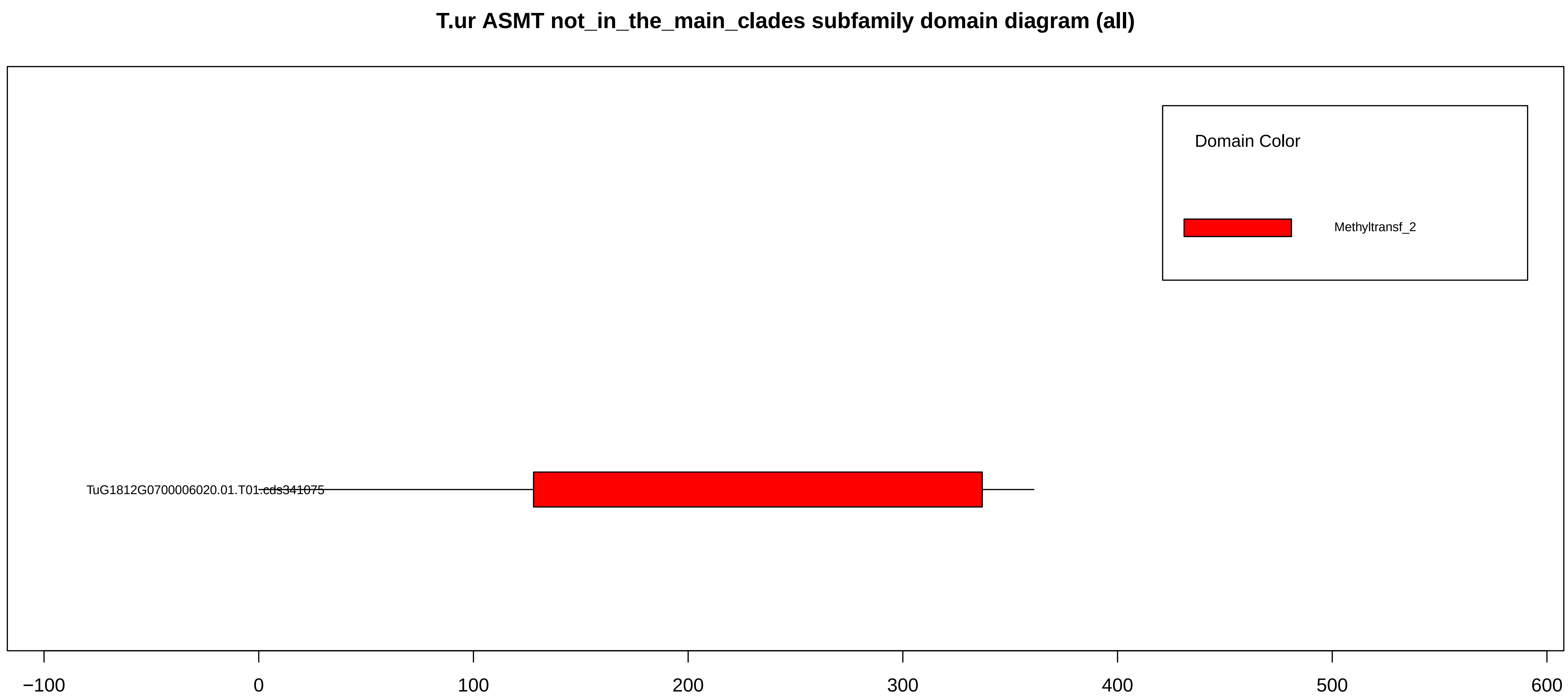
T.ur ASMT not_in_the_main_clades subfamily domain diagram (all)
Domain Color
Methyltransf_2
TuG1812G0700006020.01.T01.cds341075
−100
0
100
200
300
400
500
600

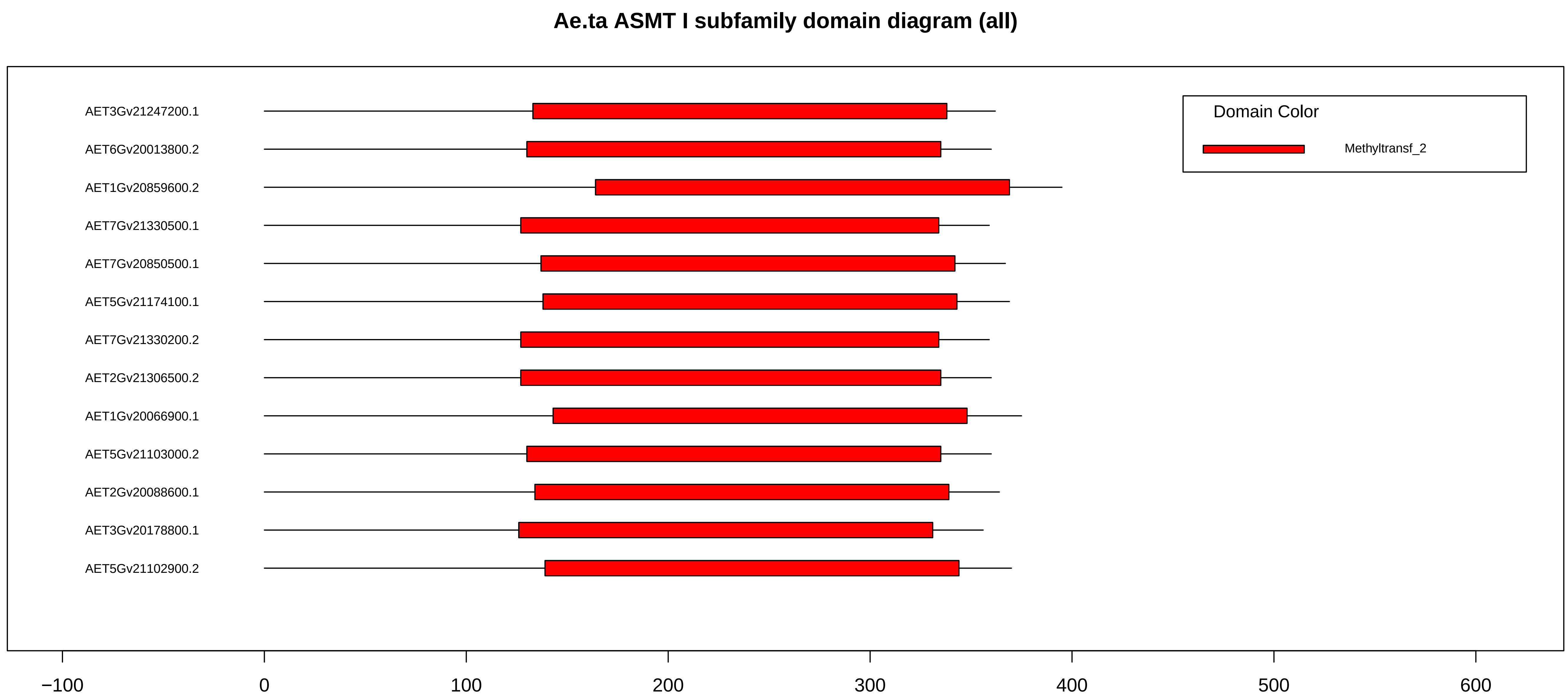

Ae.ta ASMT I subfamily domain diagram (all)
Domain Color
Methyltransf_2
AET3Gv21247200.1
AET6Gv20013800.2
AET1Gv20859600.2
AET7Gv21330500.1
AET7Gv20850500.1
AET5Gv21174100.1
AET7Gv21330200.2
AET2Gv21306500.2
AET1Gv20066900.1
AET5Gv21103000.2
AET2Gv20088600.1
AET3Gv20178800.1
AET5Gv21102900.2
−100
0
100
200
300
400
500
600

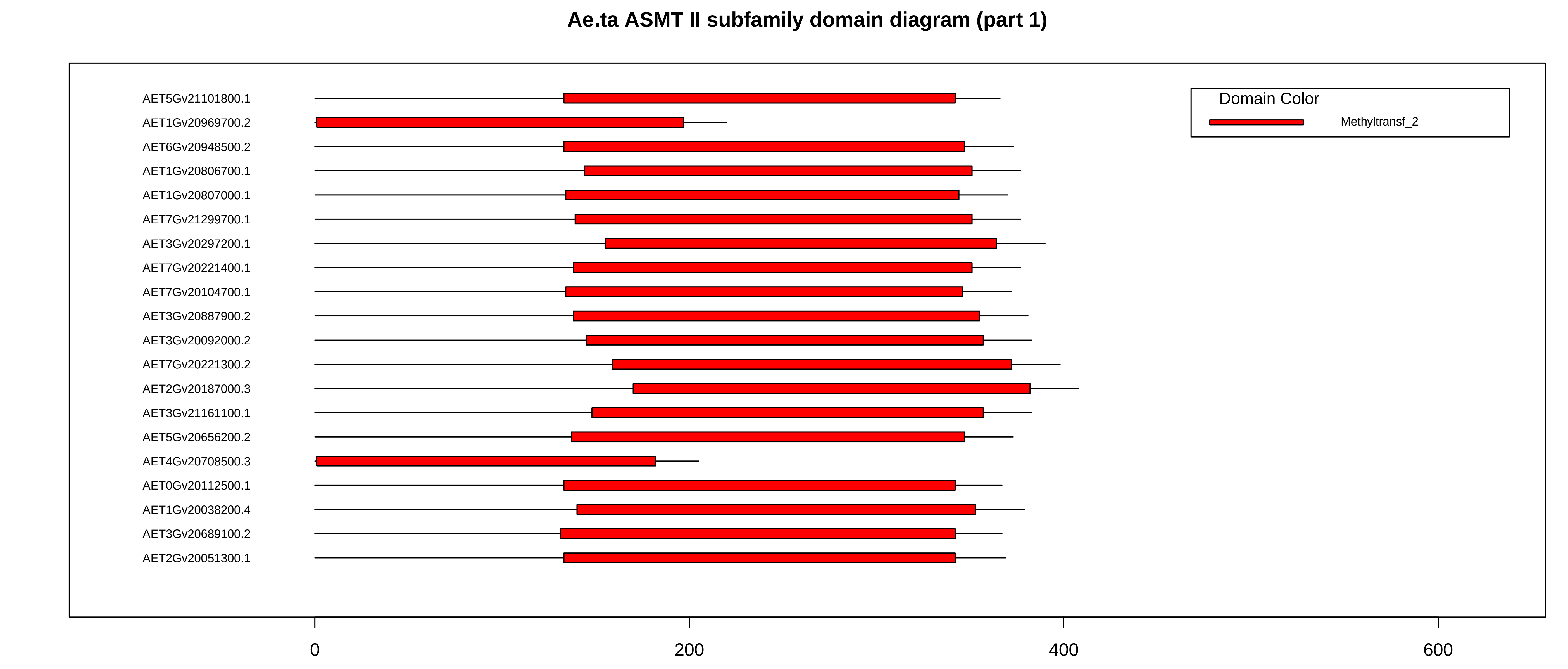
Ae.ta ASMT II subfamily domain diagram (part 1)
AET5Gv21101800.1
AET1Gv20969700.2
AET6Gv20948500.2
AET1Gv20806700.1
AET1Gv20807000.1
AET7Gv21299700.1
AET3Gv20297200.1
AET7Gv20221400.1
AET7Gv20104700.1
AET3Gv20887900.2
AET3Gv20092000.2
AET7Gv20221300.2
AET2Gv20187000.3
AET3Gv21161100.1
AET5Gv20656200.2
AET4Gv20708500.3
AET0Gv20112500.1
AET1Gv20038200.4
AET3Gv20689100.2
AET2Gv20051300.1
Domain Color
Methyltransf_2
0
200
400
600

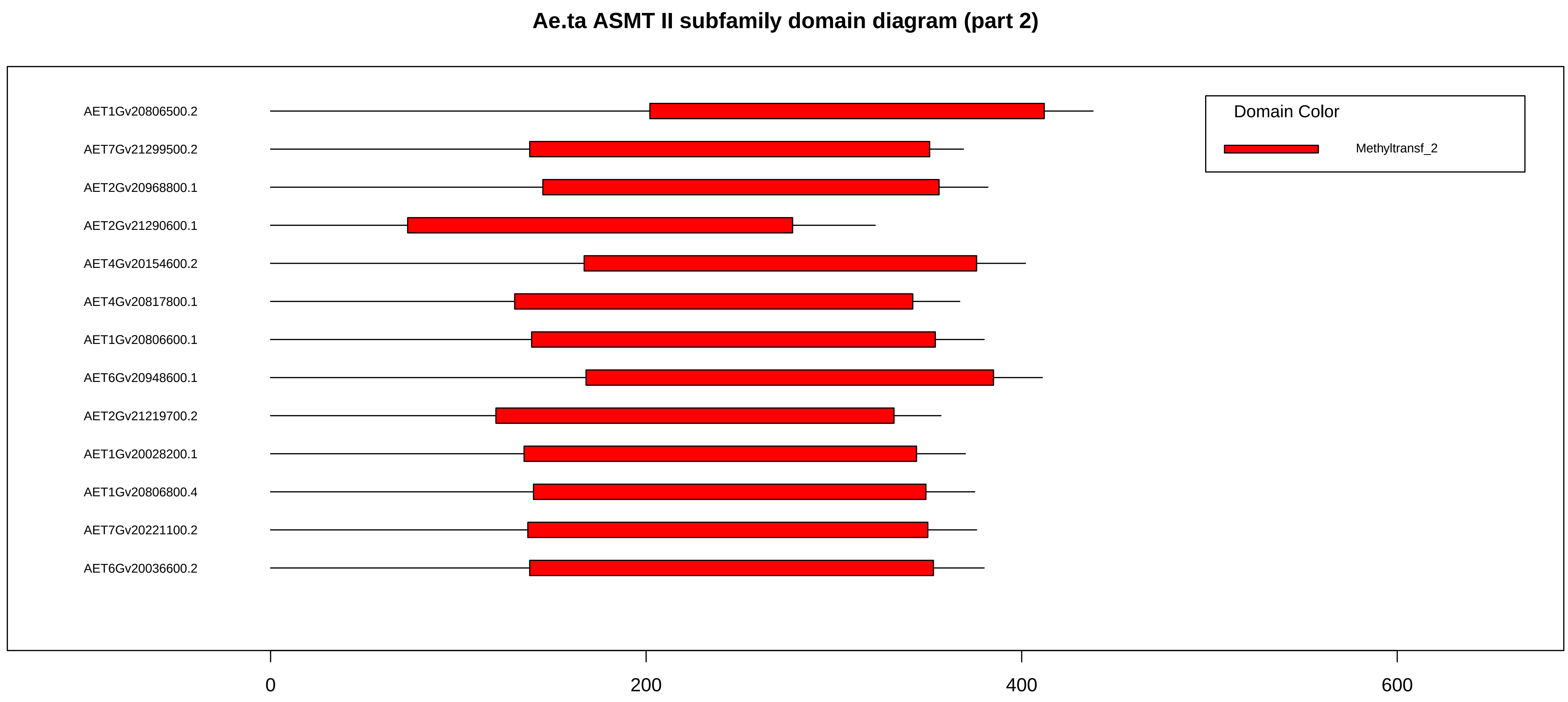
Ae.ta ASMT II subfamily domain diagram (part 2)
AET1Gv20806500.2
AET7Gv21299500.2
AET2Gv20968800.1
AET2Gv21290600.1
AET4Gv20154600.2
AET4Gv20817800.1
AET1Gv20806600.1
AET6Gv20948600.1
AET2Gv21219700.2
AET1Gv20028200.1
AET1Gv20806800.4
AET7Gv20221100.2
AET6Gv20036600.2
Domain Color
Methyltransf_2
0
200
400
600

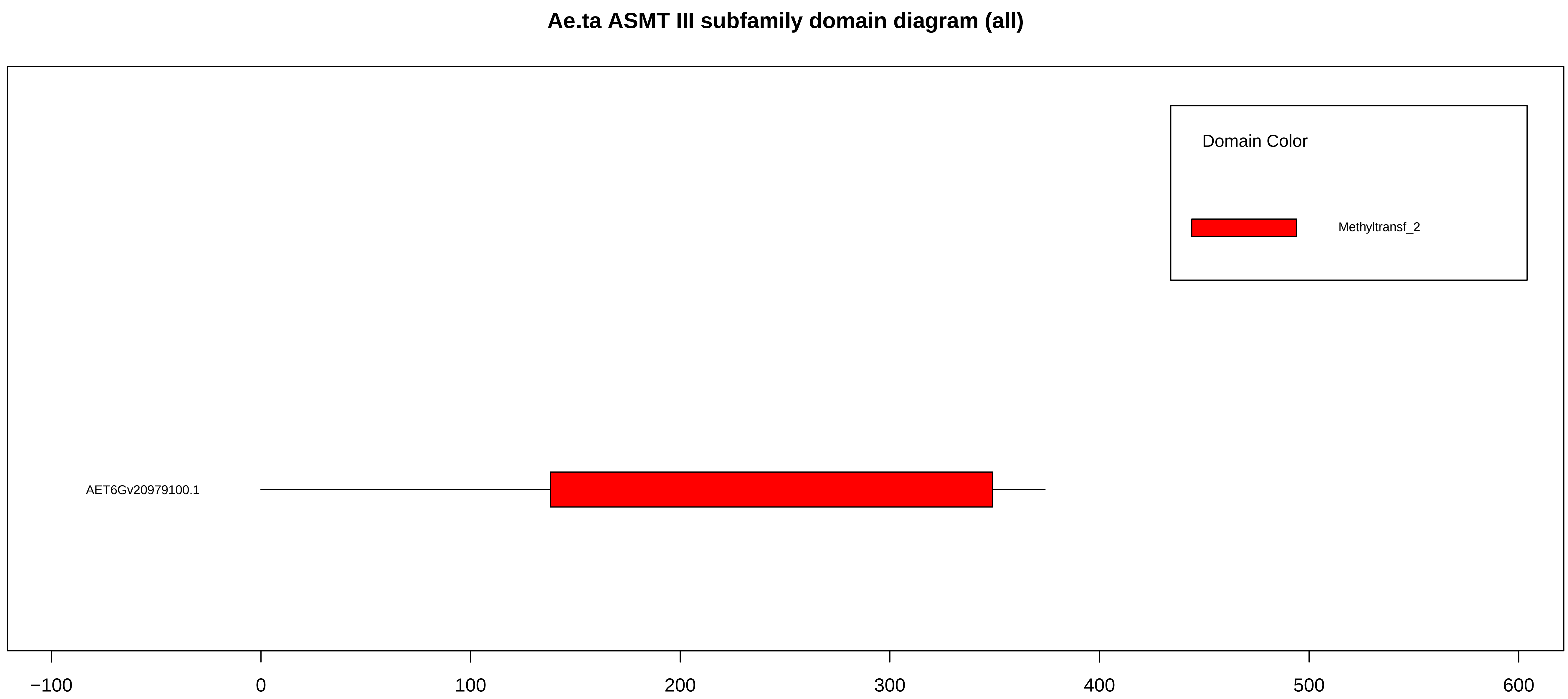
Ae.ta ASMT III subfamily domain diagram (all)
Domain Color
Methyltransf_2
AET6Gv20979100.1
−100
0
100
200
300
400
500
600

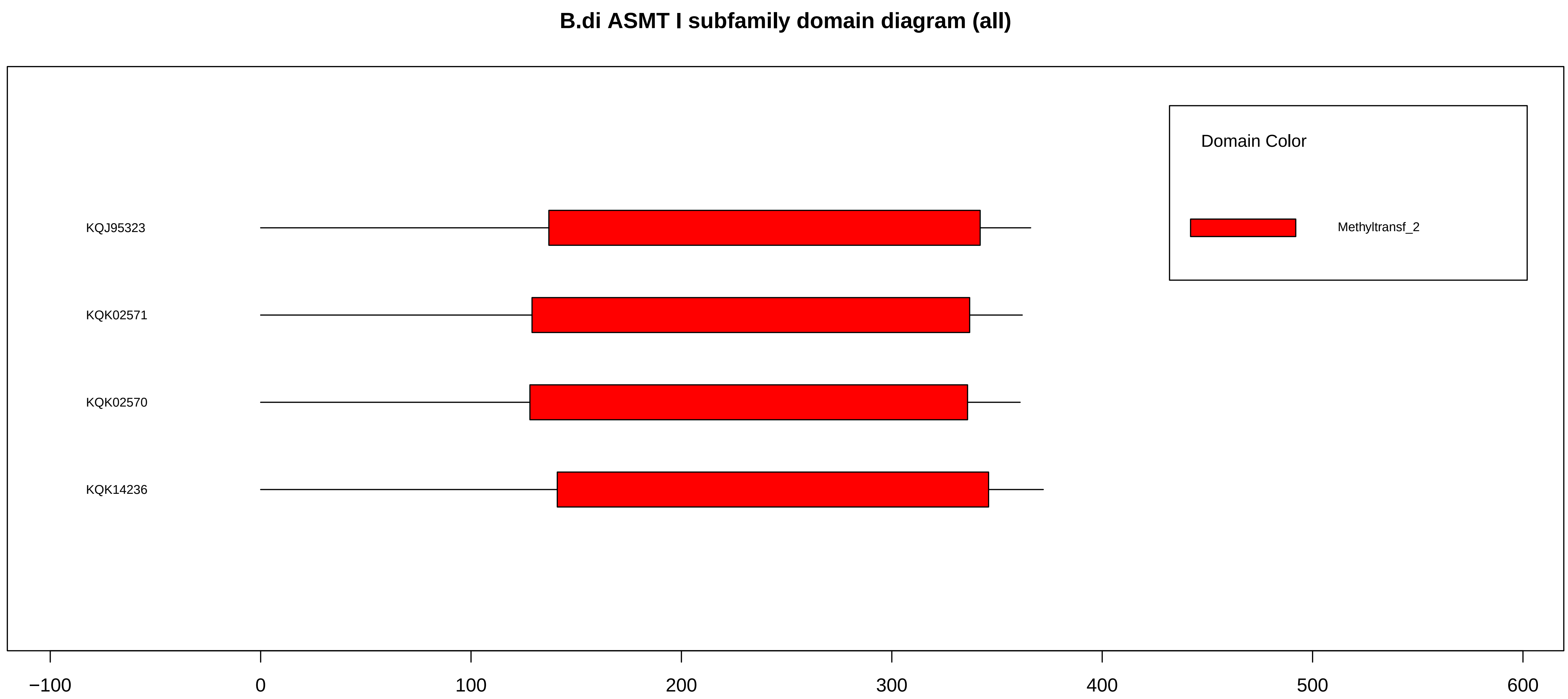
B.di ASMT I subfamily domain diagram (all)
Domain Color
Methyltransf_2
KQJ95323
KQK02571
KQK02570
KQK14236
−100
0
100
200
300
400
500
600

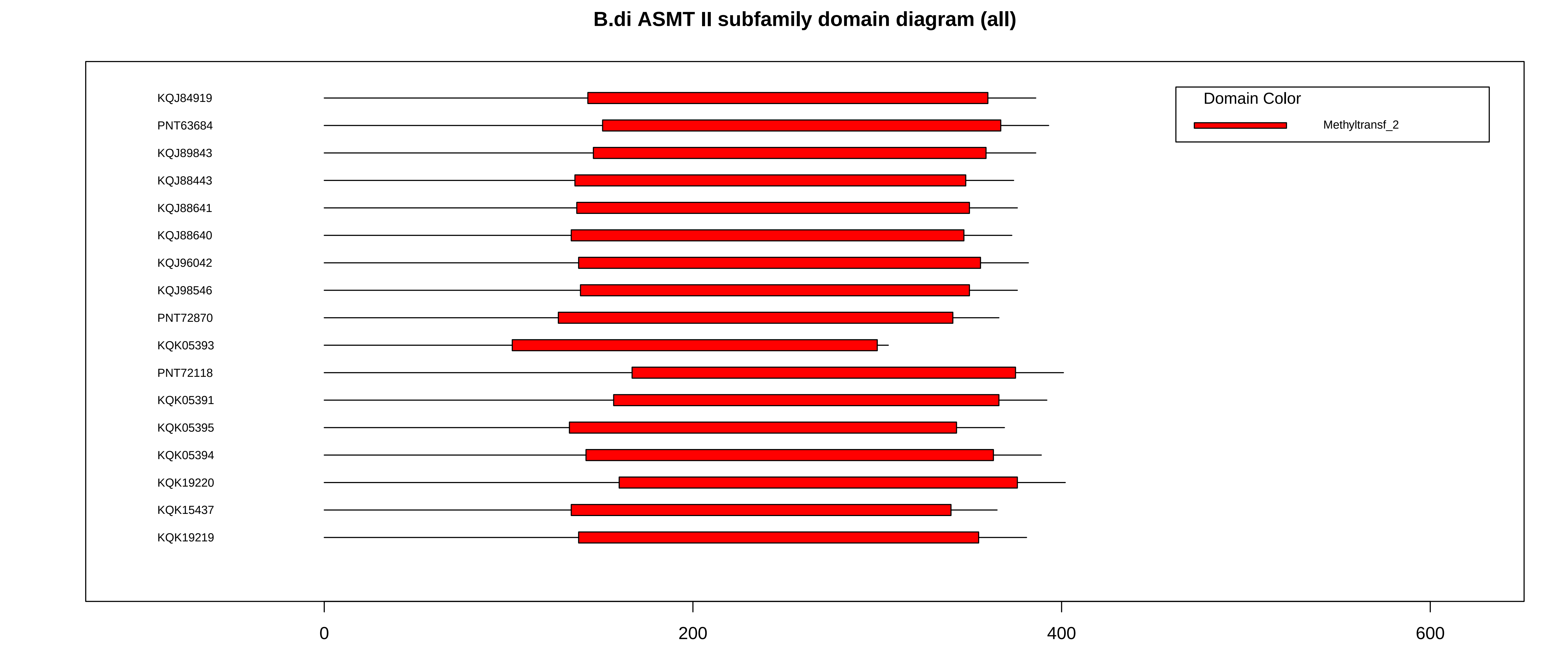
B.di ASMT II subfamily domain diagram (all)
KQJ84919
PNT63684
KQJ89843
KQJ88443
KQJ88641
KQJ88640
KQJ96042
KQJ98546
PNT72870
KQK05393
PNT72118
KQK05391
KQK05395
KQK05394
KQK19220
KQK15437
KQK19219
Domain Color
Methyltransf_2
0
200
400
600

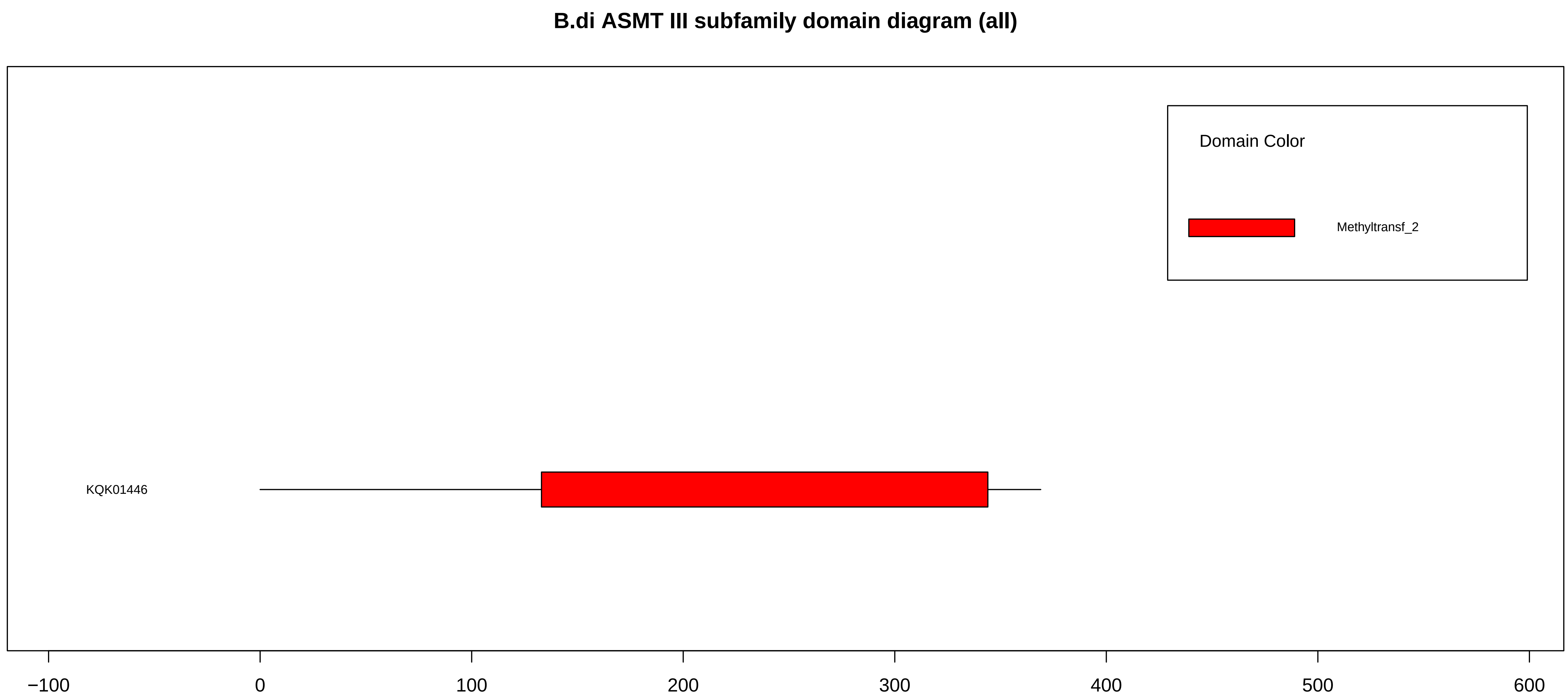
B.di ASMT III subfamily domain diagram (all)
Domain Color
Methyltransf_2
KQK01446
−100
0
100
200
300
400
500
600

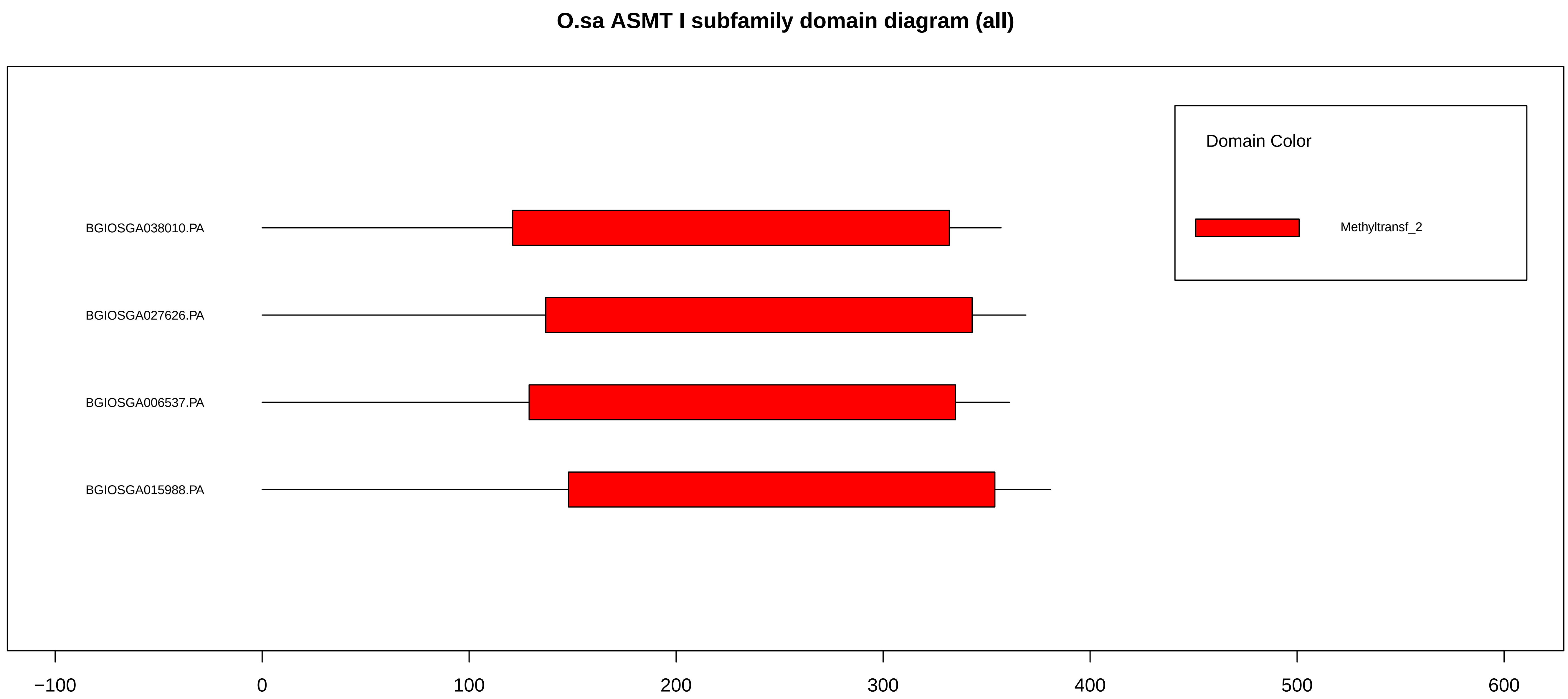

O.sa ASMT I subfamily domain diagram (all)
Domain Color
Methyltransf_2
BGIOSGA038010.PA
BGIOSGA027626.PA
BGIOSGA006537.PA
BGIOSGA015988.PA
−100
0
100
200
300
400
500
600

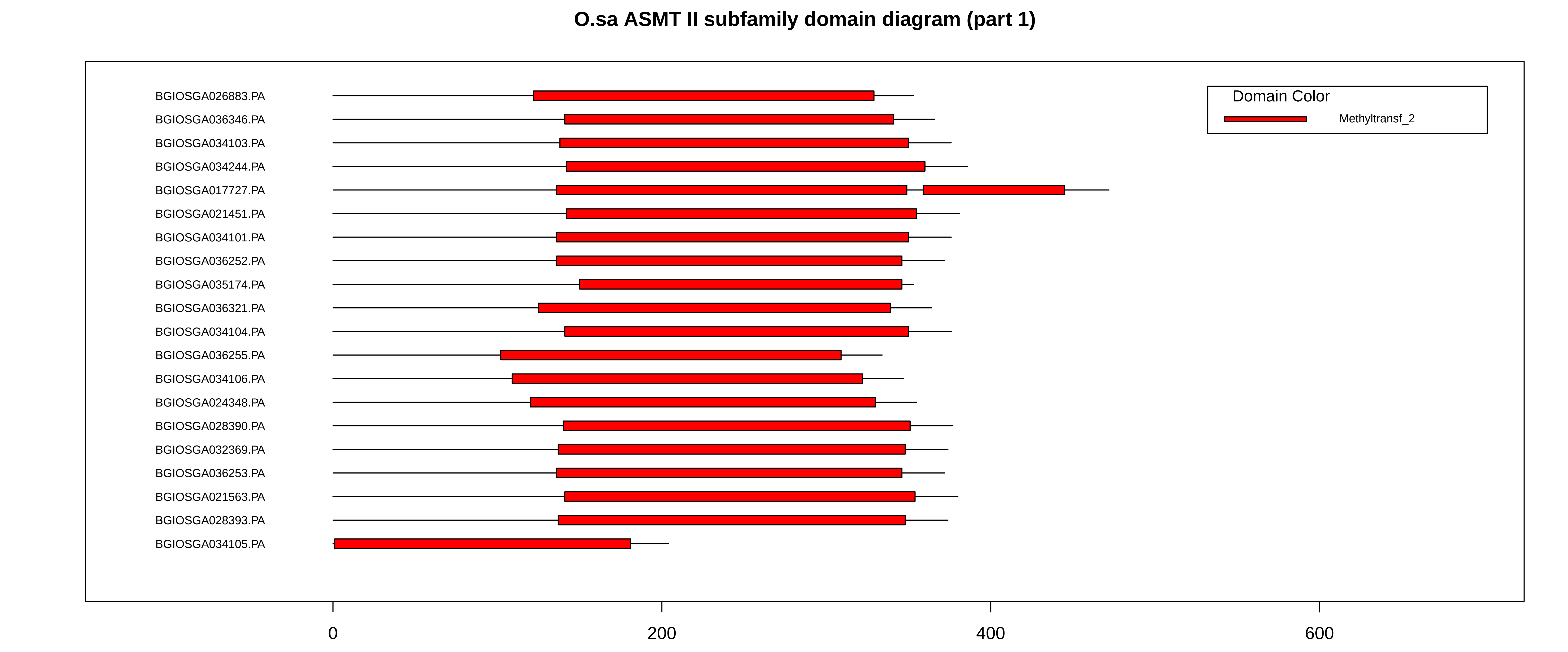
O.sa ASMT II subfamily domain diagram (part 1)
Domain Color
Methyltransf_2
BGIOSGA026883.PA
BGIOSGA036346.PA
BGIOSGA034103.PA
BGIOSGA034244.PA
BGIOSGA017727.PA
BGIOSGA021451.PA
BGIOSGA034101.PA
BGIOSGA036252.PA
BGIOSGA035174.PA
BGIOSGA036321.PA
BGIOSGA034104.PA
BGIOSGA036255.PA
BGIOSGA034106.PA
BGIOSGA024348.PA
BGIOSGA028390.PA
BGIOSGA032369.PA
BGIOSGA036253.PA
BGIOSGA021563.PA
BGIOSGA028393.PA
BGIOSGA034105.PA
0
200
400
600

O.sa ASMT II subfamily domain diagram (part 2)

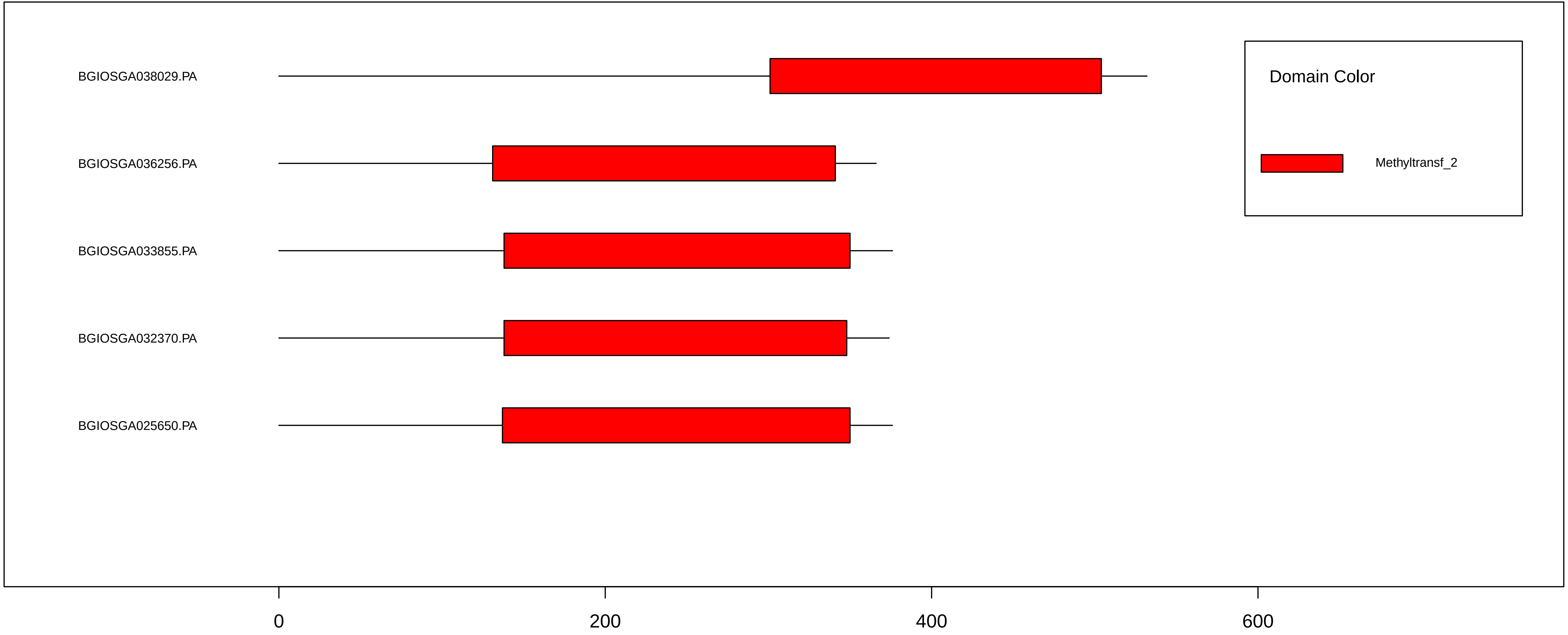

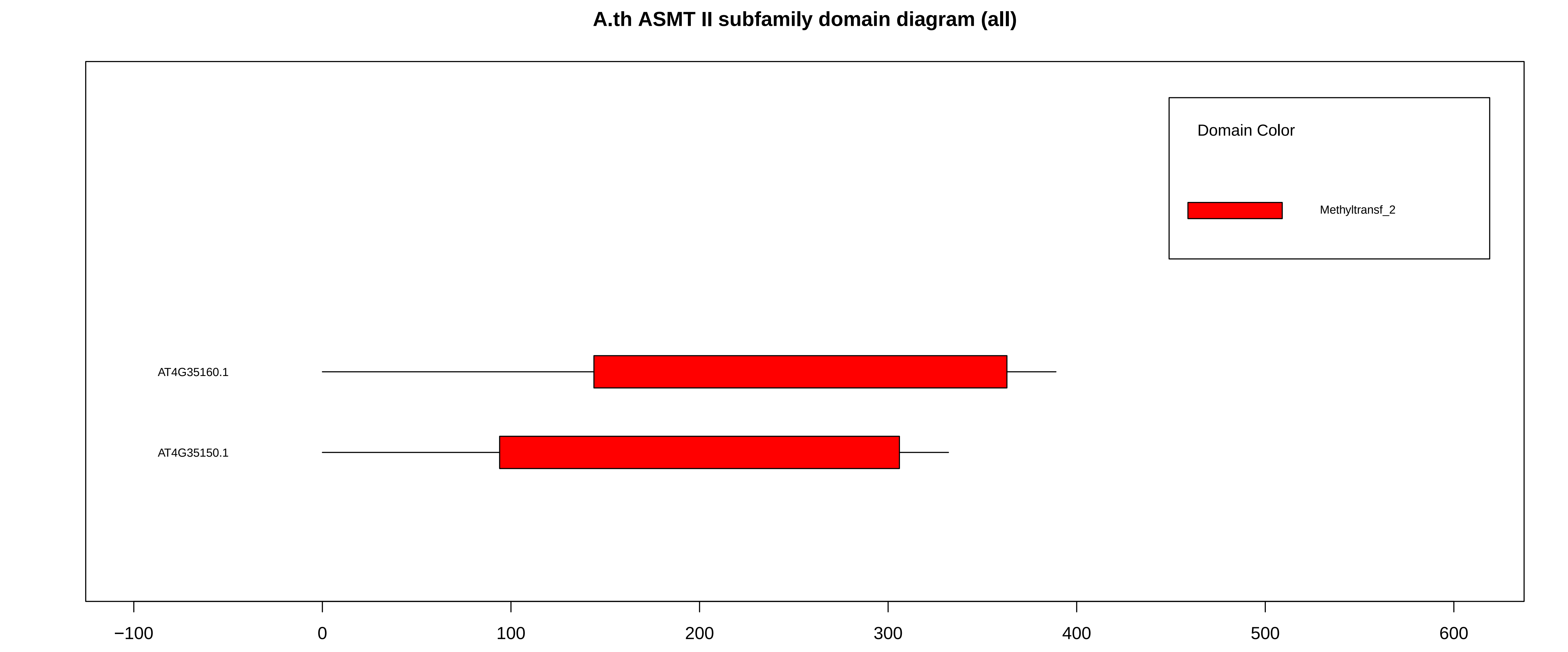

A.th ASMT II subfamily domain diagram (all)
Domain Color
Methyltransf_2
AT4G35160.1
AT4G35150.1
−100
0
100
200
300
400
500
600

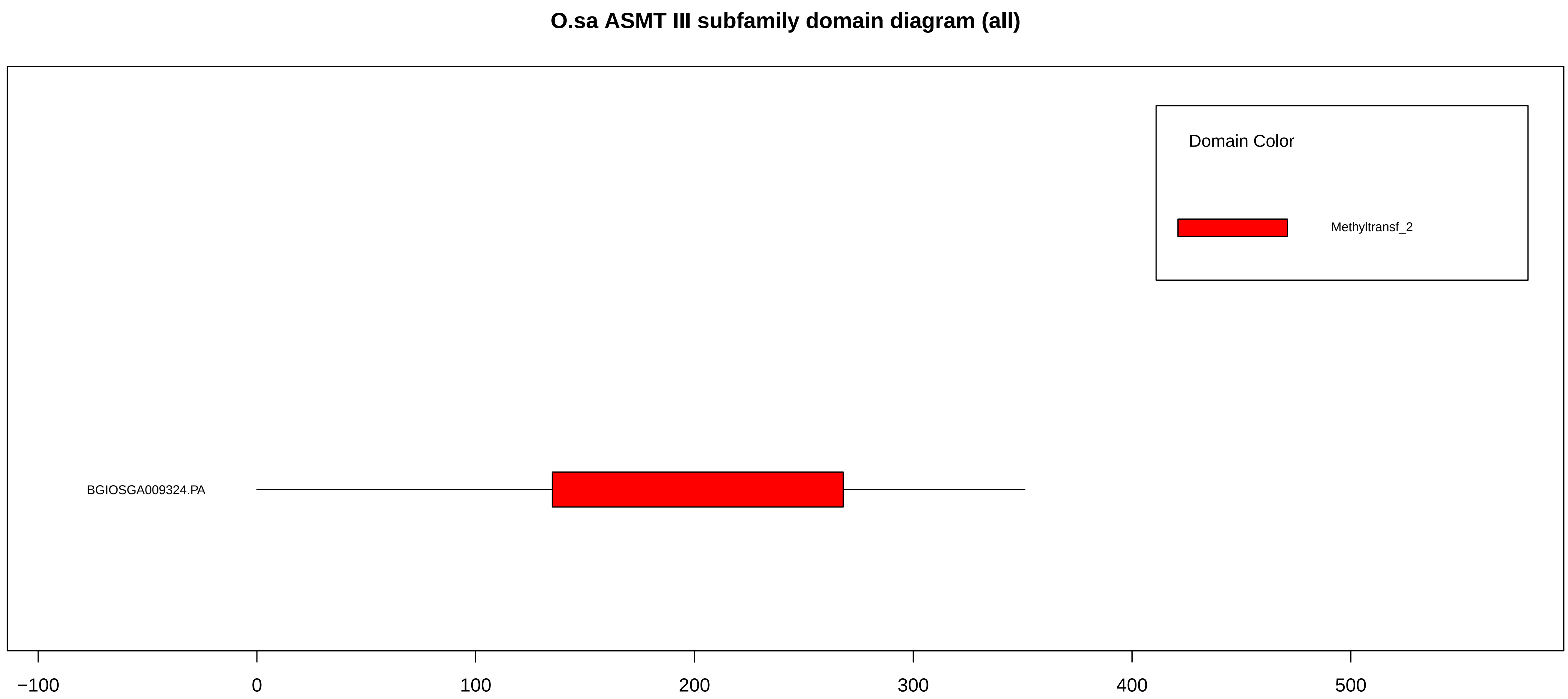

O.sa ASMT III subfamily domain diagram (all)
Domain Color
Methyltransf_2
BGIOSGA009324.PA
−100
0
100
200
300
400
500

Z.ma ASMT I subfamily domain diagram (all)

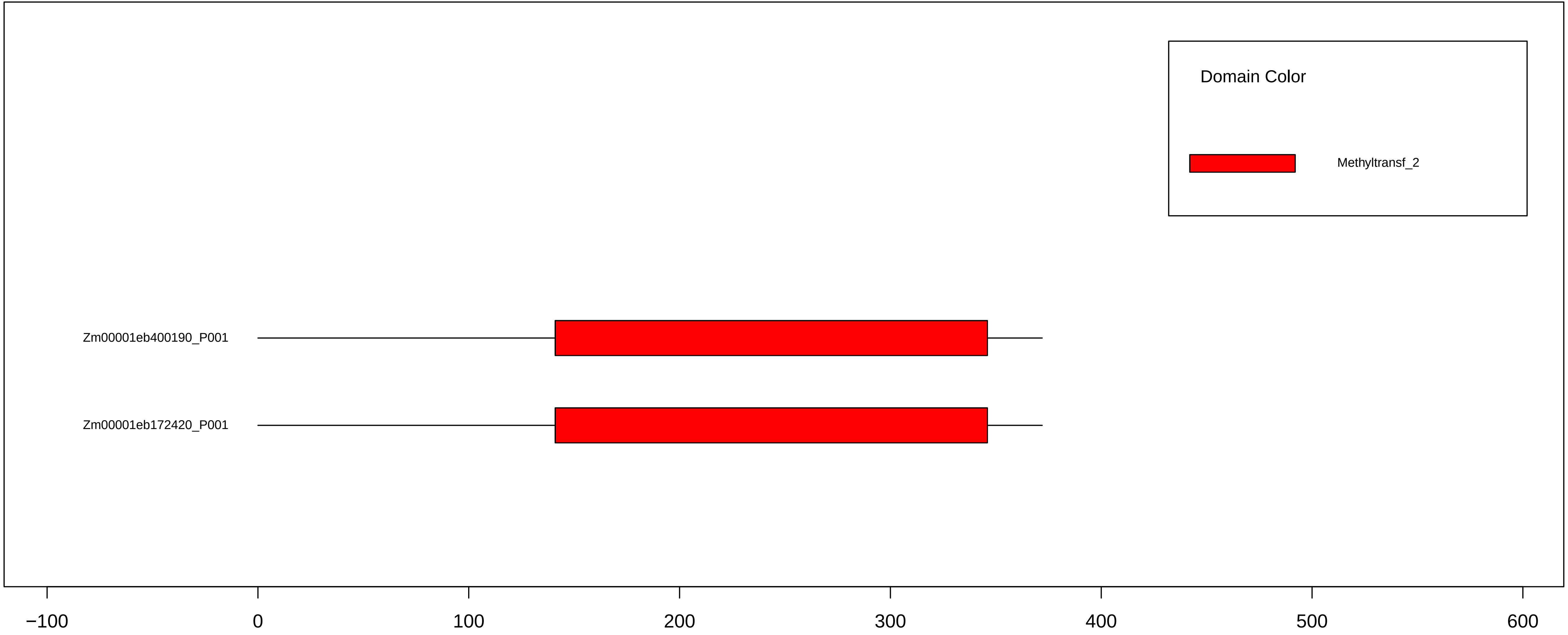

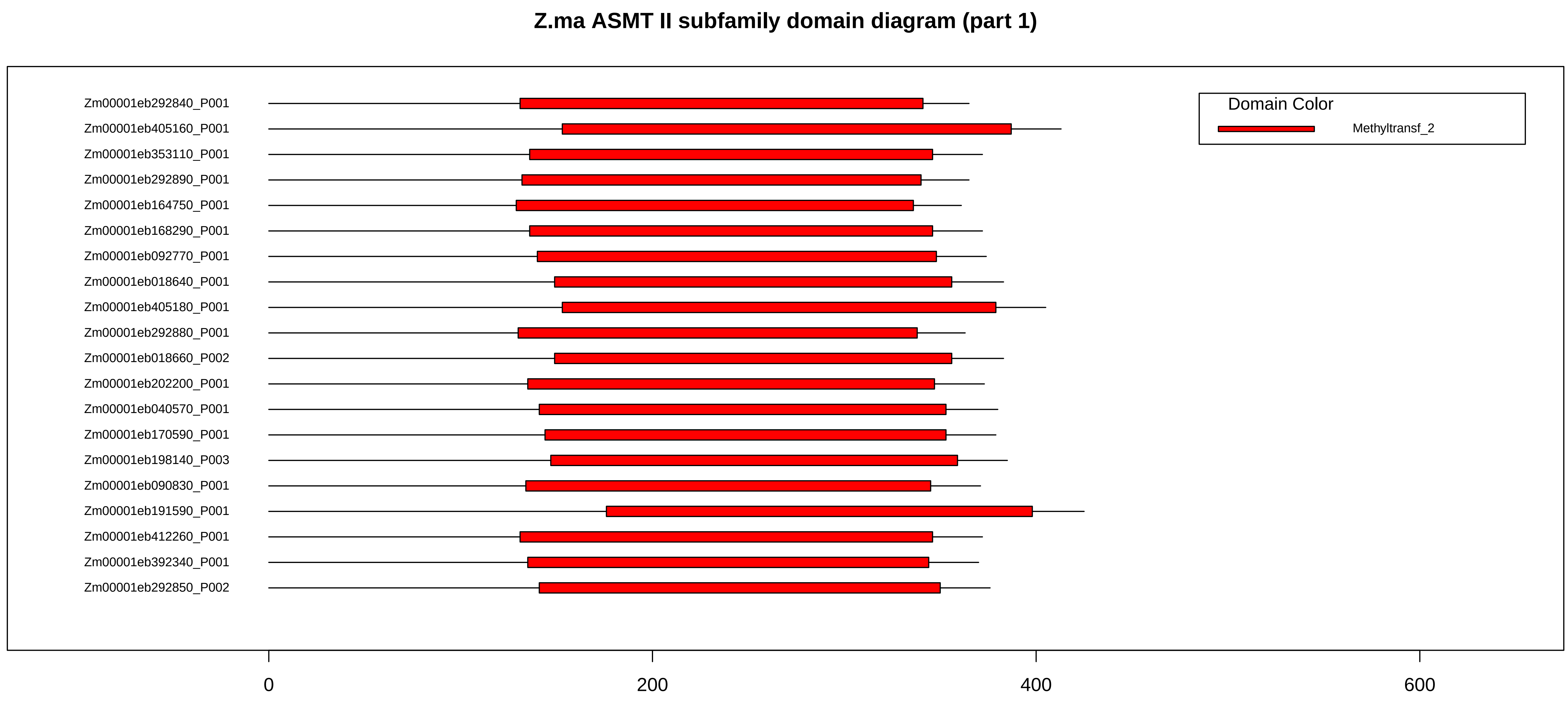
Z.ma ASMT II subfamily domain diagram (part 1)
Zm00001eb292840_P001
Zm00001eb405160_P001
Zm00001eb353110_P001
Zm00001eb292890_P001
Zm00001eb164750_P001
Zm00001eb168290_P001
Zm00001eb092770_P001
Zm00001eb018640_P001
Zm00001eb405180_P001
Zm00001eb292880_P001
Zm00001eb018660_P002
Zm00001eb202200_P001
Zm00001eb040570_P001
Zm00001eb170590_P001
Zm00001eb198140_P003
Zm00001eb090830_P001
Zm00001eb191590_P001
Zm00001eb412260_P001
Zm00001eb392340_P001
Zm00001eb292850_P002
Domain Color
Methyltransf_2
0
200
400
600

Z.ma ASMT II subfamily domain diagram (part 2)

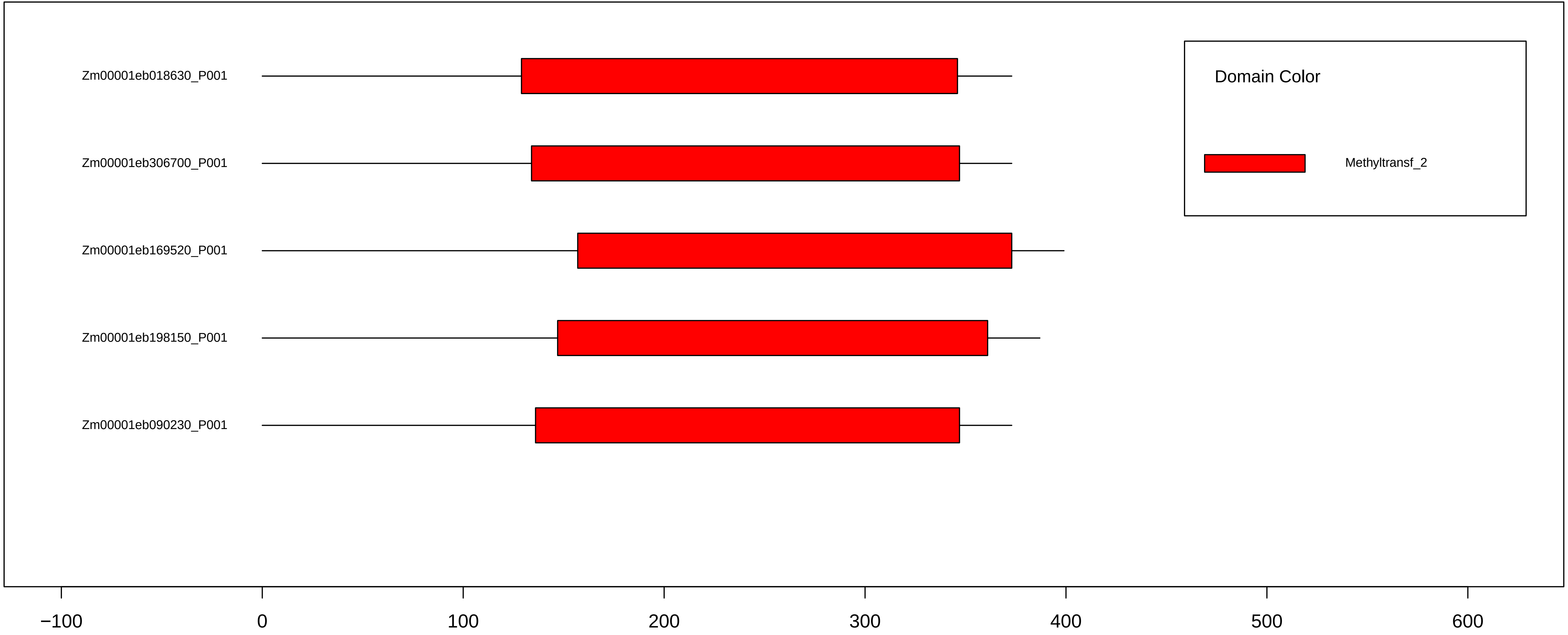

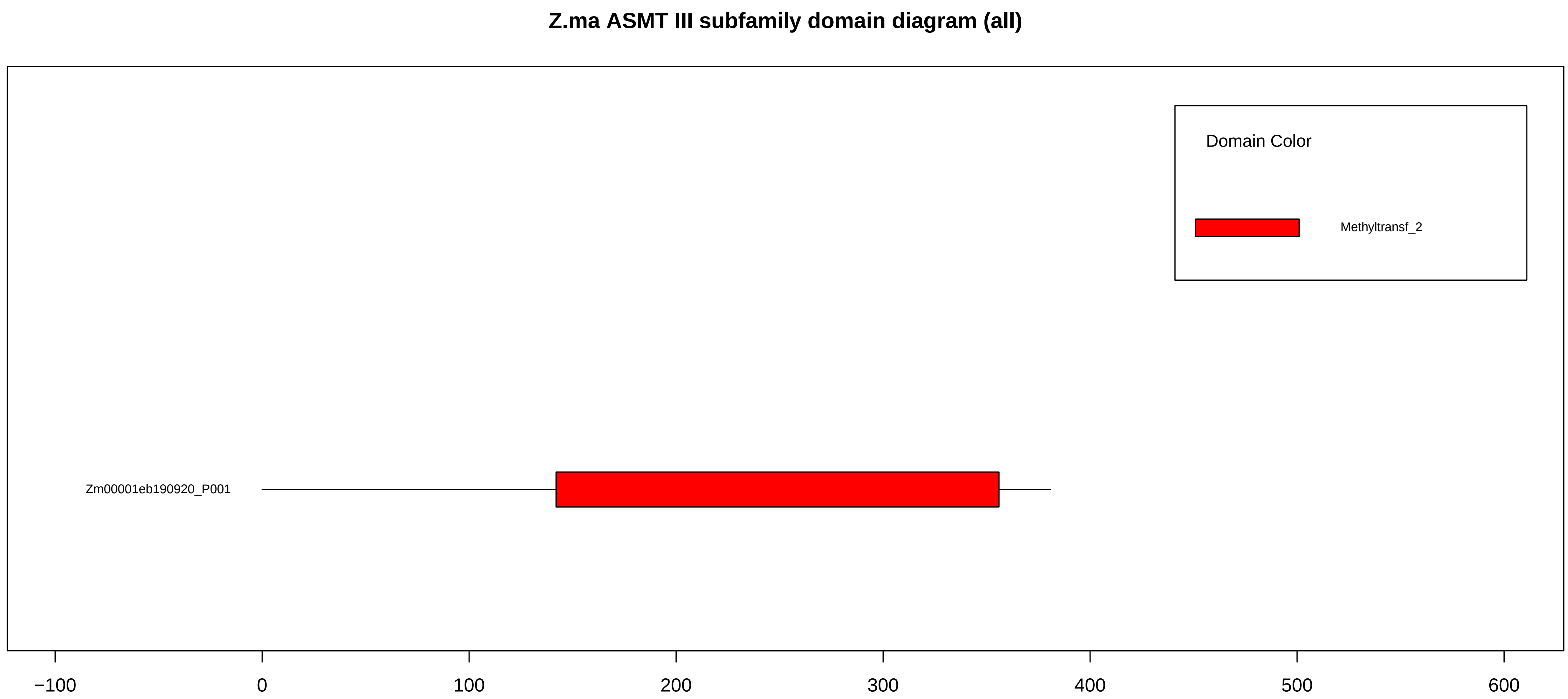
Z.ma ASMT III subfamily domain diagram (all)
Domain Color
Methyltransf_2
Zm00001eb190920_P001
−100
0
100
200
300
400
500
600

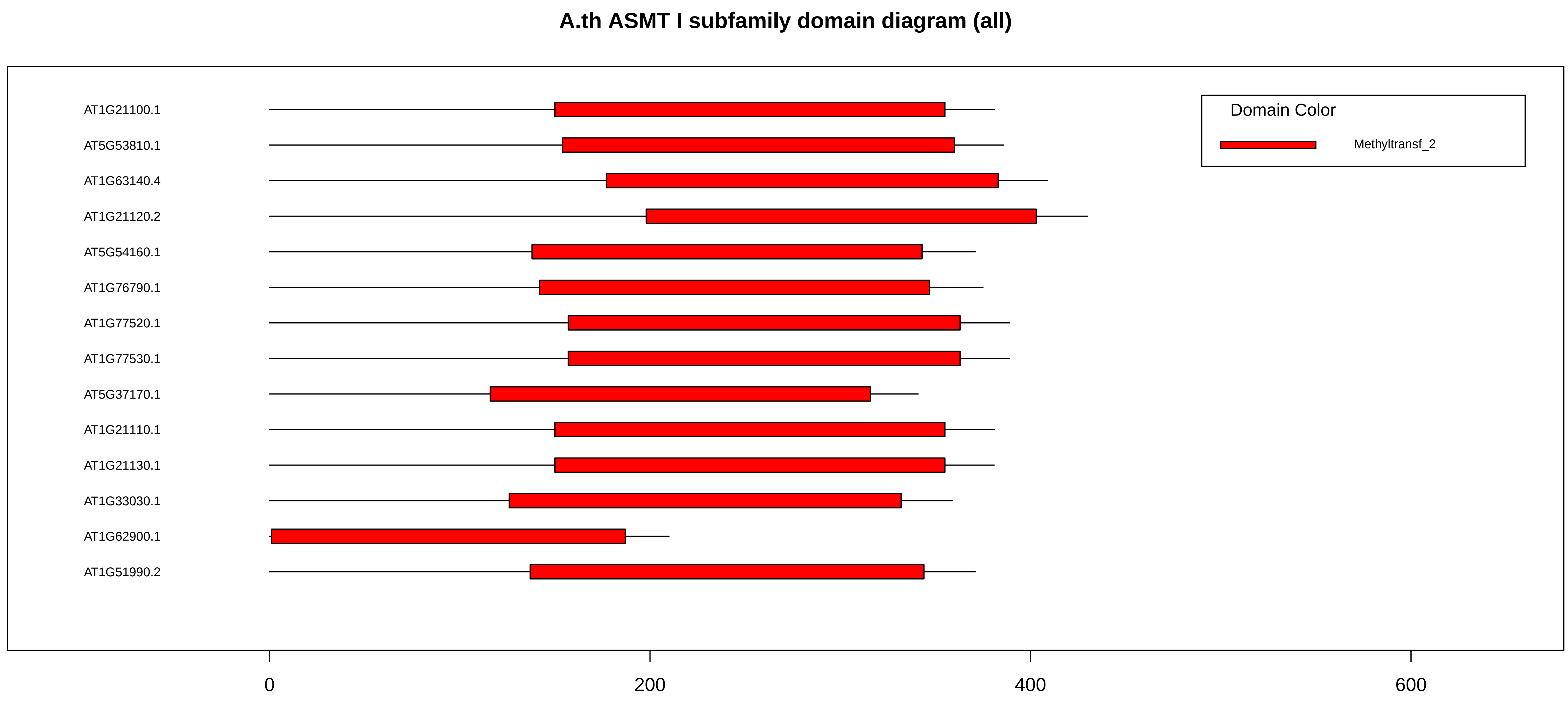
A.th ASMT I subfamily domain diagram (all)
AT1G21100.1
AT5G53810.1
AT1G63140.4
AT1G21120.2
AT5G54160.1
AT1G76790.1
AT1G77520.1
AT1G77530.1
AT5G37170.1
AT1G21110.1
AT1G21130.1
AT1G33030.1
AT1G62900.1
AT1G51990.2
Domain Color
Methyltransf_2
0
200
400
600

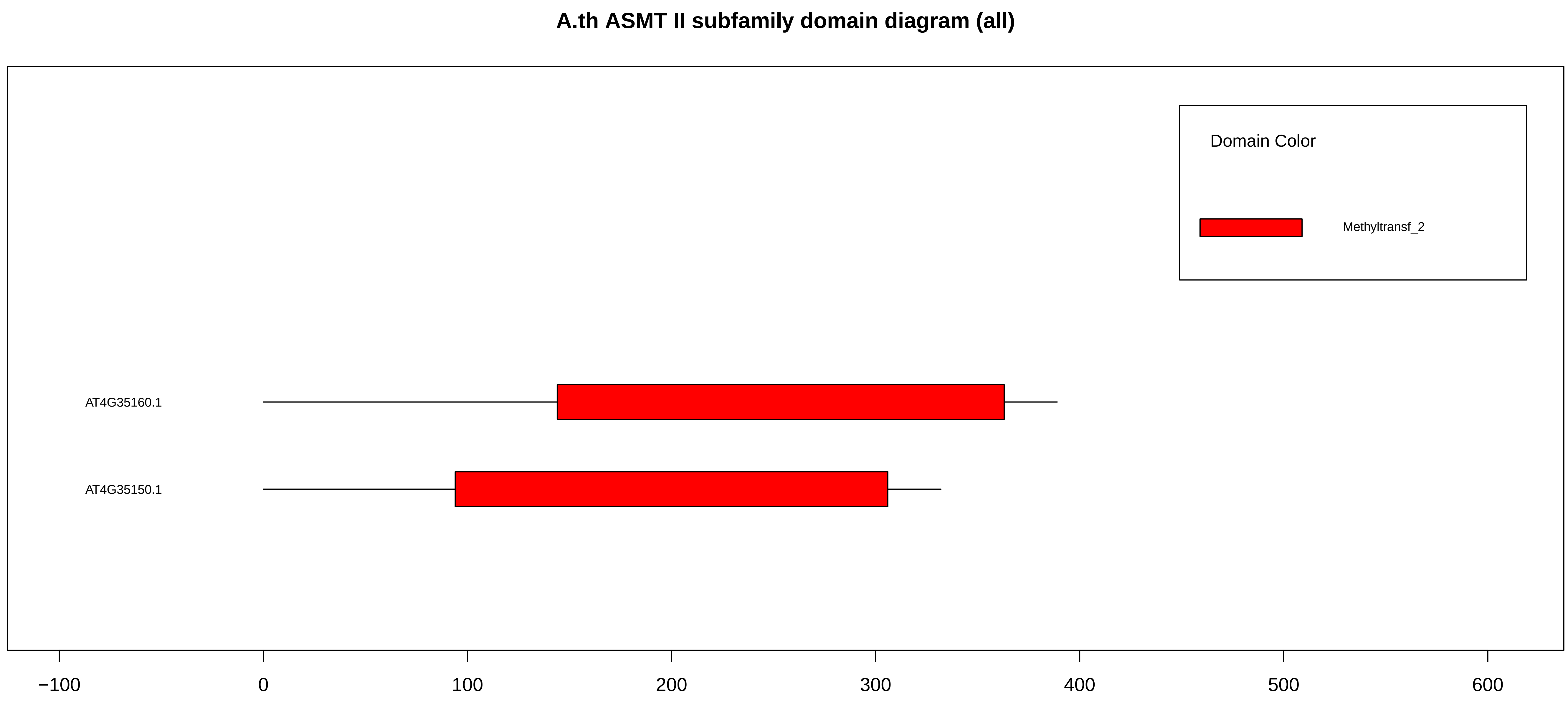
A.th ASMT II subfamily domain diagram (all)
Domain Color
Methyltransf_2
AT4G35160.1
AT4G35150.1
−100
0
100
200
300
400
500
600

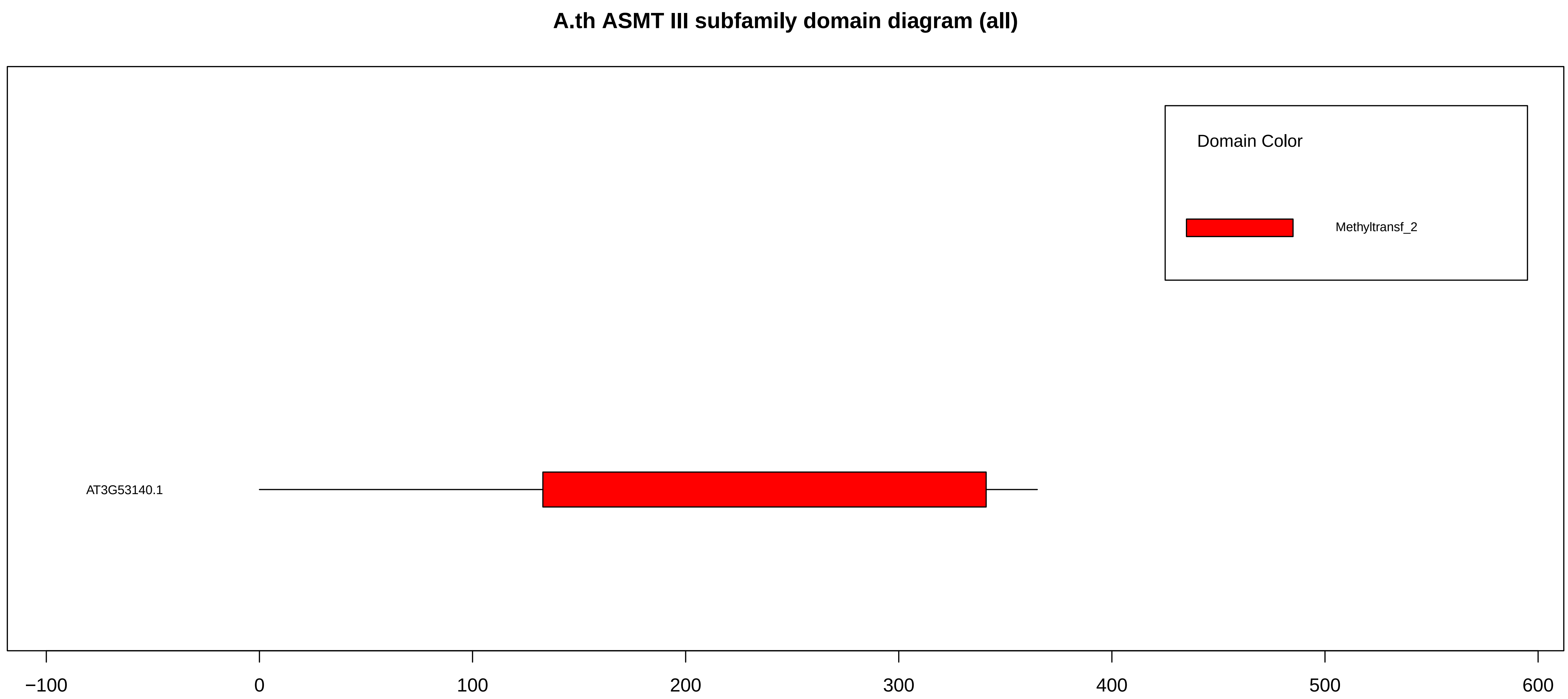
A.th ASMT III subfamily domain diagram (all)
Domain Color
Methyltransf_2
AT3G53140.1
−100
0
100
200
300
400
500
600

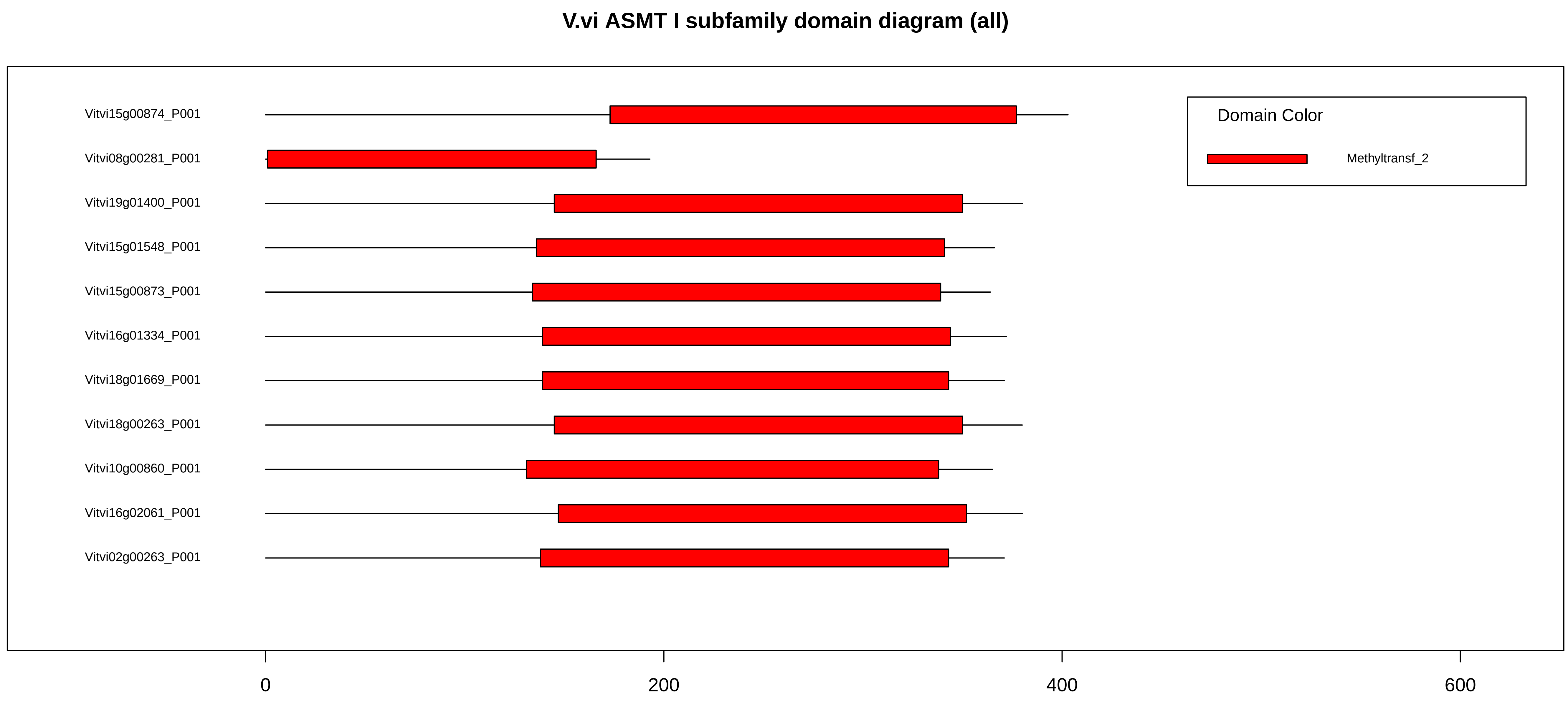
V.vi ASMT I subfamily domain diagram (all)
Vitvi15g00874_P001
Vitvi08g00281_P001
Vitvi19g01400_P001
Vitvi15g01548_P001
Vitvi15g00873_P001
Vitvi16g01334_P001
Vitvi18g01669_P001
Vitvi18g00263_P001
Vitvi10g00860_P001
Vitvi16g02061_P001
Vitvi02g00263_P001
Domain Color
Methyltransf_2
0
200
400
600

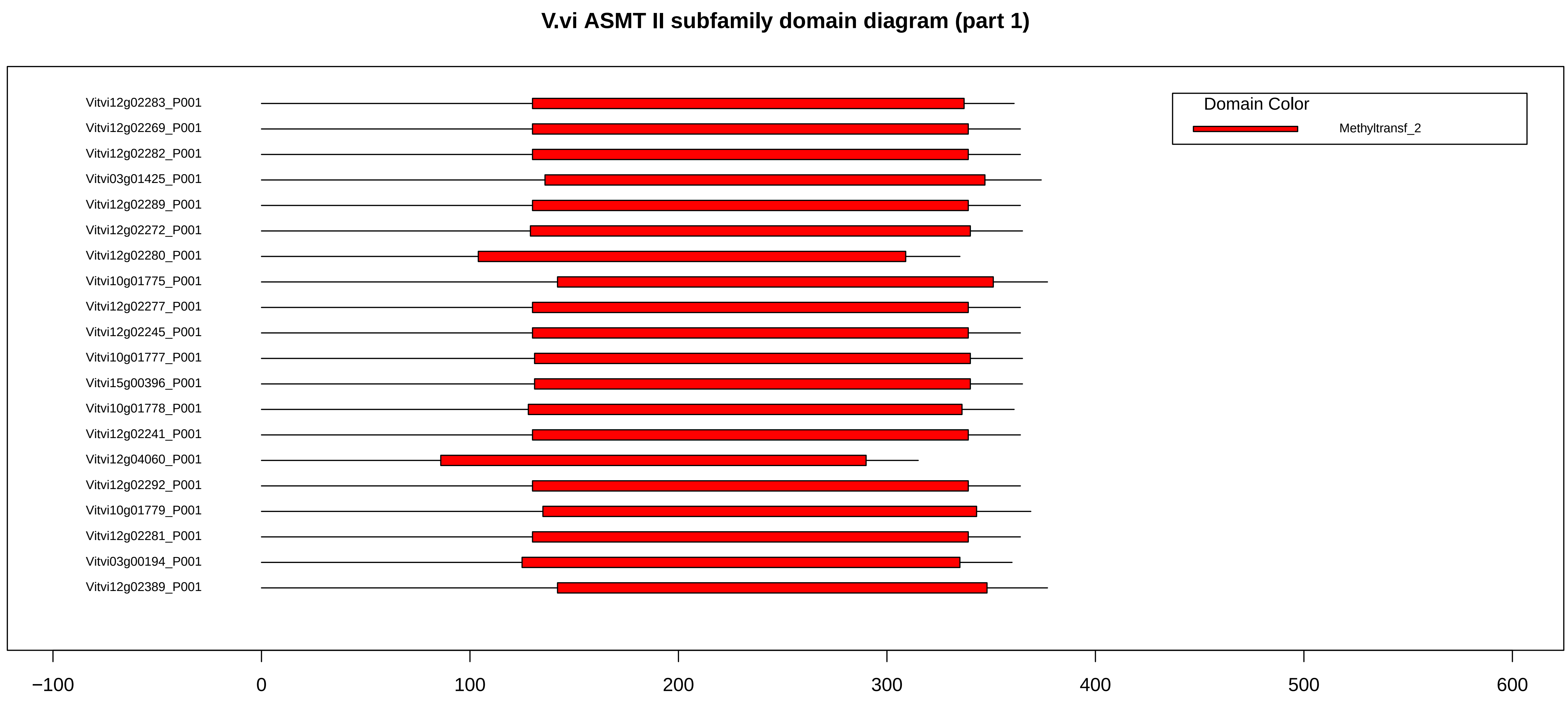

V.vi ASMT II subfamily domain diagram (part 1)
Vitvi12g02283_P001
Vitvi12g02269_P001
Vitvi12g02282_P001
Vitvi03g01425_P001
Vitvi12g02289_P001
Vitvi12g02272_P001
Vitvi12g02280_P001
Vitvi10g01775_P001
Vitvi12g02277_P001
Vitvi12g02245_P001
Vitvi10g01777_P001
Vitvi15g00396_P001
Vitvi10g01778_P001
Vitvi12g02241_P001
Vitvi12g04060_P001
Vitvi12g02292_P001
Vitvi10g01779_P001
Vitvi12g02281_P001
Vitvi03g00194_P001
Vitvi12g02389_P001
Domain Color
Methyltransf_2
−100
0
100
200
300
400
500
600

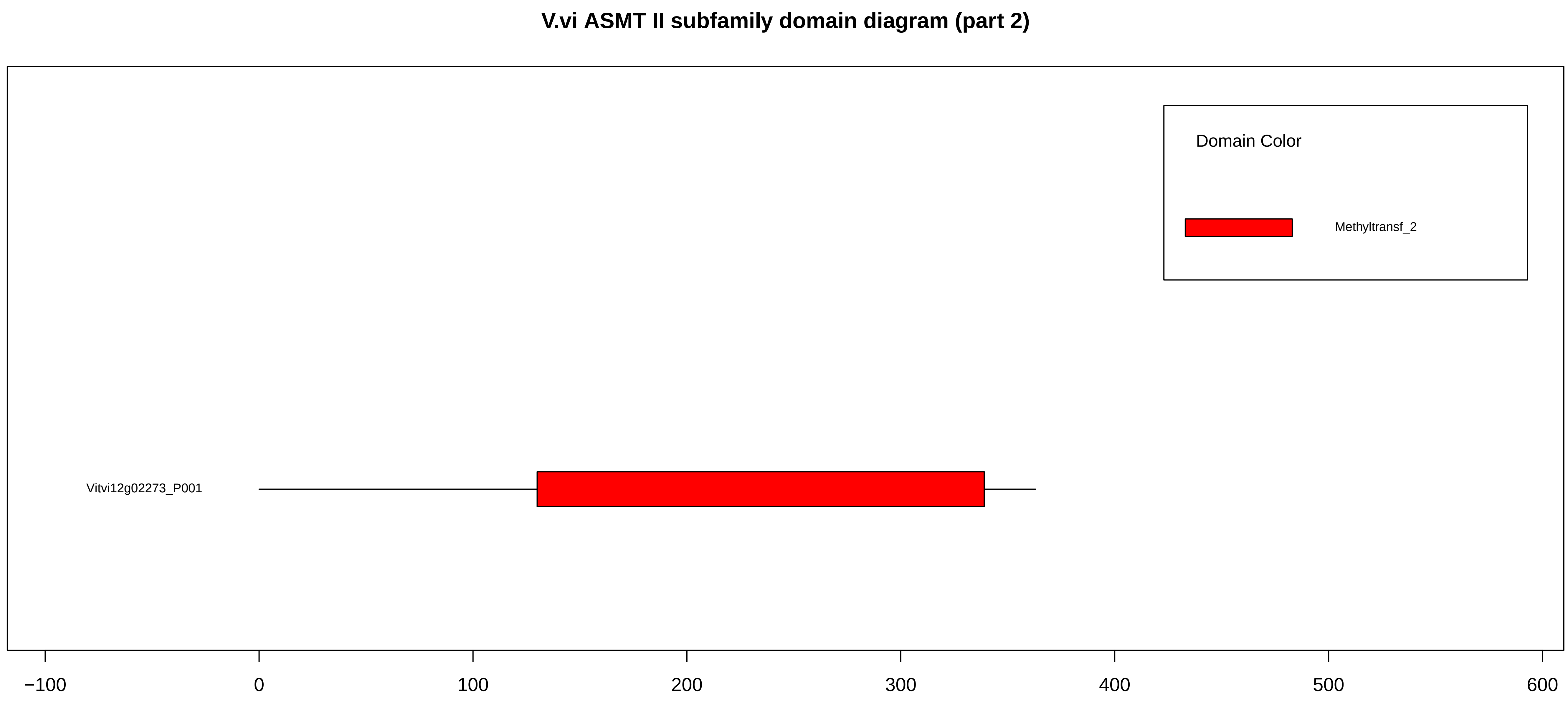
V.vi ASMT II subfamily domain diagram (part 2)
Domain Color
Methyltransf_2
Vitvi12g02273_P001
−100
0
100
200
300
400
500
600

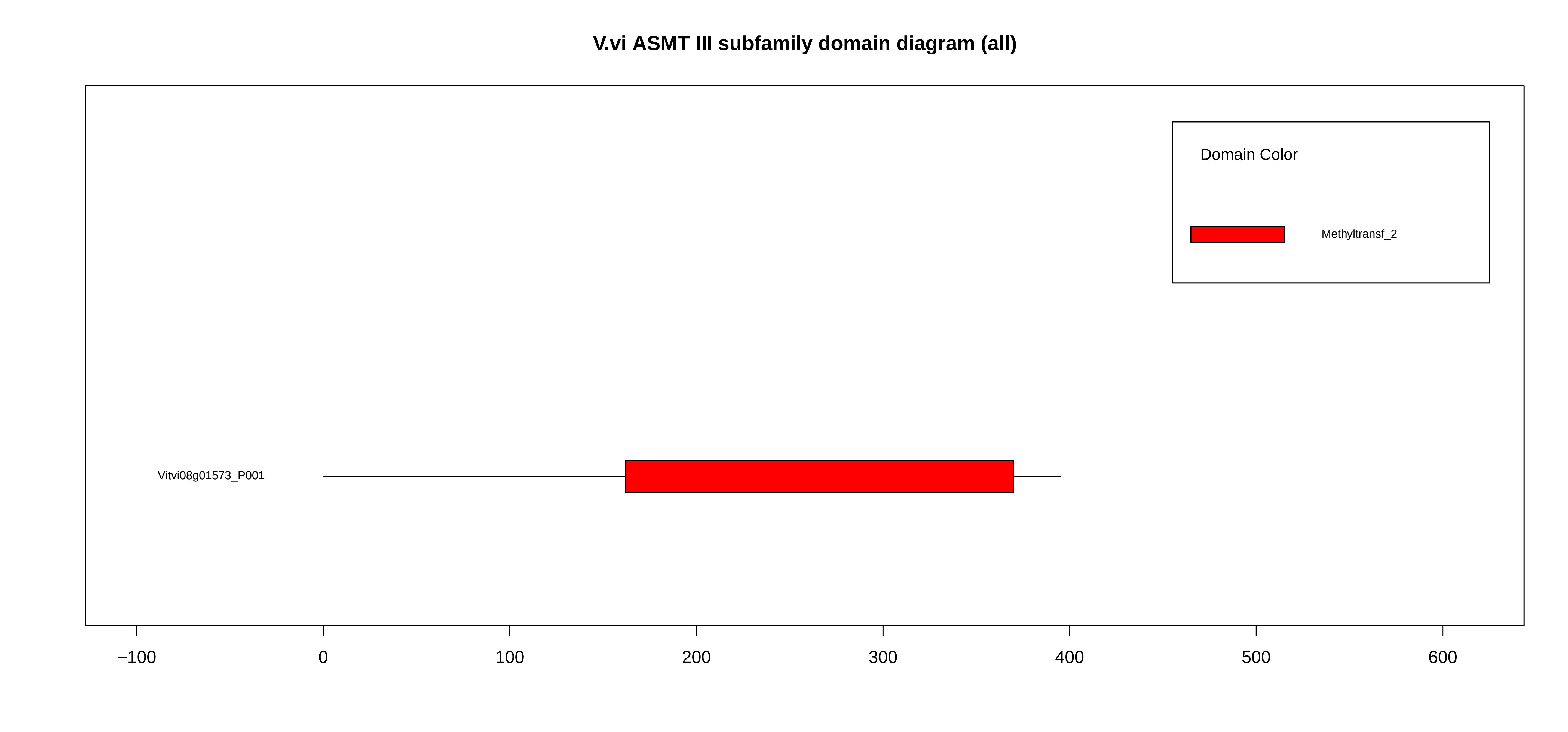
V.vi ASMT III subfamily domain diagram (all)
Domain Color
Methyltransf_2
Vitvi08g01573_P001
−100
0
100
200
300
400
500
600

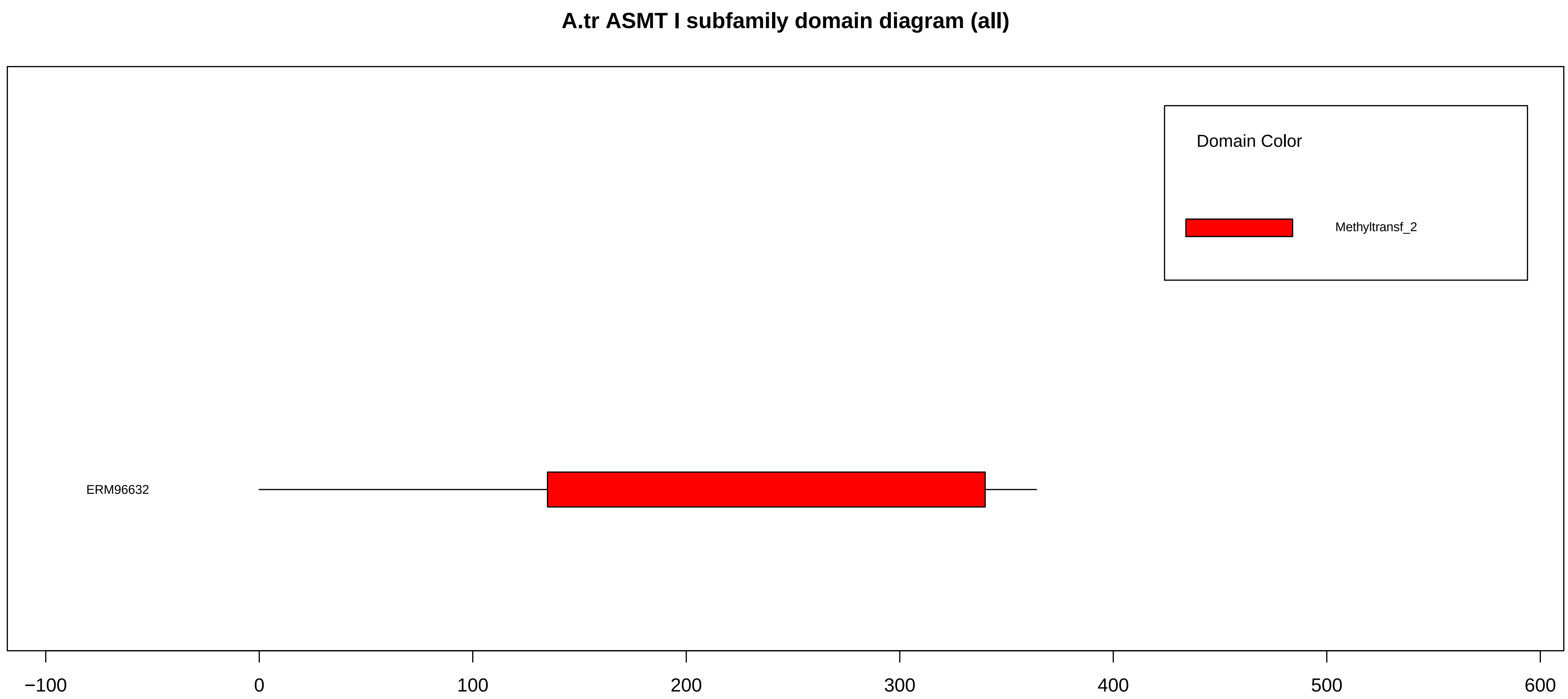
A.tr ASMT I subfamily domain diagram (all)
Domain Color
Methyltransf_2
ERM96632
−100
0
100
200
300
400
500
600

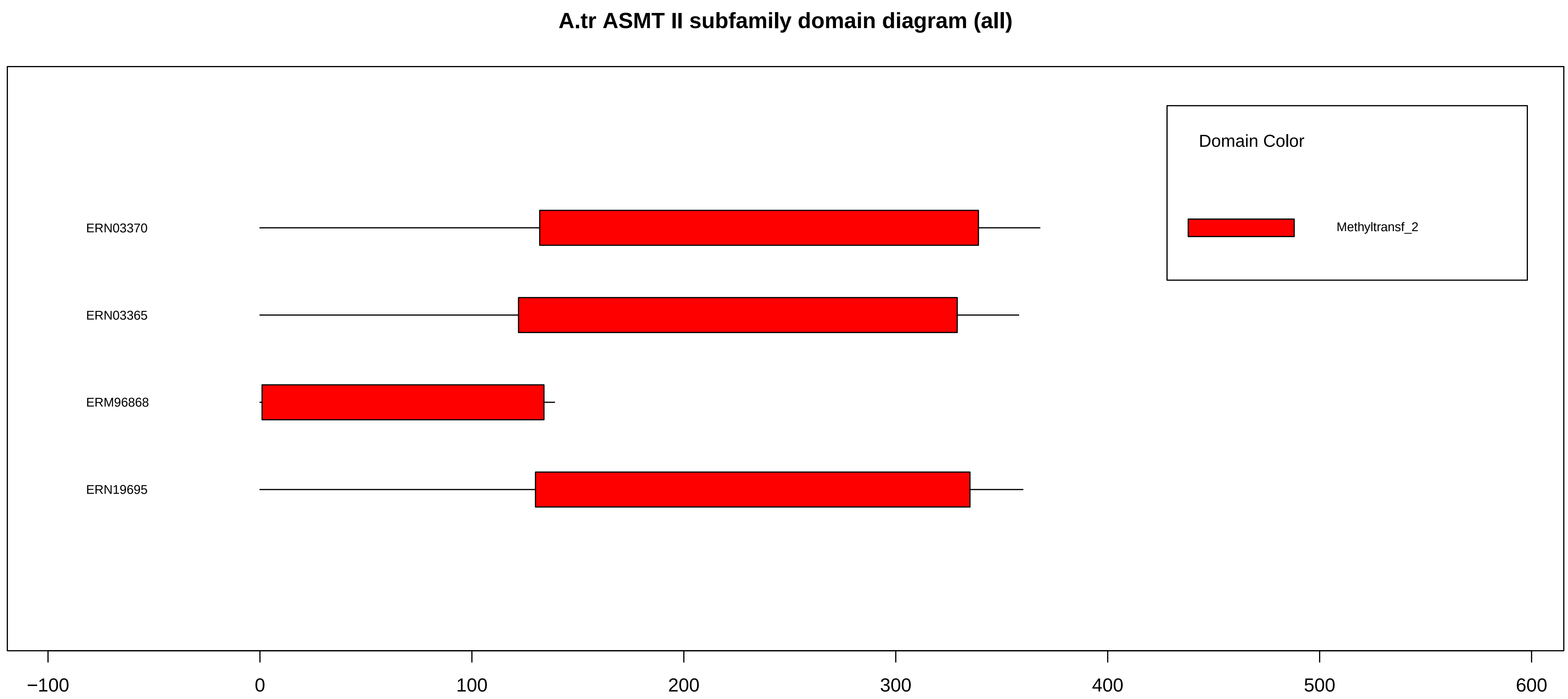
A.tr ASMT II subfamily domain diagram (all)
ERN03370
ERN03365
ERM96868
ERN19695
Domain Color
Methyltransf_2
−100
0
100
200
300
400
500
600

A.tr ASMT III subfamily domain diagram (all)

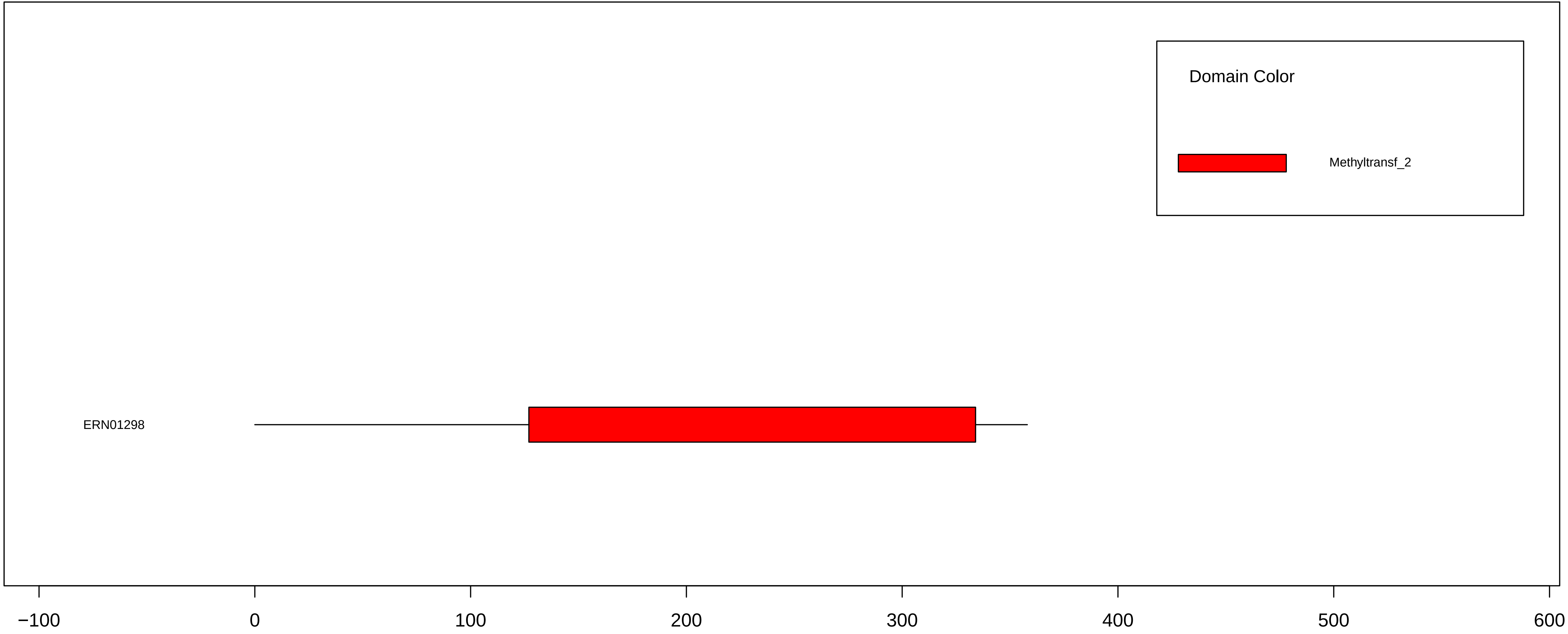

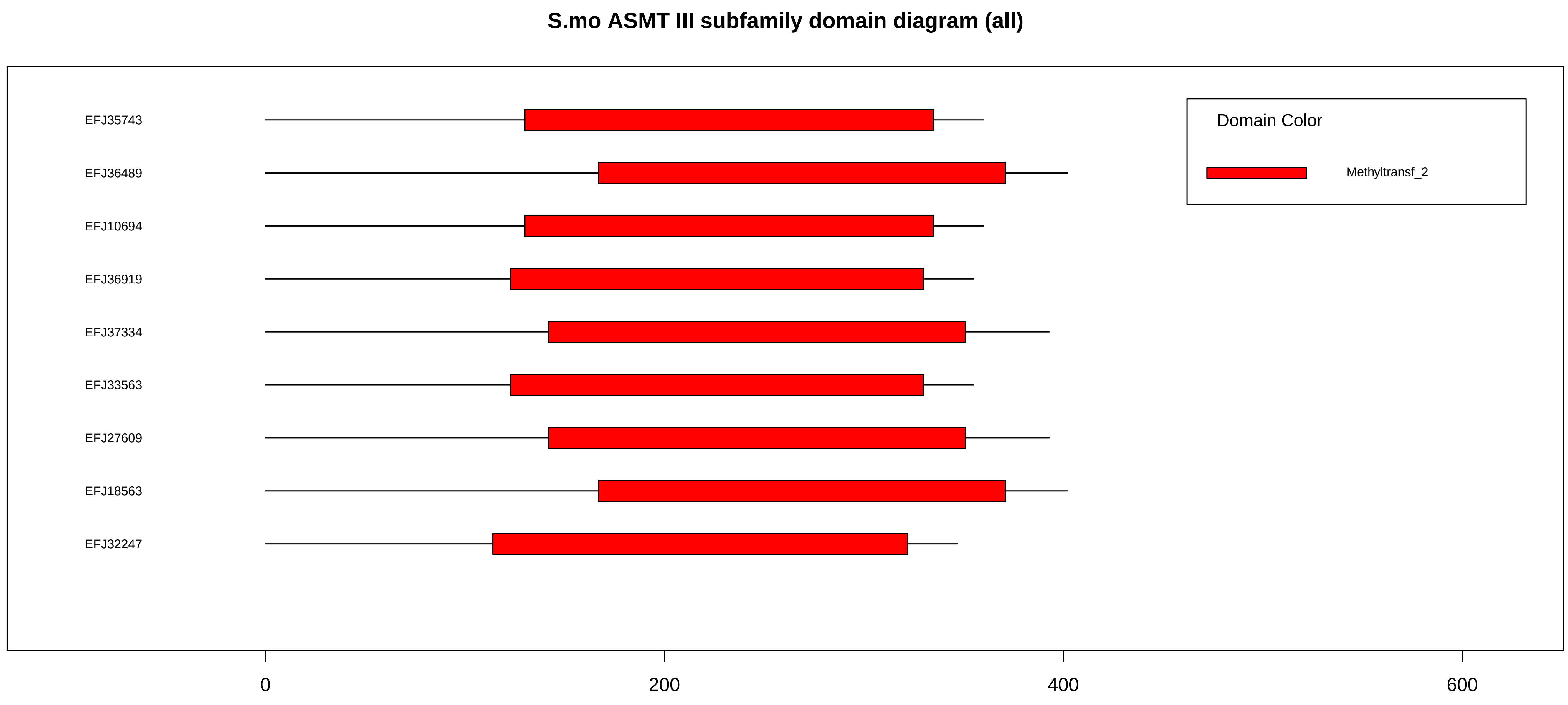
S.mo ASMT III subfamily domain diagram (all)
EFJ35743
EFJ36489
EFJ10694
EFJ36919
EFJ37334
EFJ33563
EFJ27609
EFJ18563
EFJ32247
Domain Color
Methyltransf_2
0
200
400
600

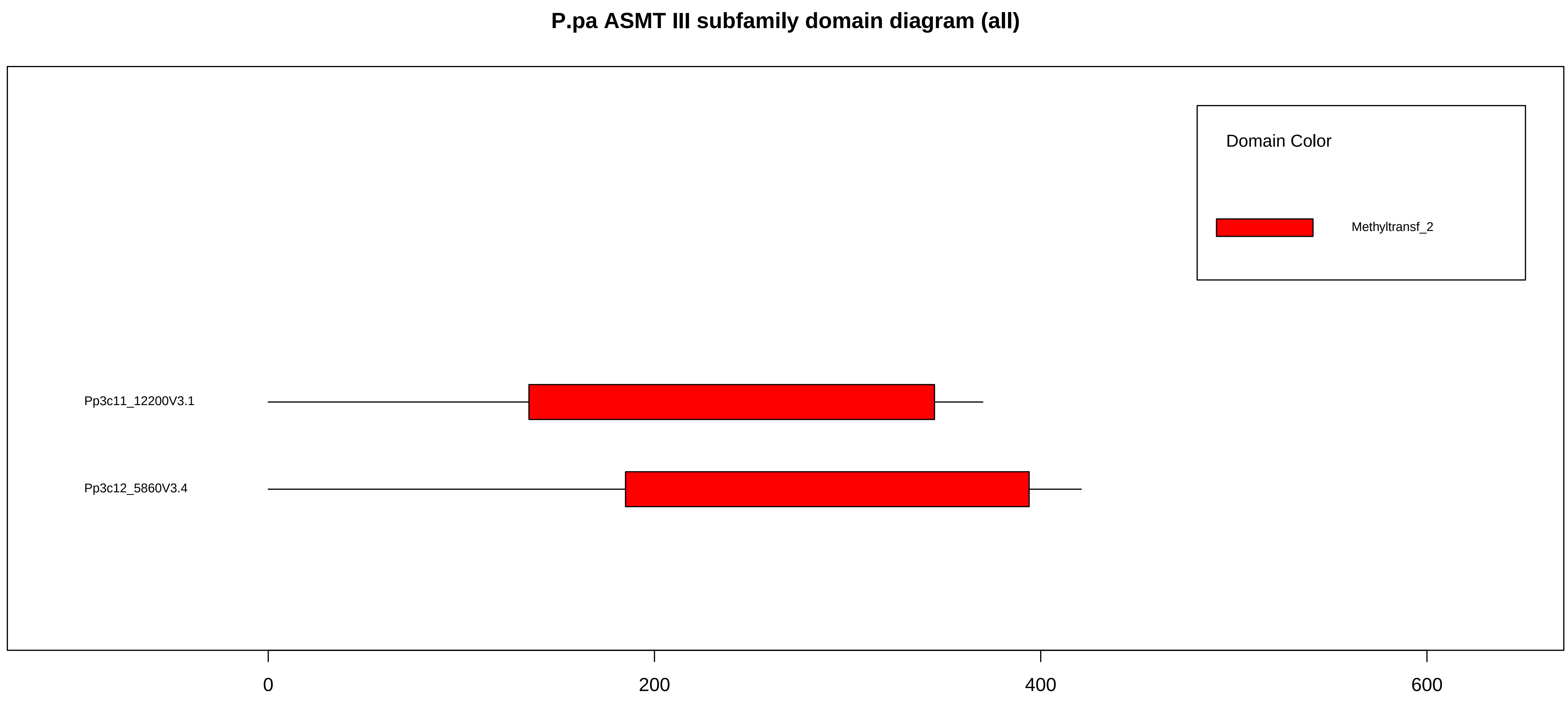

P.pa ASMT III subfamily domain diagram (all)
Domain Color
Methyltransf_2
Pp3c11_12200V3.1
Pp3c12_5860V3.4
0
200
400
600
