# Supplementary material for: Genome-Wide Identification of the ASMT Gene Family and Expression Analysis of Wheat ASMTs Under Abiotic and Biotic Stress
Source: Biology (Basel). 2026 Aug 19;15(16):1430. doi: 10.3390/biology15161430 (PMC13509220; doi:10.3390/biology15161430)
Supplement: Supplementary file 1 [file biology-15-01430-s001.zip › Figure/Figure S6.pdf]

Tae1A

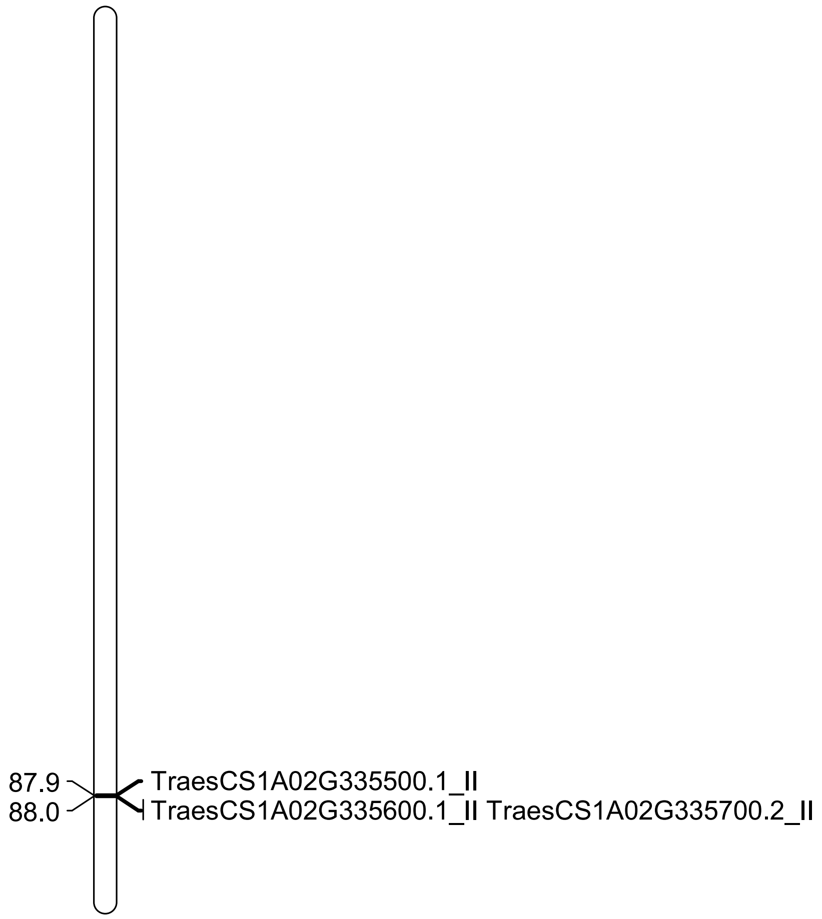
87.9
88.0
TraesCS1A02G335500.1_II
TraesCS1A02G335600.1_II TraesCS1A02G335700.2_II

Tae2A

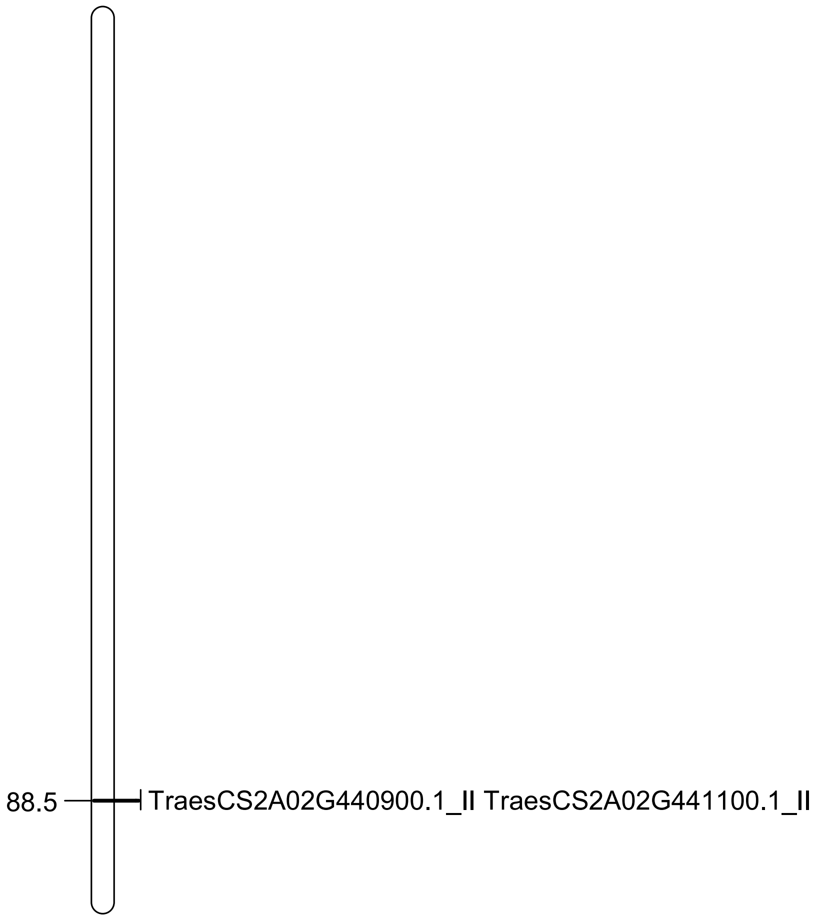
88.5
TraesCS2A02G440900.1_II TraesCS2A02G441100.1_II

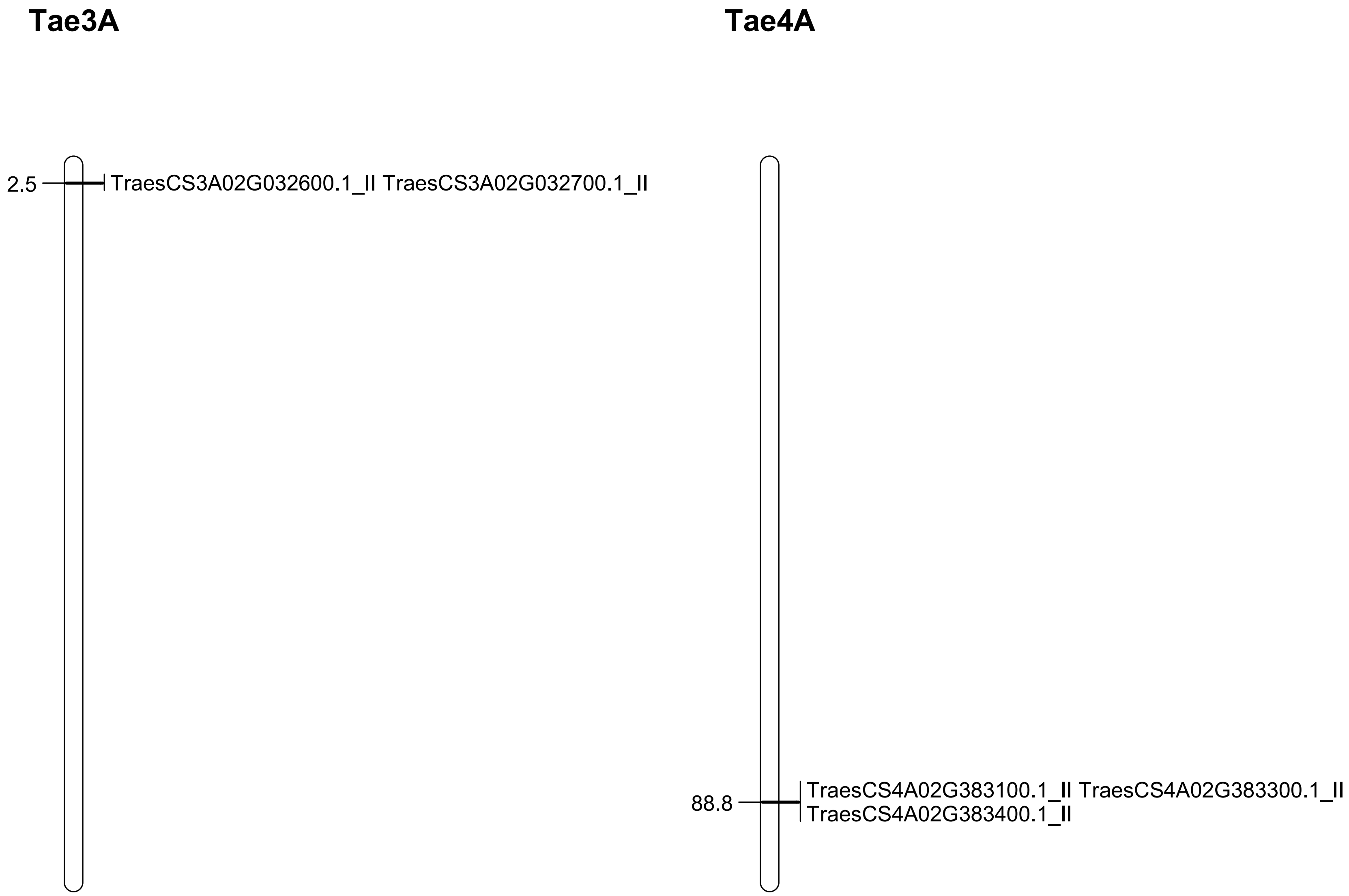
Tae3A
2.5
TraesCS3A02G032600.1_II TraesCS3A02G032700.1_II
Tae4A
88.8
TraesCS4A02G383100.1_II TraesCS4A02G383300.1_II
TraesCS4A02G383400.1_II

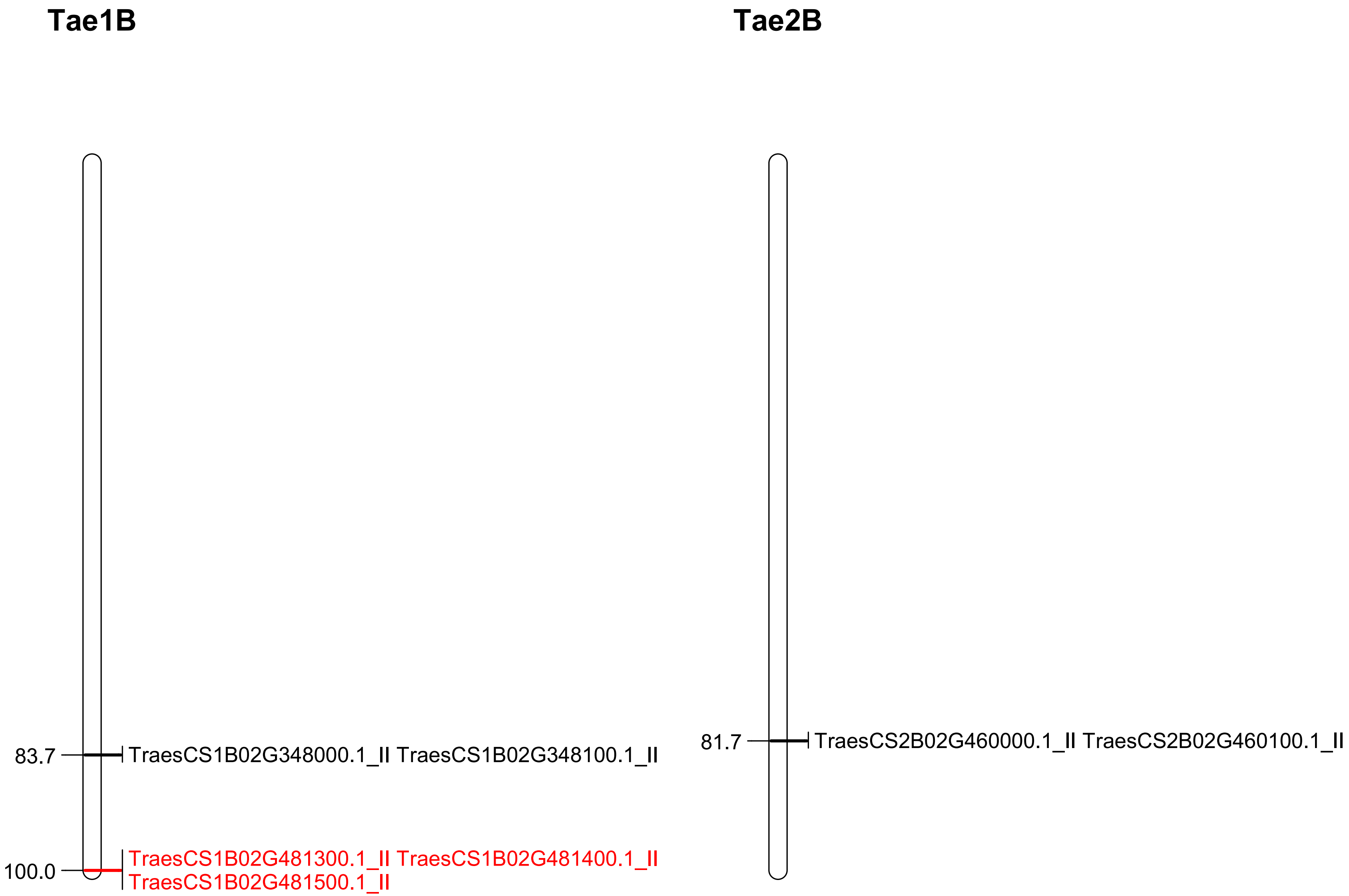
Tae1B
83.7
TraesCS1B02G348000.1_II TraesCS1B02G348100.1_II
100.0
TraesCS1B02G481300.1_II TraesCS1B02G481400.1_II
TraesCS1B02G481500.1_II
Tae2B
81.7
TraesCS2B02G460000.1_II TraesCS2B02G460100.1_II

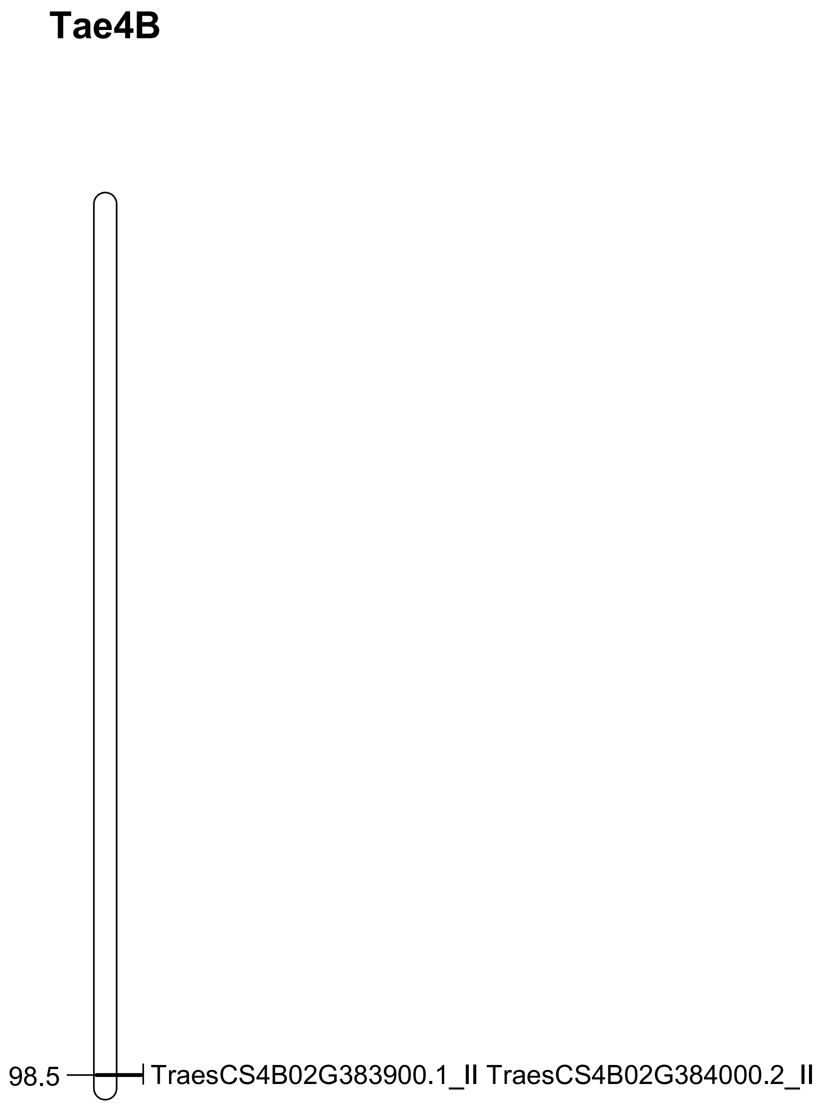
Tae4B
98.5
TraesCS4B02G383900.1_II TraesCS4B02G384000.2_II

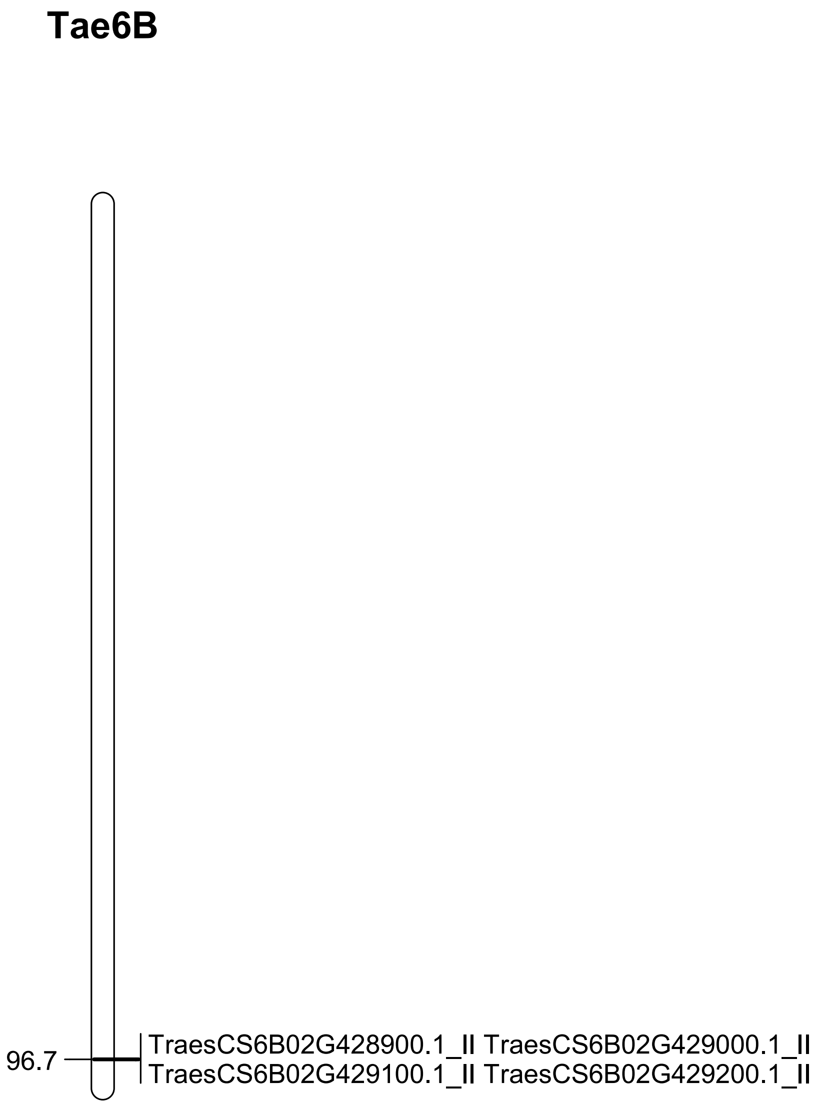
Tae6B
96.7
TraesCS6B02G428900.1_II TraesCS6B02G429000.1_II
TraesCS6B02G429100.1_II TraesCS6B02G429200.1_II

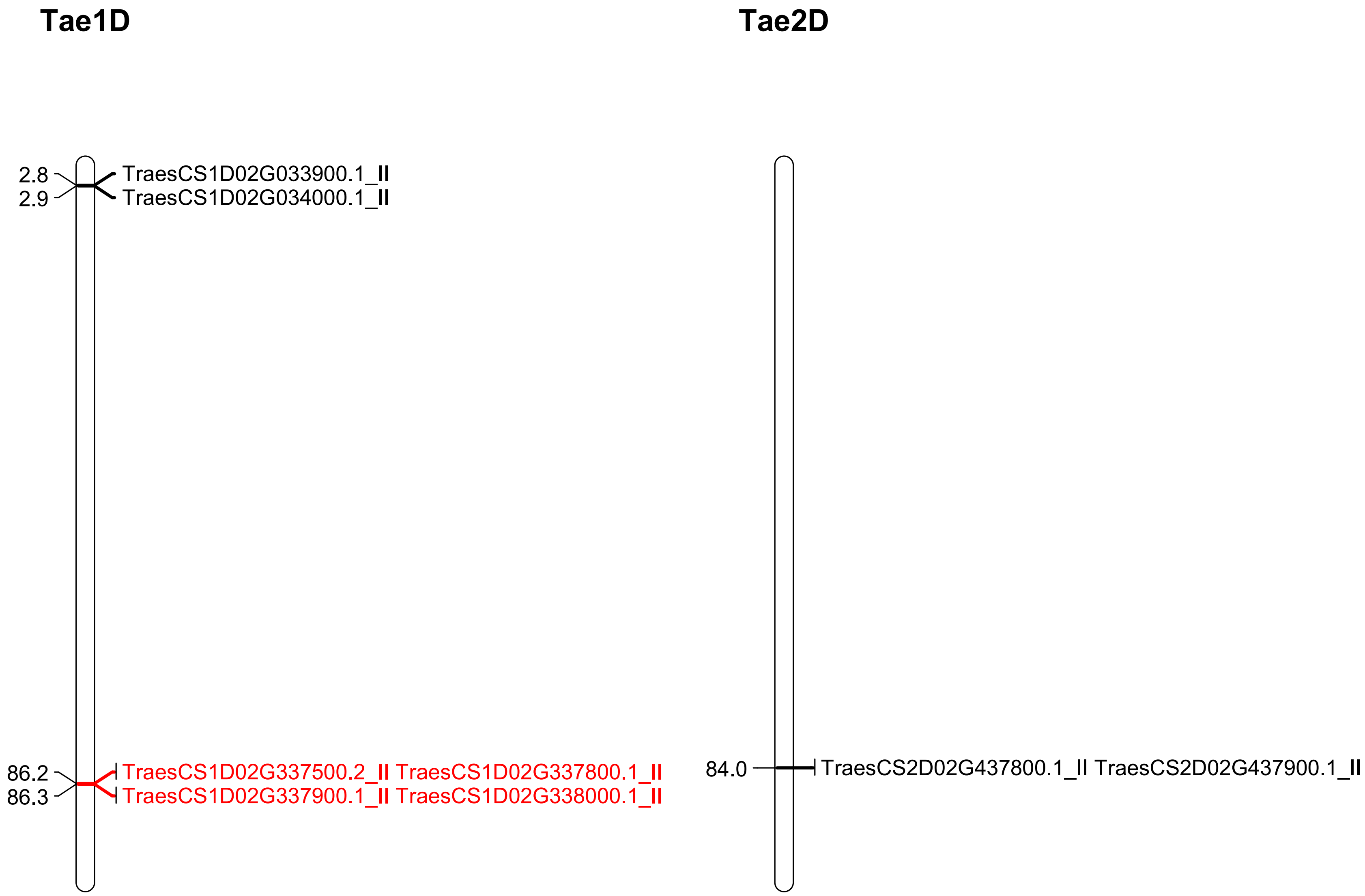
Tae1D
Tae2D
2.8
2.9
TraesCS1D02G033900.1_II
TraesCS1D02G034000.1_II
86.2
86.3
TraesCS1D02G337500.2_II TraesCS1D02G337800.1_II
TraesCS1D02G337900.1_II TraesCS1D02G338000.1_II
84.0
TraesCS2D02G437800.1_II TraesCS2D02G437900.1_II

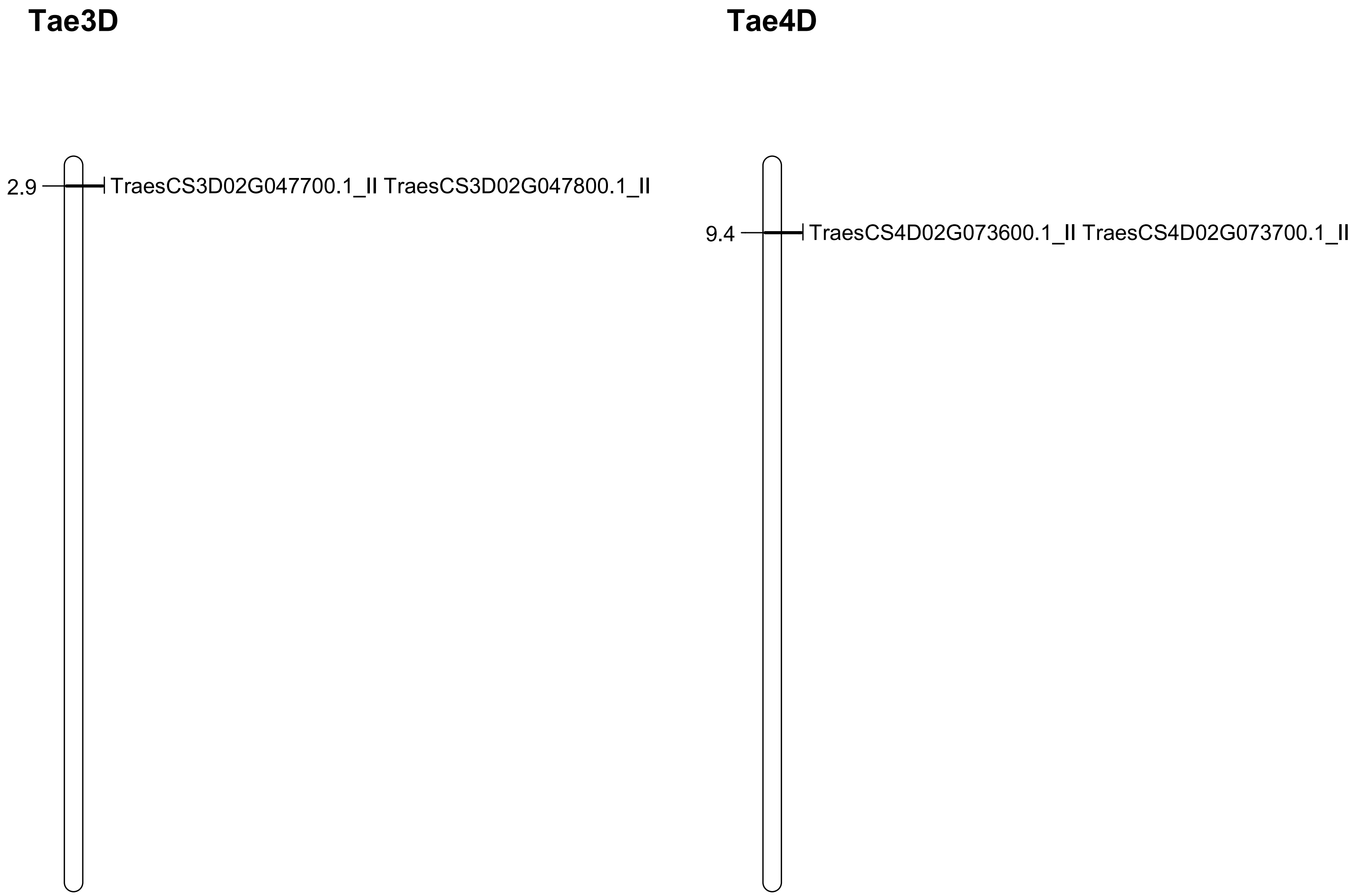
Tae3D
2.9
TraesCS3D02G047700.1_II TraesCS3D02G047800.1_II
Tae4D
9.4
TraesCS4D02G073600.1_II TraesCS4D02G073700.1_II

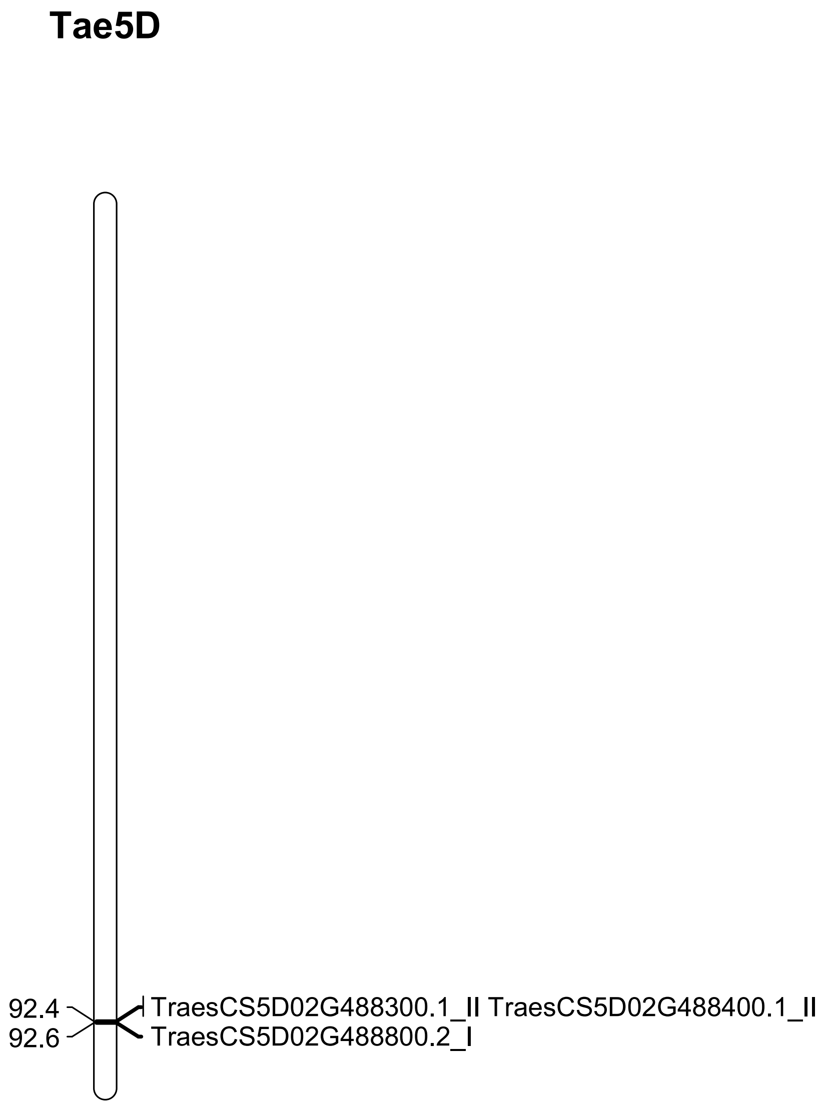
Tae5D
92.4
92.6
TraesCS5D02G488300.1_II TraesCS5D02G488400.1_II
TraesCS5D02G488800.2_I
