# Supplementary material for: Genome-Wide Identification of the ASMT Gene Family and Expression Analysis of Wheat ASMTs Under Abiotic and Biotic Stress
Source: Biology (Basel). 2026 Aug 19;15(16):1430. doi: 10.3390/biology15161430 (PMC13509220; doi:10.3390/biology15161430)
Supplement: Supplementary file 1 [file biology-15-01430-s001.zip › Figure/Figure S7.pdf]

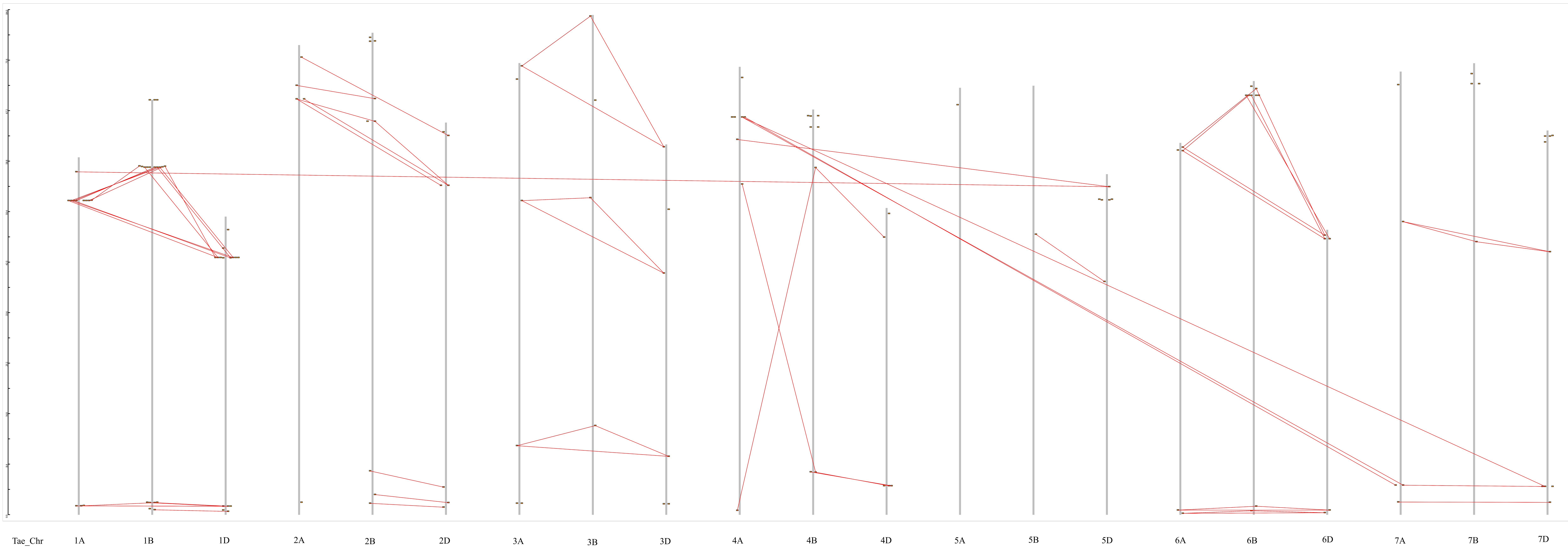

Tae_Chr
1A
1B
1D
2A
2B
2D
3A
3B
3D
4A
4B
4D
5A
5B
5D
6A
6B
6D
7A
7B
7D
