# Supplementary material for: Genome-Wide Identification of the ASMT Gene Family and Expression Analysis of Wheat ASMTs Under Abiotic and Biotic Stress
Source: Biology (Basel). 2026 Aug 19;15(16):1430. doi: 10.3390/biology15161430 (PMC13509220; doi:10.3390/biology15161430)
Supplement: Supplementary file 1 [file biology-15-01430-s001.zip › Figure/Figure S8.pdf]

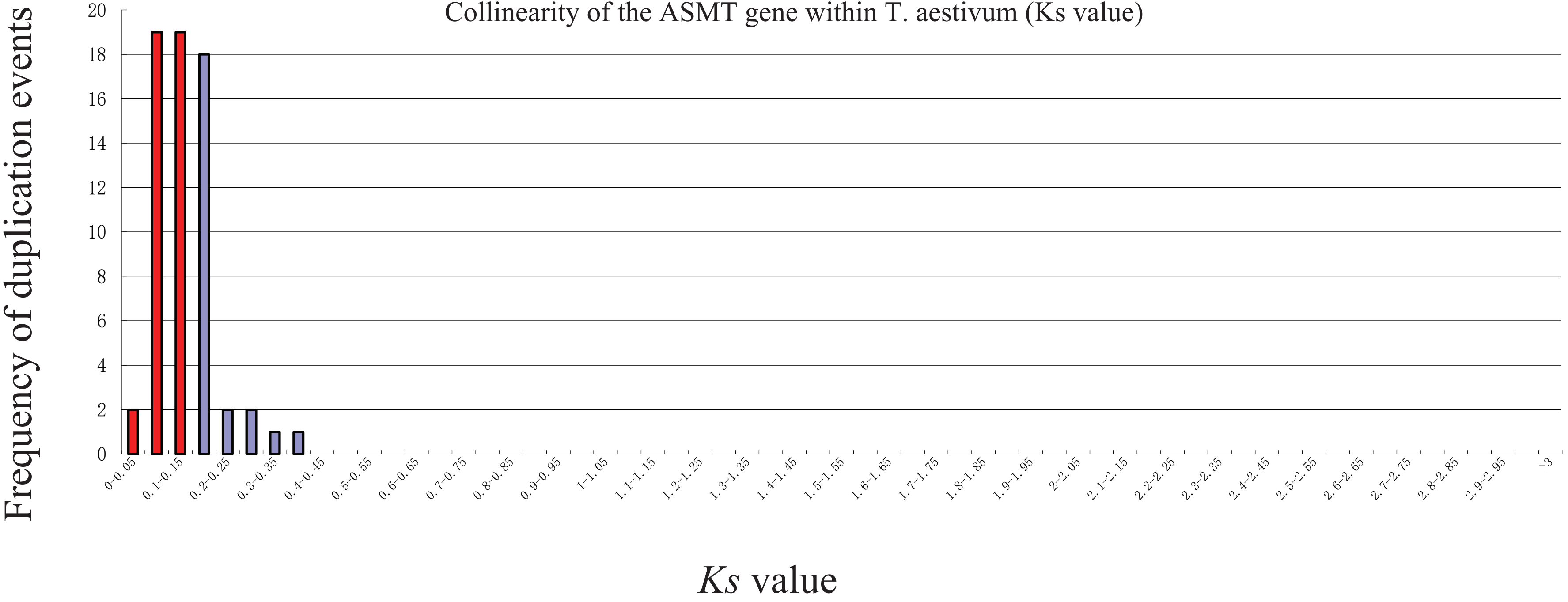
Collinearity of the ASMT gene within T. aestivum (Ks value)
Frequency of duplication events
Ks value
0
2
4
6
8
10
12
14
16
18
20
0-0.05
0.1-0.15
0.2-0.25
0.3-0.35
0.4-0.45
0.5-0.55
0.6-0.65
0.7-0.75
0.8-0.85
0.9-0.95
1-1.05
1.1-1.15
1.2-1.25
1.3-1.35
1.4-1.45
1.5-1.55
1.6-1.65
1.7-1.75
1.8-1.85
1.9-1.95
2-2.05
2.1-2.15
2.2-2.25
2.3-2.35
2.4-2.45
2.5-2.55
2.6-2.65
2.7-2.75
2.8-2.85
2.9-2.95
>3
