# Supplementary material for: Genome-Wide Identification of the ASMT Gene Family and Expression Analysis of Wheat ASMTs Under Abiotic and Biotic Stress
Source: Biology (Basel). 2026 Aug 19;15(16):1430. doi: 10.3390/biology15161430 (PMC13509220; doi:10.3390/biology15161430)
Supplement: Supplementary file 1 [file biology-15-01430-s001.zip › Figure/Figure S9.pdf]

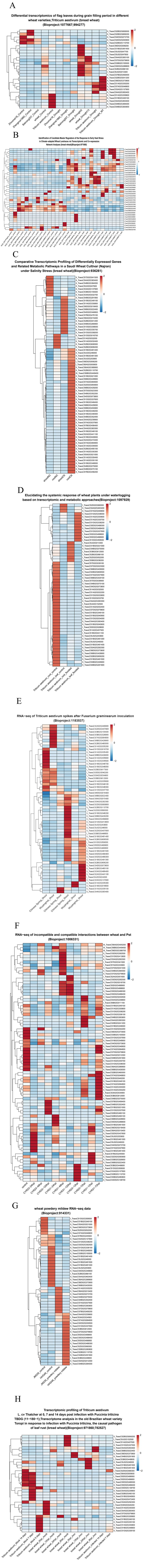
A
Differential transcriptomics of flag leaves during grain filling period in different wheat varieties;Triticum aestivum (bread wheat) (Bioproject:1077667;994277)
B
Identification of Candidate Master Regulators of the Response to Early Heat Stress in Climate−adapted Wheat Landraces via Transcriptomic and Co−expression Network Analyses (bread wheat)(Bioproject:971860)
C
Comparative Transcriptomic Profiling of Differentially Expressed Genes and Related Metabolic Pathways in a Saudi Wheat Cultivar (Najran) under Salinity Stress (bread wheat)(Bioproject:936261)
D
Elucidating the systemic response of wheat plants under waterlogging based on transcriptomic and metabolic approaches(Bioproject:1097929)
E
RNA−seq of Triticum aestivum spikes after Fusarium graminearum inoculation (Bioproject:1183537)
F
RNA−seq of incompatible and compatible interactions between wheat and Pst (Bioproject:1006331)
G
wheat powdery mildew RNA−seq data (Bioproject:914331)
H
Transcriptomic profiling of Triticum aestivum L. cv Thatcher at 0, 7 and 14 days post infection with Puccinia triticina TBDG (11−180−1);Transcriptome analysis in the old Brazilian wheat variety Toropi in response to infection with Puccinia triticina, the causal pathogen of leaf rust (bread wheat)(Bioproject:971860;782527)
